# Parallel Proteomic Comparison of Mutants With Altered Carbon Metabolism Reveals Hik8 Regulation of P_II_ Phosphorylation and Glycogen Accumulation in a Cyanobacterium

**DOI:** 10.1016/j.mcpro.2023.100582

**Published:** 2023-05-22

**Authors:** Chengcheng Huang, Xiaoxiao Duan, Haitao Ge, Zhen Xiao, Limin Zheng, Gaojie Wang, Jinghui Dong, Yan Wang, Yuanya Zhang, Xiahe Huang, Hongyu An, Wu Xu, Yingchun Wang

**Affiliations:** 1 State Key Laboratory of Molecular Developmental Biology, Institute of Genetics and Developmental Biology, Chinese Academy of Sciences, Beijing, China; 2 University of Chinese Academy of Sciences, Beijing, China; 3 Department of Chemistry, University of Louisiana at Lafayette, Lafayette, Louisiana, USA

**Keywords:** proteomics, S*ynechocystis*, carbon metabolism, cyanobacteria, phosphorylation

## Abstract

Carbon metabolism is central to photosynthetic organisms and involves the coordinated operation and regulation of numerous proteins. In cyanobacteria, proteins involved in carbon metabolism are regulated by multiple regulators including the RNA polymerase sigma factor SigE, the histidine kinases Hik8, Hik31 and its plasmid-borne paralog Slr6041, and the response regulator Rre37. To understand the specificity and the cross-talk of such regulations, we simultaneously and quantitatively compared the proteomes of the gene knockout mutants for the regulators. A number of proteins showing differential expression in one or more mutants were identified, including four proteins that are unanimously upregulated or downregulated in all five mutants. These represent the important nodes of the intricate and elegant regulatory network for carbon metabolism. Moreover, serine phosphorylation of P_II_, a key signaling protein sensing and regulating *in vivo* carbon/nitrogen (C/N) homeostasis through reversible phosphorylation, is massively increased with a concomitant significant decrease in glycogen content only in the *hik8*-knockout mutant, which also displays impaired dark viability. An unphosphorylatable P_II_ S49A substitution restored the glycogen content and rescued the dark viability of the mutant. Together, our study not only establishes the quantitative relationship between the targets and the corresponding regulators and elucidated their specificity and cross-talk but also unveils that Hik8 regulates glycogen accumulation through negative regulation of P_II_ phosphorylation, providing the first line of evidence that links the two-component system with P_II_-mediated signal transduction and implicates them in the regulation of carbon metabolism.

## Highlights

• Comparative proteomics for *Synechocystis* mutants defective in carbon metabolism.
• Identification of proteins and protein networks involved in carbon metabolism.
• P_II_ phosphorylation by Hik8 negatively regulates glycogen accumulation.

## Graphical Abstract

**Figure:**
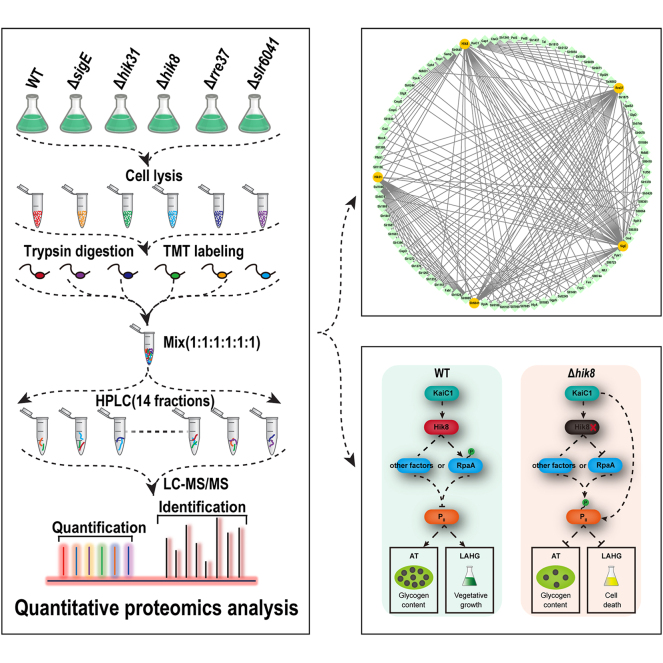

## In Brief

Comparative proteomics was simultaneously performed for five knockout mutants of *Synechocystis* each depleted of an important regulatory protein for carbon metabolism (RCM). Proteins uniquely or commonly regulated by the RCM proteins were identified. Particularly, dramatic hyperphosphorylation of P_II_ on S49 was also identified uniquely in the *hik8*-knockout mutant that is concomitant with significantly reduced glycogen content. Further experimental evidence suggests that Hik8 regulates glycogen accumulation through negative regulation of P_II_ phosphorylation.

Cyanobacteria are oxygenic photoautotrophic organisms inhabiting a wide range of environments (1). They are key players in shaping the current global ecosystem by converting CO_2_ into a utilizable carbon resource while evolving oxygen from the water through photosynthesis. Because of their resemblance to the chloroplast in the higher plants, a number of cyanobacteria strains have been effectively used as the model for photosynthesis research. More recently, cyanobacteria gained more interest because of their potential as the unique chassis (green *Escherichia coli*) for synthetic biology and the cell factory to produce clean and renewable biofuels (2, 3).

Photosynthesis and carbon metabolism are two physically and functionally interconnected processes in cyanobacteria. In addition, nitrogen assimilation and metabolism are also interwoven and coordinated with photosynthesis and carbon metabolism. The light reaction of photosynthesis occurs on the thylakoid membrane, whereas CO_2_ fixation occurs in a specialized non-membranous microcompartment formed by multiple proteins, namely, carboxysome (4). In contrast, central carbon metabolism and nitrogen assimilation mainly occur in the cytoplasm. Many cyanobacteria strains can grow photoautotrophically (AT) using CO_2_ as the sole carbon source or photoheterotrophically (PHT) using the exogenously supplied organic form of carbon such as glucose or photomixotrophically (MT) using both as the carbon source. Under AT growth mode, CO_2_ is fixed *via* the Calvin cycle and hexose-phosphates are produced through gluconeogenesis. These sugar products are subsequently converted into storage carbohydrates, such as glycogen (5). Endogenously synthesized or exogenously supplied glucose can also be catabolized through the oxidative pentose phosphorylation pathway (OPPP) and/or glycolysis to supply carbon and energy for the growth of cyanobacteria (6, 7). In cyanobacteria, a number of proteins in sugar catabolic pathways are indispensable for heterotrophic growth in dark. For example, the mutants deficient with glucose-6-phosphate dehydrogenase (Zwf) or glyceraldehyde 3-phosphate dehydrogenase 1 (Gap1) are defective in viability in the dark and unable to carry out light-activated heterotrophic growth (LAHG) (8). As the Calvin cycle, OPPP, and glycolysis take place within a single cellular compartment, many reversible reactions, enzymes, regulators, and intermediate metabolites are shared by the three processes. Moreover, the processes are physically and functionally connected with the tricarboxylic acid cycle (TCA) cycle, nitrogen assimilation, amino acid and protein metabolism, lipid metabolism, and many other metabolic processes. A single perturbation of one process may generate significant consequences on the activities and outcomes of the others. For example, the addition of glucose to a cyanobacterium culture can enhance the activity of OPPP while partly repressing the activity of photosynthesis (9). In addition, the gluconeogenic activity of the upper part of glycolysis enhances the flux of the OPPP to supply NADPH depleted in the dark (10). The metabolic pathways, particularly the central carbon metabolism that interconnects both with photosynthesis and the other metabolic pathways such as nitrogen assimilation, must be intricately and precisely regulated.

Multiple proteins have been reported as important regulators of carbon metabolism in *Synechocysti*s sp. PCC 6803 (hereafter referred to as *Synechocystis*), including Hik8 (Sll0750), Hik31 (Sll0790), and its plasmid paralog Slr6041, Rre37 (Sll1330), and SigE (Sll1689) (hereafter collectively named as the regulatory proteins for carbon metabolism, RCM) (11, 12, 13, 14, 15, 16, 17). The histidine kinases Hik8 and Hik31/Slr6041 are sensors of the two-component systems (18, 19). The ortholog of Hik8 (SasA) and its cognate response regulator RpaA in *Synechococcus elongatus* PCC 7942 (hereafter referred to as *Synechococcus*) are output components of the circadian clock and regulate global gene expression with circadian rhythmicity (20, 21). Rre37 is a response regulator of a two-component system (11), and SigE is a group 2 sigma factor of bacterial RNA polymerase (11, 12, 13, 14, 15, 16, 17, 22, 23). A plethora of proteins involved in sugar catabolic pathways such as OPPP and glycolysis are downregulated in the RCM-deficient cyanobacterial mutants, indicating that they are positively regulated by the RCM proteins. Nevertheless, for each individual protein in these pathways, its expression could be either commonly regulated by more than one RCM protein with the same or different extent of regulations or uniquely regulated by only one RCM protein. Such regulatory specificity and cross-talk may warrant the plasticity and robustness of carbon metabolism, which is necessary for maintaining metabolic and energy homeostasis to fine-tune cyanobacteria growth in response to environmental and metabolic cues. For example, nitrogen starvation can induce the expression of a number of proteins involved in sugar catabolism through the operation of one or more of the RCM proteins (24, 25, 26). Unfortunately, since the studies regarding the RCM proteins were conducted by different groups, at different time, and with different experimental conditions (15, 17, 18, 27), it is difficult to quantitatively and qualitatively evaluate such regulatory specificity and the cross-talk. The scenario may become even more complicated when concomitant differential expression of proteins in other metabolic pathways is involved. It is also important to simultaneously identify such proteins and quantitatively determine the extent of their differential expression in the RCM-deficient mutants.

Herein, we tried to address this problem by quantitatively and simultaneously identifying the proteins regulated by the five RCM proteins in the model organism *Synechocystis*. *Synechocystis* is one of the most extensively studied model cyanobacteria for photosynthesis (28, 29). It is the first cyanobacterium with a completely sequenced genome and is highly transformable (30). We generated knockout mutants for the five RCM protein-coding genes, and then quantitatively and simultaneously compared the proteomes across the five knockout mutants using a tandem-mass tag (TMT) labeling-based quantitative proteomics approach. Proteins differentially expressed in one or more of the mutants were identified and quantitatively compared. Novel insights into the regulation of carbon metabolism were provided from the result.

## Experimental Procedures

### Antibodies

The primary antibodies for Gnd, Pgk, KaiC1, PntA, Zwf, and GltB were provided by PhytoAB. The antibodies for AtpB, ClpB2, Prk, PsaD, and SbtA were purchased from Agrisera.

### Cell Culture

The wild type (WT) and mutant strains of *Synechocystis* were cultured in liquid BG-11 medium in medium light (50 μmol m^−2^ s^−1^ photons) with shaking at 30 °C. The cells were collected by centrifugation (4000*g* for 10 min) for biochemical and proteomic analyses when the culture reaches the optical density at 730 nm (OD_730_) of approximately 1.0. The optical density measures the turbidity of the cell culture (31), which positively correlates with cell density. The harvested cells were stored at −80 °C until use. For MT growth, 5 mM glucose was supplemented to the medium. For PHT growth, cells were cultured in the same condition as that used for MT growth except that 5 μM DCMU was added to the culture medium. For LAHG growth, the cells were grown in darkness in the presence of 5 mM glucose and a daily pulse of white light (at least 5 min). The starting concentrations of cells at OD_730_ of 0.05 and 0.2 were used for growth curve measurements under the phototrophic and the LAHG conditions, respectively.

### Mutants Generation

The gene knockout mutants were generated through insertional mutation and homologous recombination using the procedures as we described (13, 32).

To generate the strains carrying P_II_ S49A mutation, a *glnB* (P_II_ coding gene) knockout strain of *Synechocystis* was first generated as above described (Δ*glnB*). A DNA fragment containing wild-type *glnB* and its native promoter or a mutant copy carrying P_II_ S49A substitution was inserted in the chromosome of the Δ*glnB* mutant at a site designated “neutral site” to generate the WT equivalent (WT^e^) strain or *glnB*^S49A^/WT^e^ strain (33, 34). The insertional mutation of *hik8* in the *glnB*^S49A^/WT^e^ background was subsequently performed as above described to generate the mutant *glnB*^S49A^/WT^e^/Δ*hik8*. All mutants were selected and confirmed as described earlier, and all primers used were included in supplemental Table S1.

### Pigment Analysis

The Chl and total carotenoids in cells were extracted with N, N-dimethylformamide (DMF, Sigma-Aldrich, Saint Louis, MO), and the concentrations were determined using a previously described approach (35). The equations used for the calculation of pigment concentration are:

$$Chl\;a\;\left(\mu g/mL\right)=12.1\times {OD}_{664}\;-\;0.17\times {OD}_{625}\text{.}$$

$$Total\;Carotenoids\;\left(\mu g/mL\right)=\left({OD}_{461}\;-\;0.046\times {OD}_{664}\right)\times 4\text{.}$$

### 77K Fluorescence Measurement

77K fluorescence emission spectra were measured using an F-7000 Fluorescence Spectrophotometer (Hitachi). Cells were adjusted to a concentration of 15 μg Chl mL^−1^, and the fluorescence emission spectra (excitation at 435 nm, bandwidth of 5 nm) were recorded in the range of 650 to 800 nm and were normalized at 726 nm.

### Glycogen Content Determination

Glycogen was determined as described (36). For each measurement, 20 mL of exponentially growing *Synechocystis* cells (OD_730_ = 1) were harvested, resuspended in 400 μL 30% (w/v) KOH, and incubated at 95 °C for 2 h. Glycogen was then precipitated with 75% (v/v) ice-cold ethanol and collected by centrifugation at 10,000*g* for 10 min. The pellet was sequentially washed with 70% and 98% (v/v) ethanol and dried at 60 °C for 10 min. The isolated glycogen was resuspended in 100 mM sodium acetate (pH 4.5) and enzymatically hydrolyzed to glucose with 2 mg/mL amyloglucosidase (Sigma-Aldrich) at 60 °C for 2 h. The glycogen content was determined with a glucose assay kit (Sigma-Aldrich) according to the manufacturer’s instructions.

### Protein Preparation

Cell pellets were lysed in a buffer containing 0.4 M sucrose, 50 mM MOPS, 10 mM NaCl, 5 mM EDTA (pH 7.0), and 0.5 mM PMSF with a bead beater, and the insoluble debris was removed by centrifugation for 30 min at 5000*g* at 4 °C. After precipitation with ice-cold 10% trichloroacetic acid in acetone at −20 °C, total proteins were washed with acetone and resolubilized with 4% sodium dodecyl sulfate (SDS) in 0.1 M Tris-HCl, pH 7.6. A BCA protein assay kit (Thermo Scientific) was used to determine the protein concentration.

### Protein Digestion and TMT Labeling

Proteins were digested using the filter-aided sample preparation (FASP) method according to a previously described method with slight modifications (32, 37). Briefly, the lysates (100 μg protein for each sample) were reduced with 10 mM DTT at 37 °C for 1 h and alkylated with 55 mM iodoacetamide (IAA, Sigma-Aldrich) at room temperature for 1 h in the dark. The alkylated lysates were transferred into the Microcon YM-30 centrifugal filter units (EMD Millipore Corporation), where the denaturing buffer was replaced by the 0.1 M triethylammonium bicarbonate (TEAB, Sigma-Aldrich, Saint Louis, MO), and then digested with sequencing grade trypsin (Promega) at 37 °C overnight. The resulting tryptic peptides were collected and labeled with 6-plex TMT reagents (Thermo Scientific) by incubating peptides with ethanol-dissolved TMT reagents for 2 h at room temperature in the dark. The labeling reaction was inactivated by the addition of 5% hydroxylamine, and the labeled samples were mixed together with equal ratios before being fractionated with reversed-phase (RP) high-performance liquid chromatography (HPLC).

### RP-HPLC and Desalting

Offline basic RP-HPLC was performed using a Waters e2695 separations HPLC system coupled with a Phenomenex gemini-NX 5 μ C18 column (250 × 3.0 mm, 110 Å). The sample was separated with a 97 min basic RP-LC gradient as previously described (38). A flow rate of 0.4 mL/min was used for the entire LC separation. The separated samples were collected into 14 fractions, completely dried with a Speed-Vac concentrator, and stored at −20 °C. The dried peptides were resolubilized with 0.5% acetic acid and desalted using C18 Stage Tips (39). Desalted peptides were dried with a Speed-Vac concentrator, stored at −20 °C, and resuspended in 0.1% formic acid (FA) immediately before LC-MS/MS.

### MS Analysis

For MS analysis, the peptides were resuspended in 0.1% FA and analyzed by a LTQ Orbitrap Elite mass spectrometer (Thermo Scientific) coupled online to an Easy-nLC 1000 in the data-dependent mode. Briefly, 2 μL of peptide sample (1 μg/μL) was injected into a 25-cm length, 75-μm inner diameter capillary analytic column packed with C18 particles of 5-μm diameter (SunChrom, Friedrichsdorf, Germany). The mobile phases for the LC include buffer A (2% acetonitrile, 0.1% FA) and buffer B (98% acetonitrile, 0.1% FA). The peptides were separated using a 90-min non-linear gradient consisting of 3% to 8% B for 10 min, 8% to 20% B for 60 min, 20% to 30% B for 8 min, 30% to 100% B for 2 min, and 100% B for 10 min at a flow rate of 300 nL/min. The source voltage and current were set at 2.5 KV and 100 μA, respectively. All MS measurements were performed in the positive ion mode. The precursors were measured by survey scans in the Orbitrap with a mass range of 300 to 1800 m/z at a resolution of 120,000 at m/z 400. The 15 most abundant precursor ions (top15) from each MS scan were isolated and fragmented by high-energy collisional dissociation (HCD) with 40% collision energy for MS/MS analysis using DDA mode. The automatic gain control (AGC) and maximum injection time were set at 1 × 10^6^ and 200 ms, respectively. The MS/MS spectra were acquired at a resolution of 15,000 at m/z 400 in the Orbitrap. The duration of dynamic exclusion was set to 30 s to prevent repeat identification of peptide ions within the time duration.

The PRM experiment was performed as described (40). PRM and DDA for the PRM spectral library generation were performed using Orbitrap Lumos (Thermo Fisher Scientific). The full scan mass range was 300 to 1500 m/z and the Orbitrap resolution was 120,000 at 200 m/z. The AGC target and maximum injection time were set at 1 × 10^6^ and 30 ms, respectively. For DDA, the mass spectrometer was operated in data-dependent acquisition mode with a maximum duty cycle time of 3 s, the isolation window was 1.6 m/z, and the MS/MS spectra were acquired at a resolution of 30,000 at 200 m/z in the Orbitrap. The duration of dynamic exclusion was set to 30 s. For PRM, target precursor ions (target peptide list) (supplemental Table S6) were fragmented using HCD fragmentation with 32% collision energy and detected with Orbitrap at a mass resolution of 30,000 at 200 m/z, the isolation window was 1.4 m/z.

### Database Search

The raw MS files were searched against the *Synechocystis* proteome sequence database using the software MaxQuant (version 1.5.4.1) (41). The database containing 3672 entries was downloaded from the CyanoBase (ftp://ftp.kazusa.or.jp/pub/CyanoBase/*Synechocystis*, released on 5/11/2009). The type of search was set to report ion MS2 and the 6-plex TMT was chosen, the minimum reporter parent ion interference was set to 0.75. Trypsin was chosen as the protease for protein digestion, and the maximum of 2 was set as the allowable miscleavages. N-terminal acetylation and methionine oxidation were chosen as the variable modifications and cysteine carbamidomethylation was chosen as the fixed modification. The mass tolerances were set to 4.5 ppm for the main search and 20 ppm for precursor and fragment ions. The minimum score for unmodified peptides and modified peptides were set to 15 and 40, respectively. Other parameters were set up using the default values. The false discovery rate (FDR) was set to 0.01 for both peptide and protein identifications.

### Experimental Design and Statistical Rationale

**Fig. 1:**
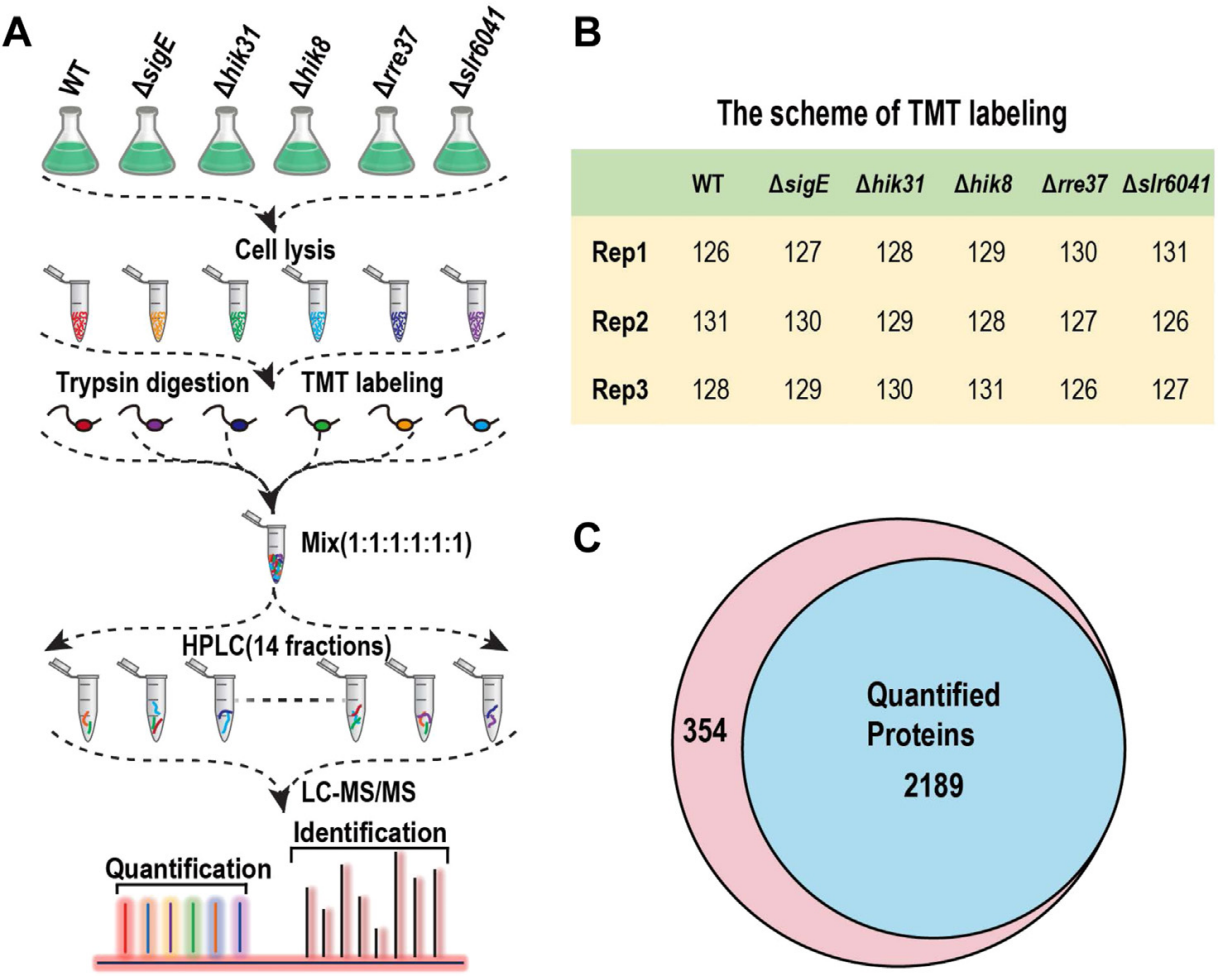
**Quantitative identification of *Synechocystis* proteomes among the WT and regulatory proteins for carbon metabolism (RCM) mutant strains under photoautotrophic (AT) condition.***A*, Schematic representation of the strategy for quantitative analysis of the *Synechocystis* proteomes using the TMT-labeling-based quantitative proteomics approach. Three biological replicates were included. *B*, The scheme of TMT labeling. Note that the orders of the TMT labeling are different among the replicates. *C*, Venn diagram shows the numbers of the total identified and quantified proteins.

Triplicated TMT labeling-based quantitative proteomic experiments were performed to analyze the differentially expressed proteins among the WT *Synechocystis* and the five RCM mutants, thereby covering three biological replicates of each sample. In each experiment, tryptic peptides from each sample were labeled with a distinct TMT reagent of the 6-plex TMT reagent in an alternating order as shown in Figure 1*B* to reduce quantitative bias (32).

Bioinformatic and statistical analyses were mainly performed using the software Perseus (version 1.5.4.1) (42). Student’s *t* test was used to determine the significance of differential expression of proteins, and Fisher’s-exact test was used for the functional enrichment analysis. A *p* value < 0.05 was used as the cutoff for all statistical analyses.

For the PRM experiment, four target peptides were selected (supplemental Table S6), which were the phosphopeptides bearing S49 phosphorylation of the protein P_II_ and their non-phosphorylated counterparts (including all peptides resulting from miscleavages). The non-phosphorylated counterparts were used as the internal control because they are expected to show the opposite direction of change in abundances to that of the corresponding phosphopeptides, given that the abundance of P_II_ is not changed among the samples to be compared (supplemental Figs. S9–S13). Three biological replicates were included to quantify the target peptides in AT/MT-grown WT and Δ*hik8*.

PRM data (Tier 2 level) were processed with Skyline (version 21.2.0.369) software (43). The DDA library was generated using msms.txt from MaxQuant (version 1.6.0.16) (44). A target peptide with a higher DOPT value (at least 0.8 in an experiment) was selected if the target peptide has two different charge states. The top 6 transitions of the target peptides (supplemental Table S7) were selected, peak picking was performed and manually inspected, and the peak area of the transitions was summed for quantification.

Student’s *t* test was used to determine the significance of the abundance difference of the target peptides. Standard deviation was used to confirm the precision of target peptides.

## Results

### Generation and the Growth Experiment of the Knockout Mutants of Genes Encoding the Five RCM Proteins

The knockout mutants were generated individually by an insertional mutation of each of the RCM-coding genes. The full segregation of the knockout mutants was confirmed by PCR detection of the wild-type (WT) DNA fragments of the corresponding genes, which should be completely undetectable in the mutants (supplemental Fig. S1) (13). The WT and the mutants were cultured in the AT, MT, PHT, and LAHG conditions and the growth curves were plotted (supplemental Fig. S2). Under the AT and MT conditions, no significant growth phenotype was observed for all the mutants. Under the PHT conditions, the growth of Δ*sigE* was severely inhibited, and the growth of Δ*hik8* was slightly affected as the culture became more yellowish. For all the other mutants, no significant difference of growth from that of the WT was observed. Under the LAHG conditions, the growth of Δ*sigE* and Δ*hik8* was nearly completely inhibited, and the growth of Δ*rre37* was also severely impaired but to a lesser extent compared to that of Δ*sigE* and Δ*hik8*. In contrast, Δ*hik31* and Δ*slr6041* grew as nearly well as the WT. The growth phenotypes of all the mutants are consistent with existing reports (11, 18, 27, 45). Note that individual inactivation of *hik31* or *slr6041* did not cause an obvious growth defect, but inactivation of both genes was reported to impair the growth with D-glucose (46), suggesting that the two genes may be functionally redundant, at least partly. For the cells cultured in the AT mode, pigment measurement demonstrates that the chlorophyll (Chl) and carotenoid content were not significantly changed in all the mutants compared with the WT (supplemental Fig. S2*E*). Measurement of 77K fluorescence shows only minor differences for the total amount and the stoichiometry of photosystem I and II among the WT and the mutants (supplemental Fig. S2*F*). Together, the results of the phenotypic analyses indicate that all the mutants grew nearly equally well under the AT conditions. Since differential protein expression in mutants could be induced directly by mutation or indirectly by secondary factors such as growth defect. All the cells used for proteomic analysis were subsequently cultured under the AT conditions to minimize the potential growth-defect induced differential protein expression.

### Quantitative Proteomic Identification of Differentially Expressed Proteins Across the RCM-Deficient Mutants

The proteomes of the WT and the five mutant cells were analyzed using a 6-plex TMT-labeling-based quantitative proteomics approach, which allows simultaneous quantification of the relative abundances of proteins in up to six independent samples (47). The overall strategy of the proteomic analysis is illustrated (Fig. 1*A*), reshuffling the order of TMT labeling between replicates was employed to avoid quantitative bias from each TMT reagent (Fig. 1*B*) (13). In total, 2543 proteins were identified with 2189 proteins containing quantitative TMT information in at least two replicates (Fig. 1*C*), and the latter represents the selected subset of proteins for further statistical and bioinformatics analyses (supplemental Table S2).

**Fig. 2:**
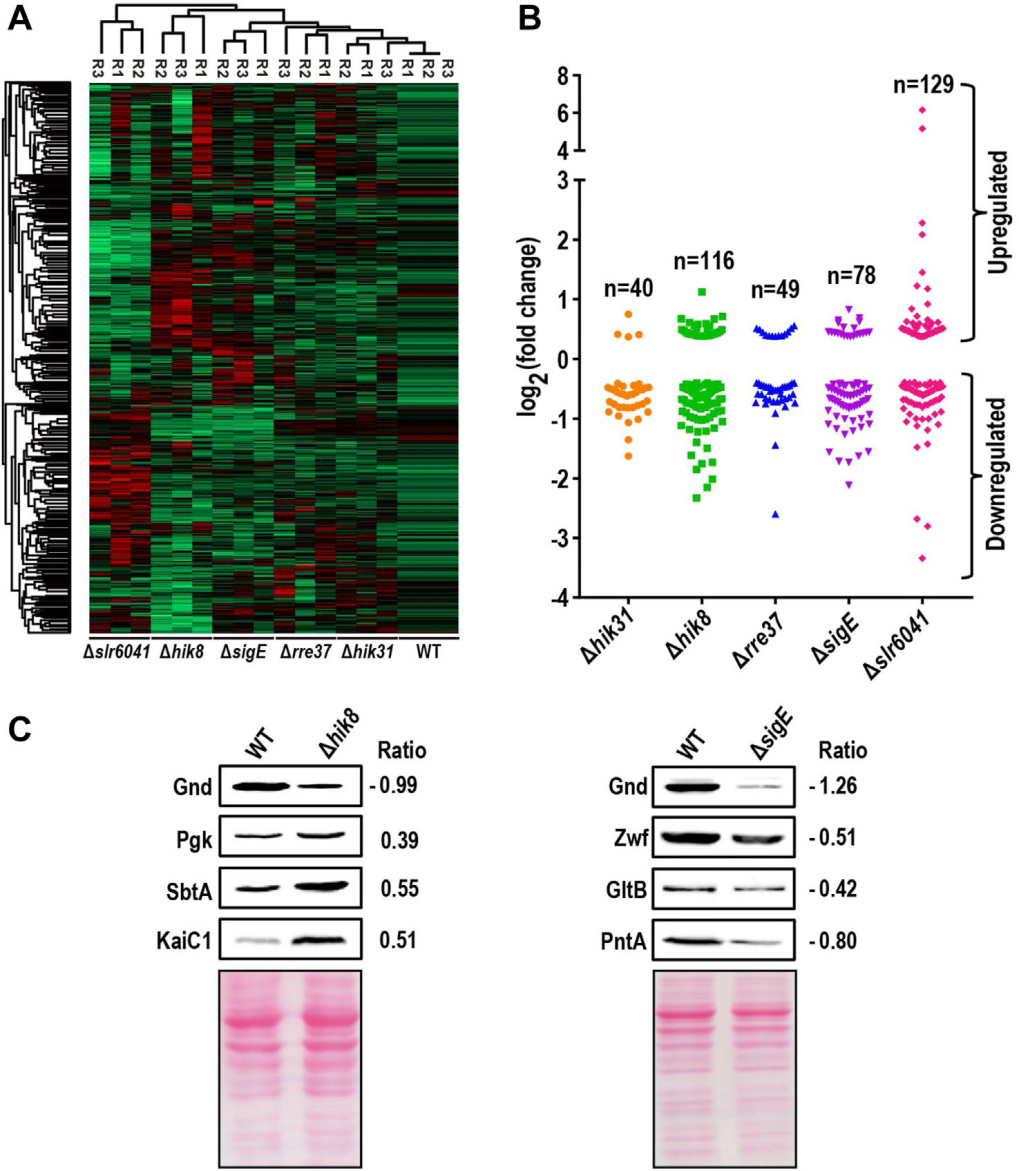
**Determination of differentially expressed proteins (DEPs) among the RCM mutants.***A*, Clustering analysis of the z-scored TMT reporting ion intensities to evaluate the reproducibility of the quantitative analysis. *B*, Distribution of fold changes of DEPs in the RCM mutants. Student’s *t* test *p* value (*p* < 0.05) and fold change threshold (FC>1.3) were used as the thresholds to filter for the DEPs. *C*, Western blotting confirmation of indicated DEPs, the log_2_ transformed fold-changes measured by TMT were shown to the right side of each panel. Ponceau staining was used as the loading control.

The overall reproducibility of the TMT quantitation is high, as indicated by the high correlation efficiency between any two of the biological replicates (R^2^ > 0.91) (supplemental Fig. S3). The fold-changes of each protein in all the mutants relative to the WT were calculated and Z-scored, and used for a clustering analysis. All the replicates from each of the samples were correctly clustered (Fig. 2*A*), further demonstrating high reproducibility of the quantification. Student’s *t* test with a threshold *p* < 0.05 and a fold-change threshold were used to sequentially filter the DEPs (32, 37, 40, 48). To determine the fold-change threshold, the TMT ratios (mutant/WT) for each mutant were firstly calculated for all proteins in all three replicates. The mean of the TMT ratios for each replicate was then calculated, which is expected to be close to 1 because samples in TMT channels were mixed with an equal ratio. The TMT ratios in each replicate were then normalized by the corresponding mean and the distribution of the resulting normalized ratios was calculated and plotted for all replicates of all mutants (supplemental Fig. S4). The result shows that at least 95.5% of the normalized ratios are smaller than 1.3, suggesting that a fold change of 1.3 is a reasonable threshold for quantitation with an estimated FDR smaller than 5%. In addition, due to the well-known precursor ion interference that usually causes a compressed ratio in isobaric labeling-based quantitative proteomics (49, 50), a protein with a measured 1.3-fold change in abundance may have a greater extent of differential expression. By applying both Student's *t* test *p* < 0.05 and a fold change of 1.3, 40, 116, 49, 78, and 129 proteins were identified as differentially expressed proteins (DEPs)in Δ*hik31*, Δ*hik8*, Δ*rre37*, Δ*sigE*, and Δ*slr6041*, respectively (Fig. 2*B*). A few DEPs were further validated through immunoblotting (Fig. 2*C*), and all these proteins were previously implicated in carbon metabolism except KaiC1 (7, 15, 27, 51, 52, 53, 54). The circadian clock protein KaiC1 and its ortholog in *Synechococcus* interact directly with Hik8 and its ortholog SasA, respectively (20, 26, 55), and the interaction is important for the regulation of circadian rhythmicity (20). Meanwhile, immunoblotting for other proteins without significant TMT-measured changes consistently show only slight variations in abundance among all the RCM mutants (supplemental Fig. S5). For Δ*hik31* and Δ*sigE*, DNA microarray-based transcriptomic data from similar photoautotrophic conditions is available in reported studies and was compared with our proteomics data, and the majority of overlapping genes show a similar trend of differential expression at both mRNA and protein levels (supplemental Fig. S6). Only a few genes show opposite directions of differential expression at the two levels, this is not surprising because it is well accepted that mRNA and protein levels are not always positively correlated (56). Similar comparisons were also performed for Δ*hik8* and Δ*rre37*. The two mutants do not have either a full list of transcriptomics data (Δ*hik8*) or the transcriptomics data obtained under the photoautotrophic growth conditions (Δ*rre37*). Therefore, we manually collected the transcription data of individual genes from the literature and performed the comparisons (11, 17, 27, 57). The results are similar to those of Δ*hik31* and Δ*sigE* (supplemental Fig. S6).

**Fig. 3:**
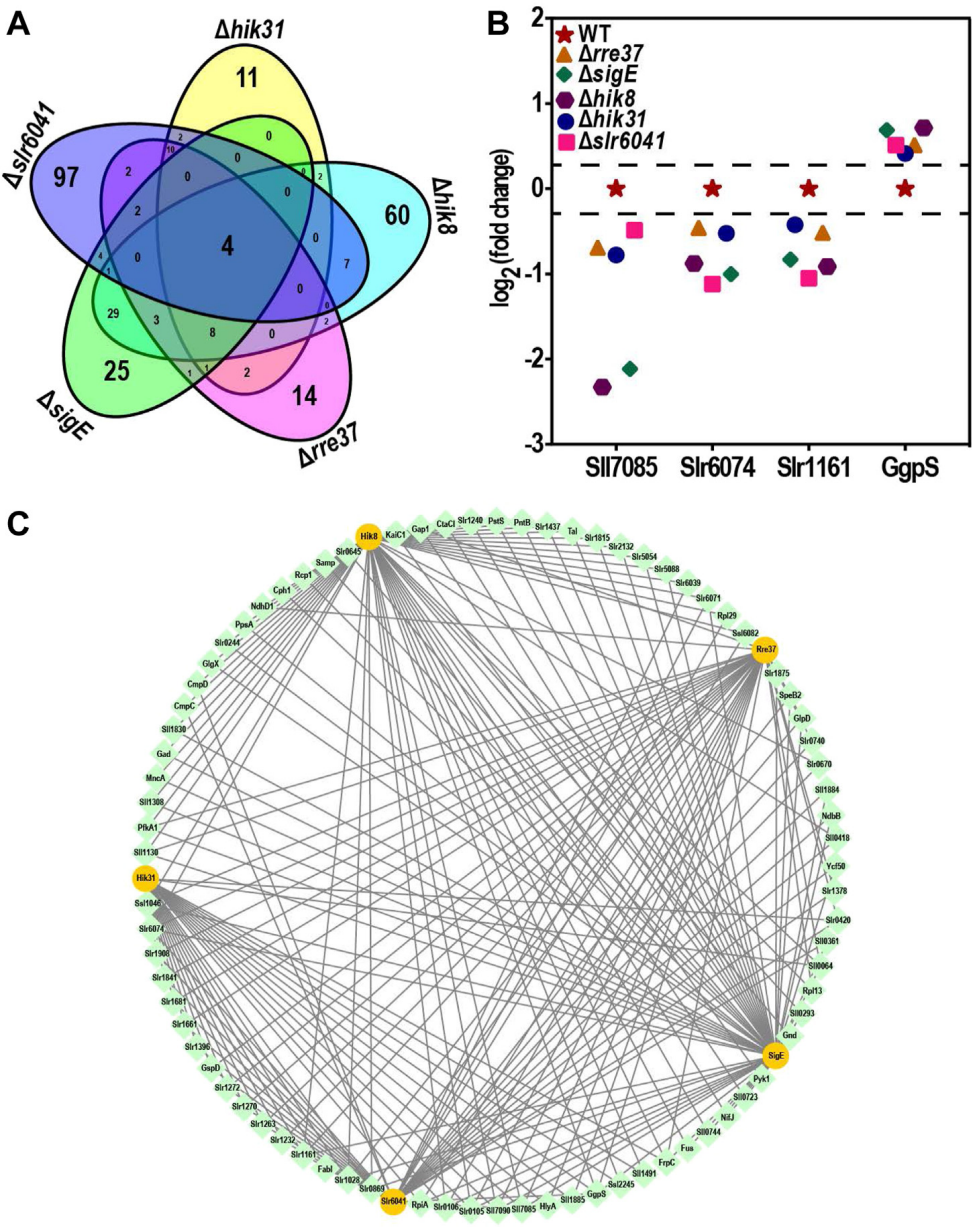
**The differentially expressed proteins (DEPs) commonly and specifically regulated by RCM proteins.***A*, Venn diagram showing the DEPs commonly and specifically regulated by the RCM proteins (FC>1.3, *p* < 0.05). *B*, DEPs commonly regulated by all the five RCM proteins. *C*, the regulatory network for 80 DEPs showing differential expression in at least two RCM mutants. The RCM proteins were included as the hub and highlighted in *yellow*.

The number of DEPs overlapping among the RCM mutants or specific to a particular mutant is shown by the Venn diagram (Fig. 3*A*). In total, only 4 DEPs were overlapping across all five mutants. These include Sll7085, Slr6074 and Slr1161 that were downregulated and glucosylglycerolphosphate synthase (GgpS) that was upregulated in all mutants (Fig. 3*B*). For the mutant-specific DEPs, the numbers are 11, 60, 14, 25, and 97 respectively for Δ*hik31*, Δ*hik8*, Δ*rre37*, Δ*sigE* and Δ*slr6041*, representing the list of proteins uniquely regulated by the corresponding RCM protein (supplemental Table S3). For better visualization of proteins regulated by at least two of the RCM proteins, a regulatory network for 80 proteins with a significant change in at least two mutant strains was constructed using the five RCM proteins as the hubs (Fig. 3*C* and supplemental Table S4). The network clearly demonstrates the cross-talk of regulation that might be important for *Synechocystis* to grow and cope with metabolic and environmental changes.

### Commonly and Differentially Regulated Functions in the Mutants

**Fig. 4:**
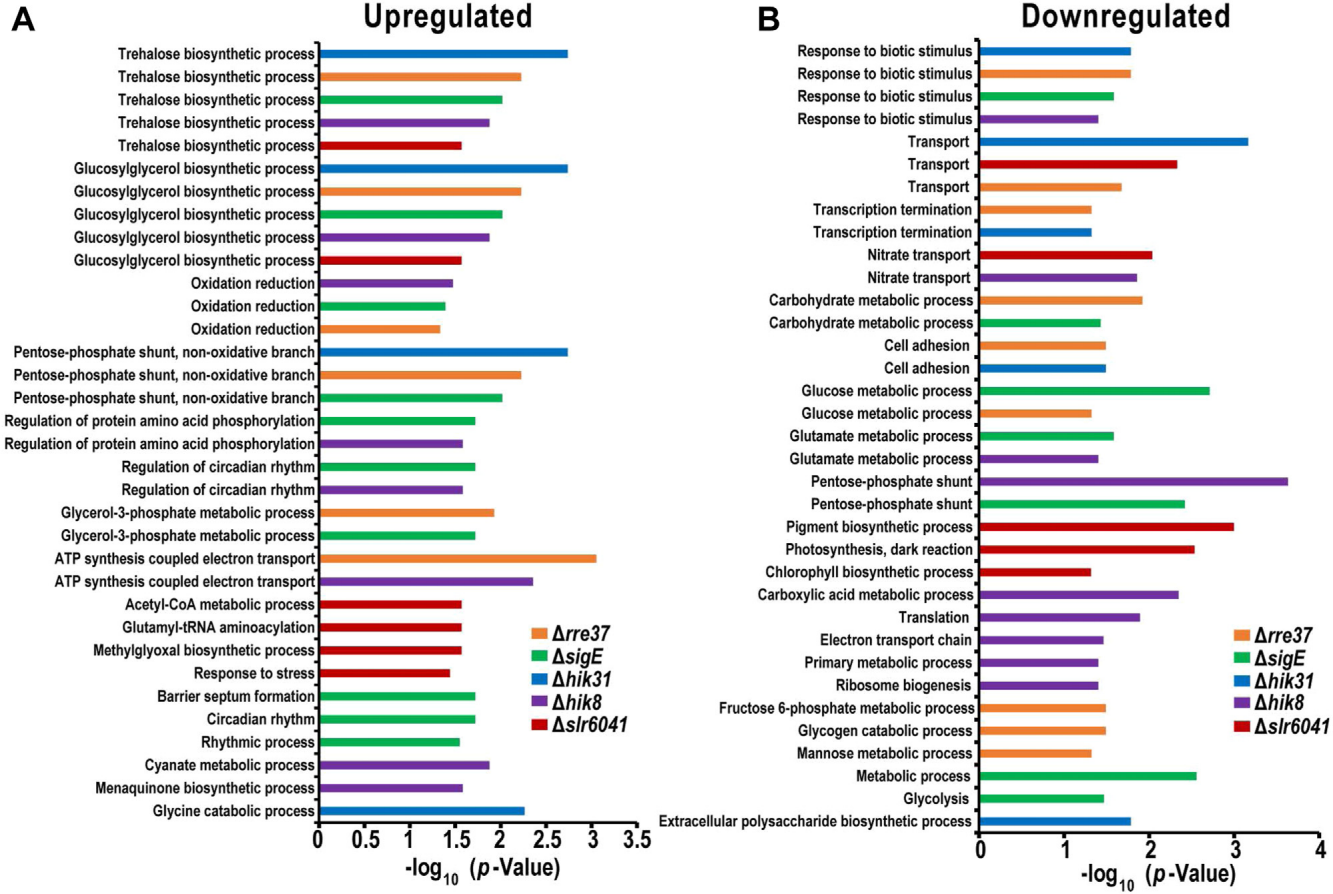
**Enriched functions in the differentially expressed proteins (DEPs) across the five RCM mutants.** Fisher’s-exact test for the functional enrichment in the upregulated (*A*) and downregulated (*B*) proteins across the RCM mutants. The gene ontology term biological process (GOBP) was used for the enrichment assay, *p* value < 0.05 and enrichment factor >1.5 were used as the thresholds.

To gain insight into the functions that are commonly or differentially regulated by the five RCM proteins, the enrichment of the Gene Ontology (GO) terms was assayed by Fisher’s-exact test for the DEPs in each RCM mutant. Among the upregulated proteins, two GO terms, the trehalose biosynthesis process, and the glucosylglycerol biosynthetic process, are enriched in all mutants (Fig. 4*A*) suggesting that all the five RCM proteins are involved in the negative regulation of the two processes. A few other GO terms are enriched in three, two, or only one mutant, indicative of the common or unique function regulated by the corresponding RCM proteins. Similarly, among the downregulated proteins, the GO term response to the biotic stimulus is enriched in four out of the five mutants, with the exception of the mutant Δ*slr6041*. The other GO terms are enriched in three, two or only one mutant (Fig. 4*B*).

It is expected that the functions related to carbon metabolism are impacted in the RCM mutants. Indeed, a number of GO terms associated with carbon metabolism were enriched in the DEPs. For the upregulated proteins, the nonoxidative branch of the pentose phosphate shunt is enriched in Δ*hik31*, Δ*rre37*, and Δ*sigE*. For the downregulated proteins, carbohydrate metabolic process, glucose metabolic process, pentose phosphate shunt, fructose-phosphate metabolic process, glycogen catabolic process, mannose metabolic process, and glycolysis are enriched in up to two mutants. The result further illustrates the specificity and cross-talk of the regulations on carbon metabolism by the RCM proteins. Remarkably, the deletion of *hik31* or its plasmid-borne copy *slr6041* differentially impacts a number of functions (Fig. 4), though the two proteins share more than 99% sequence similarity. The result is consistent with a previous report (18), and suggests that the two proteins are not completely redundant, though they can partially complement each other.

**Fig. 5:**
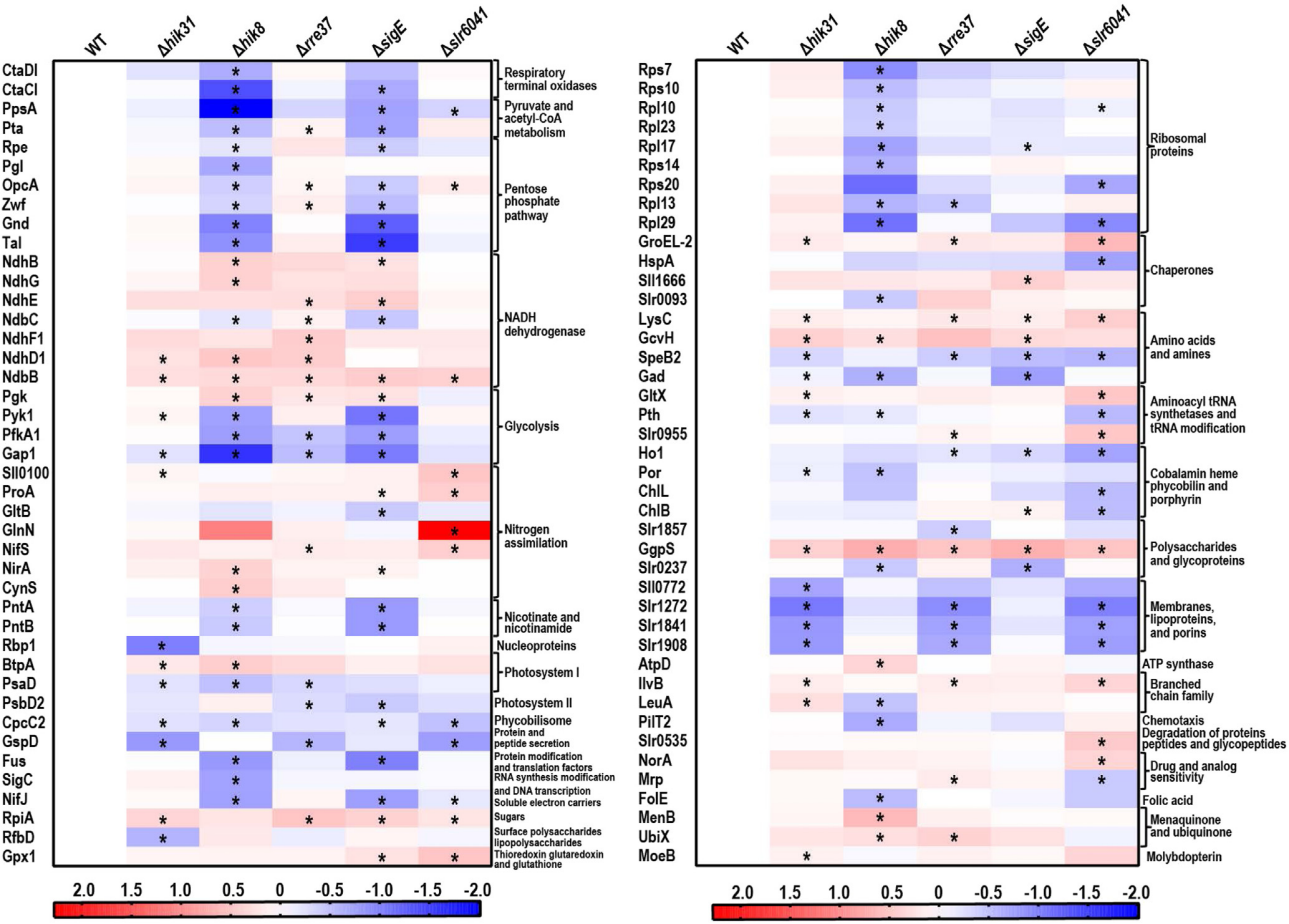
**Heatmap visualization of the differentially expressed proteins (DEPs) assigned with known functional categories.** Only DEPs in the known functional categories according to CyanoBase were included, and the DEPs in the categories hypothetical and unknown were not included. The color scale shows the abundance of each protein in each RCM mutant relative to that in the WT: *blue* indicates low level, *red* indicates high level, and white indicates no change. ∗*p* value < 0.05.

For better visualization and functional categorization of the DEPs, all DEPs with known functional categories of the CyanoBase were grouped and displayed in a heat map that shows the fold-changes of all DEPs in all mutants (Fig. 5 and supplemental Table S5). Again, common and differential regulation of the functions by the five RCM proteins are evident. The overall expression patterns of the DEPs are more similar between Δ*sigE* and Δ*hik8* and between Δ*hik31* and Δ*slr6041*. In Δ*rre37*, the expression patterns of some DEPs such as ribosomal proteins are more similar to that in Δ*sigE* and Δ*hik8*, whereas some others are more similar to that in Δ*hik31* and Δ*slr6041* such as lipoproteins and porins. In contrast, significant downregulation of lipoproteins and porins (Slr1908, Slr1841, Slr1272, and Sll0772) occurs in Δ*rre37*, Δ*hik31*, and Δ*slr6041*, but not in the other two mutants, although they display the trend of downregulation more or less in all mutants. It is evident that the numbers of DEPs in Δ*hik8*, Δ*sigE*, and Δ*slr6041* are greater than those in Δ*rre37* and Δ*hik31*. Notably, several ribosomal proteins were downregulated significantly in Δ*hik8*, including Rpl29, Rps14, Rpl13, Rpl17, Rpl23, Rpl10, Rps10, and Rps7. The result is in line with a previous microarray result (27).

For DEPs involved in carbon metabolism, 6 DEPs are involved in the pentose phosphate pathway, and their overall downregulation was apparent in Δ*sigE* and Δ*hik8*, but not in the other three mutants. Particularly, transaldolase (Tal) and 6-phosphogluconate dehydrogenase (Gnd) were downregulated in both Δ*sigE* and Δ*hik8*; Zwf, glucose 6-phosphate dehydrogenase assembly protein (OpcA), and pentose-5-phosphate-3-epimerase were significantly downregulated in Δ*sigE*, and they were also downregulated more or less in Δ*hik8* but not statistically significant. The 6-phosphogluconolactonase was downregulated only in Δ*hik8*. Four DEPs are involved in glycolysis, and their overall downregulation in Δ*sigE*, Δ*hik8* and Δ*rre37* is apparent except for phosphoglycerate kinase, which was upregulated in Δ*hik8*. Particularly, Gap1 and phosphofructokinase (PfkA1) were downregulated significantly in all three mutants and show the trend of weak downregulation in the other two mutants, pyruvate kinase (Pyk1) was downregulated significantly in Δ*sigE* and Δ*hik8* but not in Δ*rre37*. Overall, the differential expression pattern of the proteins involved in sugar catabolism is consistent with previous reports (15, 27). It is noticeable that no significant downregulation of the aforementioned DEPs occurs in Δ*hik31* or Δ*slr6041*, possibly due to the functional redundancy of Hik31 and Slr6041 (18).

Of all DEPs involved in sugar catabolism, Gap1 is important for glycolysis and Zwf catalyzes the first and the rate-limiting step of the OPPP (7, 54). Depletion of Gap1 and Zwf was reported to impair the viability of *Synechocystis* under the LAHG condition (58, 59), and the extents of downregulation of the two proteins in Δ*hik8*, Δ*sigE*, and Δ*rre37* correlate well with the extents of the reduced viability of the mutants in LAHG condition (supplemental Fig. S2*D*). Together, these results suggest that the decreased viability of Δ*hik8*, Δ*sigE*, and Δ*rre37* could mainly be attributed to the downregulation of Zwf and Gap1.

### Decreased Glycogen Content and Upregulated P_II_ Phosphorylation in AT-Grown Δ*hik8*

**Fig. 6:**
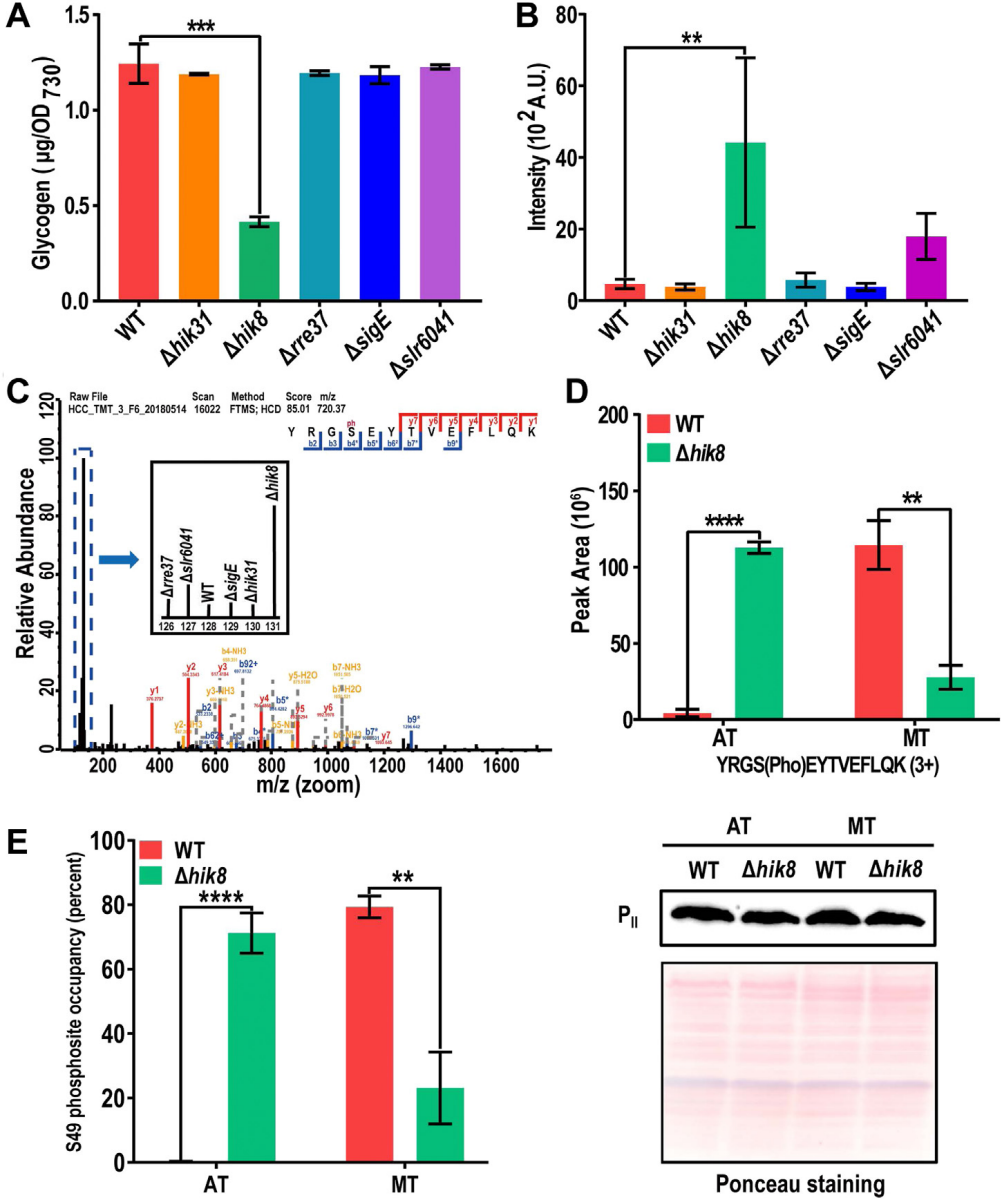
**Concomitant upregulation of P**_**II**_**phosphorylation and decrease of glycogen content in Δ*hik8*.***A*, Determination of glycogen contents among the WT and RCM mutants under AT condition. The cells were cultured under medium light intensity. ∗∗∗: *p* value < 0.001. *B*, quantitative identification of P_II_ S49 phosphorylation across the RCM mutants. Bars show the median abundances of a phosphopeptide from P_II_ bearing the S49 phosphosite measured from all replicates of the corresponding RCM mutants. Error bars represent standard deviations. ∗∗*p* value < 0.01. *C*, a representative mass spectrum of the phosphopeptide indicated in (*B*). The region containing TMT report ions (boxed by dashed lines) is horizontally zoomed in and displayed in the inset to show the relative abundance of the phosphopeptide in each sample. *D*, Bar graph shows the peak area of the phosphopeptides in (*C*) in the WT and Δ*hik8* cultured under AT and mixotrophic (MT) conditions. The measurement was performed using parallel reaction monitoring (PRM)-based target proteomics approach. ∗∗∗∗*p* value < 0.0001, ∗∗*p* value < 0.01. *E*, Calculation of P_II_ S49 phosphosite occupancy in the indicated *Synechocystis* strains and growth conditions (*left panel*). The calculation was performed using a previously described method (106). The phosphopeptide YRG**Sp**EYTVEFLQK and its non-phosphorylated counterparts (G**S**EYTVEFLQK and YRG**S**EYTVEFLQK) were used for quantitative PRM measurement (supplemental Tables S6–S8), the total P_II_ level was detected by Western blotting and the quantitation was performed using the software ImageJ (right panel). ∗∗*p* value < 0.01, ∗∗∗∗*p* value < 0.0001.

Glycogen is a major carbon and energy storage compound in cyanobacteria (60). Glycogen catabolism and cellular respiration are critical for cell viability in darkness (26, 61, 62, 63). Depletion of the RCM proteins could affect glycogen accumulation. Measurement of the glycogen content reveals that it was significantly decreased in Δ*hik8* as previously reported (27), but not in the other mutants (Fig. 6*A*). Remarkably, the proteins involved in glycogen synthesis were not significantly downregulated in Δ*hik8* (supplemental Table S5), suggesting there might exist a novel mechanism underlying Hik8-dependent glycogen accumulation. The accumulation of glycogen was reported to be regulated by the intracellular C/N balance, which is in turn sensed and regulated by the phosphorylation status of the protein P_II_ in cyanobacteria (64, 65, 66). P_II_ acts as a multitasking signal-integrating regulator whose phosphorylation state mirrors the cellular ATP and 2-oxoglutarate (2-OG) levels (67). The canonical P_II_ S49 phosphorylation is one of the most dominant phosphorylation events in *Synechocystis* and can be easily detected by liquid chromatography-tandem mass spectrometry (LC-MS) (68, 69). To reveal the P_II_ S49 phosphorylation status in the RCM mutants, we re-searched the raw mass spectrometry files against the *Synechocystis* proteome database including phosphorylation as the variable modification, and quantitatively identified with high confidence a tryptic peptide bearing the P_II_ S49 phosphosite in all replicates (Fig. 6, *B* and *C*). Importantly, the P_II_ S49 phosphorylation level, as quantified by TMT, was upregulated more than 6-fold in Δ*hik8* in comparison with that in the WT, and also upregulated in Δ*slr6041* but to a much less extent (Fig. 6*B*). In all other mutants, P_II_ S49 phosphorylation level was not significantly different from that in the WT (Fig. 6*B*). To confirm the upregulation of P_II_ S49 phosphorylation and to ask whether the high level of phosphorylation is constitutive or regulable in Δ*hik8*, we specifically quantified the abundances of the phosphopeptide in Figure 6*C* in AT/MT-grown WT and Δ*hik8* cells using a parallel reaction monitoring (PRM)-based target proteomics approach and calculated the S49 phosphorylation occupancy (40). The PRM result confirms the significant upregulation of P_II_ S49 phosphorylation in AT-grown Δ*hik8*, which is a nearly 26-fold increase in abundance compared with that in the WT (Fig. 6*D* and supplemental Fig. S7). Notably, the fold increase quantified by PRM is much greater than quantified by TMT due to the ratio compression effect of the isobaric labeling-based approach (70, 71). Nevertheless, both methods corroborate the strong increase of P_II_ S49 phosphorylation in AT-grown cells. In the MT-grown Δ*hik8*, the S49 phosphorylation level significantly decreased compared with that in AT-grown Δ*hik8* (Fig. 6*D* and supplemental Fig. S8), whereas in the MT-grown WT cells, the P_II_ S49 phosphorylation was significantly upregulated to a level nearly commensurate with that in the AT-grown Δ*hik8*, and the upregulation is consistent with our previous measurement using a TMT-labeling-based proteomics approach (32). P_II_ S49 phosphorylation occupancy was also calculated for the cells grown under both conditions, the result revealed that nearly 70% of P_II_ molecules are phosphorylated in Δ*hik8*, whereas less than 4% are phosphorylated in the WT under the AT conditions (Fig. 6*E*, supplemental Table S8). Together, the PRM results suggest that the P_II_ S49 phosphorylation in Δ*hik8* is regulable, at least through the change of nutrition mode.

### P_II_ S49A Mutation Restores Glycogen Accumulation and Dark Viability of Δ*hik8*

**Fig. 7:**
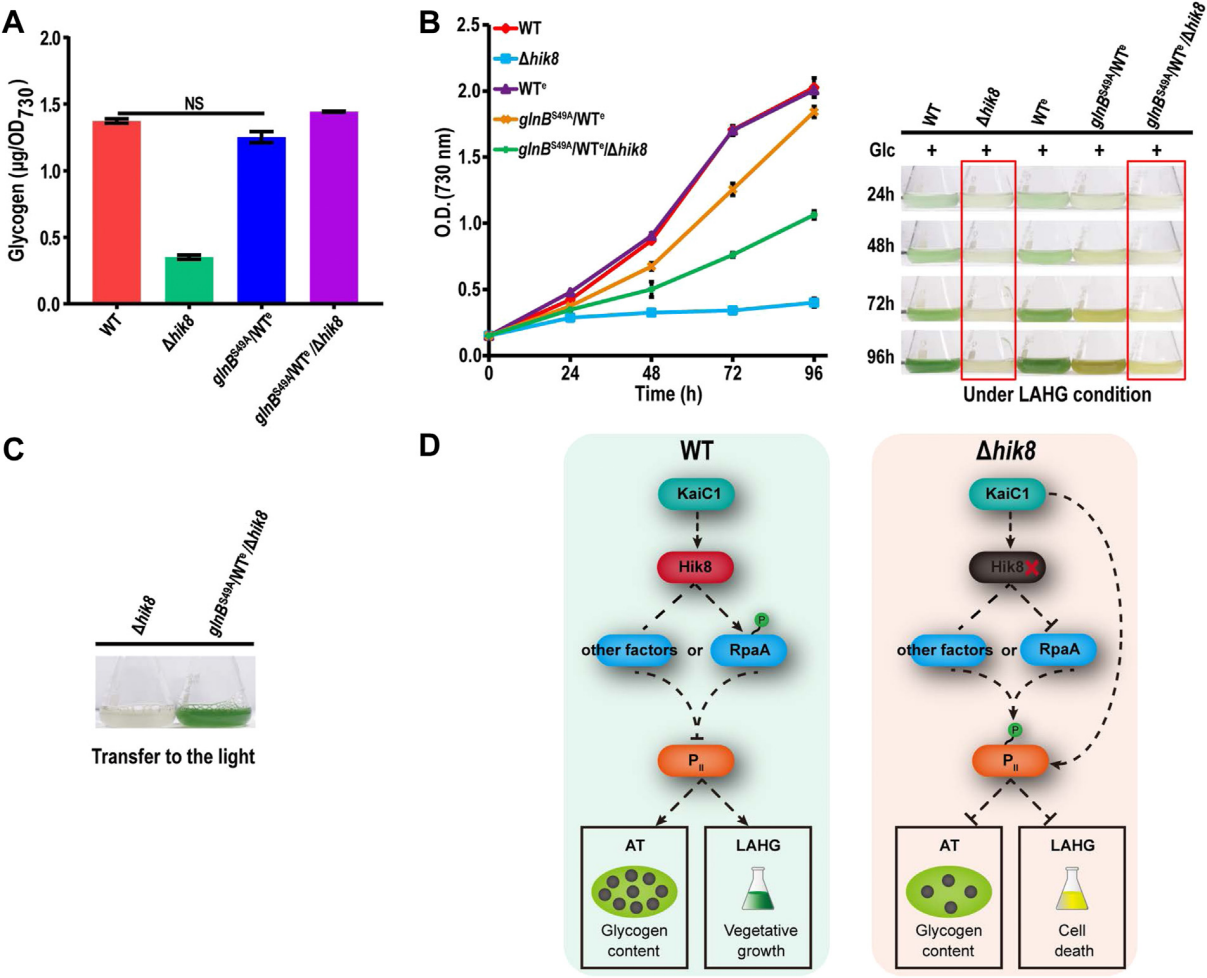
**P**_**II**_**S49A substitution restored the glycogen content and rescued the dark viability of Δ*hik8*.***A*, Bars show the glycogen content measured for the indicated strains. All strains were cultured under AT conditions. Error bars: standard deviations. NS: not significant. *B*, *Left*: growth curves of the indicated strains under the light-activated heterotrophic growth (LAHG) condition. *Right*: The images of the cultures photographed at the indicated time points to show the color changes of the cells during the time course. *C*, After 96 h LAHG growth, the indicated strains (also shown in the red box in (*B*)) were transferred to continuous light and illuminated for another 48 h. *D*, A working model for Hik8 regulation of glycogen accumulation and dark viability through negative regulation of P_II_ phosphorylation.

To investigate whether there is a causal relationship between the upregulation of P_II_ S49 phosphorylation and the decrease of the glycogen content in AT-grown Δ*hik8*, we introduced a site-specific mutation on *glnB*, the coding gene of P_II_, to introduce a non-phosphorylatable S49A mutation in P_II_ in the background of both Δ*hik8* and the WT equivalent strains (WT^e^) (supplemental Fig. S14 and Experimental procedures). Glycogen determination shows that there is no significant difference in glycogen content between the two strains, and the glycogen content in *glnB*^S49A^/WT^e^/Δ*hik8* was dramatically increased to a level comparable to that in the WT, and the level is at least two-fold higher than that in Δ*hik8* (Fig. 7*A*). These data indicate P_II_ S49A mutation can successfully recover the glycogen content in Δ*hik8* but with little effect on the glycogen accumulation in the WT. Together, these results strongly suggest that hyperphosphorylation of P_II_ in Δ*hik8* accounts for the reduced glycogen content.

To further ask whether P_II_ S49A mutation can rescue Δ*hik8* in LAHG, a lethal condition for Δ*hik8* as observed by us and others (supplemental Fig. S2*D*) (27), the growth experiment was performed for the WT and the mutants in the LAHG condition. The growth curve shows that *glnB*^S49A^/WT^e^/Δ*hik8* can grow in LAHG (Fig. 7*B*). The concentration of the cells continued to increase during the time course of culturing, though the growth rate was slower than that of the WT and the color of the cells were yellowish. In contrast, the growth of Δ*hik8* was completely stopped after 24 h incubation in LAHG and the color of the culture became pale white. To further confirm the viability of the mutants, Δ*hik8* and *glnB*^S49A^/WT^e^/Δ*hik8* were re-illuminated with light after 96 h incubation in the dark. The culture of *glnB*^S49A^/WT^e^/Δ*hik8* became fresh green after 48 h incubation in light (Fig. 7*C*), indicating that the mutant cells survived the 96 h dark incubation. In contrast, the color of the Δ*hik8* culture did not change, indicative of cell death. Noticeably, the growth of *glnB*^S49A^/WT^e^ was also significantly repressed as indicated by the slower growth and yellowish color of the culture, though the repression was not as severe as observed for the other two mutants (Fig. 7*B*). The observation suggests that P_II_ S49A mutation cause pleiotropic effects that are detrimental for the WT but beneficial for Δ*hik8*. This also explains that the P_II_ S49A mutation did not restore the growth of Δ*hik8* to the comparable level of that of the WT. Based on these results, we proposed a model to depict the Hik8-regulated P_II_ phosphorylation, glycogen accumulation, and dark viability (Fig. 7*D*). Shown in the model, regulation of the circadian clock (KaiC1-Hik8-RpaA) maintains P_II_ phosphorylation at a low level in the photoautotrophically growing WT *Synechocystis*. Depletion of Hik8 leads to hyperphosphorylation of P_II,_ resulting in the decrease of the glycogen content and the dark viability of *Synechocystis*. In addition to Hik8 and RpaA, KaiC1 is the only other circadian clock protein shown in the model, because KaiC1 directly interacts with Hik8 and is the only upregulated circadian oscillator protein in Δ*hik8* (supplemental Fig. S17) (20, 26, 55). KaiC1 is not a typical serine/threonine kinase but was reported to have autokinase and autophosphatase activities (72). Therefore, it is also possible that upregulated KaiC1 directly hyperphosphorylates P_II_ in Δ*hik8* (Fig. 7*D*). In addition to RpaA, other unknown factors could also be involved to regulate P_II_ phosphorylation in downstream of Hik8 (Fig. 7*D*).

## Discussion

Cyanobacteria coordinately regulate diverse types of cellular metabolism in response to environmental and internal changes. The regulatory schemes that control carbon metabolism are very complex due to the large number of proteins, metabolites, and pathways involved. For more robust and precise regulation in response to the changing environmental and internal conditions, it is desirable that a subset of proteins in a pathway are commonly regulated by multiple proteins or a subset of proteins in different pathways are exclusively regulated by a single protein. For carbon metabolism, a few proteins have been implicated in such an intricate and elegant regulation, including SigE, Hik8, Rre37, Hik31, and Slr6041(11–17). However, the identities of the common and separate targets regulated by these RCM proteins have not been comprehensively investigated. In this study, a 6-plex TMT-labeling-based quantitative proteomics strategy was explored to simultaneously compare the proteomes of the mutants each depleted of one of the RCM proteins. Common and separate targets of the RCM proteins were identified (Fig. 3*C* and supplemental Table S3), functions that are commonly regulated by multiple RCM proteins or specifically regulated by a single RCM protein were uncovered (Fig. 4). The results provide a holistic perspective of proteome changes to understand the regulatory mechanism of carbon metabolism in *Synechocystis*. Remarkably, four proteins were found to be commonly regulated by all the five RCM proteins (Fig. 3*B*). Of these, transcription of *ggpS* was reported be upregulated in response to osmotic stress and salt stress (73, 74, 75), and the transcription of *slr1161* was reported to be repressed by the stresses of high light, 3-(3,4-dichlorophenyl)-1,1-dimethyl urea (DCMU), and 2,5-dibromo-3-methyl-6-isopropyl-p-benzoquinone (DBMIB) (76, 77, 78). The unanimous upregulation and downregulation of GgpS and Slr1161, respectively, in all five RCM mutants suggest that the mutants need to cope with the stresses generated more or less by the depletion of the RCM proteins. Sll7085 is a member of the CRISPR-associated proteins superfamily (48, 79), which is downregulated in all mutants but to greater extents in Δ*sigE* and Δ*hik8*. The coding gene of Sll7085 is part of an operon on the endogenous plasmid pSYSA (80), and the operon also encodes the other components of the same CRISPR complex. It remains elusive why Sll7085 but not the other components of the CRISPR complex were dramatically downregulated in the two mutants. Slr6074 is not previously associated with any stress responses, indicating it could be a *bona fide* carbon metabolism-related target of RCM proteins. Indeed, the knockout mutant of *slr6074* displayed severe defects in trophic growth, and the results for this part of the work will be reported separately.

The dark viability of Δ*sigE*, Δ*hik8*, and Δ*rre37* in the presence of glucose was all impaired as also shown by previous reports (supplemental Fig. S2*D*) (15, 62, 81). Impaired sugar catabolism has been proposed to account for this phenotype because of the inability to provide the energy necessary for dark survival through catabolizing glucose (8, 27). Moreover, the accumulation of glucose to a certain level could also be toxic for cyanobacteria (13, 46, 82, 83). In *Synechocystis*, and many other cyanobacteria strains as well, OPPP is the major route for catabolizing glucose in the dark (7). This, together with the downstream part of glycolysis that starts from the reaction catalyzed by Gap1, channels the vast majority of carbon flux for the production of energy, reducing equivalent, and building blocks for biomass (84). In *Synechocystis*, *Nostoc* sp. ATCC 29133, and *Synechococcus*, mutants depleted of Zwf displayed impaired dark viability in the presence of glucose (10, 58, 85). In Δ*hik8* and Δ*sigE*, four proteins in OPPP including Zwf, OpcA, Gnd, and Tal were unanimously downregulated, indicative of defective OPPP. Thus, the impaired dark viability of the two mutants could be mainly attributable to the downregulation of these proteins. In addition, three proteins in glycolysis including Pyk1, PfkA1, and Gap1 were downregulated in both mutants, and this may also contribute more or less to the impaired dark growth (84). In Δ*rre37*, however, only two of the aforementioned proteins in sugar catabolism were downregulated (PfkA1 and Gap1), and the extent of downregulation is not as great as that in Δ*sigE* and Δ*hik8*. This result may correlate with the relatively less serve repression of the growth of glucose in the dark as observed (supplemental Fig. S2*D*). Nevertheless, the mechanism of impaired dark viability in the presence of glucose is complex, alteration of other proteins in carbon metabolism may also be attributable to this phenotype.

Dramatically decreased glycogen content and massive upregulation of P_II_ S49 phosphorylation in Δ*hik8* but not in the other RCM mutants are the most prominent findings in the present study (Fig. 6). Decreased glycogen content in an AT-grown *hik8*-deficient mutant was previously reported (27), but it is a bit surprising that this occurs only in Δ*hik8* but not in other mutants such as Δ*sigE*, considering that expression patterns of many proteins in carbon metabolism are very similar between in Δ*hik8* and Δ*sigE*. The observation underscores the specificity of Hik8 in regulating carbon metabolism, particularly, glycogen accumulation. The massive increase of P_II_ phosphorylation in Δ*hik8* but not in the other mutants perfectly correlates, though negatively, with the decreased glycogen content (Fig. 6), and this prompted us to investigate their causal relationship. The successful restoration of glycogen content to a level comparable with that in the WT by P_II_ S49A mutation suggests that hyperphosphorylation of P_II_ is more probably attributable to the reduced glycogen content in Δ*hik8*. It is highly remarkable that the glycogen contents in different *Synechocystis* strains in the current study are at a basal (∼1 μg/OD_730_) or sub-basal level (Fig. 6*A*), which is up to two orders of magnitude lower than that induced by nitrogen starvation (86, 87). It has been well established that nitrogen starvation-induced P_II_ phosphorylation positively correlates with massive accumulation of glycogen in WT *Synechocystis* (88, 89), but this does not necessarily contradict our result that hyperphosphorylation of P_II_ negatively correlates with basal level glycogen content in Δ*hik8*. It is expectable that basal and stimulated levels of glycogen accumulation might be differentially regulated at both molecular and metabolic levels. Recently, Orthwein *et al.* reported under nitrogen starvation a small protein PirC (Sll0944) specifically binds to and inhibits 2,3-phosphoglycerate-independent phosphoglycerate mutase (PGAM), an enzyme important for the lower part of the glycolysis pathway. Inhibition of PGAM would re-direct the carbon flux from the photosynthetic Calvin cycle to glycogen synthesis, and result in massive accumulation of glycogen in nitrogen-starved cells. Under nitrogen repletion, the binding of PirC by P_II_ sequesters PirC from and activates PGAM, which directs carbon flux to the downstream TCA cycle and represses glycogen synthesis (88, 89). In our case, P_II_ S49A mutation restored glycogen from a sub-basal level to the basal level in Δ*hik8* but did not significantly change the basal level glycogen content in the WT (Fig. 7*A*). If P_II_ S49A mutation inhibits P_II_-PirC interaction and promoters PirC binding to and inhibiting PGAM, and consequently increases glycogen content in Δ*hik8*, then it is expectable that S49A mutation would also significantly increase the glycogen content in the WT, which is obviously not the case (Fig. 7*A*). Thus, the mechanism of PirC-regulated glycogen synthesis could be more appropriate to explain the induced massive accumulation of glycogen that requires a dramatic directional change in metabolic carbon flux. However, whether the mechanism is equally applicable to explain the regulation of glycogen accumulation at a basal or sub-basal level is still questionable. Together, although P_II_ S49 phosphorylation was regarded as a well-known signal for the intracellular C/N balance (90, 91, 92), its regulation on glycogen accumulation, particularly at the basal level under the control of Hik8, is just revealed. To our knowledge, this is the first line of evidence that links P_II_ phosphorylation to a histidine kinase of the two-component system in cyanobacteria.

Notwithstanding the effective restoration of the glycogen content and the prevention of cell death in Δ*hik8* by P_II_ S49A mutation (Fig. 7), the dark growth of *glnB*^S49A^/WT^e^/Δ*hik8* with glucose was not restored to a level commensurate with that of the *glnB*^S49A^/WT^e^, and the later was also partly repressed in growth compared with the WT (Fig. 7*B*). The observation suggests that P_II_ S49A substitution favors dark survival of Δ*hik8* in the presence of glucose. The increased glycogen resulting from S49A substitution in *glnB*^S49A^/WT^e^/Δ*hik8* and its catabolism might provide energy, through respiratory electron transport, necessary for Δ*hik8* to survive the prolonged darkness (27). Nevertheless, the P_II_ S49A substitution is also harmful to cell growth as displayed by the growth phenotype of *glnB*^S49A^/WT^e^ (Fig. 7*B*). This is not surprising because P_II_ S49A is unmodifiable and hence loses the flexibility to regulate or fine-tune C/N metabolism through reversible phosphorylation, which is harmful for the cyanobacterium to cope with the adverse environment, such as prolonged darkness. Nevertheless, the network of carbon metabolism is highly complex and interconnected with nitrogen metabolism (64, 65). Multiple pathways including a number of reactions (uni-direction or bi-direction) are confined within the same cellular compartment. Coordination of these pathways and reactions requires tight and precise spatiotemporal regulation that occurs at multiple levels on mRNAs, proteins, and metabolites, and different parts of a pathway may also be differentially regulated to precisely divert the carbon flux to different destinations in response to environmental changes. Therefore, a detailed mechanism of Hik8-regulated glycogen accumulation, P_II_ phosphorylation, and dark survival with glucose is far from elucidated.

Reversible P_II_ S49 phosphorylation is a well-established mechanism for sensing the state of C/N homeostasis in many cyanobacteria strains and has been extensively discussed (67). Nevertheless, the functions of phosphorylated and unphosphorylated forms of P_II_ are not well studied. P_II_ is necessary for ammonium-responsive inhibition of nitrate assimilation (93). It was reported that neither S49A nor S49D substitution affects nitrate assimilation and ammonium-responsive inhibition of nitrate assimilation in *Synechocystis* and *Synechococcus* (94, 95), and S49D mutation dramatically reduced P_II_-interaction with Amt1, NrtD, and UrtE, which are involved in transporting ammonium, nitrate, and urea, respectively (96). Other than this scarce information is available regarding the phenotype of cyanobacteria strains carrying P_II_ S49A substitution. Our results strongly suggest that P_II_ phosphorylation is involved in the regulation of the tropic growth of *Synechocystis*. Specifically, the capability of P_II_ being phosphorylated is important for dark growth (Fig. 7*B* and supplemental Fig. S15), but not as important for AT and MT growth (supplemental Fig. S16).

Hik8 is a well-known two-component sensory histidine kinase involved in the regulation of circadian rhythmicity. The Hik8 ortholog in *Synechococcus*, SasA, interacts directly with the circadian clock protein KaiC (20), and the latter has both autokinase and autophosphatase activities towards phosphorylation and dephosphorylation of S431 and T432 on KaiC, respectively. The dual phosphorylation of KaiC was reported to be the basis of circadian rhythm in cyanobacteria (72). Prominently, the *Synechocystis* ortholog of KaiC, namely KaiC1, is the only circadian clock protein upregulated in Δ*hik8* (Fig. 2*C* and supplemental Fig. S17). KaiC1 also interacts directly with Hik8 (20, 26, 55). KaiC1 could regulate the circadian rhythm of the phosphotransfer activity from Hik8/SasA to its cognate response regulator RpaA/Rre31 (20, 97, 98, 99), and subsequently regulate rhythmic oscillation of gene expression and metabolic activities (21). RpaA depletion induces a major metabolic shift of the mutant that largely phenocopies Δ*hik8* (100). It is conceivable that the inactivation of Hik8 changed the phosphorylation status of RpaA and its activity as a transcription factor and affected the expression of one or more genes that regulate the phosphorylation of P_II_. The observed upregulation of KaiC1 in Δ*hik8* could be a feedback response of Hik8 inactivation (supplemental Fig. S17). Indeed, P_II_ phosphorylation is also modulated in response to light and darkness (32), indicating that P_II_ phosphorylation could also be under circadian regulation. Unfortunately, to date the identity of the kinases responsible for P_II_ phosphorylation remains elusive, and it has been suggested that multiple protein kinases that complement with each other are involved (101).

Overexpression RpaA in *Synechocystis* was reported to slow down glycogen degradation in dark (102). It is conceivable that the inactivation of RpaA or Hik8 may accelerate glycogen degradation, resulting in reduced glycogen content. In *Synechococcus*, the glycogen content displayed a typical circadian oscillation peaking at the subjective dusks and reaching troughs at the subjective dawns (103, 104), such a circadian rhythm of glycogen content is more likely regulated by the output signals from the circadian clock mediated by Hik8-RpaA. The abolished circadian rhythm in Δ*hik8* is expected to disrupt the circadian rhythm of the glycogen accumulation and arrest the mutant in a state with constitutively low glycogen content as also suggested by Kawasaki *et al* (104), and this process is probably mediated through hyperphosphorylation of P_II_.

Together, our findings could help to make connections among Hik8, KaiC1, and P_II_, and to place P_II_-controlled C/N homeostasis under the regulation of the circadian clock with Hik8-RpaA as the major output pathway.

## Data Availability

The mass spectrometry proteomics data have been deposited to the ProteomeXchange Consortium *via* the PRIDE partner repository with the dataset identifier PXD035864 and PXD035898 (105).

## Supplemental data

This article contains supplemental data (106).

## Conflict of interest

The authors declare no competing interests.
