# Supplementary material for: Parallel Proteomic Comparison of Mutants With Altered Carbon Metabolism Reveals Hik8 Regulation of PII Phosphorylation and Glycogen Accumulation in a Cyanobacterium
Source: Mol Cell Proteomics. 2023 May 22;22(7):100582. doi: 10.1016/j.mcpro.2023.100582 (PMC10315926; doi:10.1016/j.mcpro.2023.100582)
Supplement: Supplemental Figures S1–S18 [file mmc1.pdf]

Supplementary Materials for

Parallel proteomic comparison of mutants with altered carbon metabolism reveals Hik8 regulation of P_{II} phosphorylation and glycogen accumulation in a cyanobacterium

Chengcheng Huang[1,2,ξ], Xiaoxiao Duan[1,2,ξ], Haitao Ge[1], Zhen Xiao[1,2], Limin Zheng[1,2], Gaojie Wang[1,2], Jinghui Dong[1,2], Yan Wang[1,2], Yuanya Zhang[1], Xiahe Huang[1], Hongyu An[1,2], Wu Xu[3], and Yingchun Wang[1,2]*

Running title: Regulation of the subproteome in carbon metabolism

[1]State Key Laboratory of Molecular Developmental Biology, Institute of Genetics and Developmental Biology, Chinese Academy of Sciences, No.1 West Beichen Rd., Beijing 100101, China.

[2]University of Chinese Academy of Sciences.

[3]Department of Chemistry, University of Louisiana at Lafayette, Lafayette, LA 70504, USA.

[ξ]These authors contributed equally to this work.

*Corresponding author

This PDF file includes:
Figures S1-S18

Supplemental data:

Fig. S1. Confirmation of the knockout mutants for genes encoding RCM proteins.

Fig. S2. Phenotypes of the knockout mutants for genes encoding the RCM proteins.

Fig. S3. Correlational analyses of the TMT reporter intensities among the triplicated samples.

Fig. S4. Determination of the fold change threshold for DEPs.

Fig. S5. Western-blotting validation of a few proteins without significant changes in abundance among the WT and the mutants.

Fig. S6. Comparison of gene expression at the mRNA and protein levels for the indicated *Synechocystis* mutants.

Fig. S7. Measurement of P_{II} S49 phosphorylation in AT-grown WT and Δ*hik8* through PRM.

Fig. S8. Measurement of P_{II} S49 phosphorylation in MT-grown WT and Δ*hik8* through PRM.

Fig. S9. Measurement of the peptide GSEYTVEFLQK in AT-grown WT and Δ*hik8* through PRM.

Fig. S10. Measurement of the peptide GSEYTVEFLQK in MT-grown WT and Δ*hik8* through PRM.

Fig. S11. Measurement of the peptide YRGSEYTVEFLQK in AT-grown WT and Δ*hik8* through PRM.

Fig. S12. Measurement of the peptide YRGSEYTVEFLQK in MT-grown WT and Δ*hik8* through PRM.

Fig. S13. PRM-quantified peak area of the non-phosphorylated peptides containing P_{II} S49 site in AT/MT-grown WT and Δ*hik8*.

Fig. S14. Confirmation of the mutants with P_{II} S49A substitution.

Fig. S15. Growth curves of the WT, Δ*hik8,* and *glnB*^{S49A}/WT^{e} under the PHT conditions.

Fig. S16. Growth curves of WT, Δ*hik8,* and *glnB*^{S49A}/WT^{e} under AT (A) and MT conditions (B).

Fig. S17. The volcano plot shows all proteins quantitatively analyzed by TMT in Δ*hik8*.

Fig. S18. Annotated spectra for all proteins identified with a single peptide.

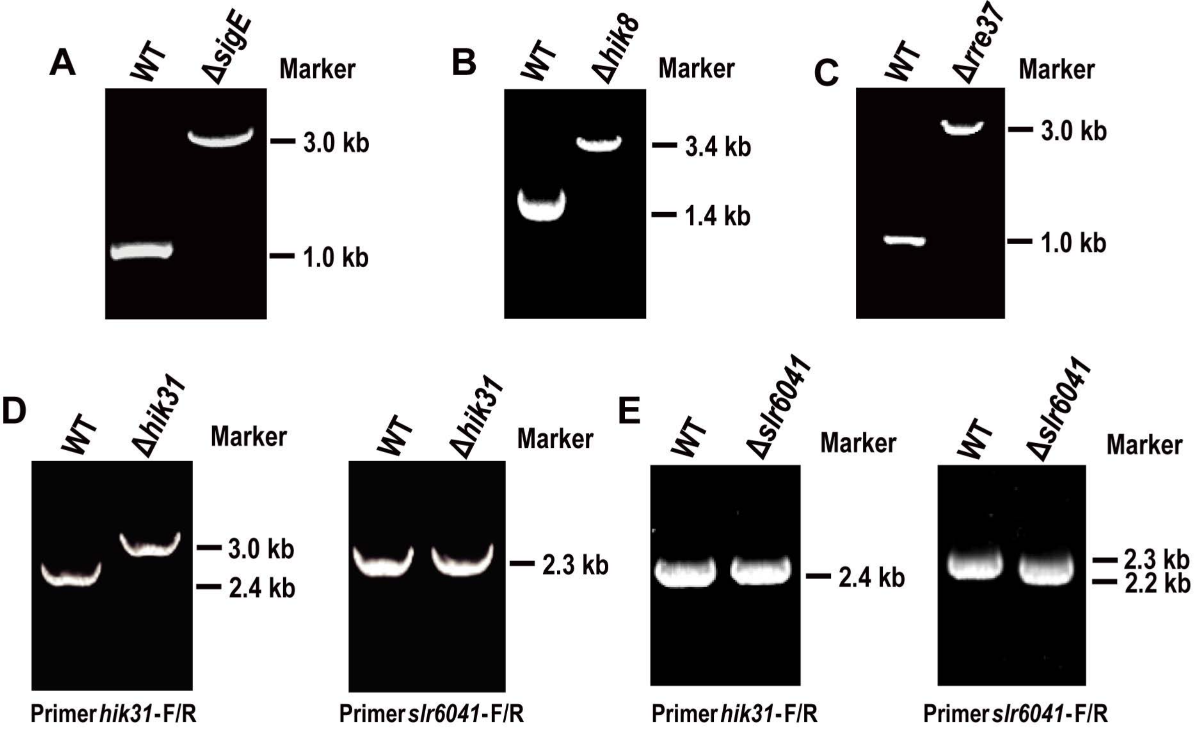

Supplemental Fig. S1. Confirmation of the knockout mutants for genes encoding RCM proteins. The complete segregation of the indicated mutants was confirmed by PCR. For each mutant, the same pair of primers were used to amplify a DNA fragment from the WT and the mutant covering the region where the antibiotic resistant gene is inserted. In addition, the chromosome (Δ*hik31*) and the plasmid (Δ*slr6041*) copy of hik31 were deleted individually from each position.

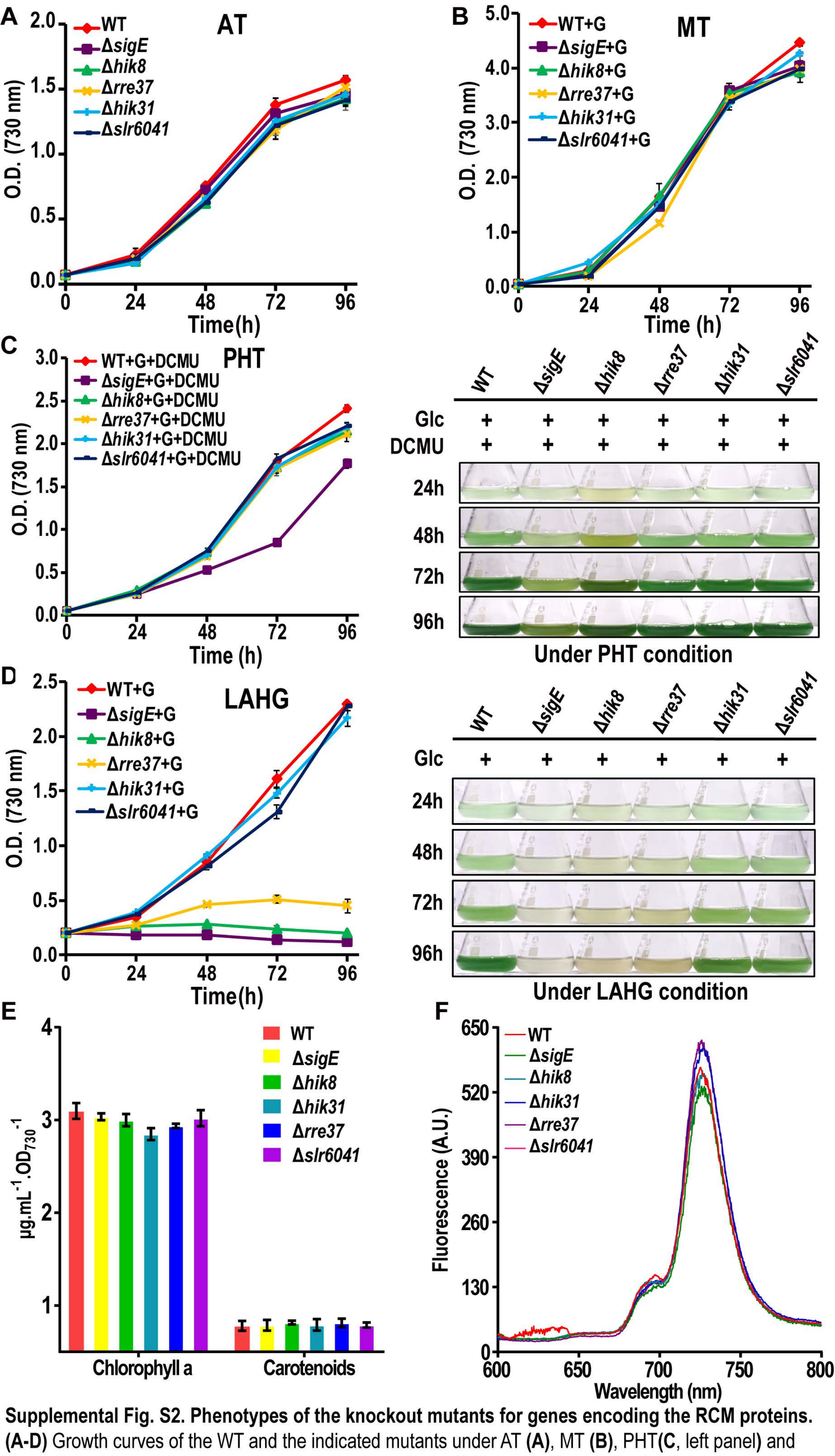

Supplemental Fig. S2. Phenotypes of the knockout mutants for genes encoding the RCM proteins. **(A-D)** Growth curves of the WT and the indicated mutants under AT **(A)**, MT **(B)**, PHT**(C**, left panel**)** and LAHG **(D**, left panel**)** conditions. The cells cultured in PHT **(C**, right panel**)** and LAHG **(D**, right panel**)** conditions were photographed at the indicated time points to show the color change. **(E)** The pigment contents of the WT and the indicated mutants were measured under the AT condition. Error bars: standard deviations. **(F)** Low-temperature (77K) fluorescence emission spectra of the WT and the indicated mutants grown under the AT condition.

A

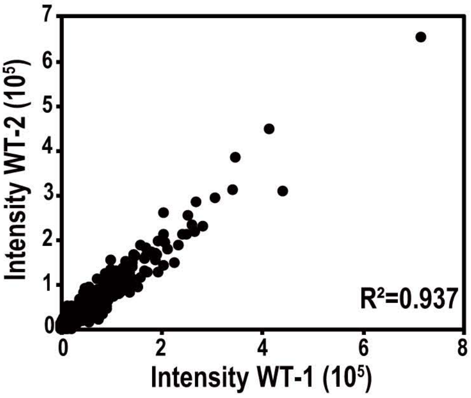

R^2 (WT)

	R1	R2	R3
R1	1		
R2	0.937	1	
R3	0.923	0.930	1

B

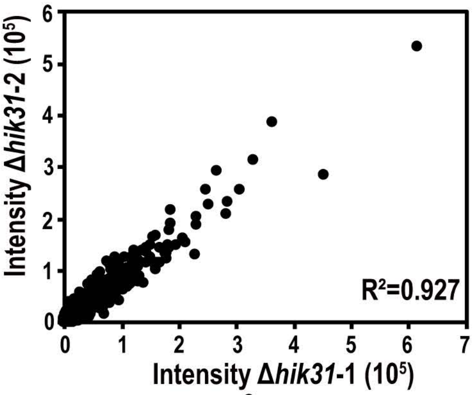

R^2 (Δ*hik31*)

	R1	R2	R3
R1	1		
R2	0.927	1	
R3	0.935	0.924	1

C

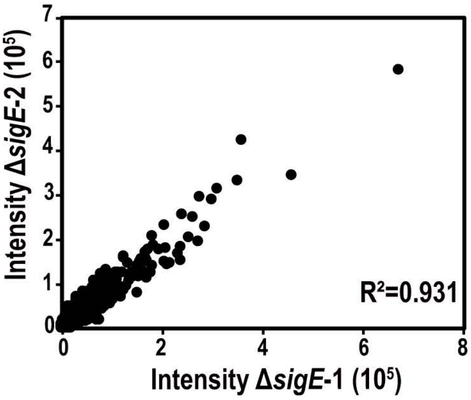

R^2 (Δ*sigE*)

	R1	R2	R3
R1	1		
R2	0.931	1	
R3	0.928	0.930	1

D

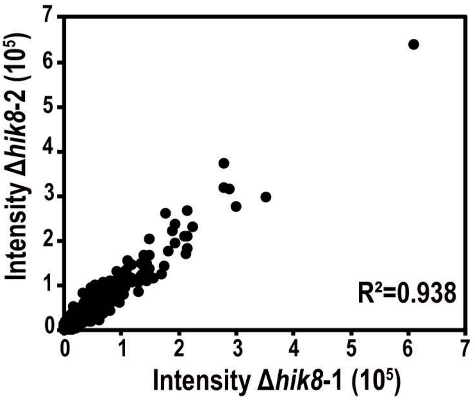

R^2 (Δ*hik8*)

	R1	R2	R3
R1	1		
R2	0.938	1	
R3	0.921	0.934	1

E

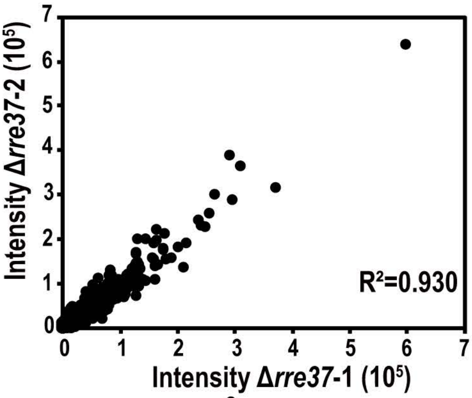

R^2 (Δ*rre37*)

	R1	R2	R3
R1	1		
R2	0.930	1	
R3	0.933	0.911	1

F

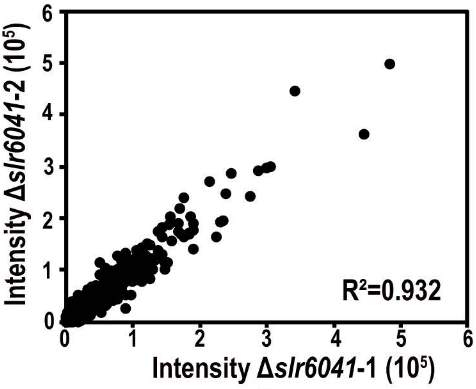

R^2 (Δ*slr6041*)

	R1	R2	R3
R1	1		
R2	0.932	1	
R3	0.922	0.912	1

Supplemental Fig. S3. Correlational analyses of the TMT reporter intensities among the triplicated samples. Pair-wised comparison of the normalized reporter ion intensities among the triplicated samples in the WT and each mutant in the experiments. In each experiment three biological replicates were included (R1-R3). The scatter-plots show the comparison between two representative biological replicates in each experiment. The corresponding correlation coefficient (R^2) values for each comparison are shown in the tables.

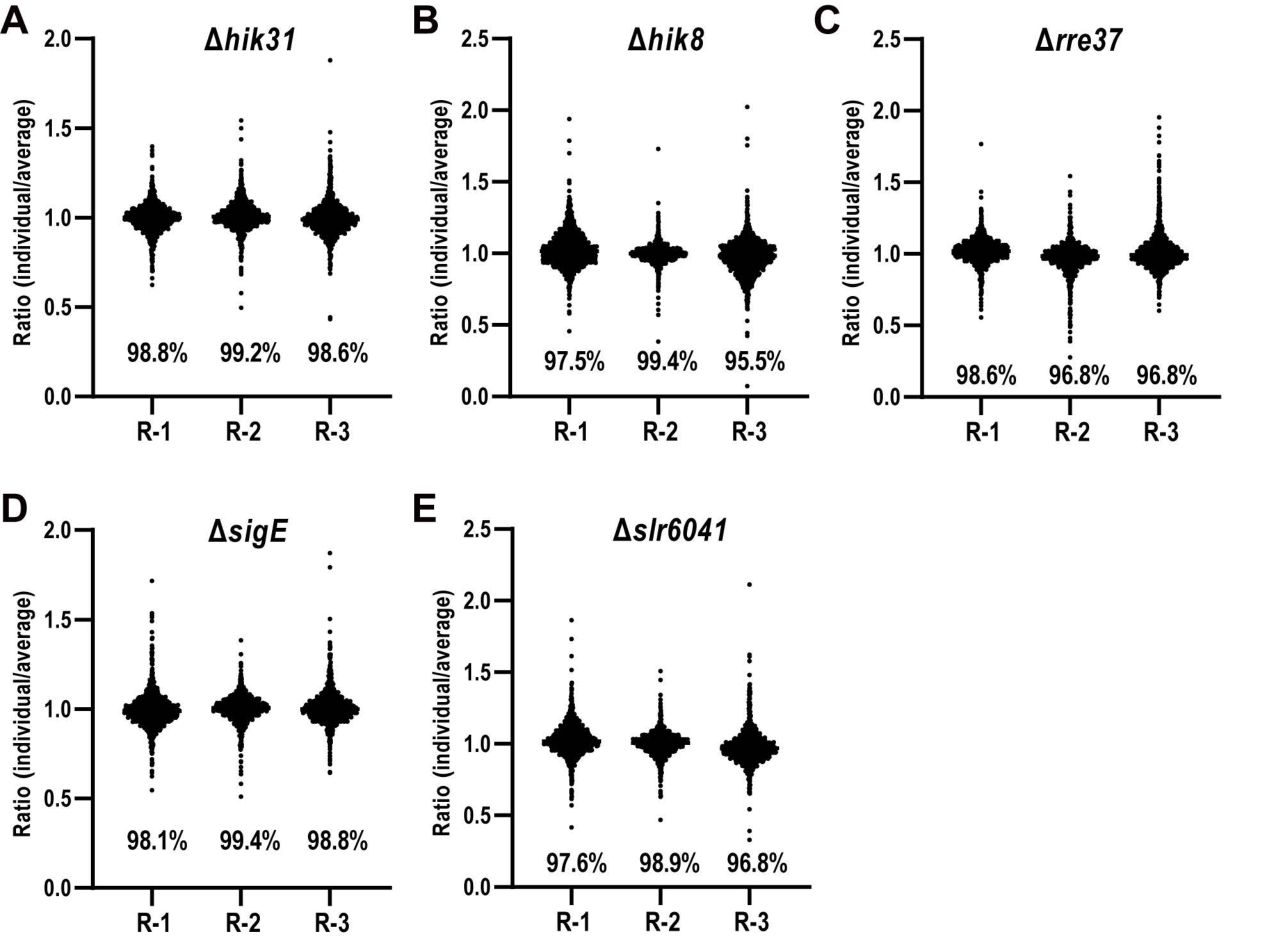

Supplemental Fig. S4. Determination of the fold change threshold for DEPs.

(A-E) For each mutant the TMT ratios (mutant/WT) were firstly calculated for all proteins in all three replicates, and the average of the TMT ratio for each replicate was then calculated. Each individual ratio in the replicate was compared with the average ratio (individual/average) and the distribution of the resulting ratios was calculated and plotted for all replicates of all mutants. The percentage in the figure shows the precentage of the ratios that are smaller than 1.3.

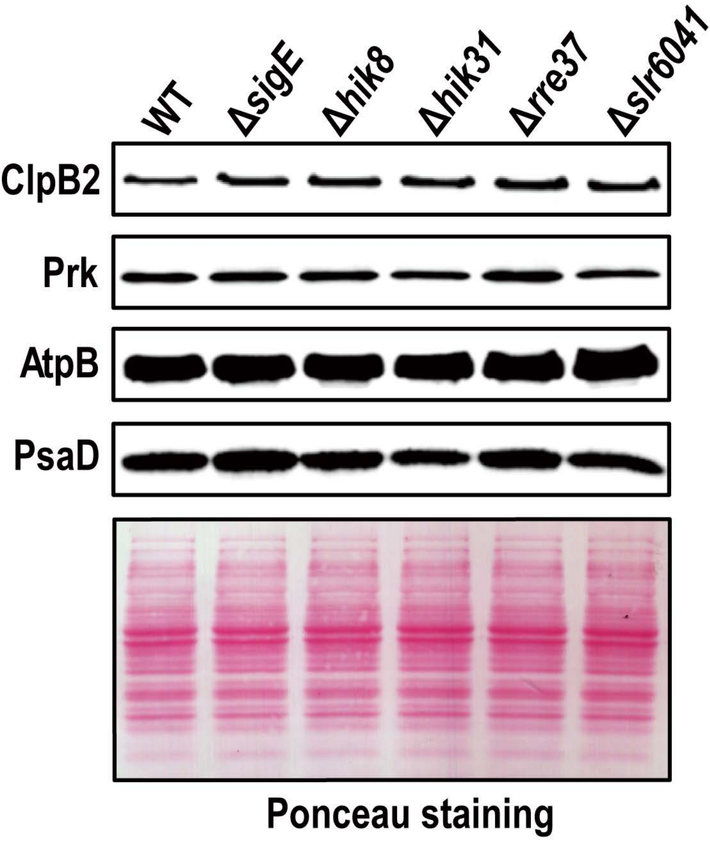

Supplemental Fig. S5. Western-blotting validation of a few proteins without significant changes in abundance among the WT and the mutants. Ponceau staining was used as the loading control.

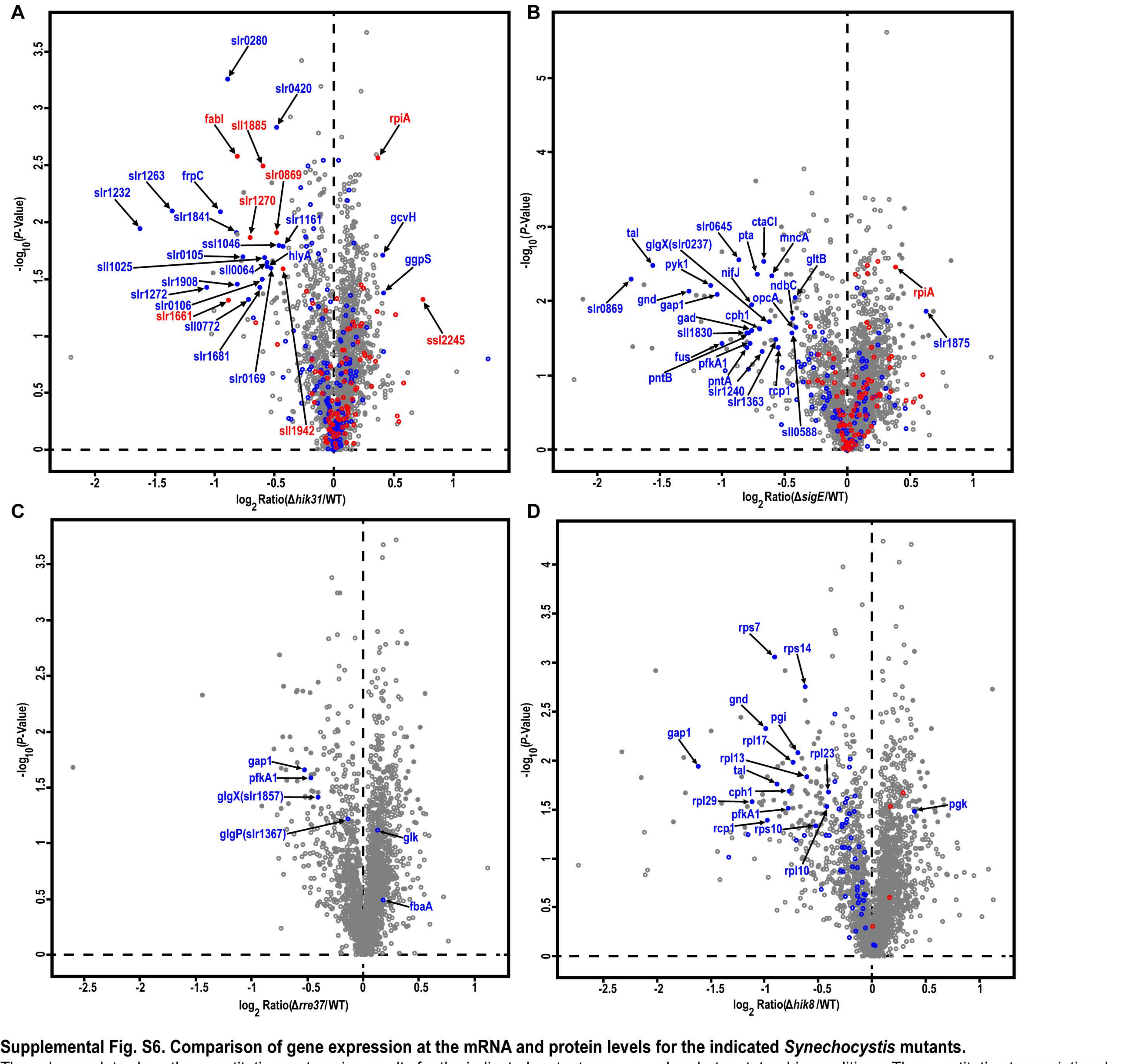

Supplemental Fig. S6. Comparison of gene expression at the mRNA and protein levels for the indicated *Synechocystis* mutants.

The volcano plots show the quantitative proteomics results for the indicated mutants grown under photoautotrophic conditions. The quantitative transcriptional information was either collected from reported DNA-microarray data (Δ*hik31*, Δ*sigE*), or manually annotated for individual gene from the literature (Δ*hik8*, Δ*rre37*) in the mutant grown under the similar trophic conditions. The mRNA levels were mapped to the corresponding proteins and displayed either in blue (downregulation relative to the WT) and in red (upregulation relative to the WT). Filled cycles: differentially expressed proteins (DEPs). Cycles: non-DEPs.

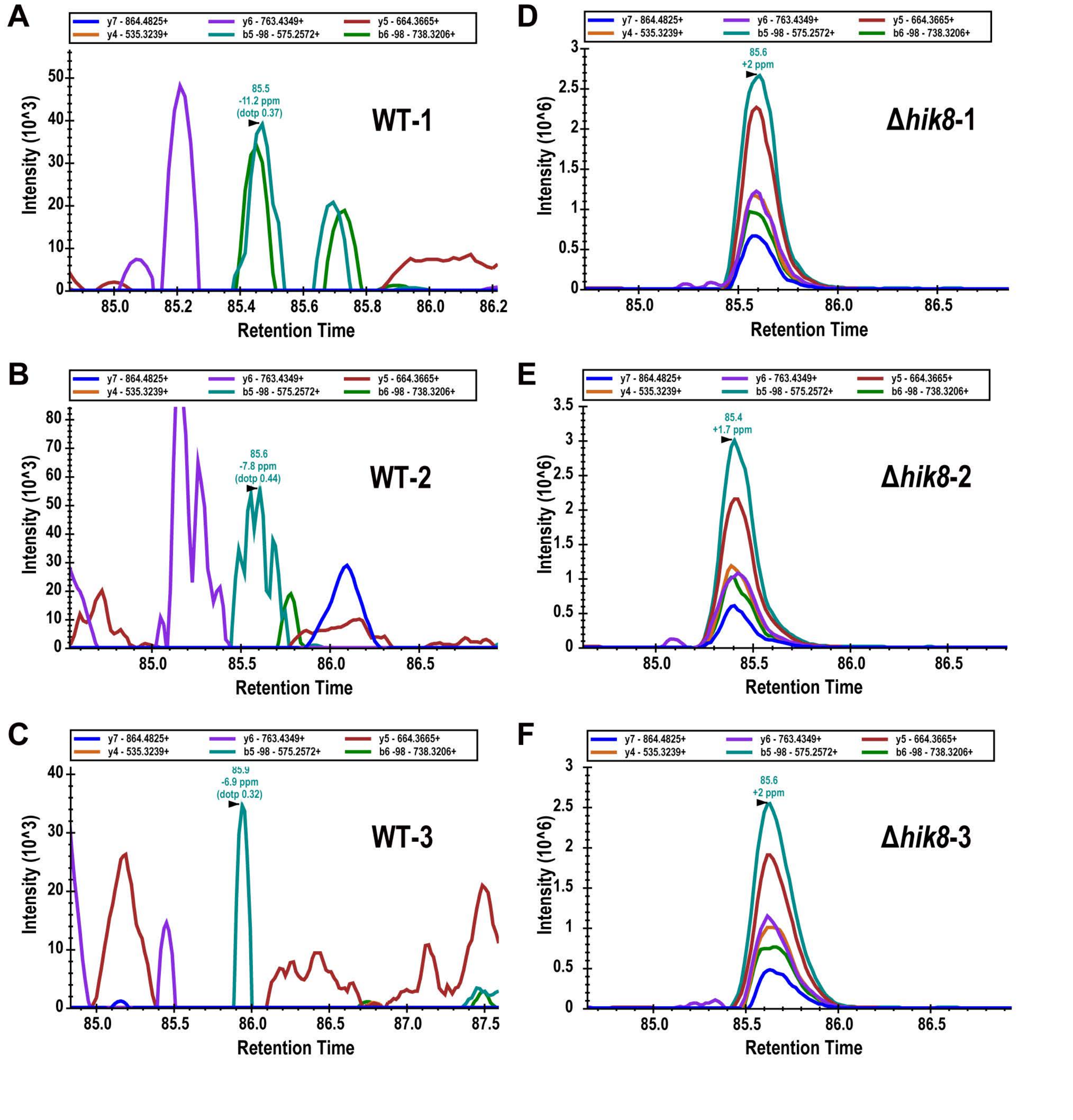

Supplemental Fig. S7. Measurement of P_{II} S49 phosphorylation in AT-grown WT and Δ*hik8* through PRM. Shown are the extracted-ion chromatograms (XICs) of the fragment ions from the peptide in Fig. 6*D*.

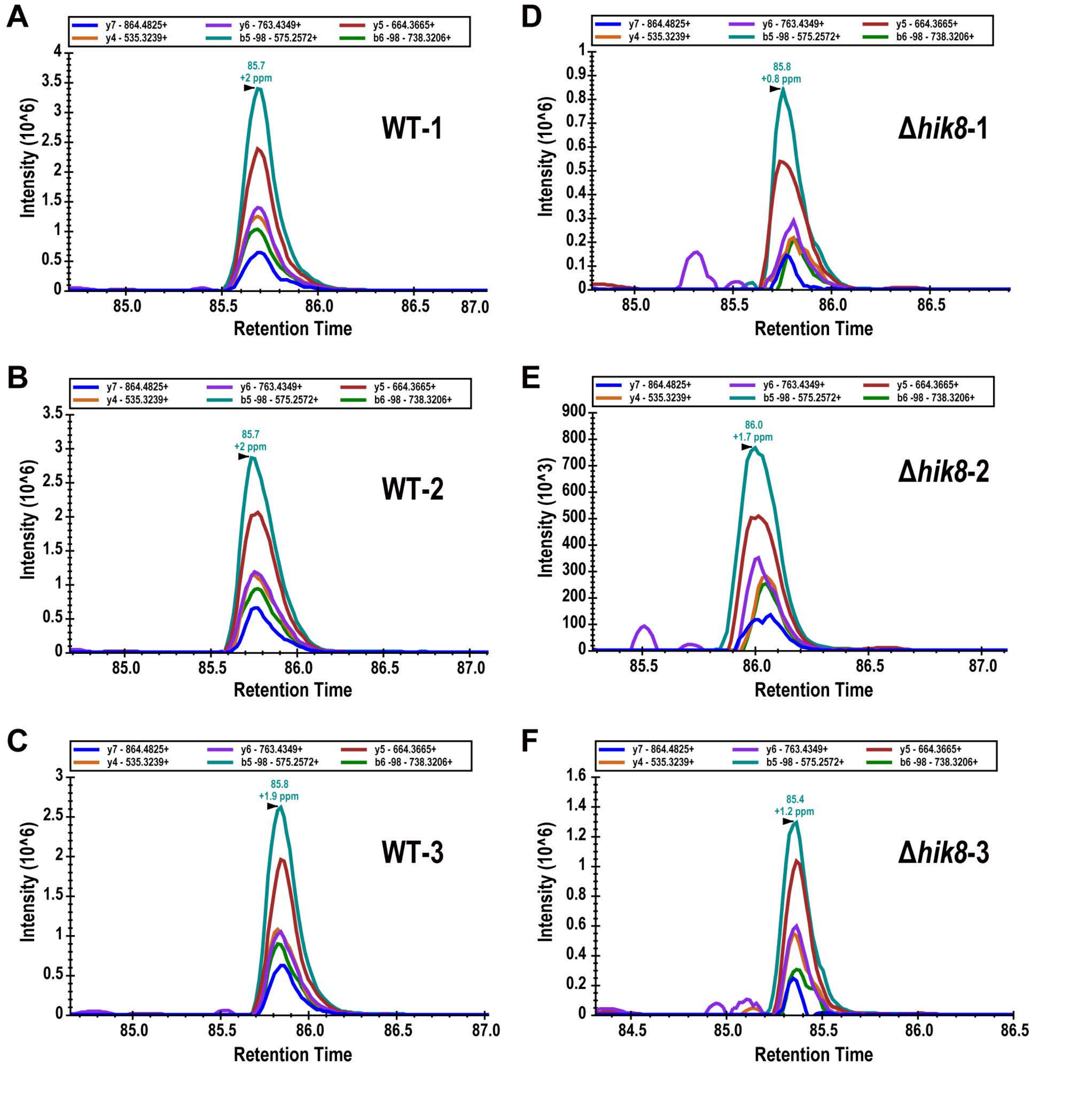

Supplemental Fig. S8. Measurement of P_{II} S49 phosphorylation in MT-grown WT and Δ*hik8* through PRM. Shown are the XICs of the fragment ions from the peptide in Fig. 6*D*.

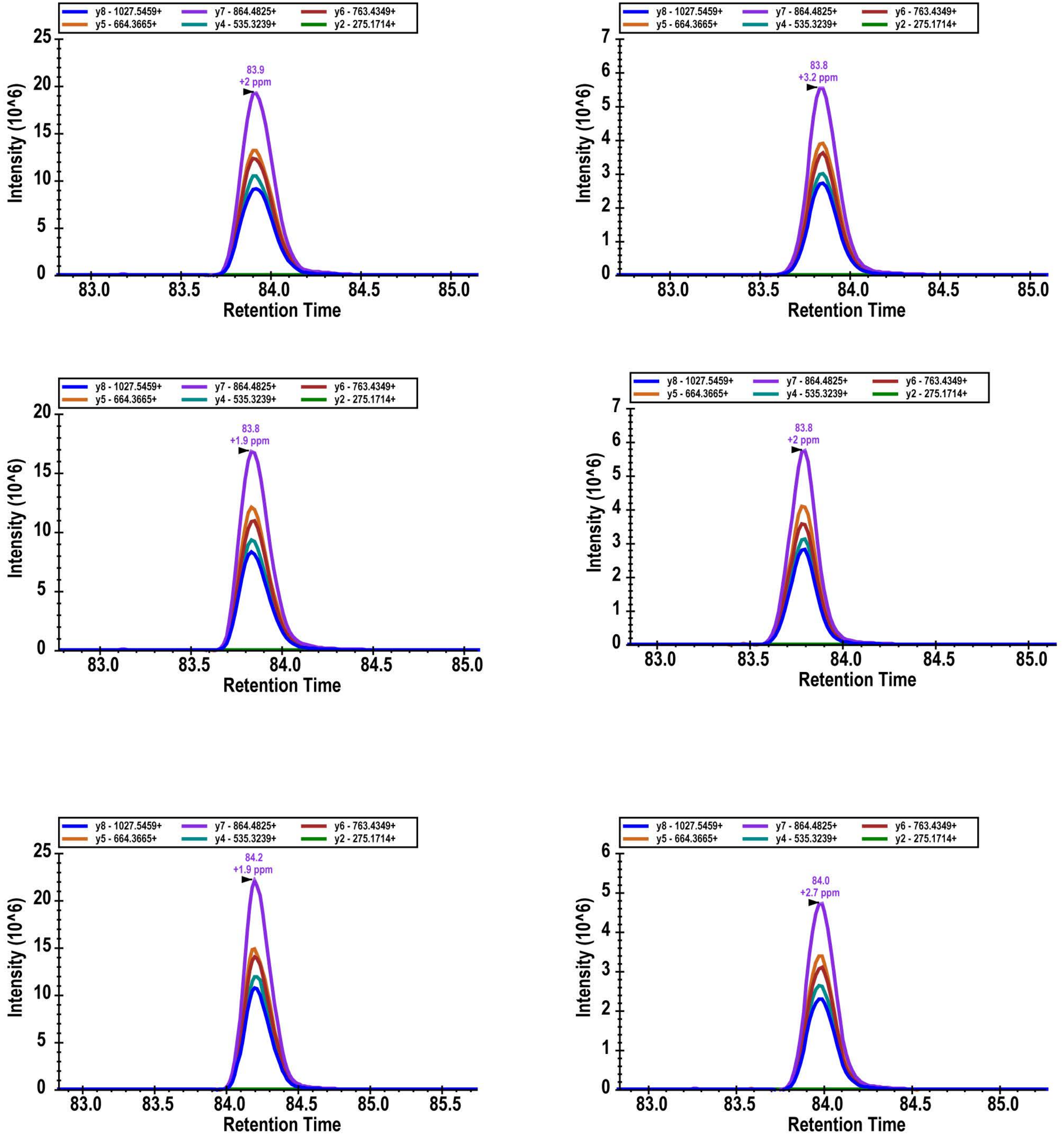

Supplemental Fig. S9. Measurement of the peptide GSEYTVEFLQK in AT-grown WT and Δ*hik8* through PRM.

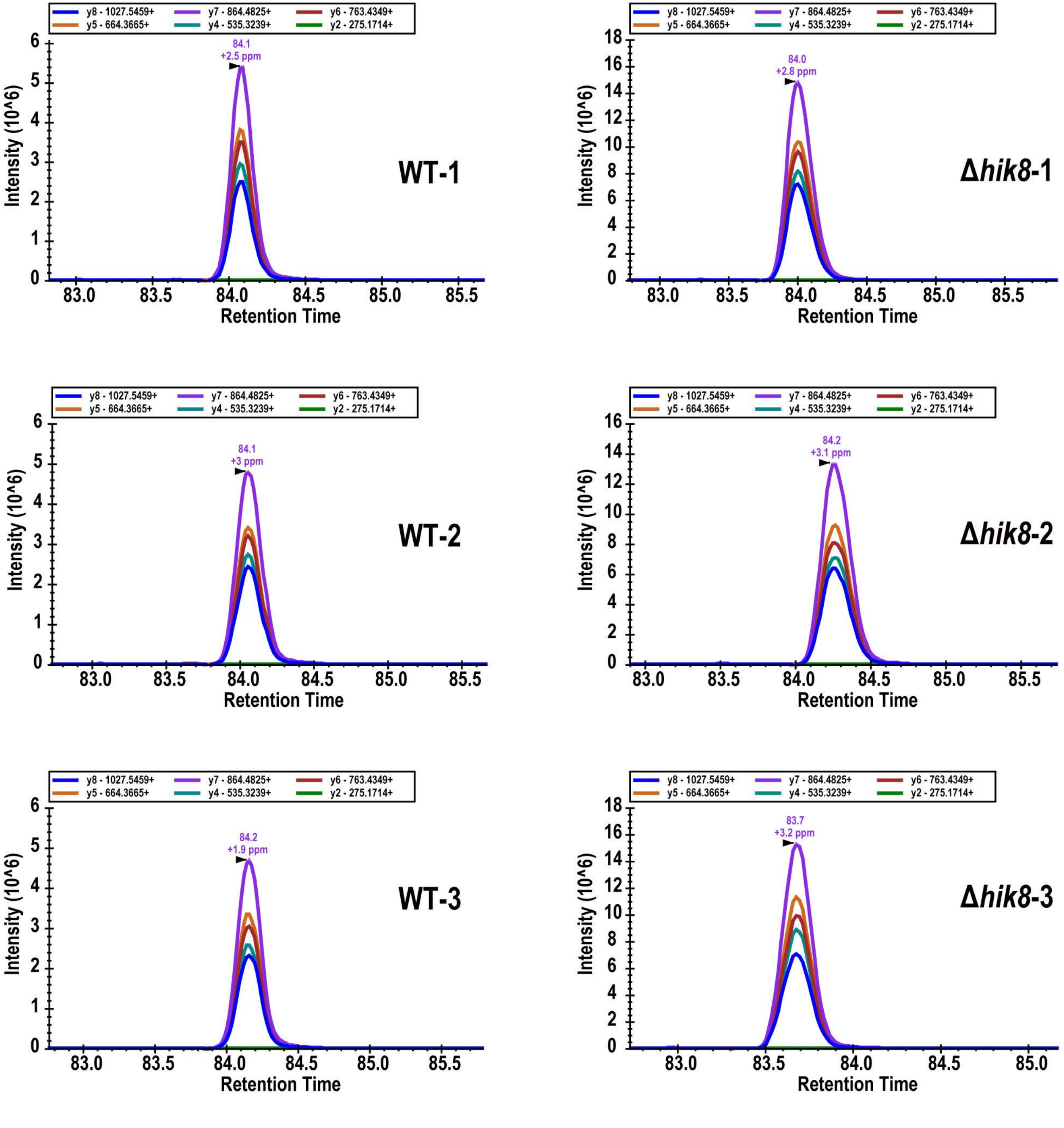

Supplemental Fig. S10. Measurement of the peptide GSEYTVEFLQK in MT-grown WT and Δ*hik8* through PRM.

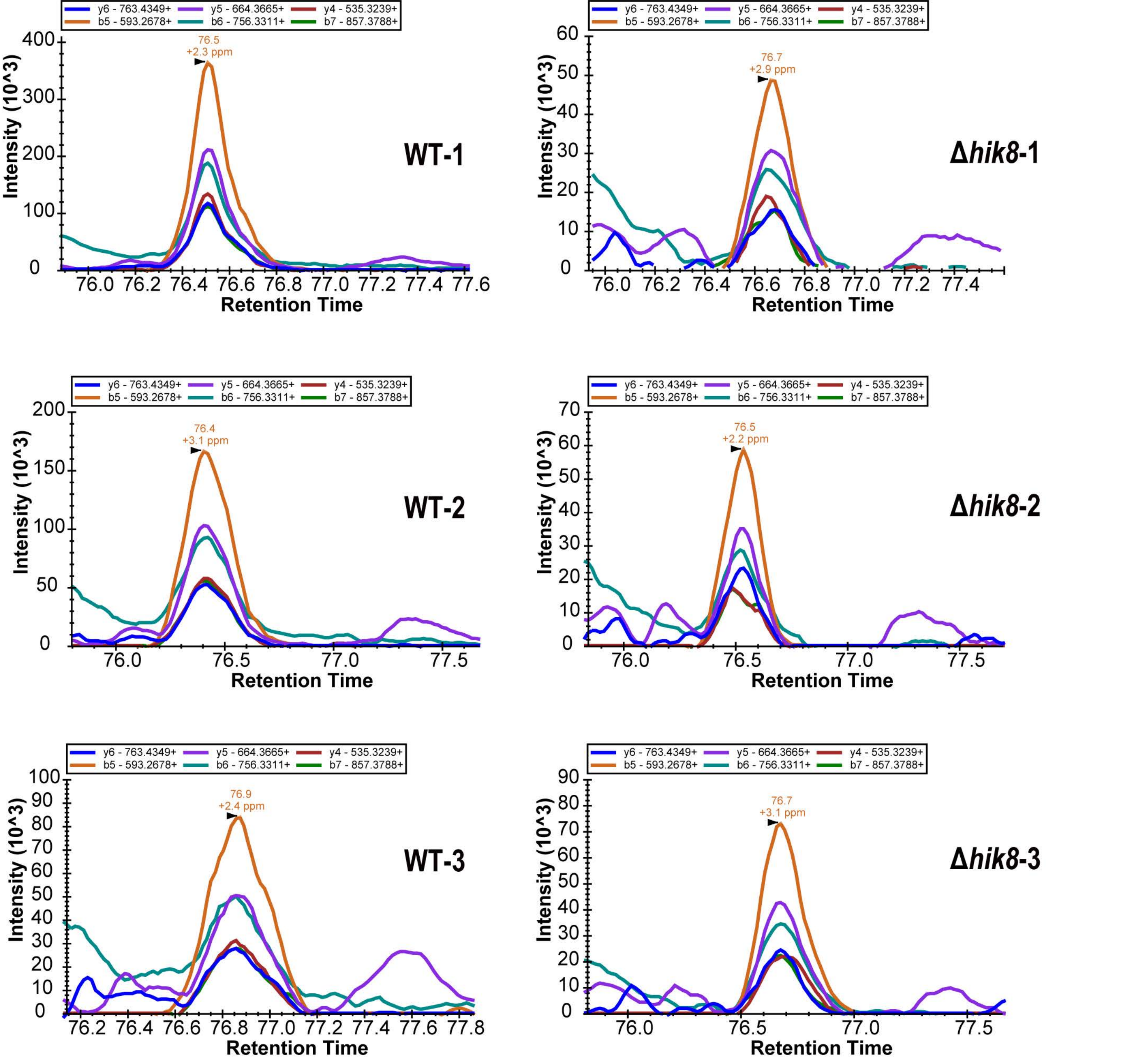

Supplemental Fig. S11. Measurement of the peptide YRGSEYTVEFLQK in AT-grown WT and Δ*hik8* through PRM.

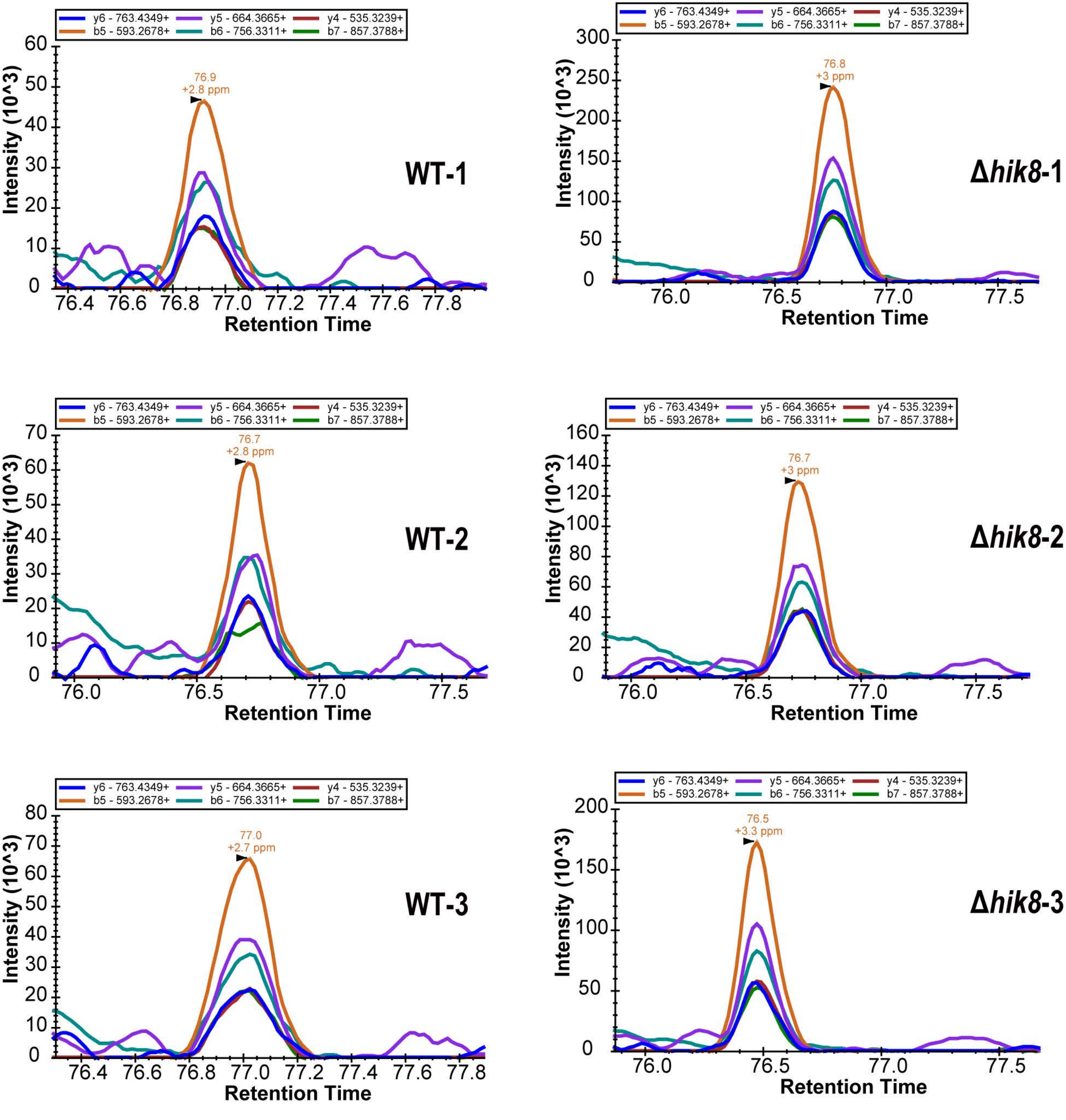

Supplemental Fig. S12. Measurement of the peptide YRGSEYTVEFLQK in MT-grown WT and Δ*hik8* through PRM.

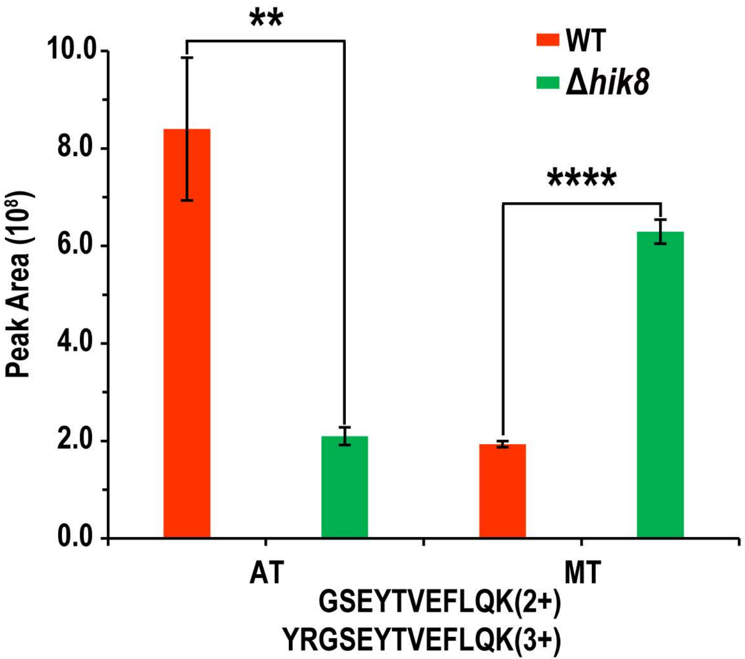

Supplemental Fig. S13. PRM-quantified peak area of the non-phosphorylated peptides containing P_{II} S49 site in AT/MT-grown WT and Δ*hik8*. **: *p* value < 0.01, ****: *p* value < 0.0001.

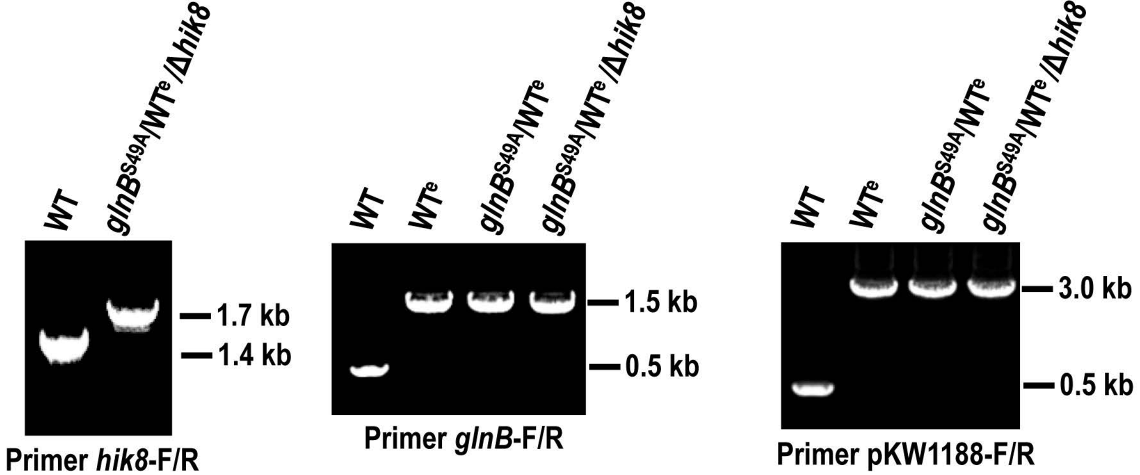

Supplemental Fig. S14. Confirmation of the mutants with P_{II} S49A substitution. The complete segregation of the indicated mutants was confirmed by PCR using the corresponding primers (supplemental Table S1).

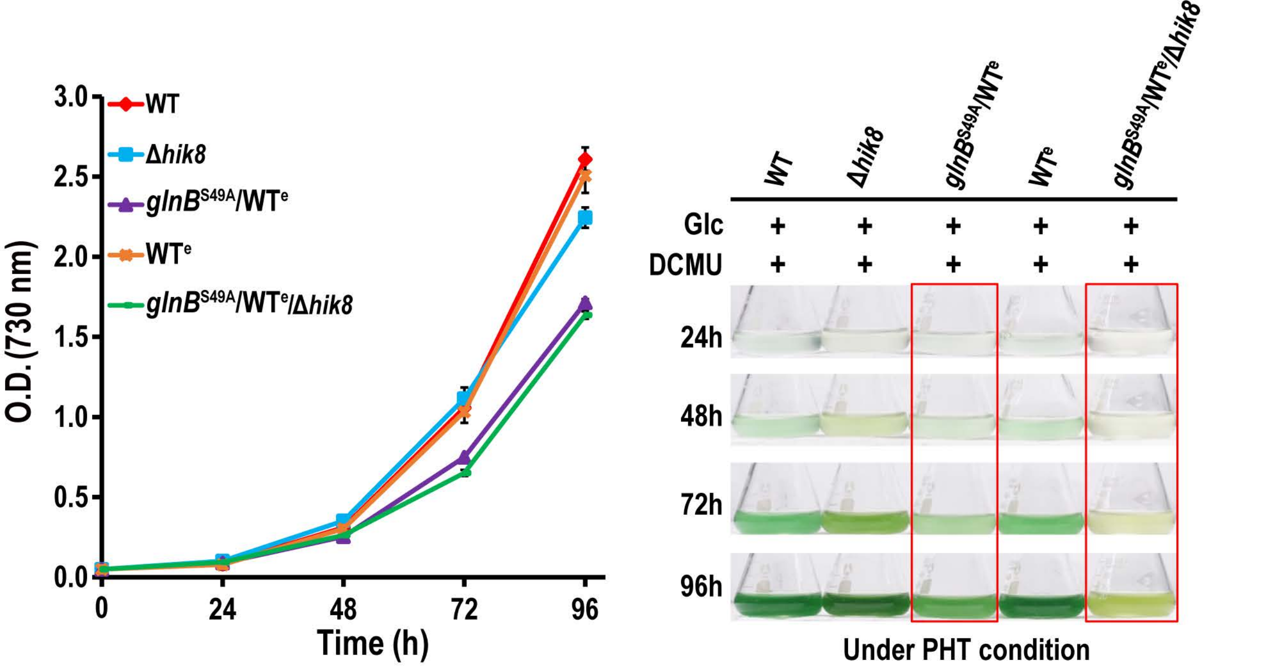

Supplemental Fig. S15. Growth curves of the WT, Δ*hik8*, and *glnB*^{S49A}/WT^{e} under the PHT conditions. The images of cell cultures were taken at the indicated time points (Right panel).

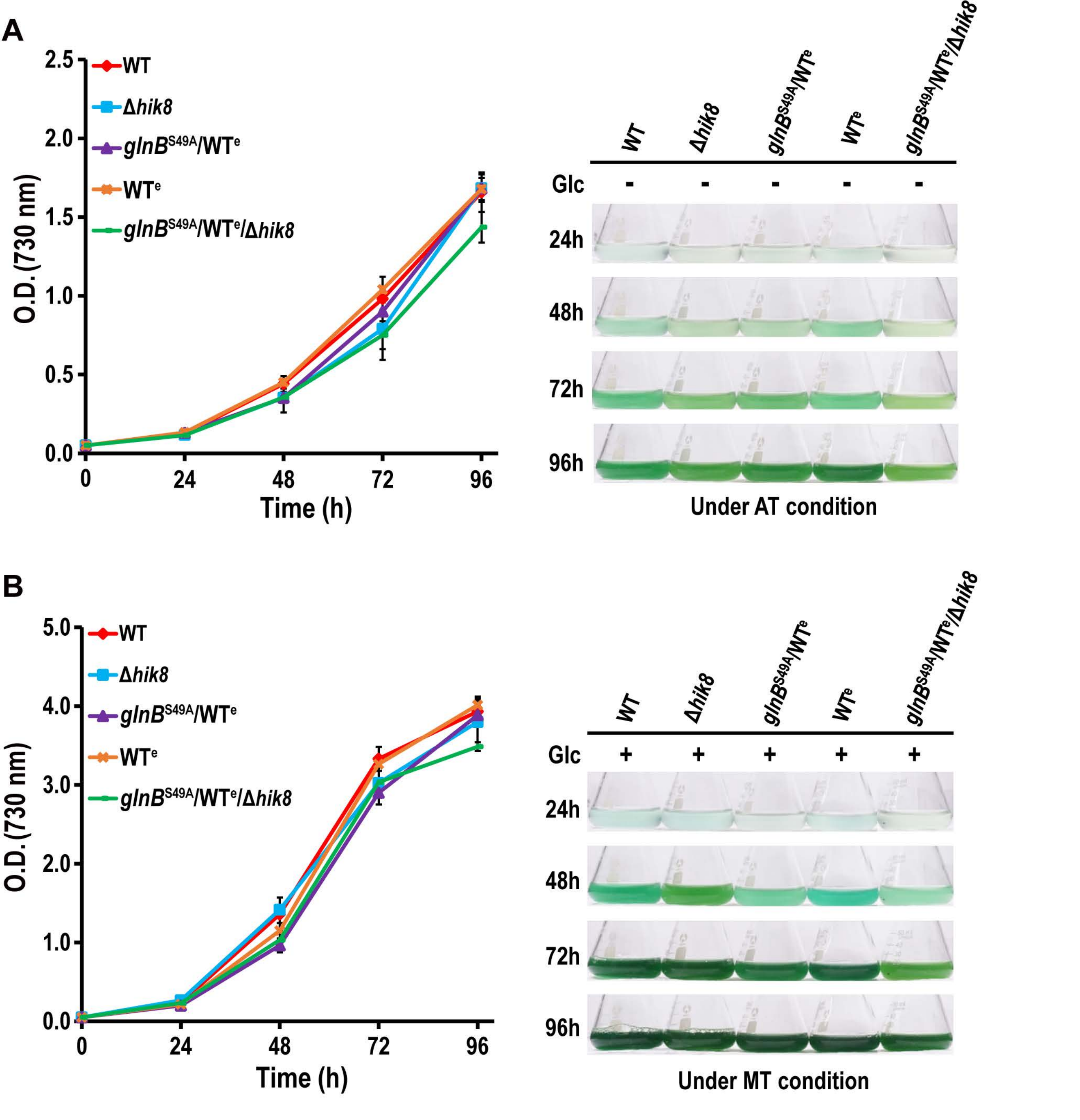

Supplemental Fig. S16. Growth curves of WT, Δ*hik8*, and *glnB^{S49A}*/WT^{e} under AT (A) and MT conditions (B). The images of cell cultures were taken at the indicated time points (Right panel).

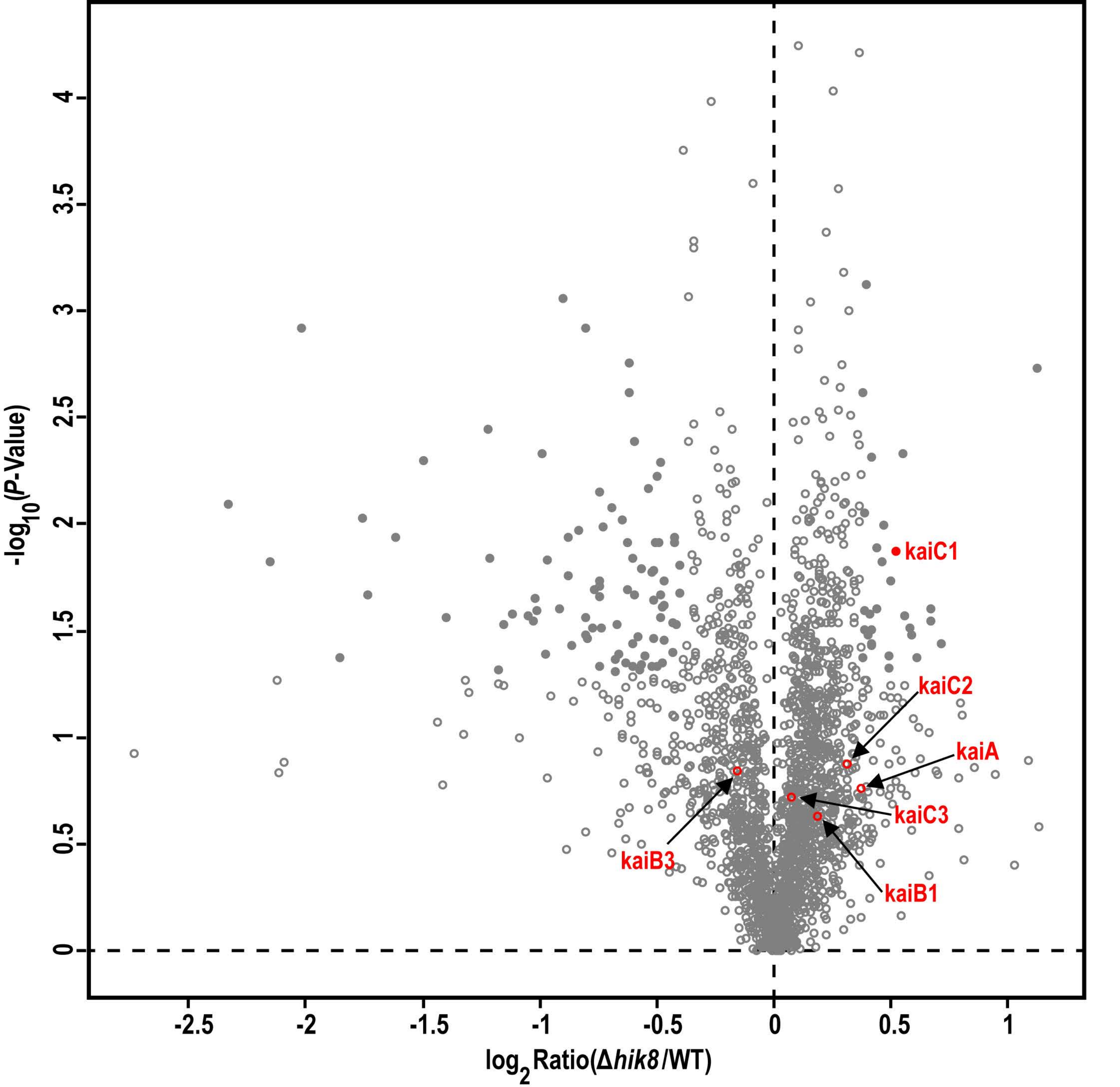

Supplemental Fig. S17. The volcano plot shows all proteins quantitatively analyzed by TMT in Δ*hik8*.
X-axis, the fold change (log_2 transformed) of the abundance in Δ*hik8* relative to the WT, y-aixs, the logarithm-transformed *p*<-value. Filled cycles: DEPs in Δ*hik8*. Proteins involved in circadian clock are labeled.

Supplemental Fig. S18.

Annotated spectra for all proteins identified with a single peptide.

Raw File	Scan	Method	Score	m/z
HCC_TMT_2_F3_20180514	16926	FTMS; HCD	160.36	895.04

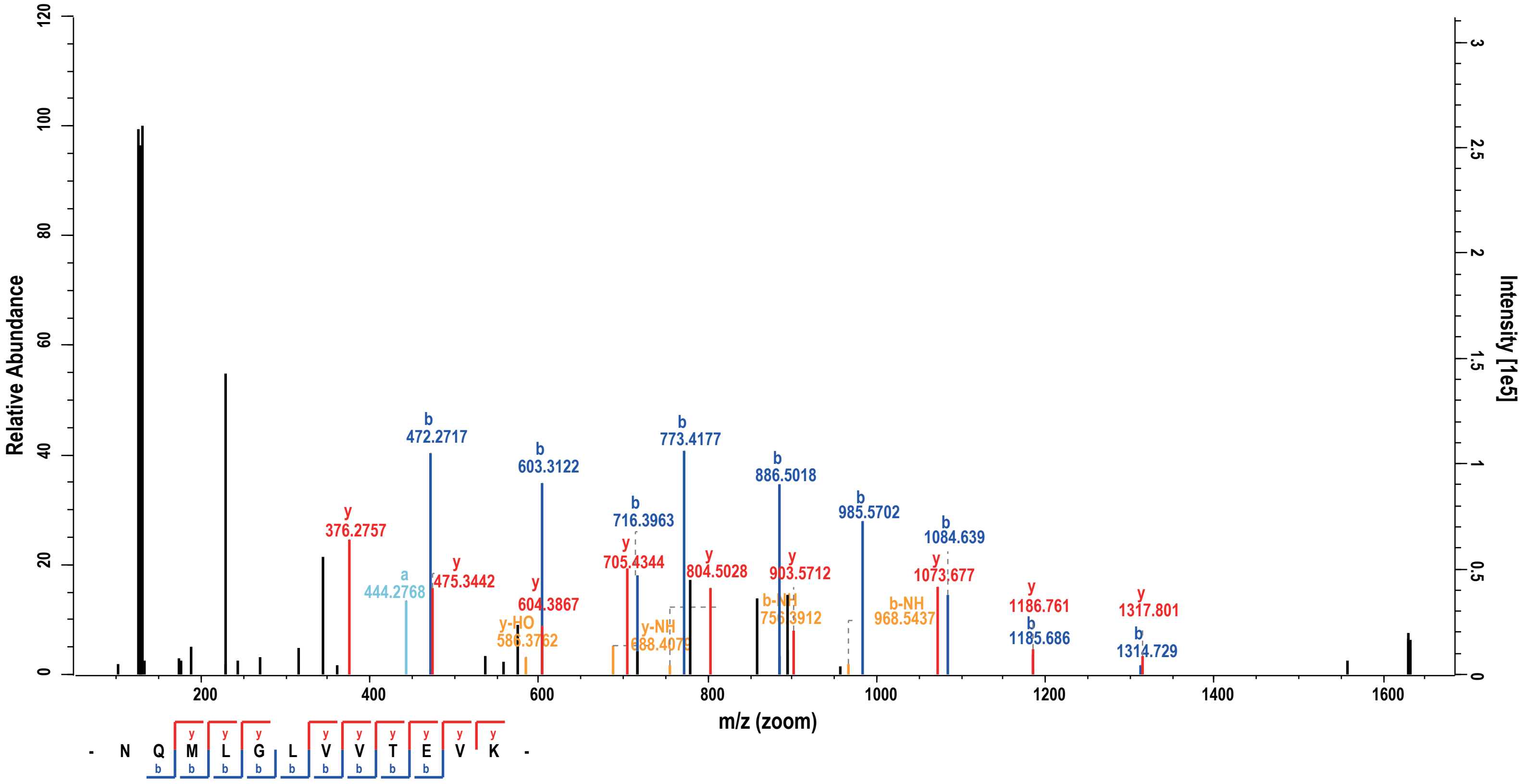

sll0040 positive phototaxis protein, homologous to chemotaxis protein CheW

Raw File	Scan	Method	Score	m/z
HCC_TMT_2_F11_20180514	5876	FTMS; HCD	219.43	825.93

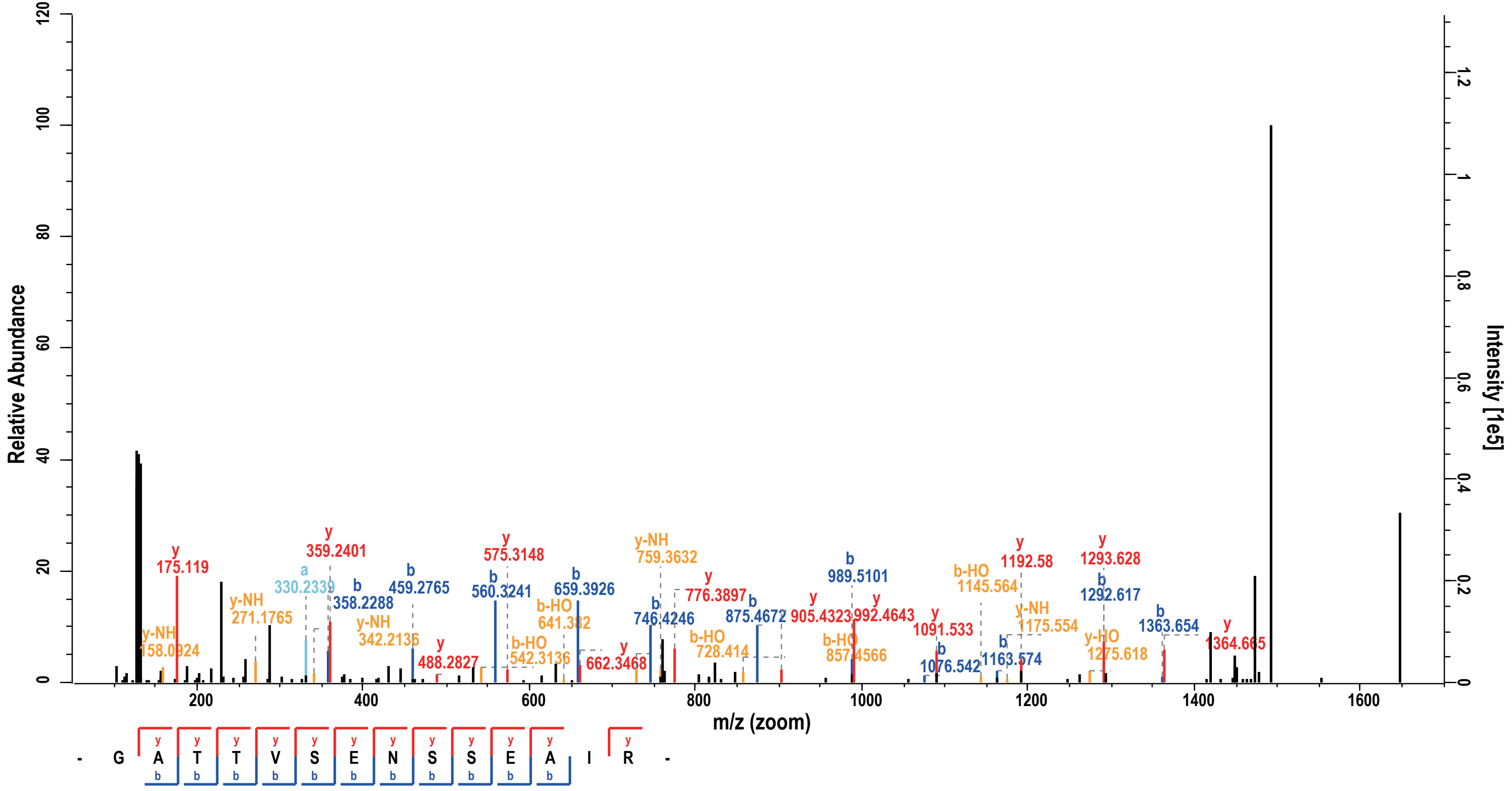

sll0109 chorismate mutase

Raw File	Scan	Method	Score	m/z
HCC_TMT_1_F3_20180514	13955	FTMS; HCD	144.09	744.41

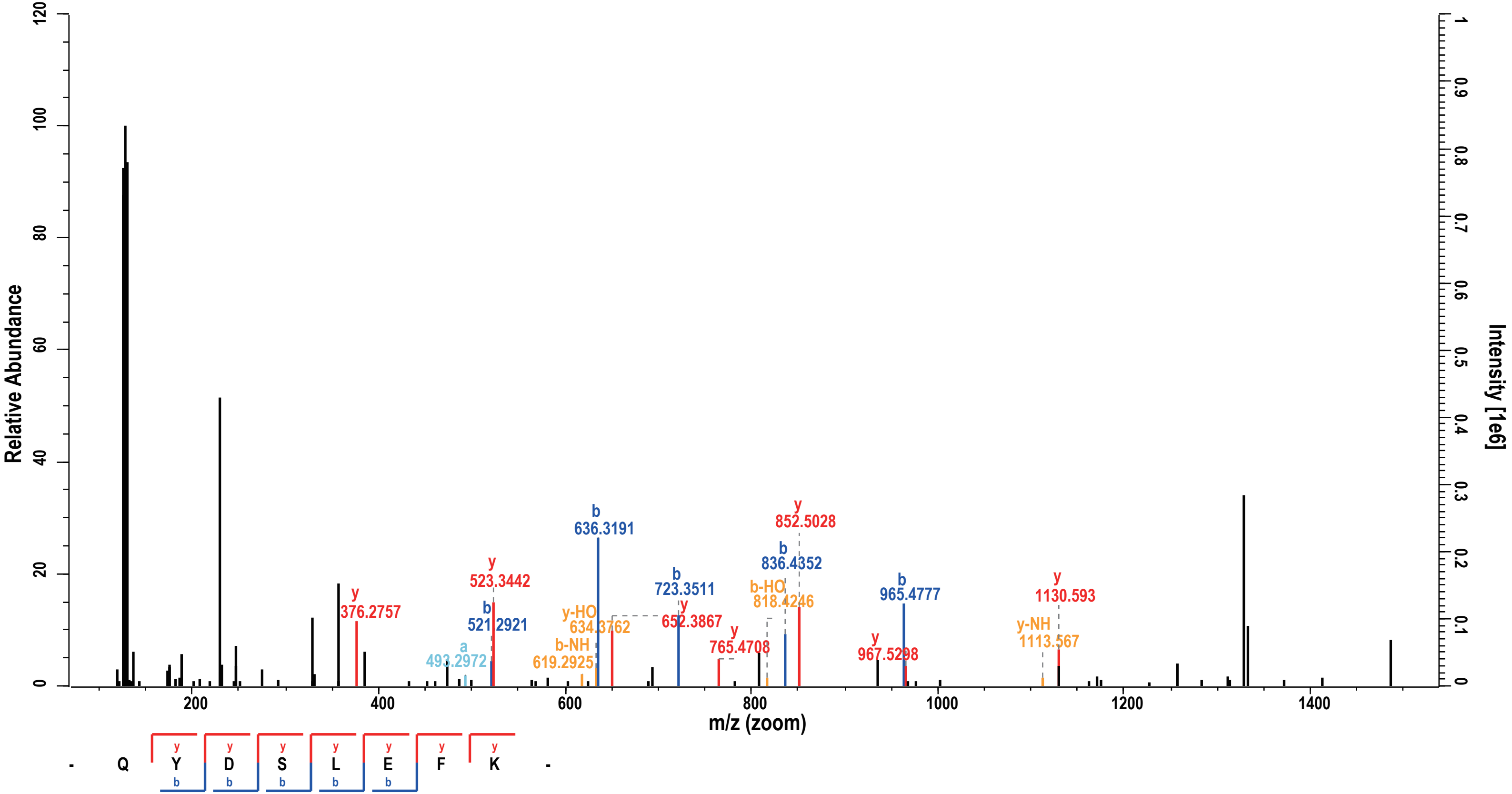

sll0210 bacitracin resistance protein

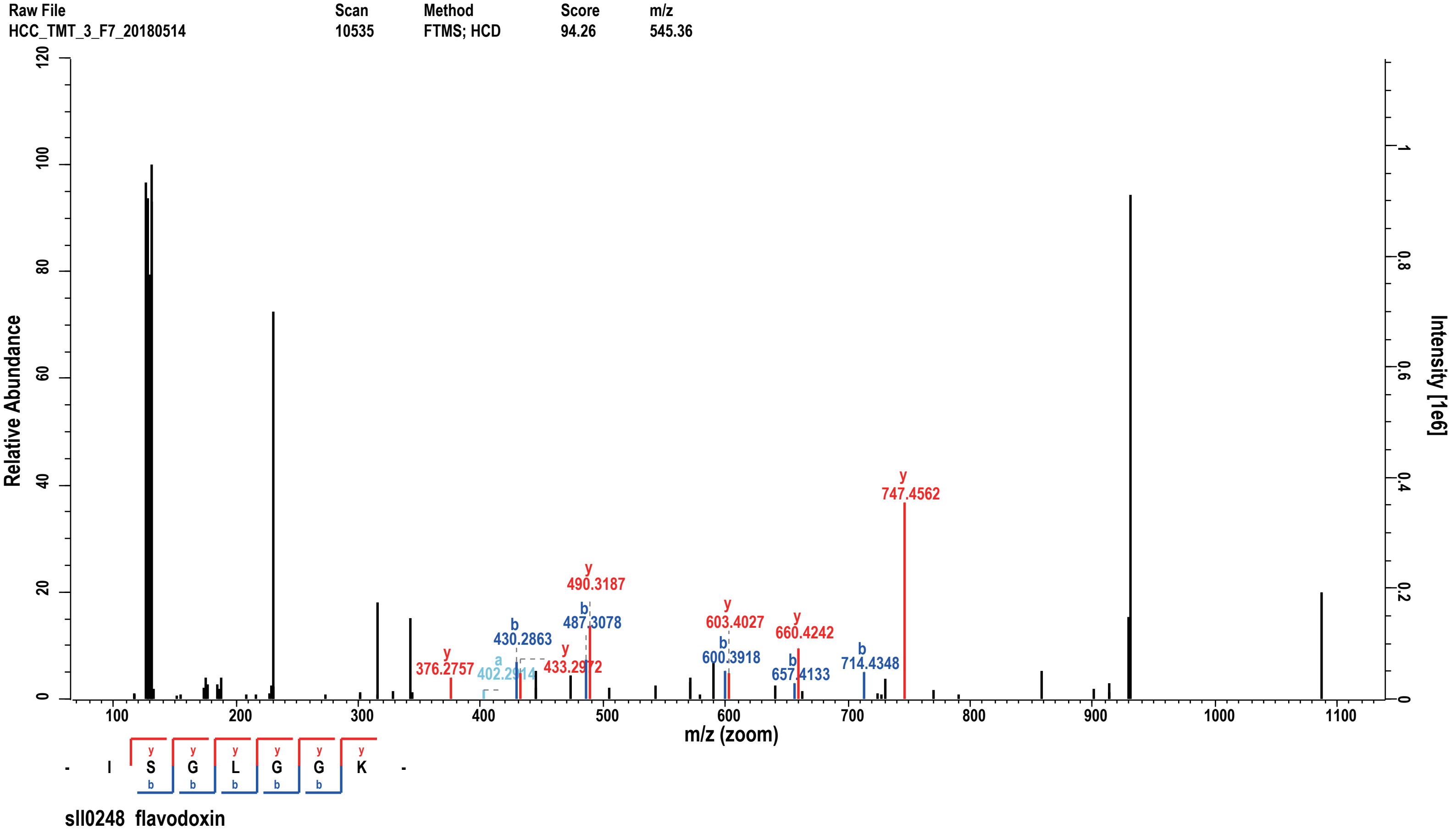
Raw File
HCC_TMT_3_F7_20180514
Scan
10535
Method
FTMS; HCD
Score
94.26
m/z
545.36
Relative Abundance
Intensity [1e6]
m/z (zoom)
y
376.2757
a
402.2914
b
430.2863
y
433.2972
b
487.3078
y
490.3187
b
600.3918
y
603.4027
b
657.4133
y
660.4242
b
714.4348
y
747.4562
- I S G L G G K -
sll0248 flavodoxin

Raw File	Scan	Method	Score	m/z
HCC_TMT_1_F13_20180514	15581	FTMS; HCD	129.85	855.47

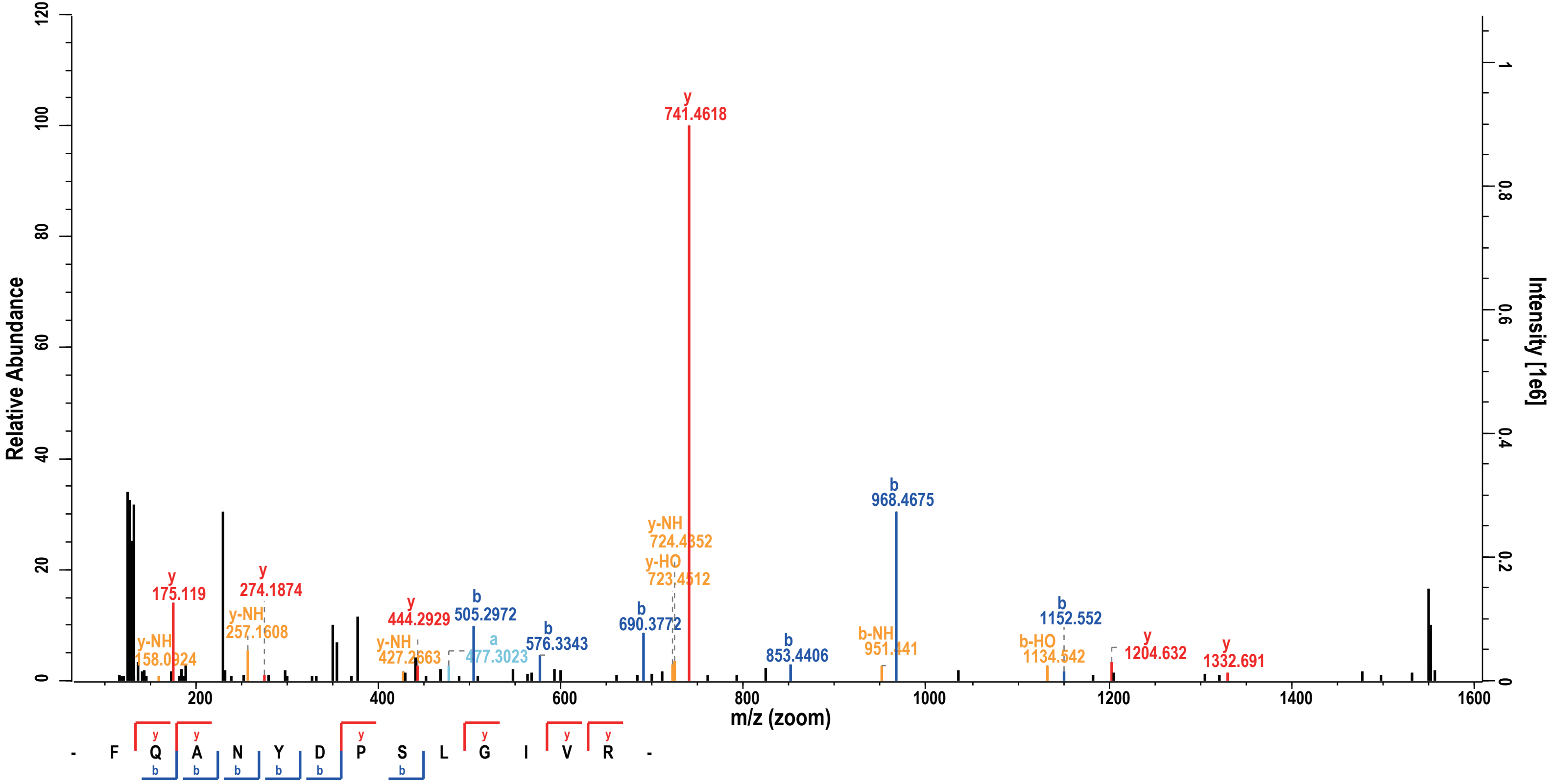

sll0263 unknown protein

Raw File	Scan	Method	Score	m/z
HCC_TMT_3_F4_20180514	16924	FTMS; HCD	111.65	964.52

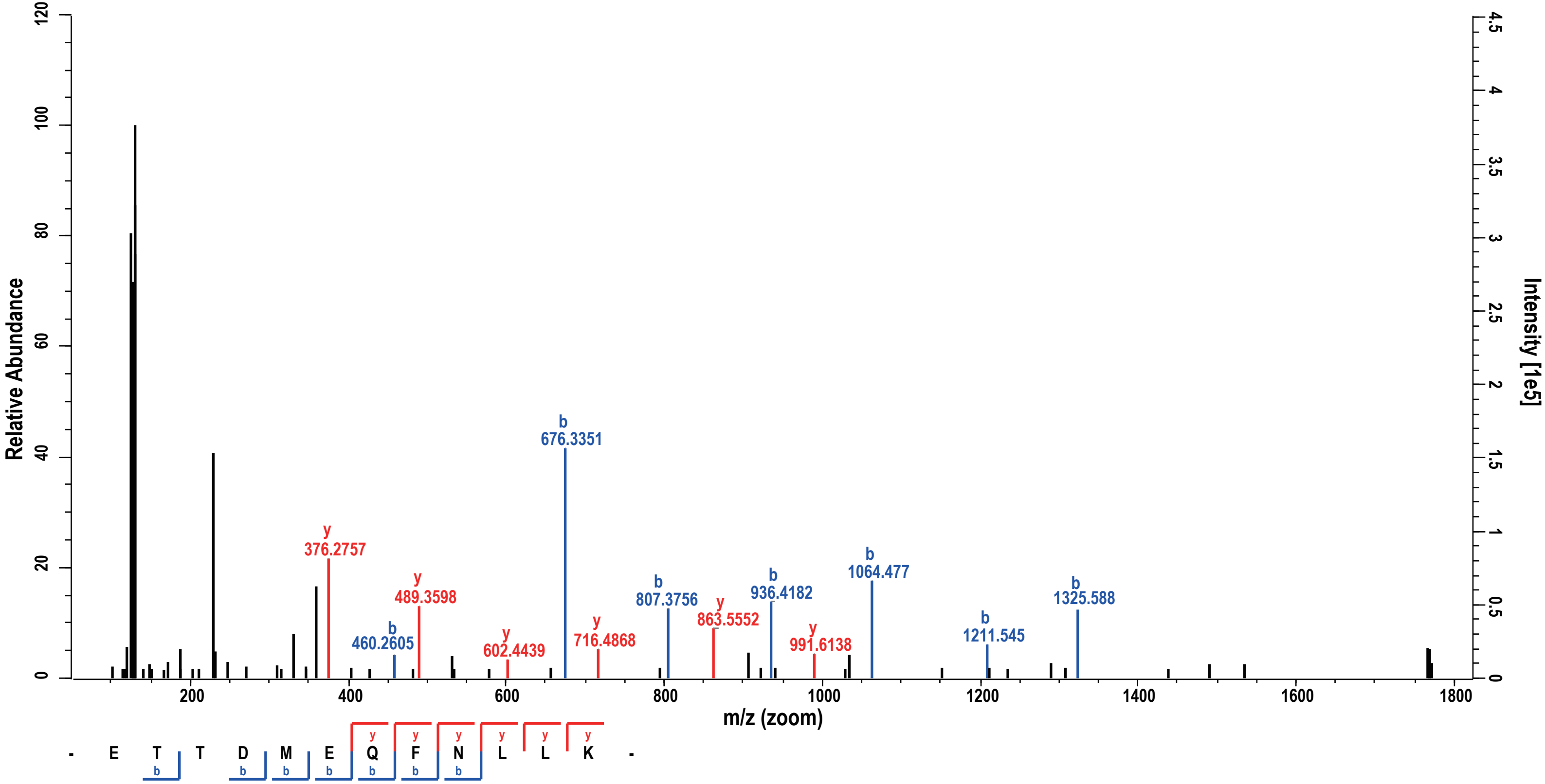

sll0522 NADH dehydrogenase subunit 4L

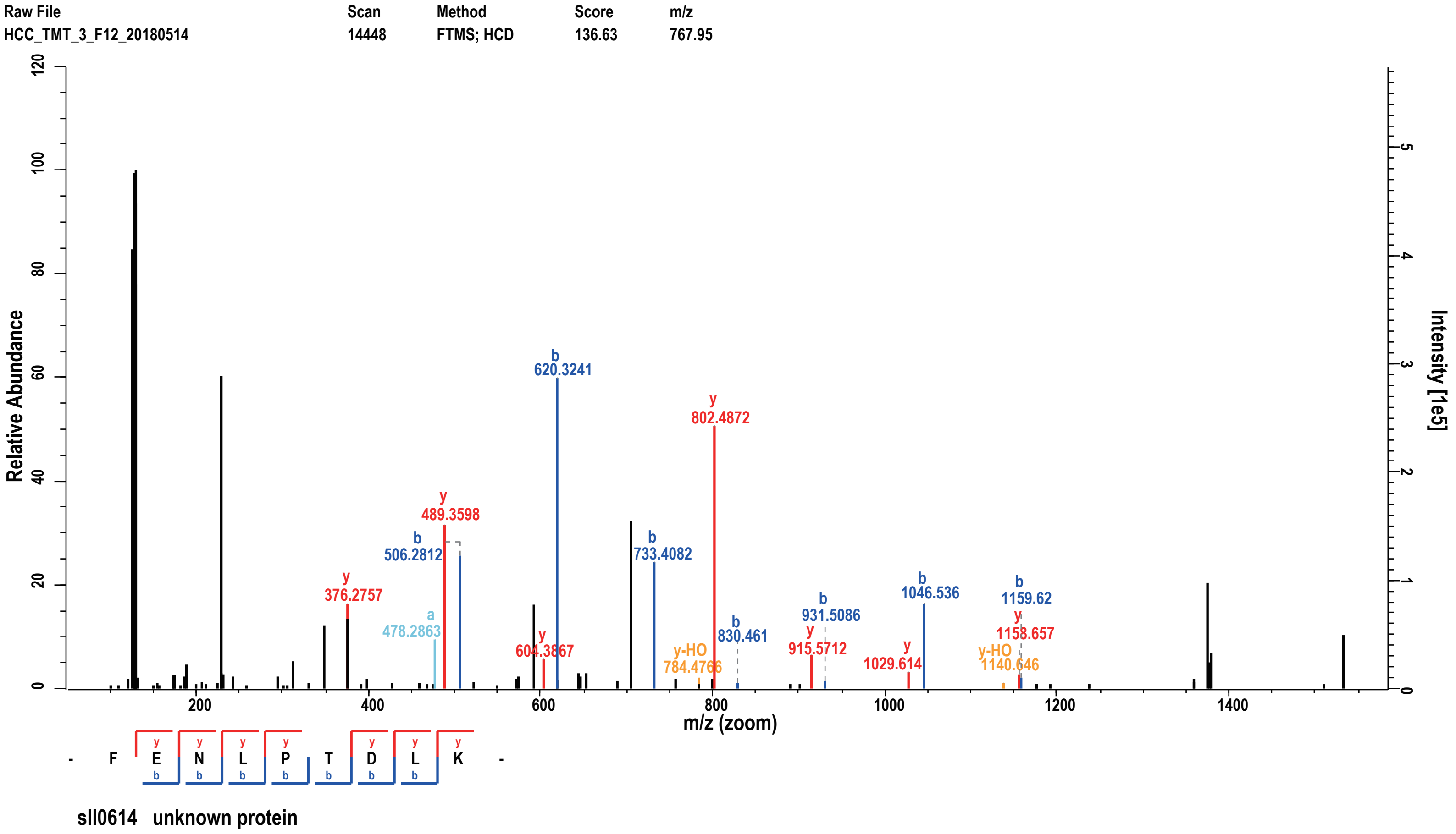
Raw File
HCC_TMT_3_F12_20180514
Scan
14448
Method
FTMS; HCD
Score
136.63
m/z
767.95
Relative Abundance
Intensity [1e5]
m/z (zoom)
y 376.2757
a 478.2863
y 489.3598
b 506.2812
y 604.3867
b 620.3241
b 733.4082
y-HO 784.4766
y 802.4872
b 830.461
y 915.5712
b 931.5086
y 1029.614
b 1046.536
y-HO 1140.646
y 1158.657
b 1159.62
- F E N L P T D L K -
sll0614 unknown protein

Raw File	Scan	Method	Score	m/z
HCC_TMT_1_F3_20180514	16049	FTMS; HCD	145.89	757.97

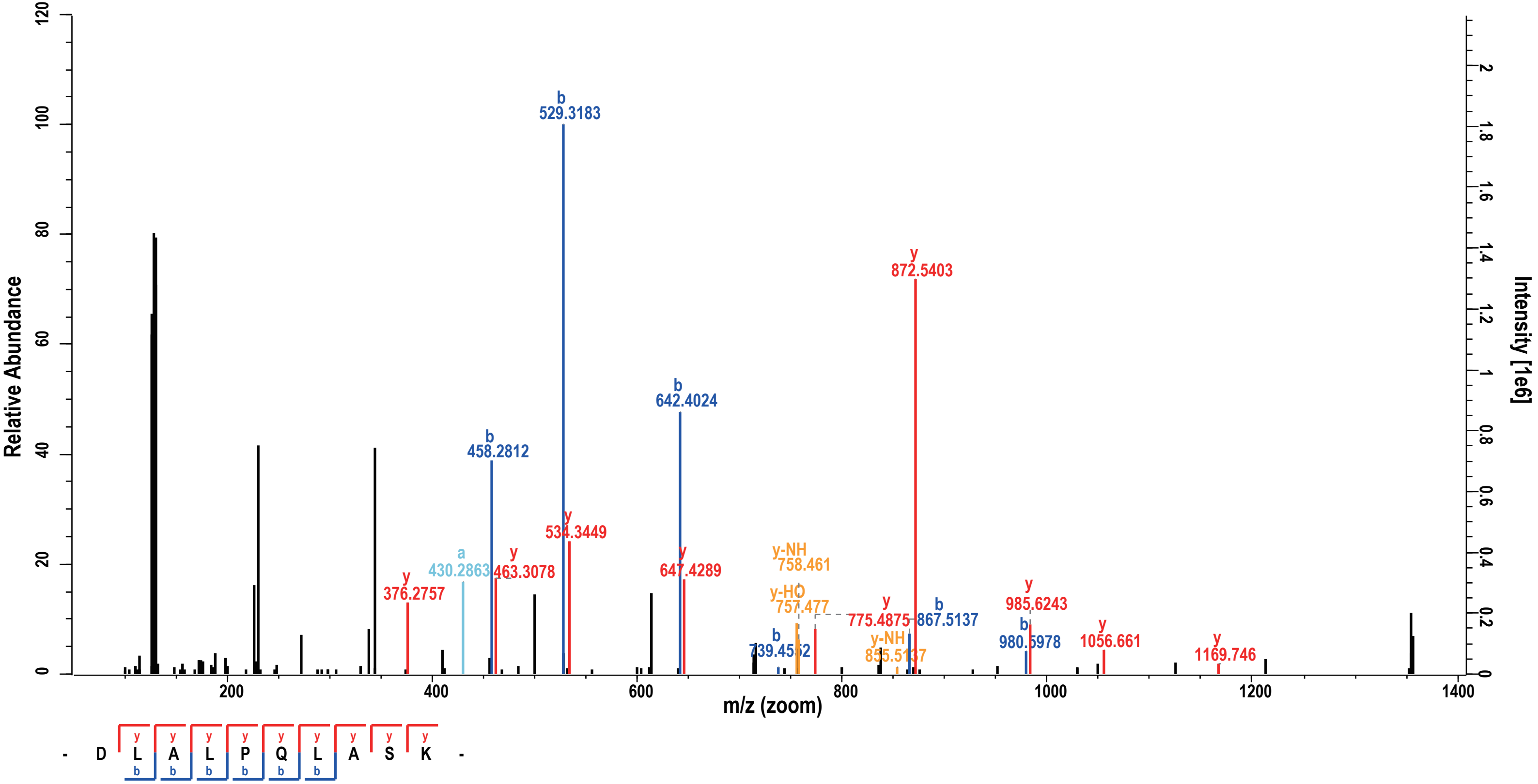

sll0629 alternative photosystem I reaction center subunit X

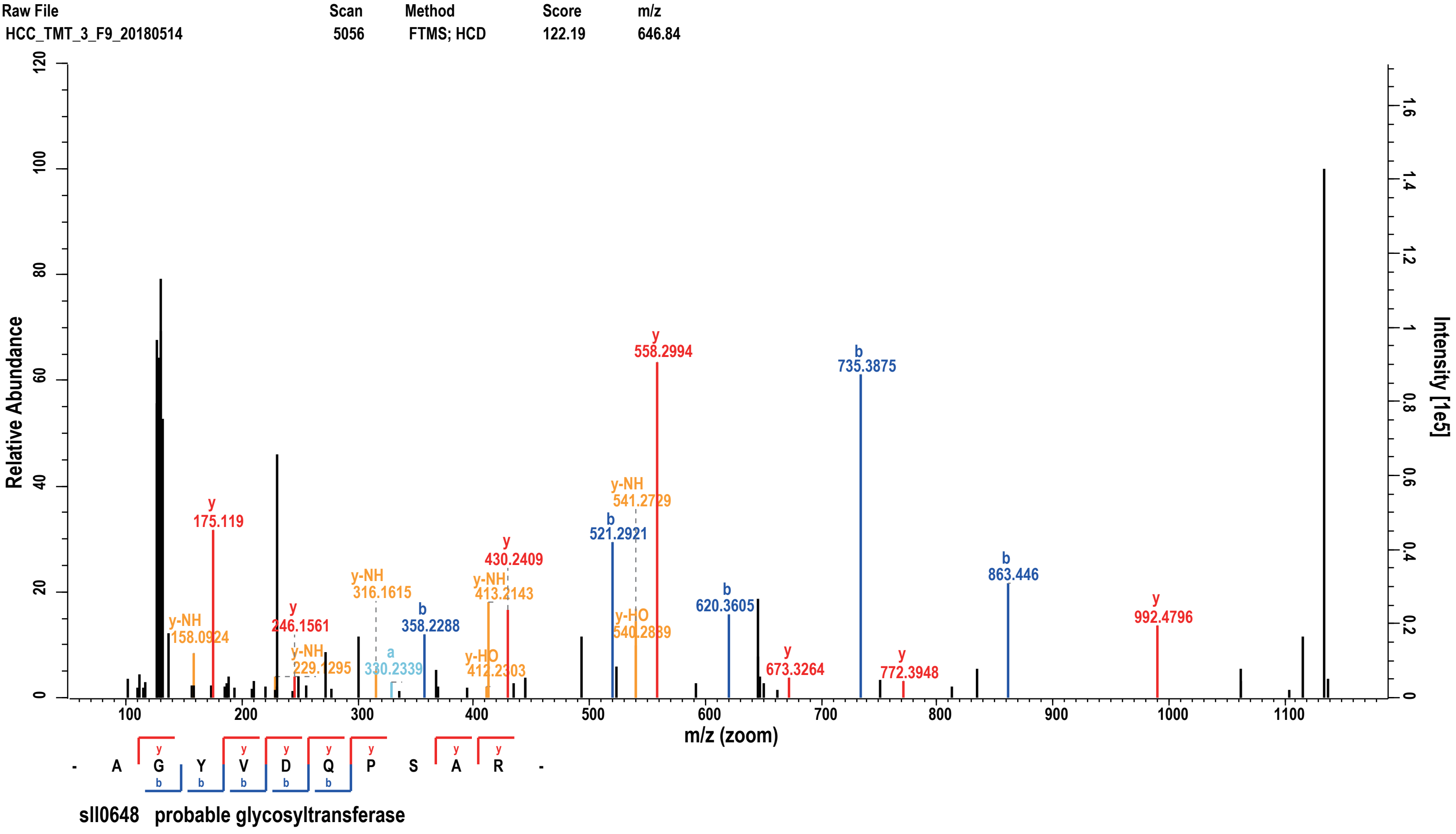
Raw File
HCC_TMT_3_F9_20180514
Scan
5056
Method
FTMS; HCD
Score
122.19
m/z
646.84
Relative Abundance
Intensity [1e5]
m/z (zoom)
y 175.119
y-NH 158.0924
y 246.1561
y-NH 229.1295
y-NH 316.1615
a 330.2339
b 358.2288
y-HO 412.2303
y-NH 413.2143
y 430.2409
b 521.2921
y-NH 541.2729
y-HO 540.2889
y 558.2994
b 620.3605
y 673.3264
b 735.3875
y 772.3948
b 863.446
y 992.4796
- A G Y V D Q P S A R -
sll0648 probable glycosyltransferase

Raw File	Scan	Method	Score	m/z
HCC_TMT_3_F8_20180514	3004	FTMS; HCD	96.49	552.8

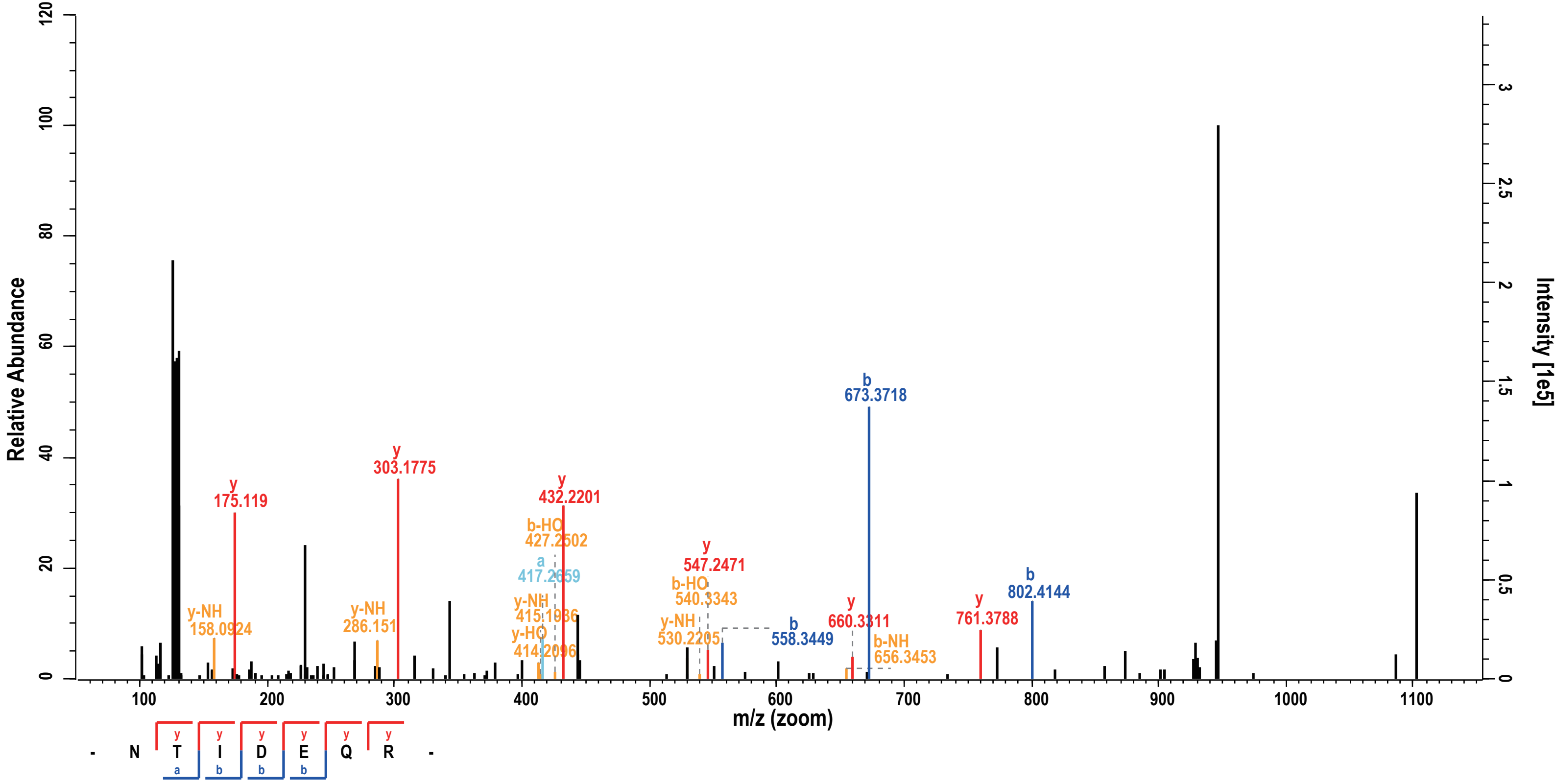

sll0802 hypothetical protein

Raw File	Scan	Method	Score	m/z
HCC_TMT_1_F4_20180514	10606	FTMS; HCD	121.02	818.92

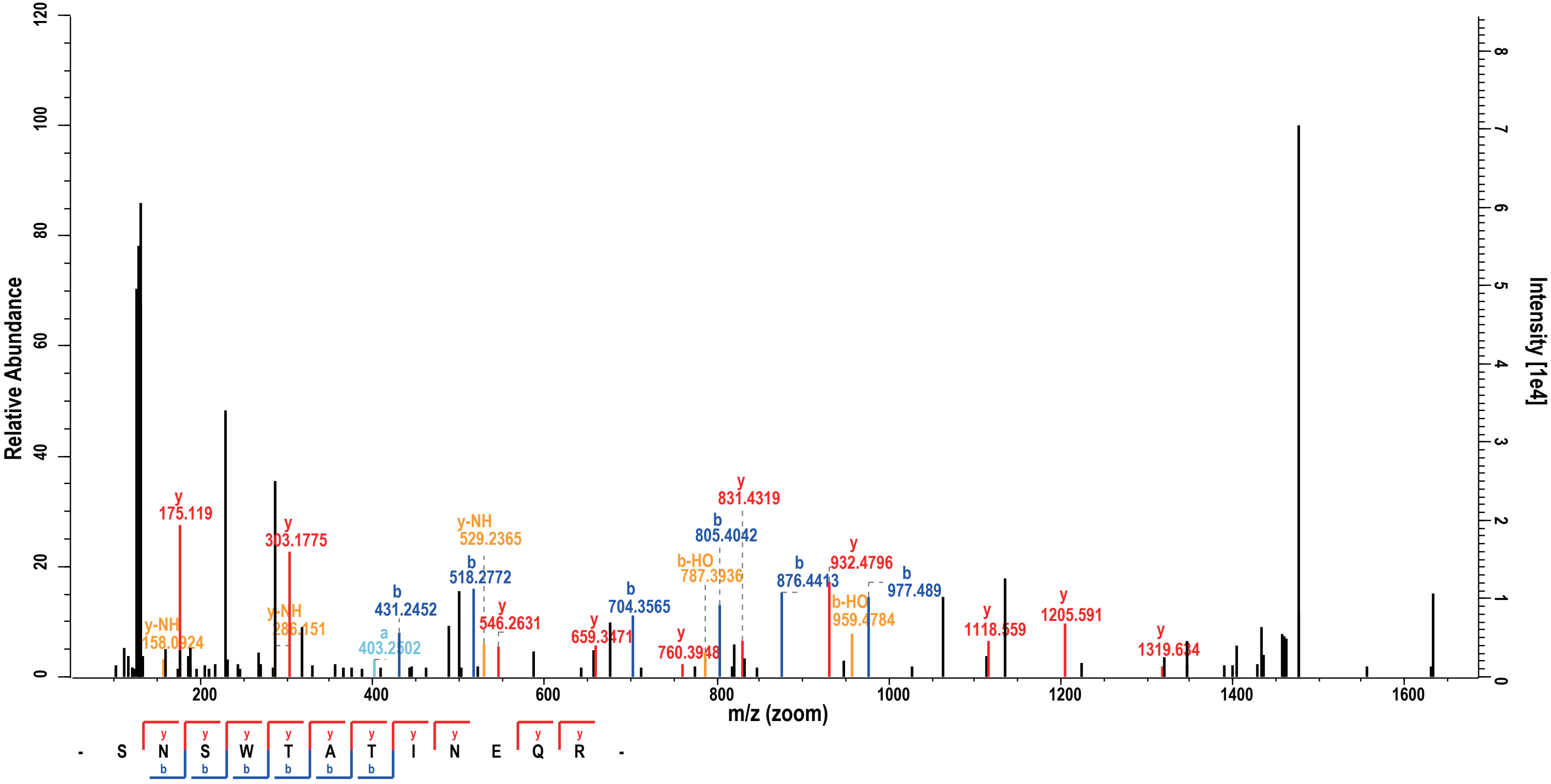

sll0803 hypothetical protein

Raw File	Scan	Method	Score	m/z
HCC_TMT_2_F6_20180514	12136	FTMS; HCD	149.96	694.39

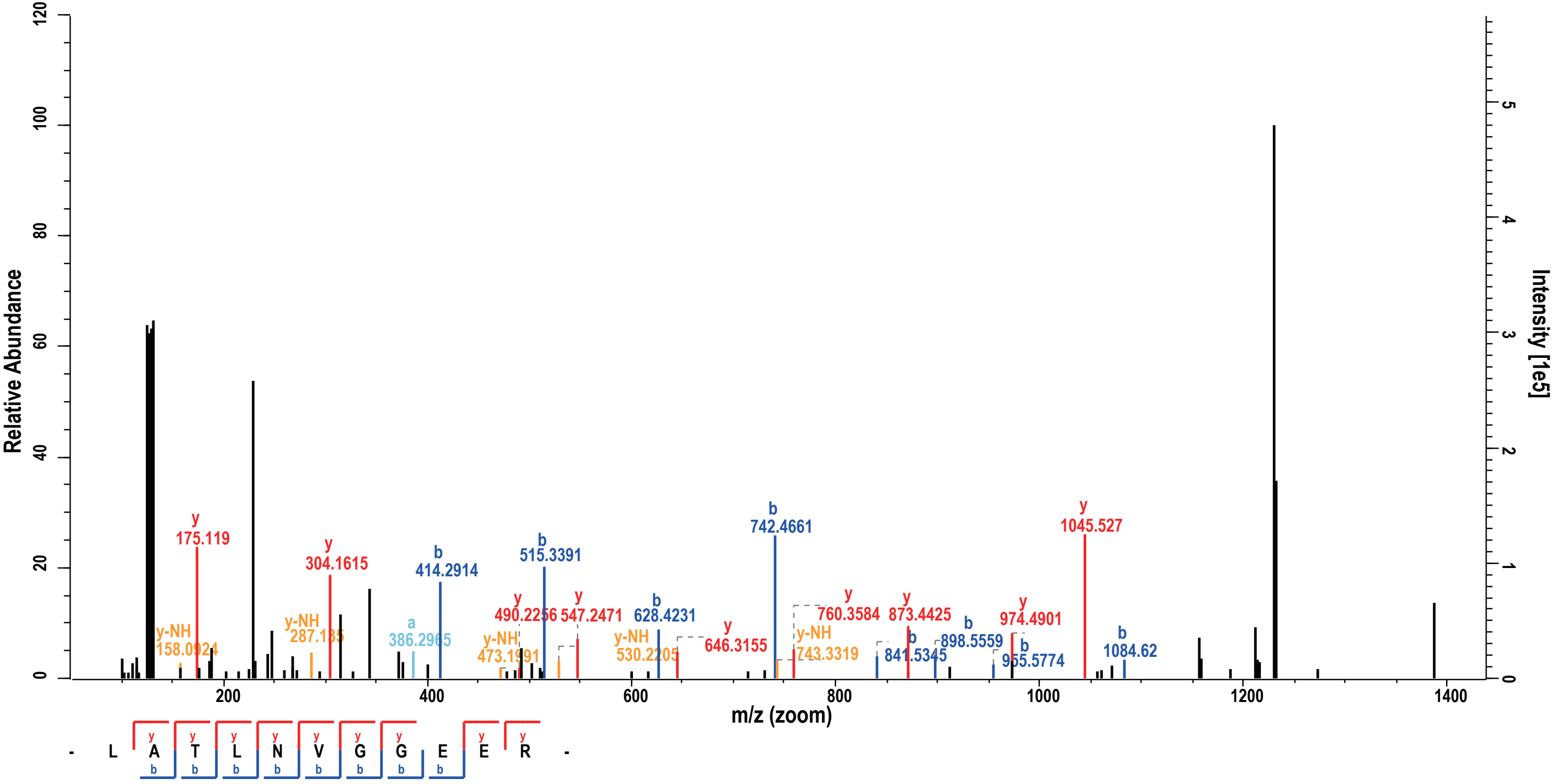

sll0829 probable methyltransferase

Raw File	Scan	Method	Score	m/z
HCC_TMT_3_F14_20180514	11534	FTMS; HCD	110.12	584.31

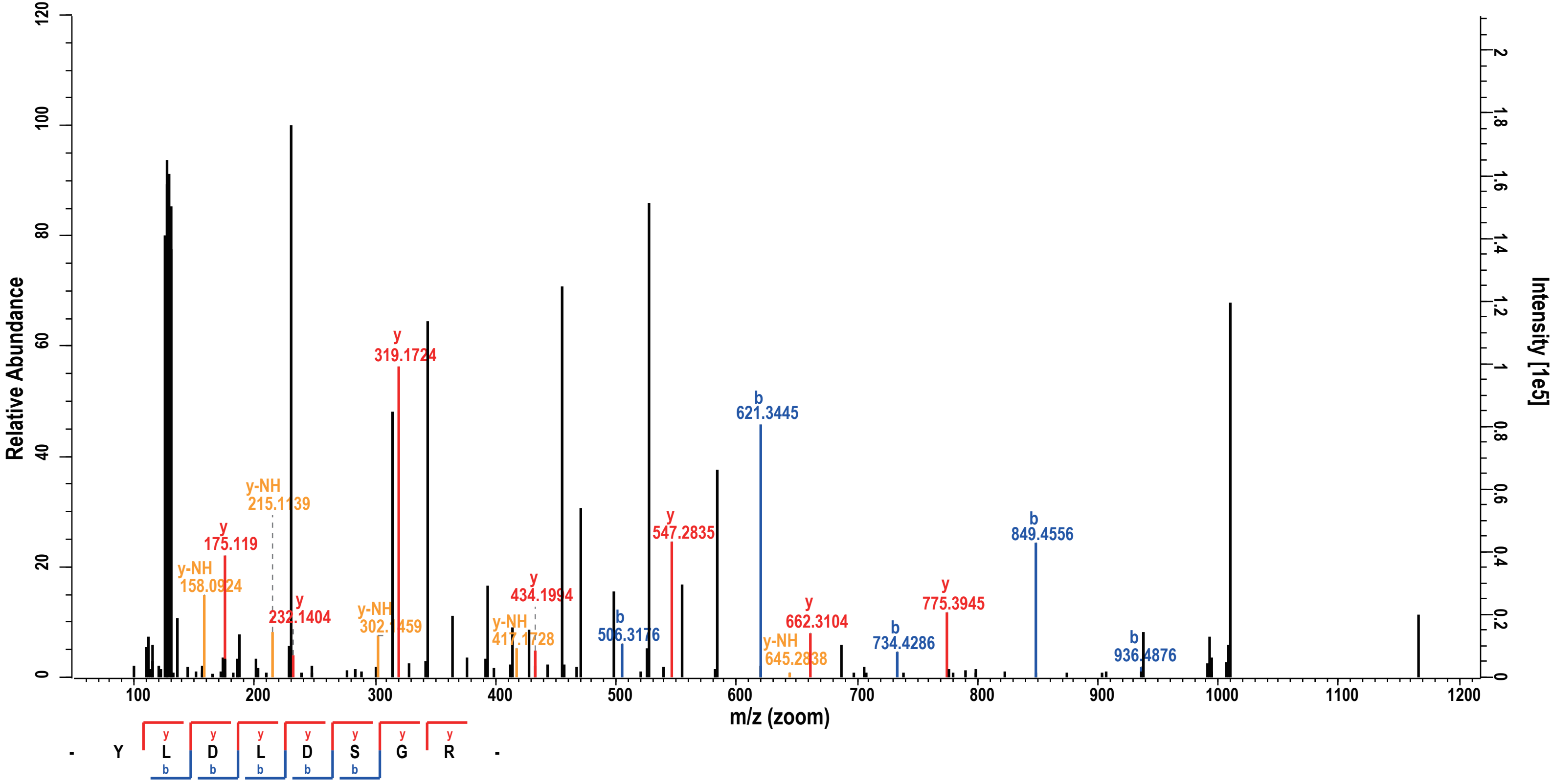

sll0832 hypothetical protein

Raw File	Scan	Method	Score	m/z
HCC_TMT_2_F12_20180514	6276	FTMS; HCD	78.52	673.91

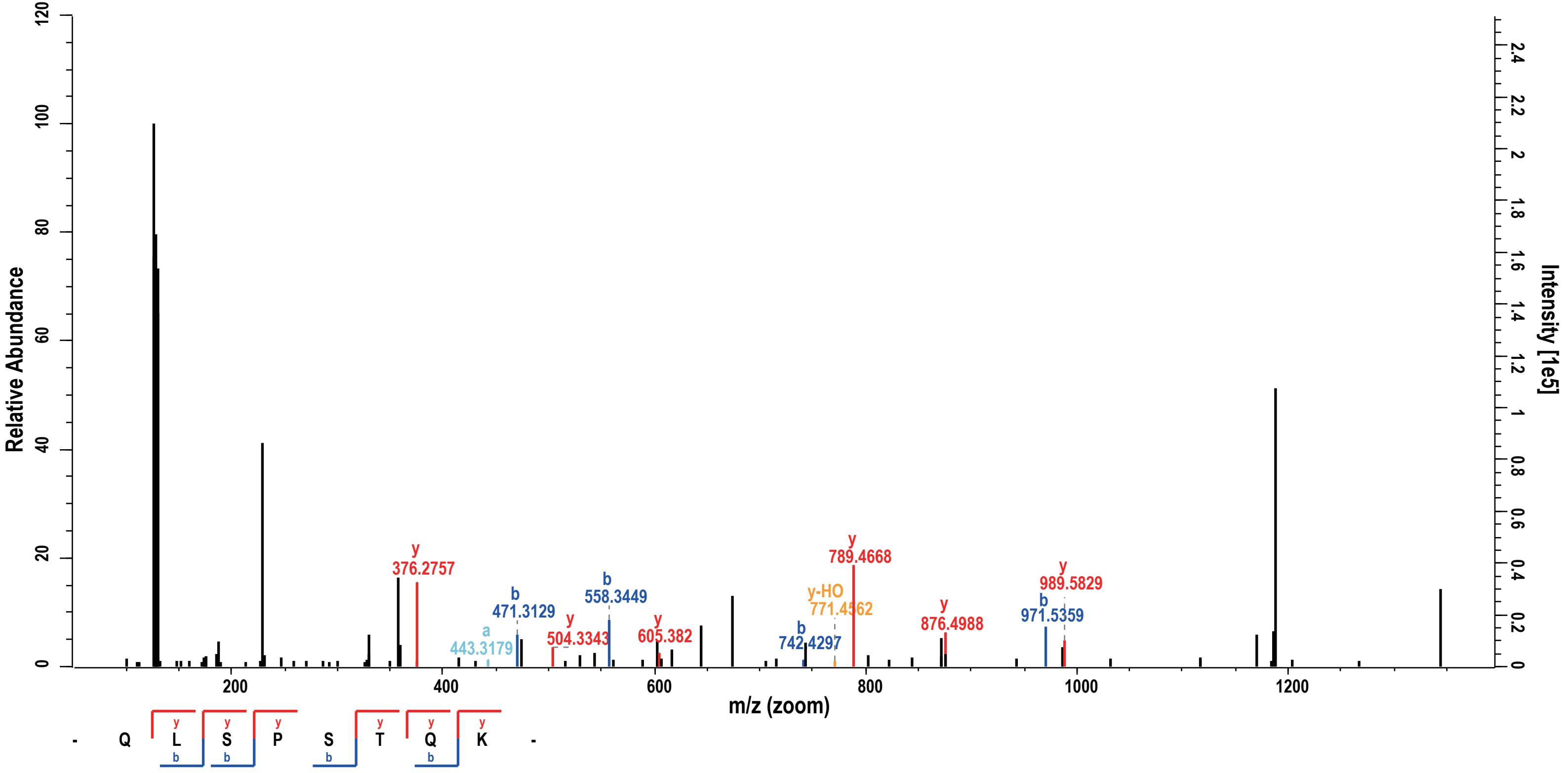

sll1154 putative antibiotic efflux protein

Raw File	Scan	Method	Score	m/z
HCC_TMT_2_F8_20180514	6013	FTMS; HCD	68.89	678.88

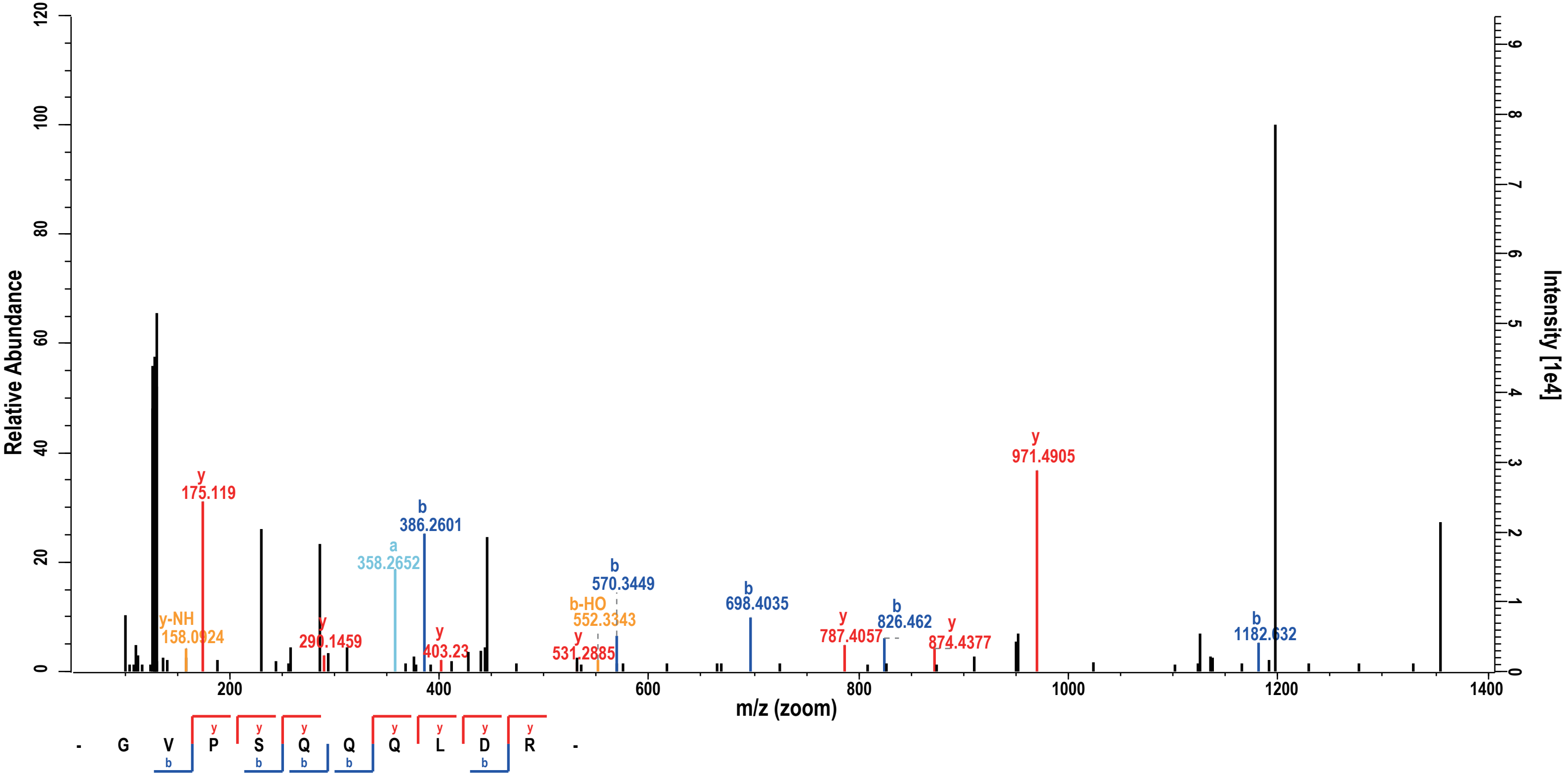

sll1191 hypothetical protein

Raw File	Scan	Method	Score	m/z
HCC_TMT_3_F10_20180514	15929	FTMS; HCD	117.02	662.4

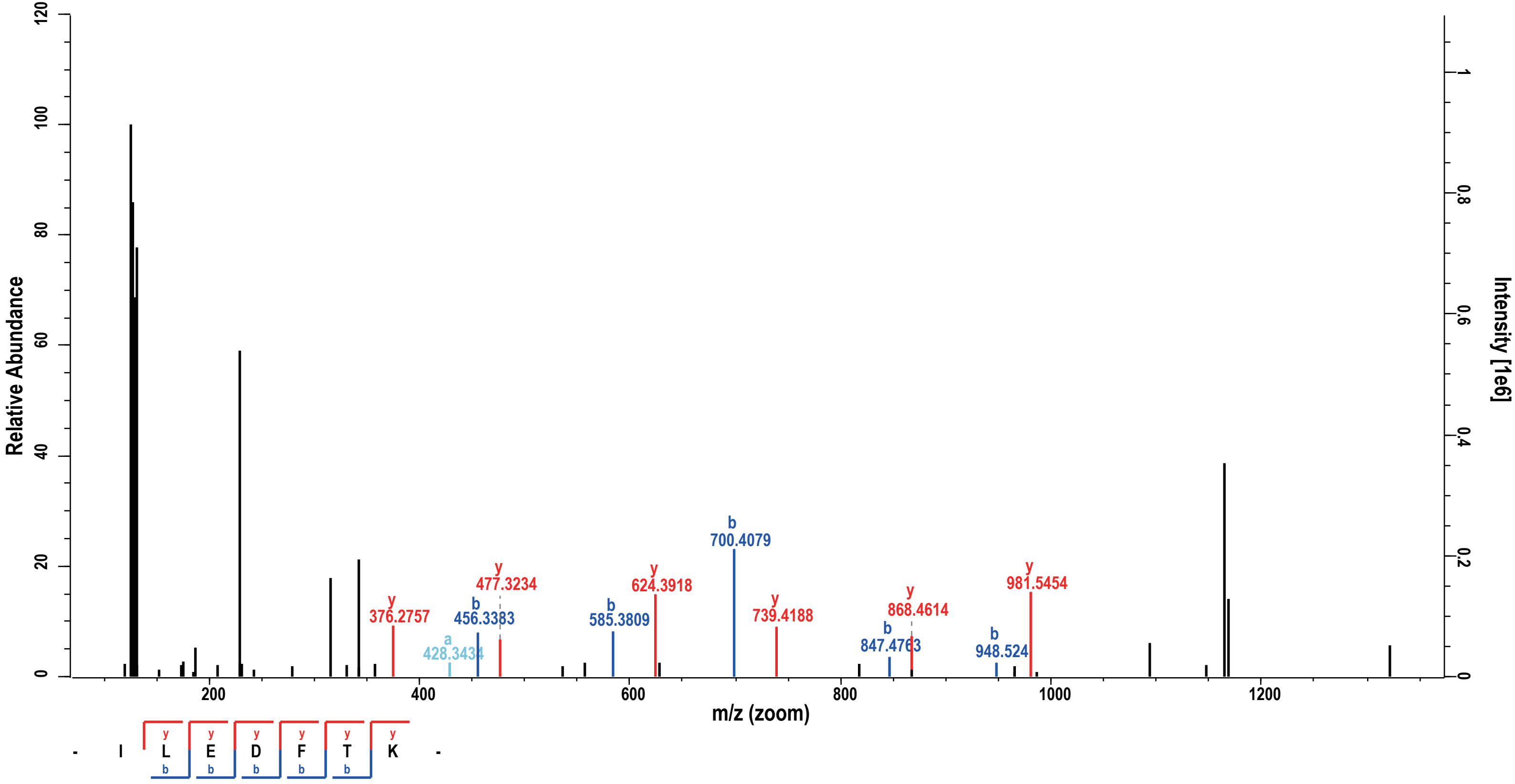

sll1322 ATP synthase A chain of CF(0)

Raw File	Scan	Method	Score	m/z
HCC_TMT_2_F15_20180514	11827	FTMS; HCD	197.3	971.05

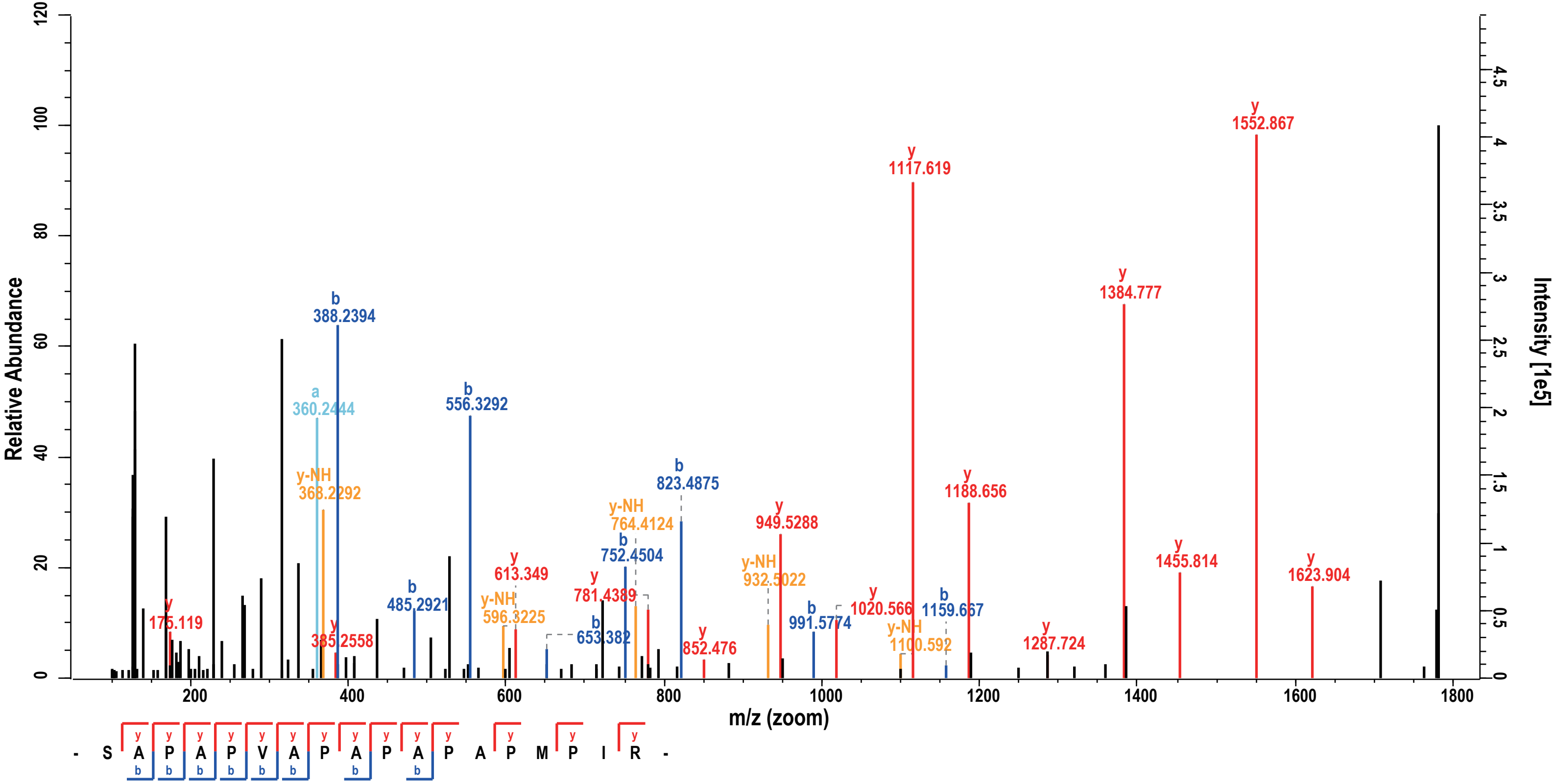

sll1338 unknown protein

Raw File	Scan	Method	Score	m/z
HCC_TMT_1_F7_20180514	8121	FTMS; HCD	126.67	595.38

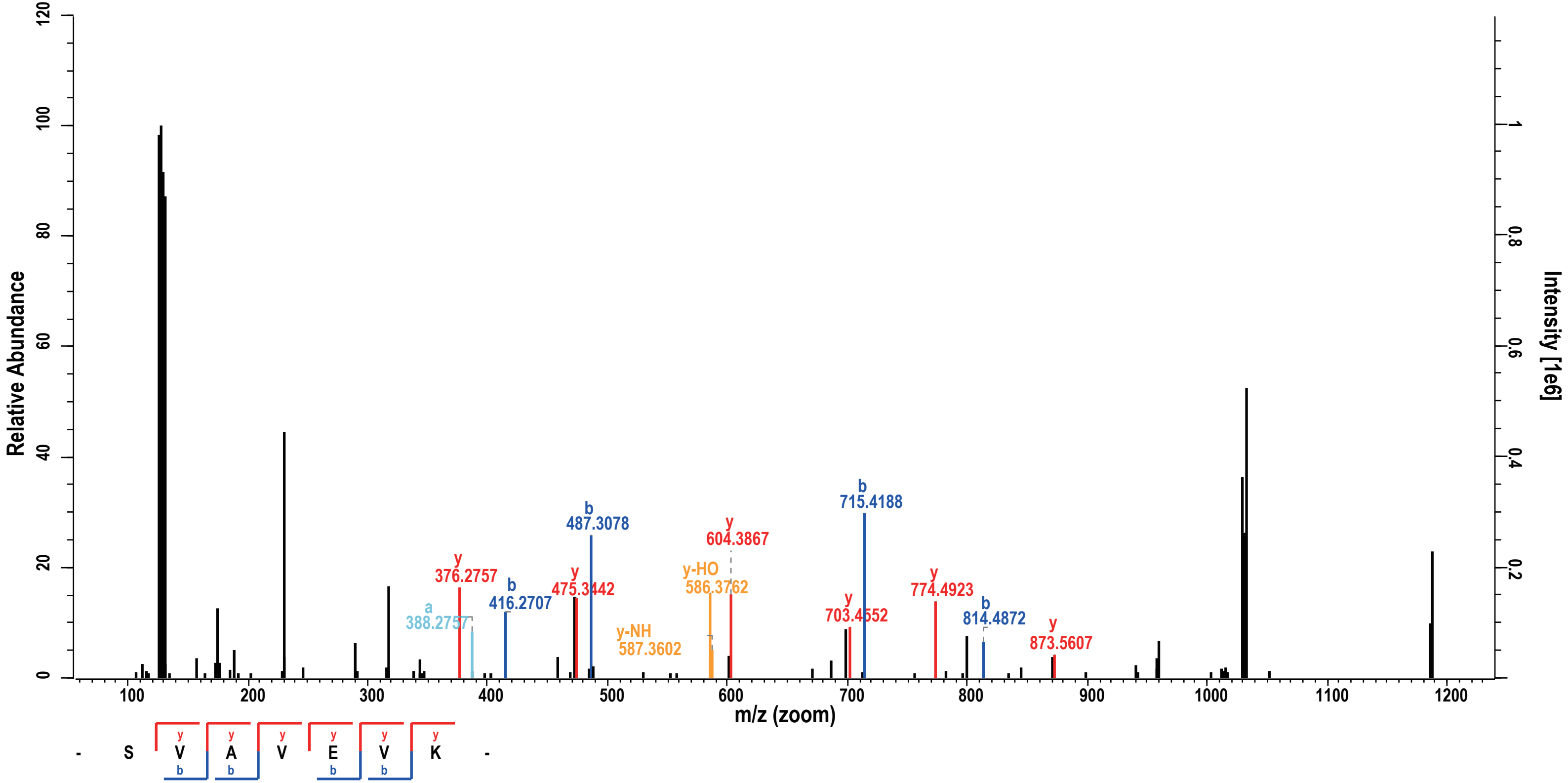

sll1426 unknown protein

Raw File	Scan	Method	Score	m/z
HCC_TMT_2_F5_20180514	10692	FTMS; HCD	117.56	544.82

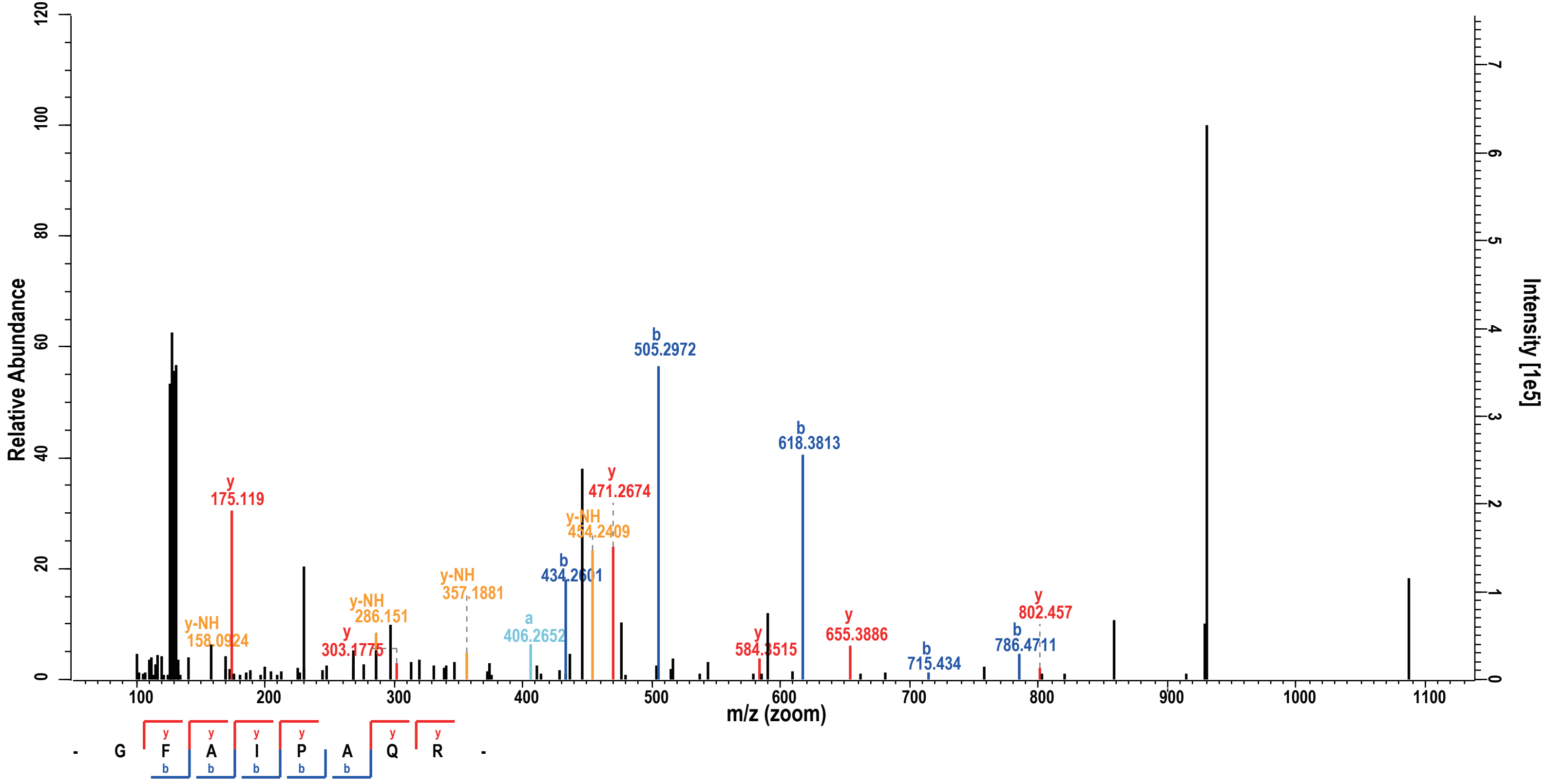

sll1486 hypothetical protein

Raw File	Scan	Method	Score	m/z
HCC_TMT_3_F5_20180514	8144	FTMS; HCD	168.83	705.41

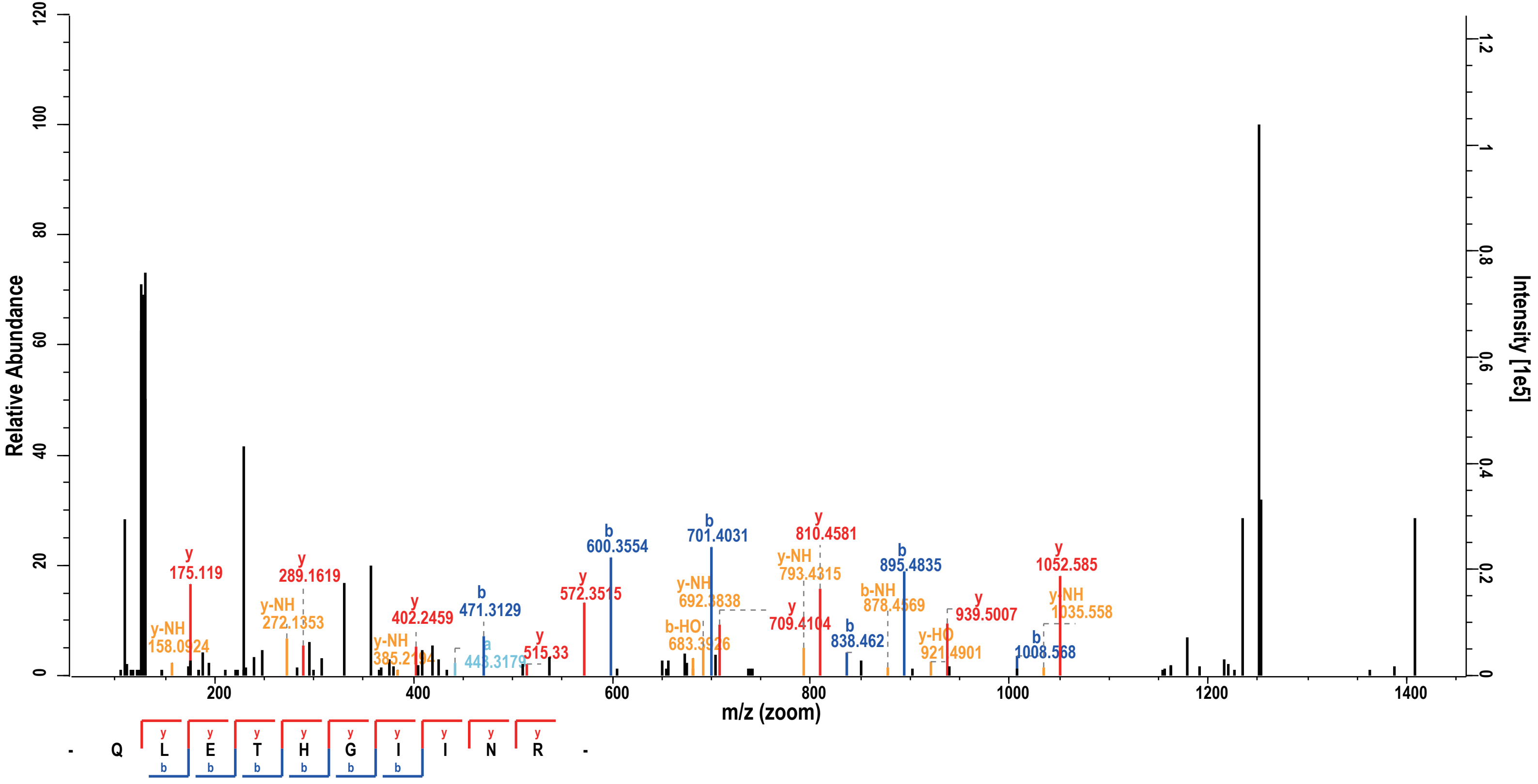

sll1512 hypothetical protein

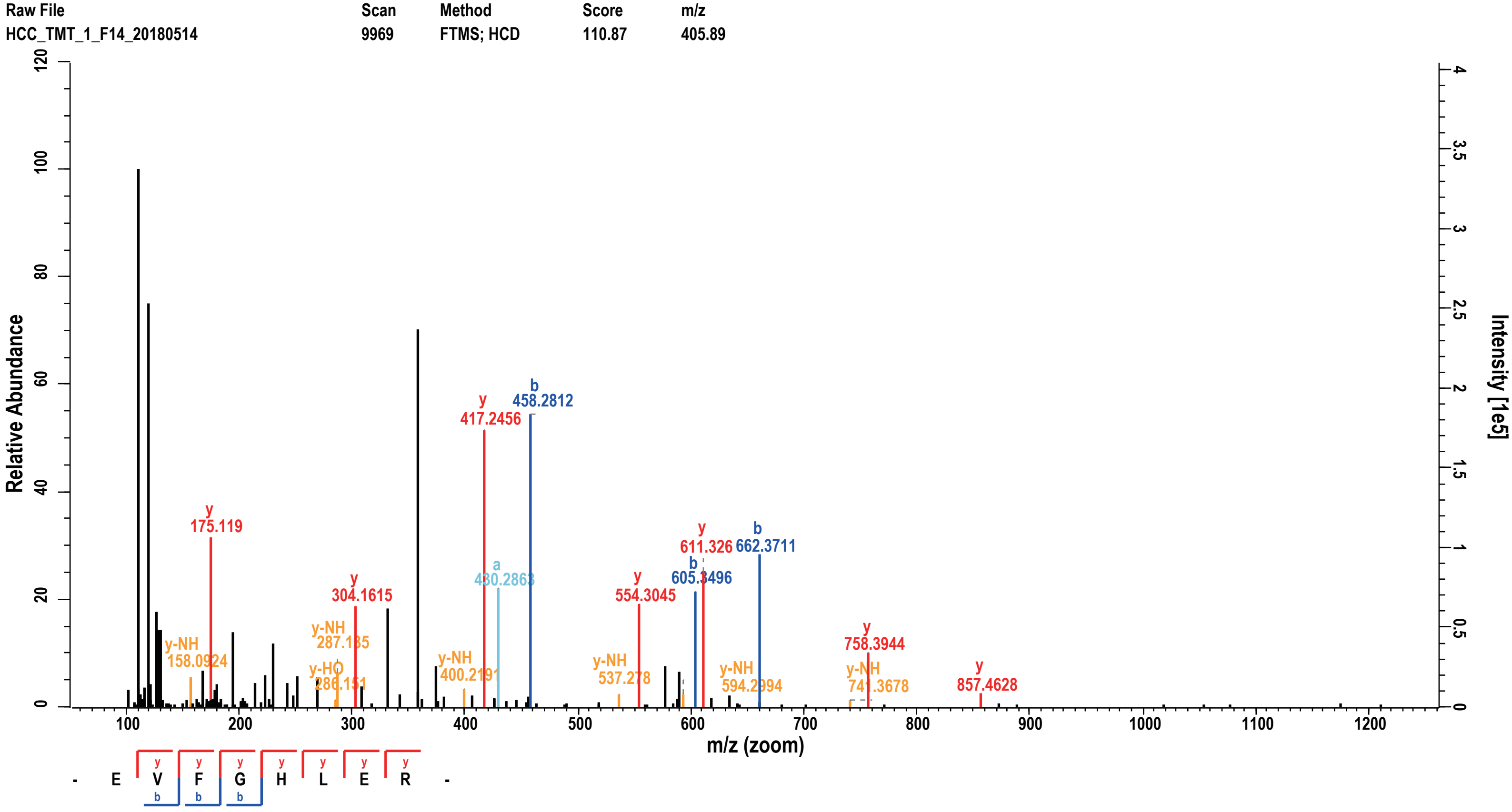

sll1638 hypothetical protein

Raw File	Scan	Method	Score	m/z
HCC_TMT_2_F15_20180514	13757	FTMS; HCD	98.05	770.4

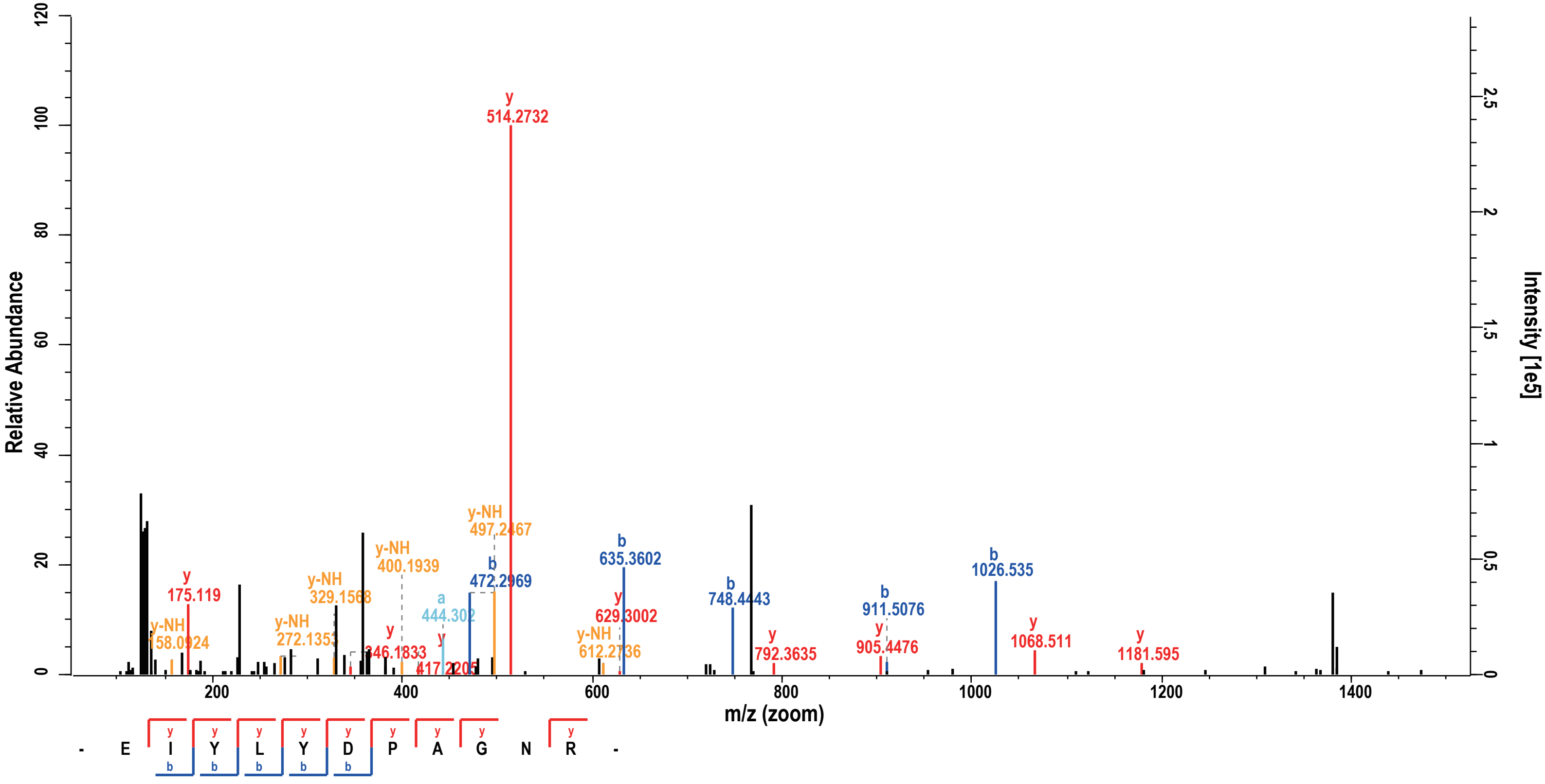

sll1658 hypothetical protein

Raw File	Scan	Method	Score	m/z
HCC_TMT_3_F11_20180514	15019	FTMS; HCD	78.69	625.34

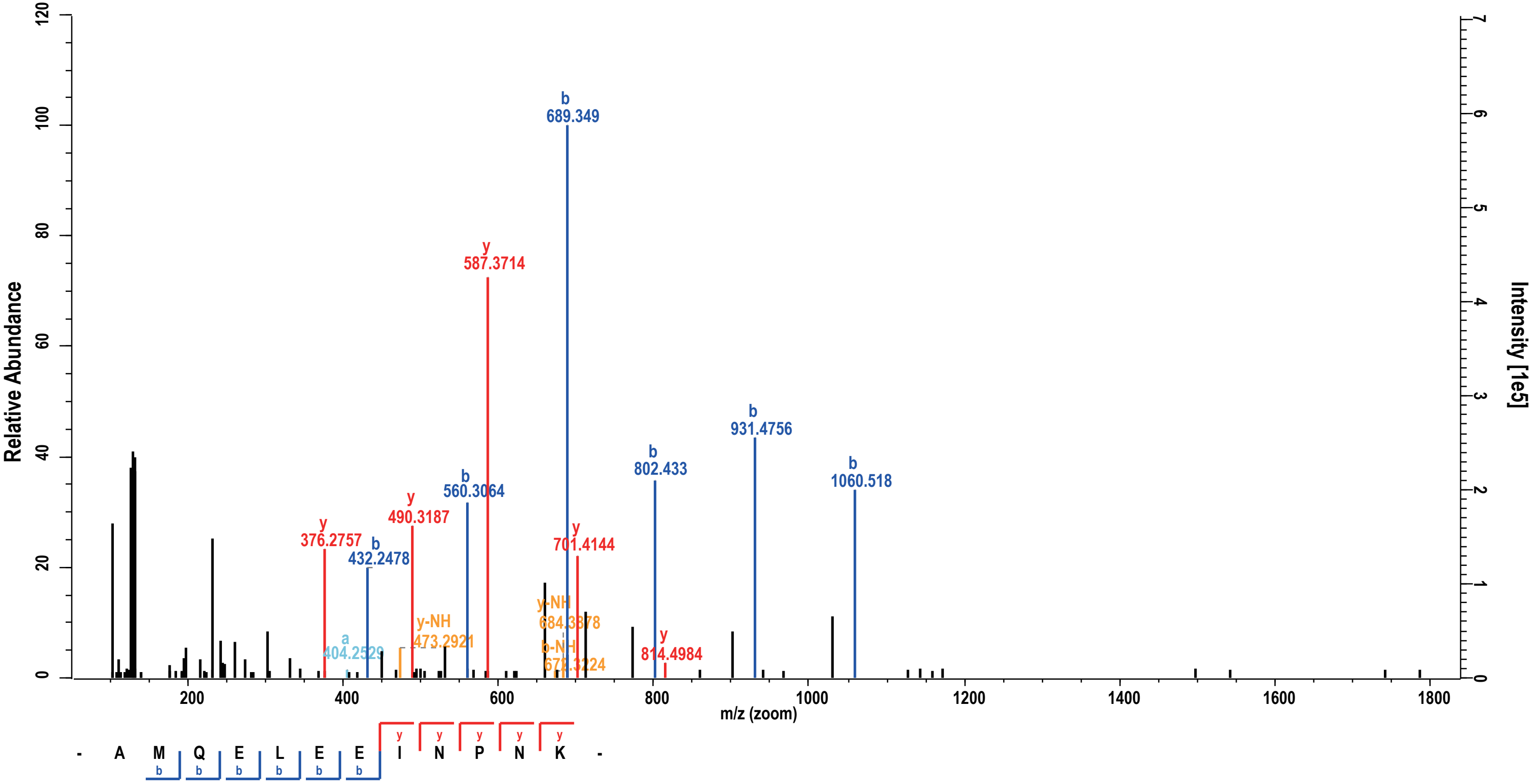

sll1677 similar to spore maturation protein B

Raw File	Scan	Method	Score	m/z
HCC_TMT_3_F13_20180514	8498	FTMS; HCD	116.3	708.43

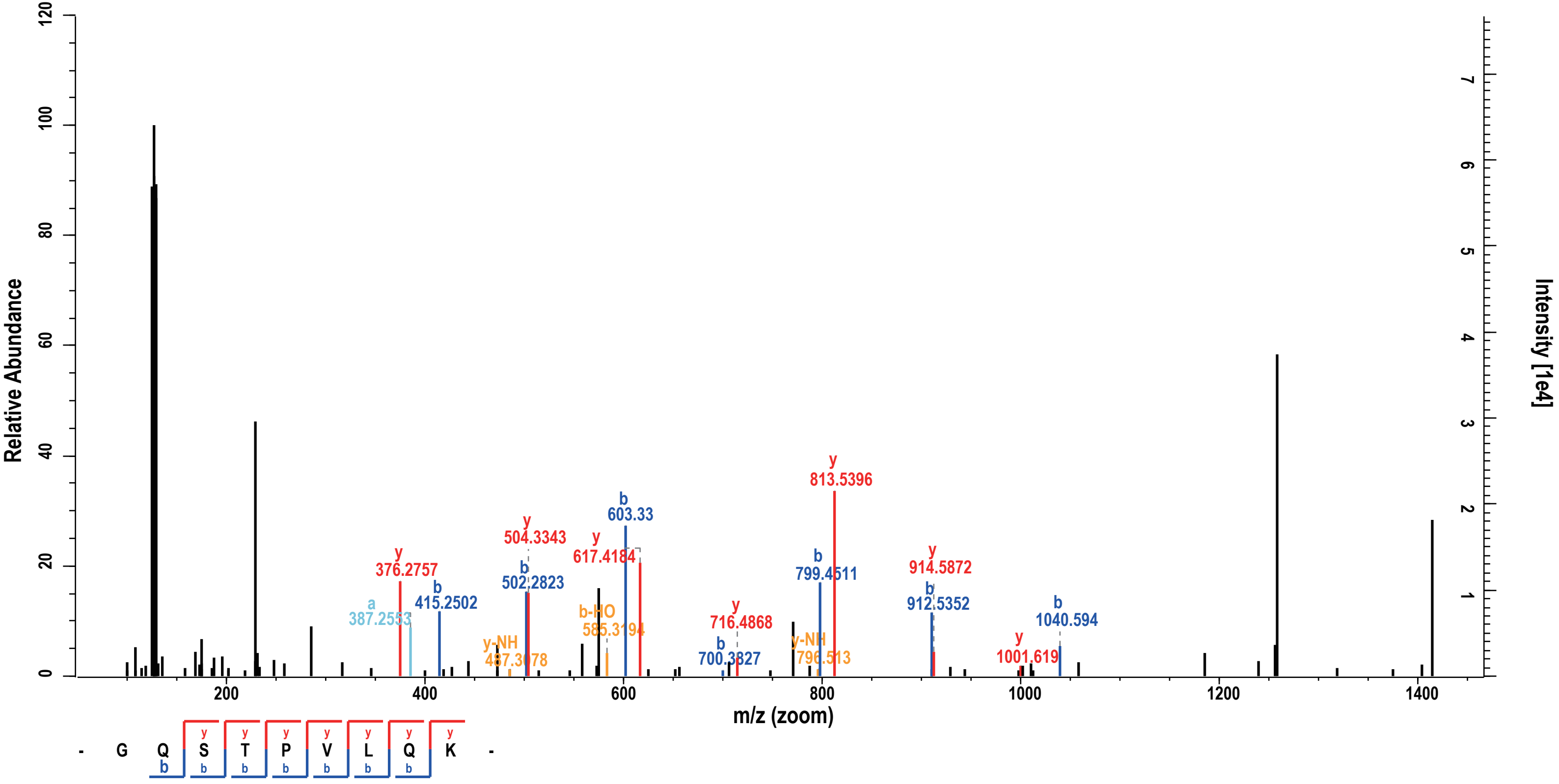

sll1878 iron(III)-transport ATP-binding protein

Raw File	Scan	Method	Score	m/z
HCC_TMT_1_F13_20180514	8417	FTMS; HCD	180.07	713.4

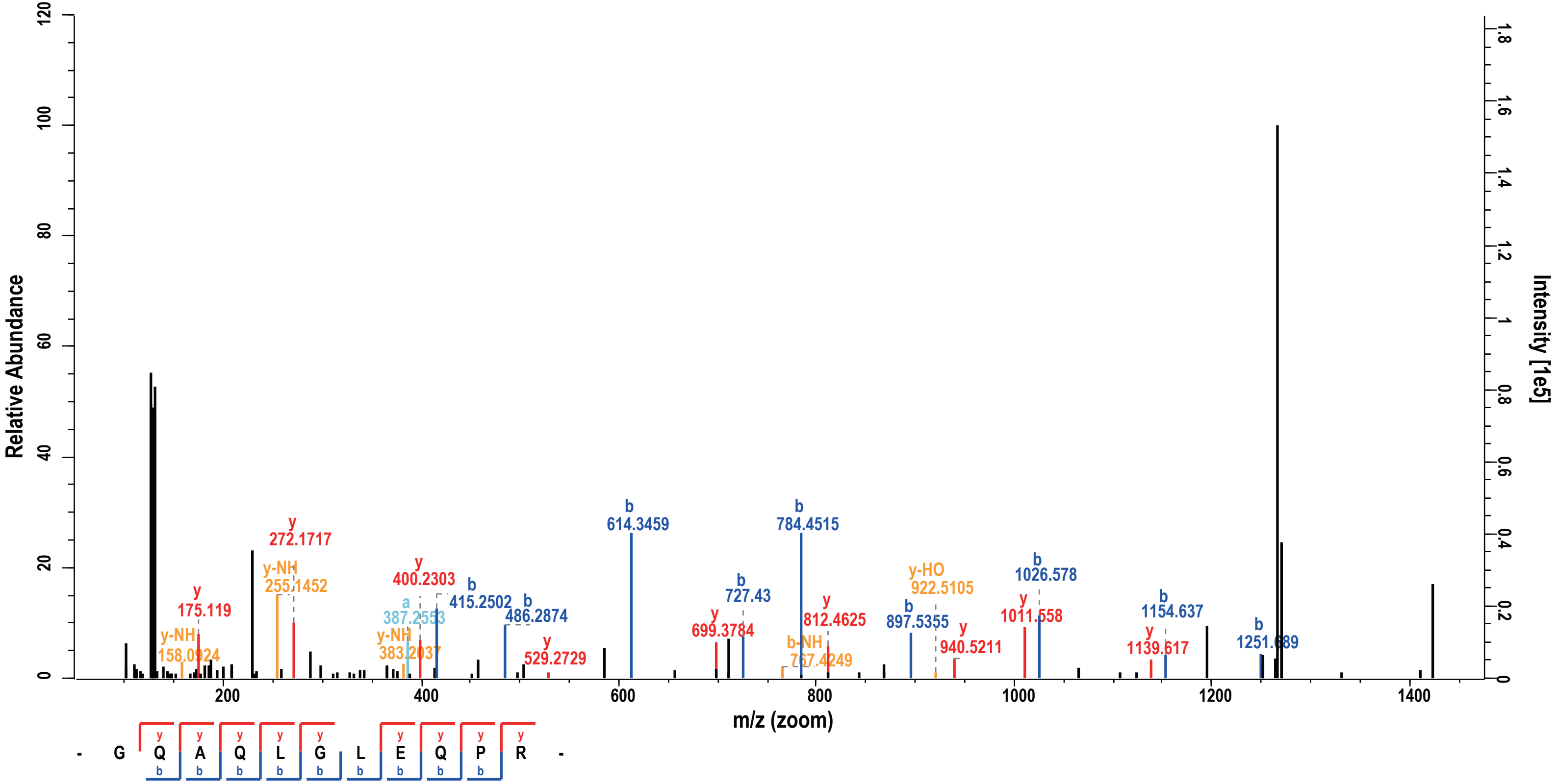

sll1882 unknown protein

Raw File	Scan	Method	Score	m/z
HCC_TMT_2_F12_20180514	8634	FTMS; HCD	122.52	685.88

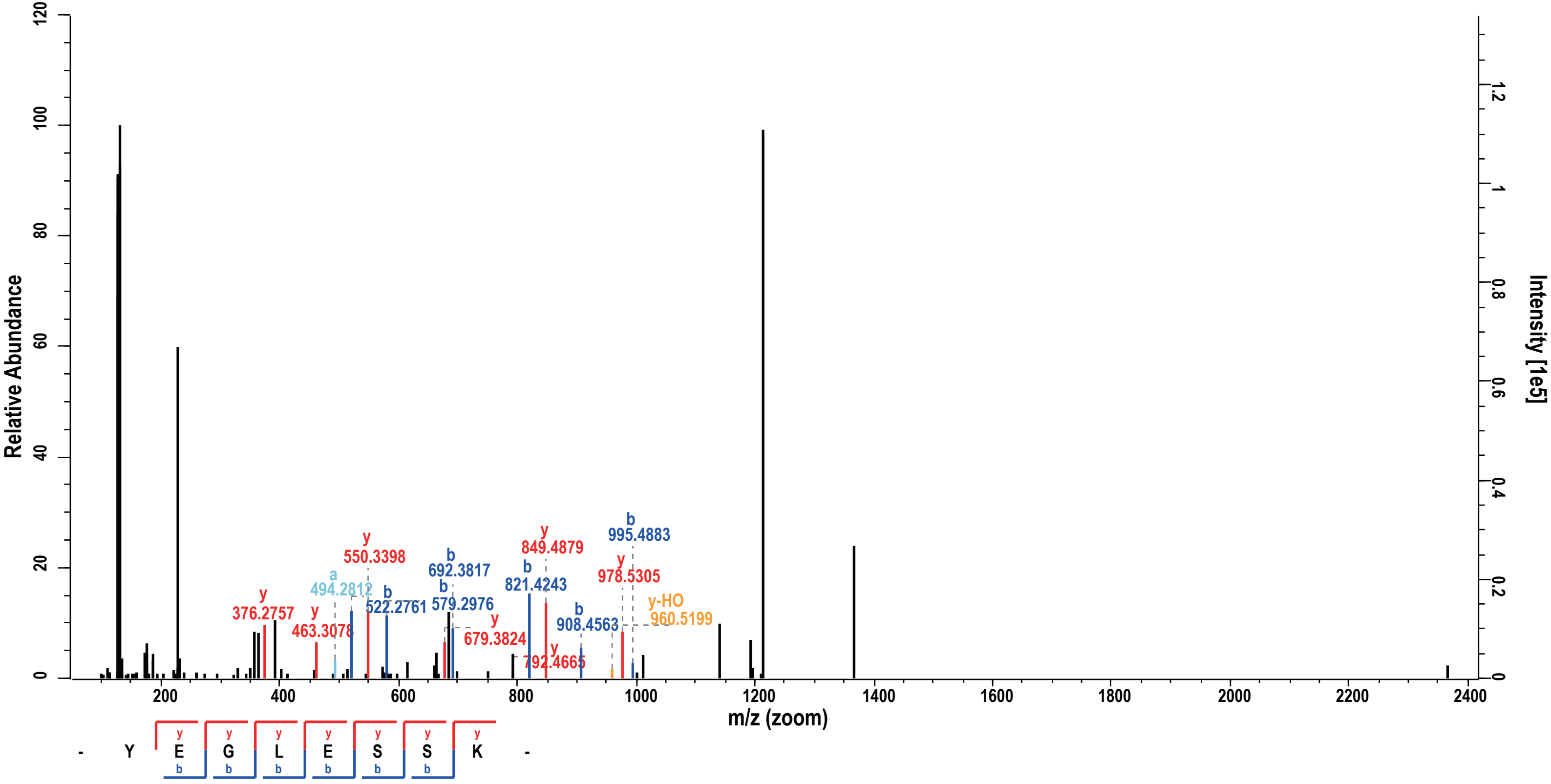

sll1942 unknown protein

Raw File	Scan	Method	Score	m/z
HCC_TMT_3_F12_20180514	5394	FTMS; HCD	96.25	623.88

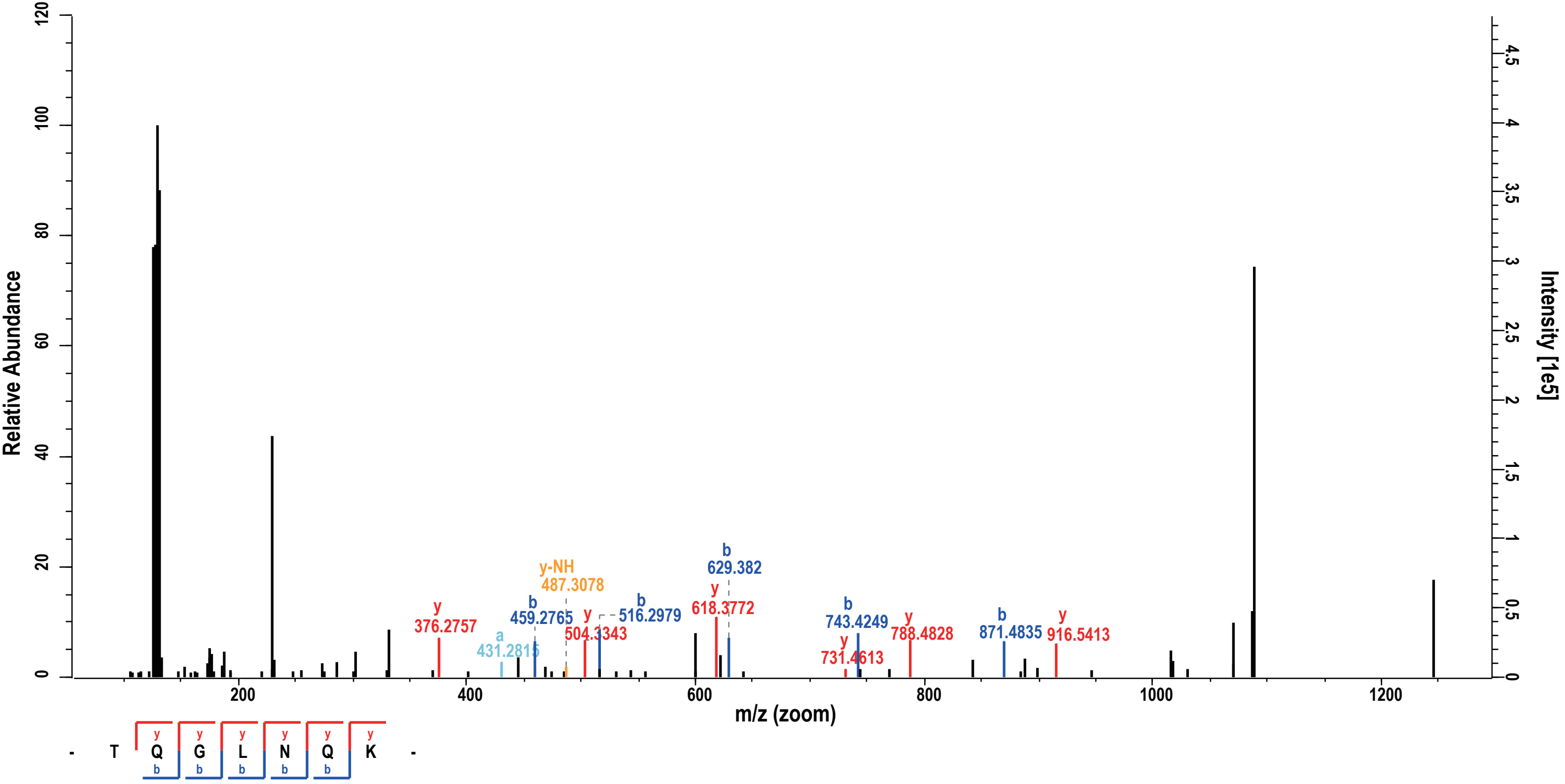

sll2006 hypothetical protein

Raw File	Scan	Method	Score	m/z
HCC_TMT_2_F11_20180514	10012	FTMS; HCD	141.39	704.91

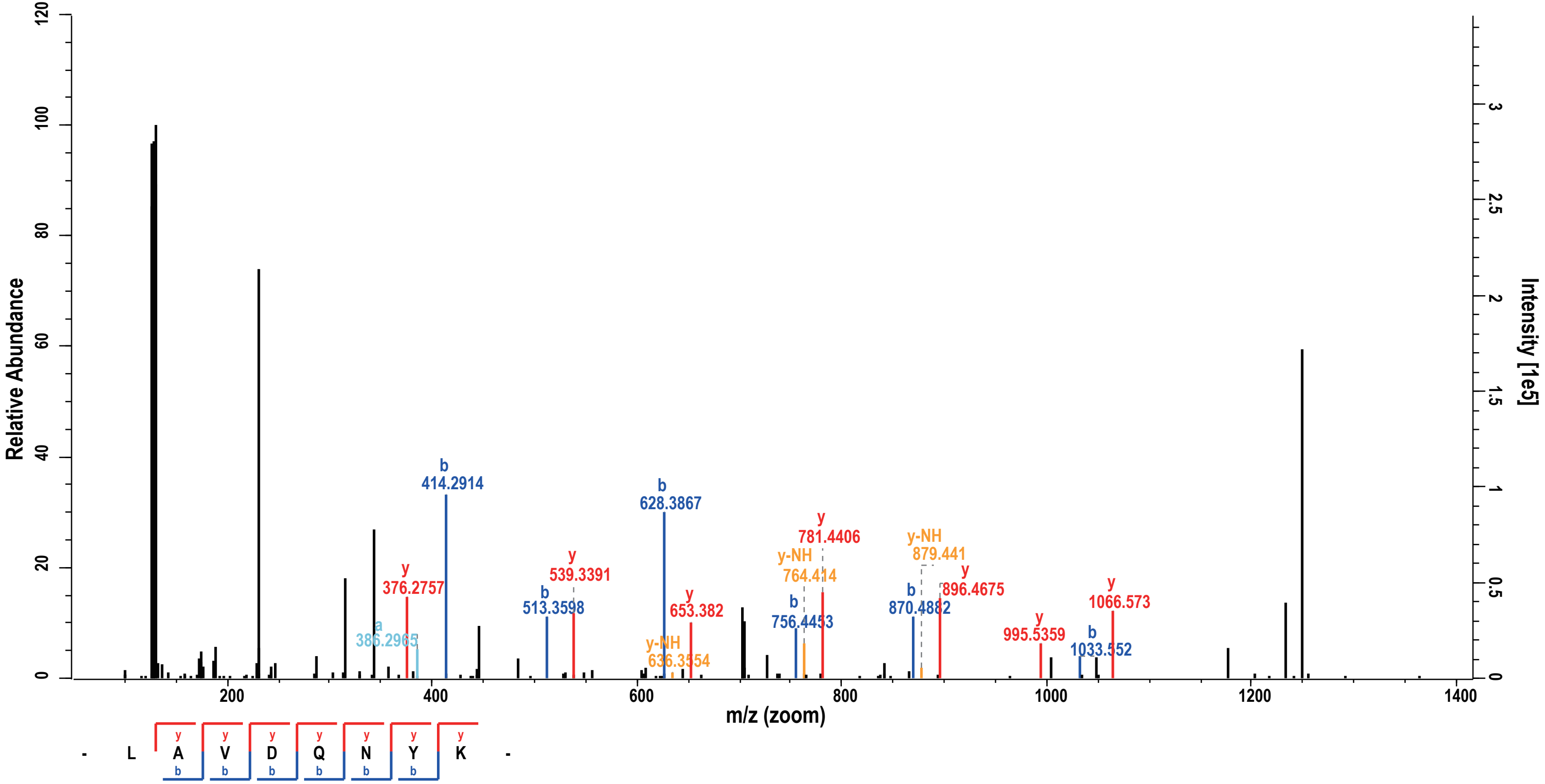

sll7030 hypothetical protein

Raw File	Scan	Method	Score	m/z
HCC_TMT_2_F13_20180514	14937	FTMS; HCD	132.08	589.35

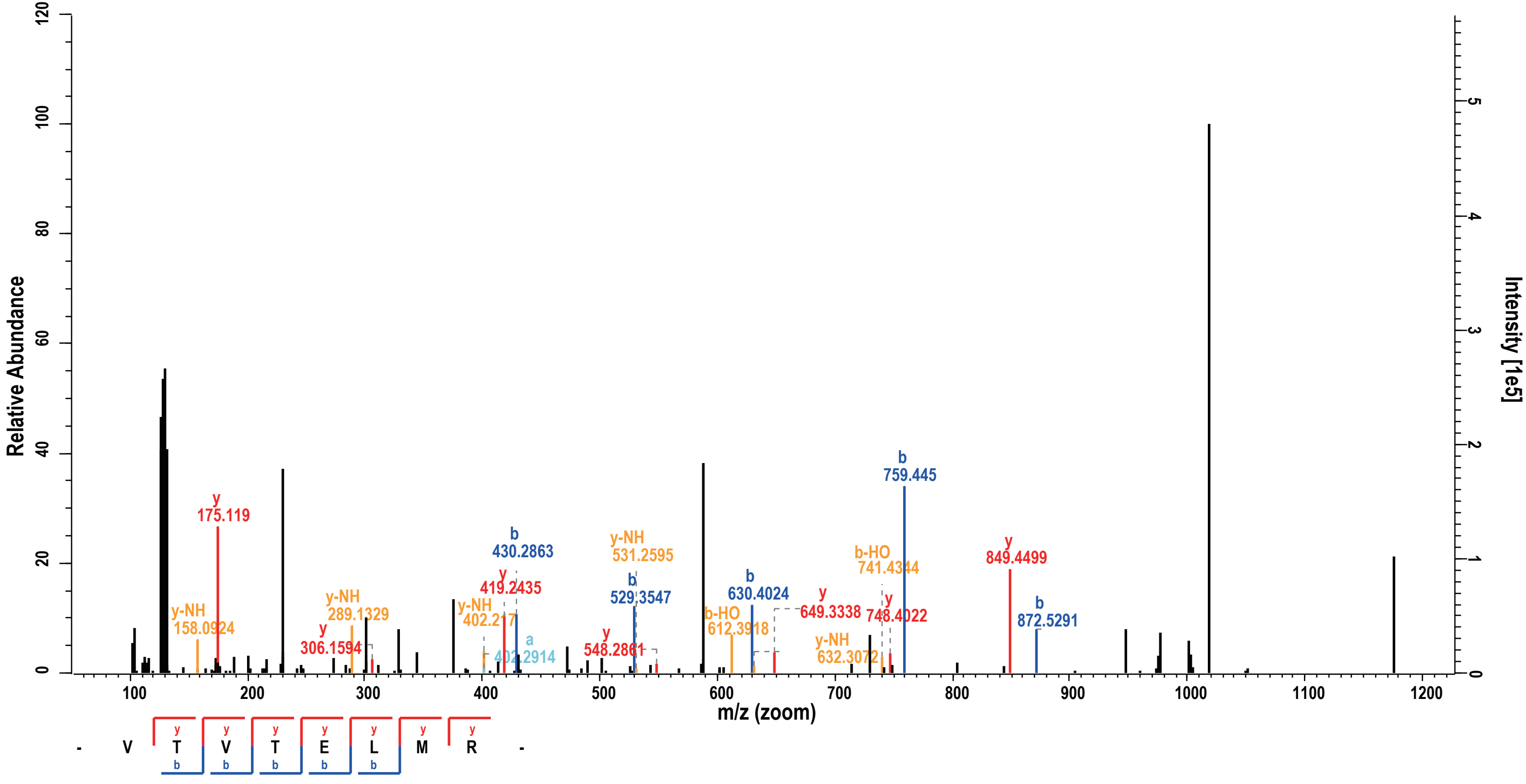

sll8027 hypothetical protein

Raw File	Scan	Method	Score	m/z
HCC_TMT_3_F3_20180514	7873	FTMS; HCD	81.64	468.95

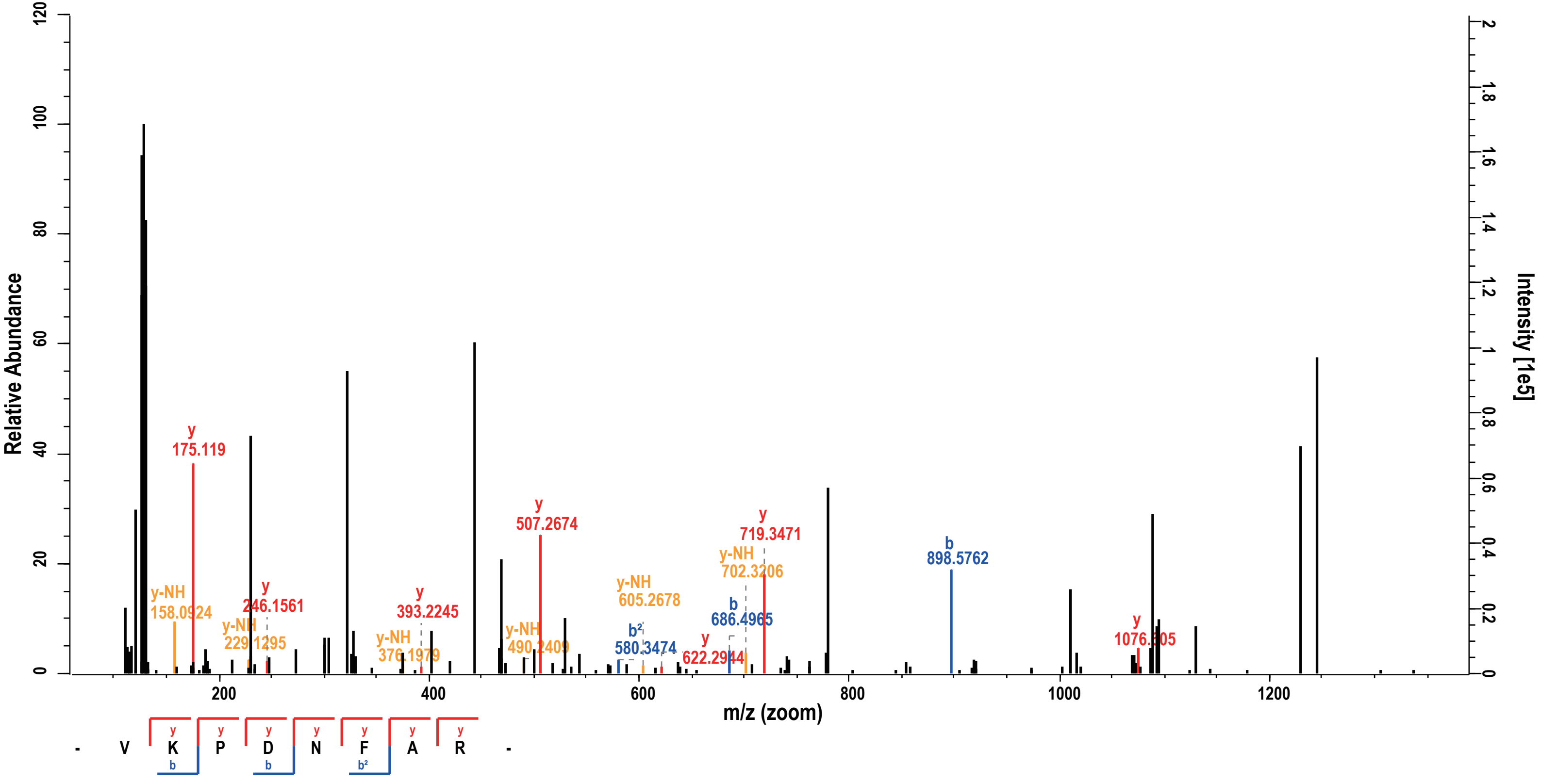

slr0022 hypothetical protein

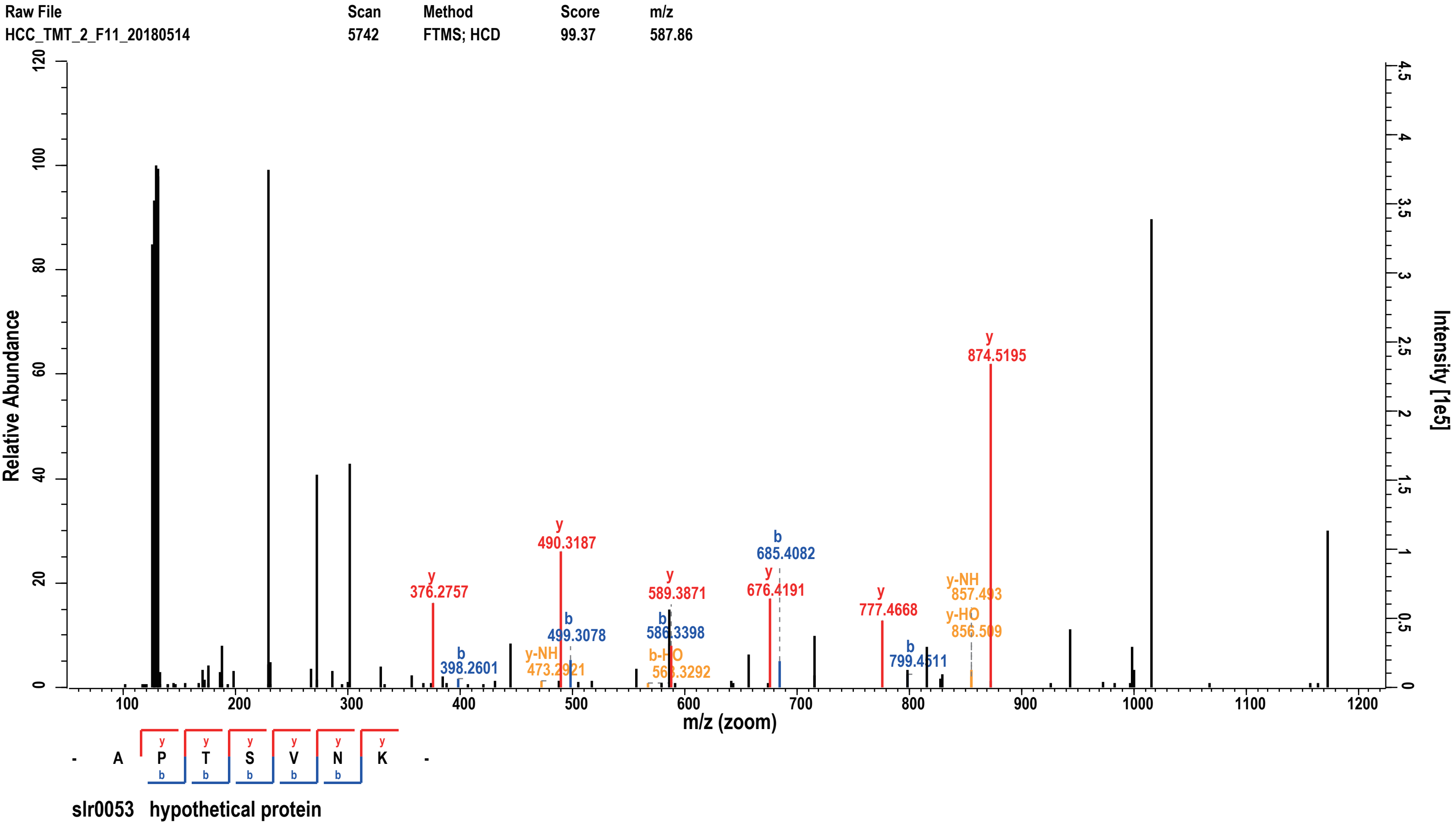

Raw File
HCC_TMT_2_F11_20180514
Scan
5742
Method
FTMS; HCD
Score
99.37
m/z
587.86
Relative Abundance
Intensity [1e5]
m/z (zoom)
y 376.2757
b 398.2601
y-NH 473.2921
y 490.3187
b 499.3078
b-HO 568.3292
b 586.3398
y 589.3871
y 676.4191
b 685.4082
y 777.4668
b 799.4511
y-HO 856.509
y-NH 857.493
y 874.5195
- A P T S V N K -
slr0053 hypothetical protein

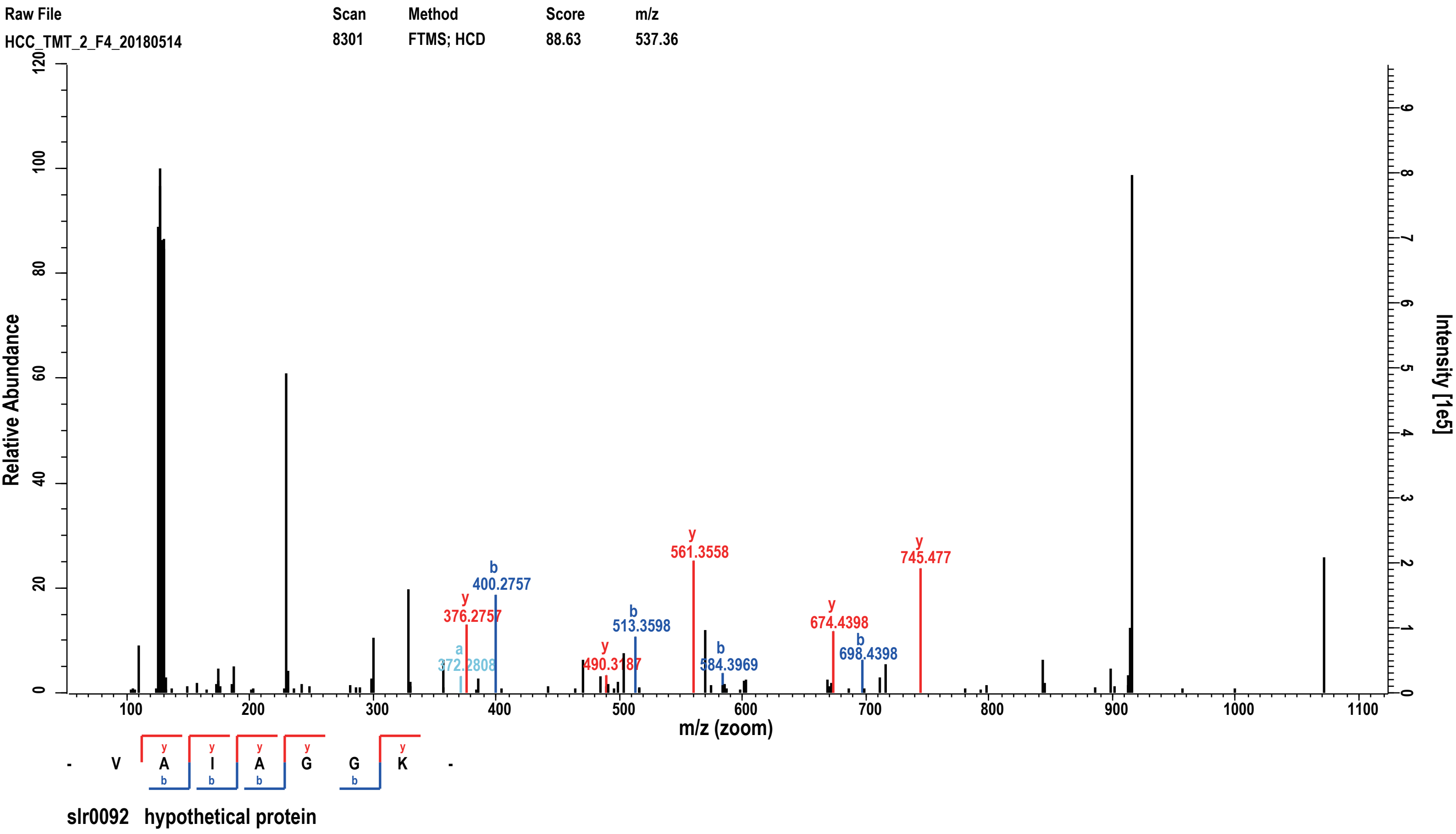
Raw File
HCC_TMT_2_F4_20180514
Scan
8301
Method
FTMS; HCD
Score
88.63
m/z
537.36
Relative Abundance
Intensity [1e5]
m/z (zoom)
a 372.2808
y 376.2757
b 400.2757
y 490.3187
b 513.3598
y 561.3558
b 584.3969
y 674.4398
b 698.4398
y 745.477
- V A I A G G K -
slr0092 hypothetical protein

Raw File	Scan	Method	Score	m/z
HCC_TMT_3_F5_20180514	4017	FTMS; HCD	105.25	538.28

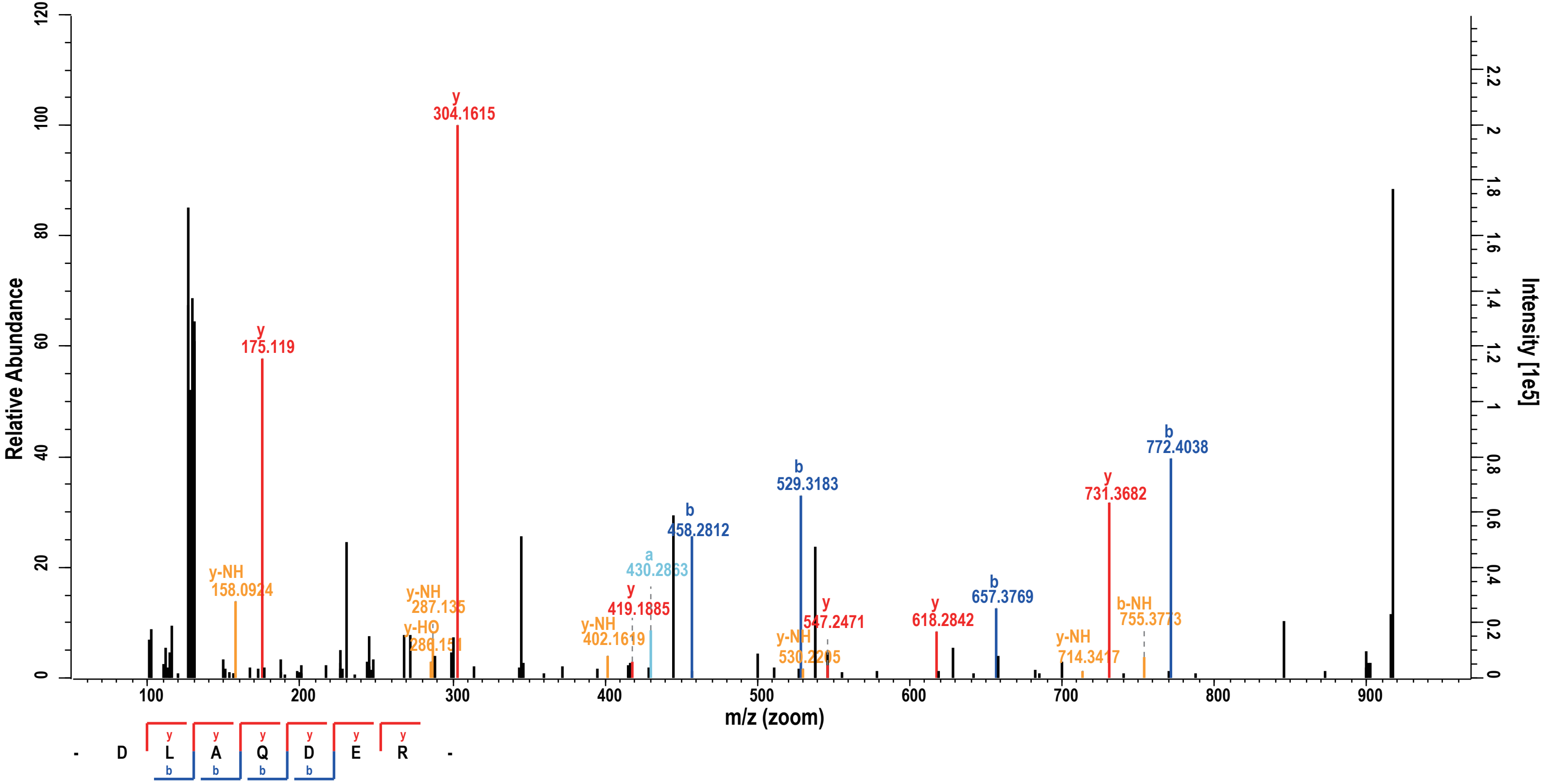

slr0095 O-methyltransferase

Raw File	Scan	Method	Score	m/z
HCC_TMT_1_F6_20180514	8614	FTMS; HCD	166.14	804.95

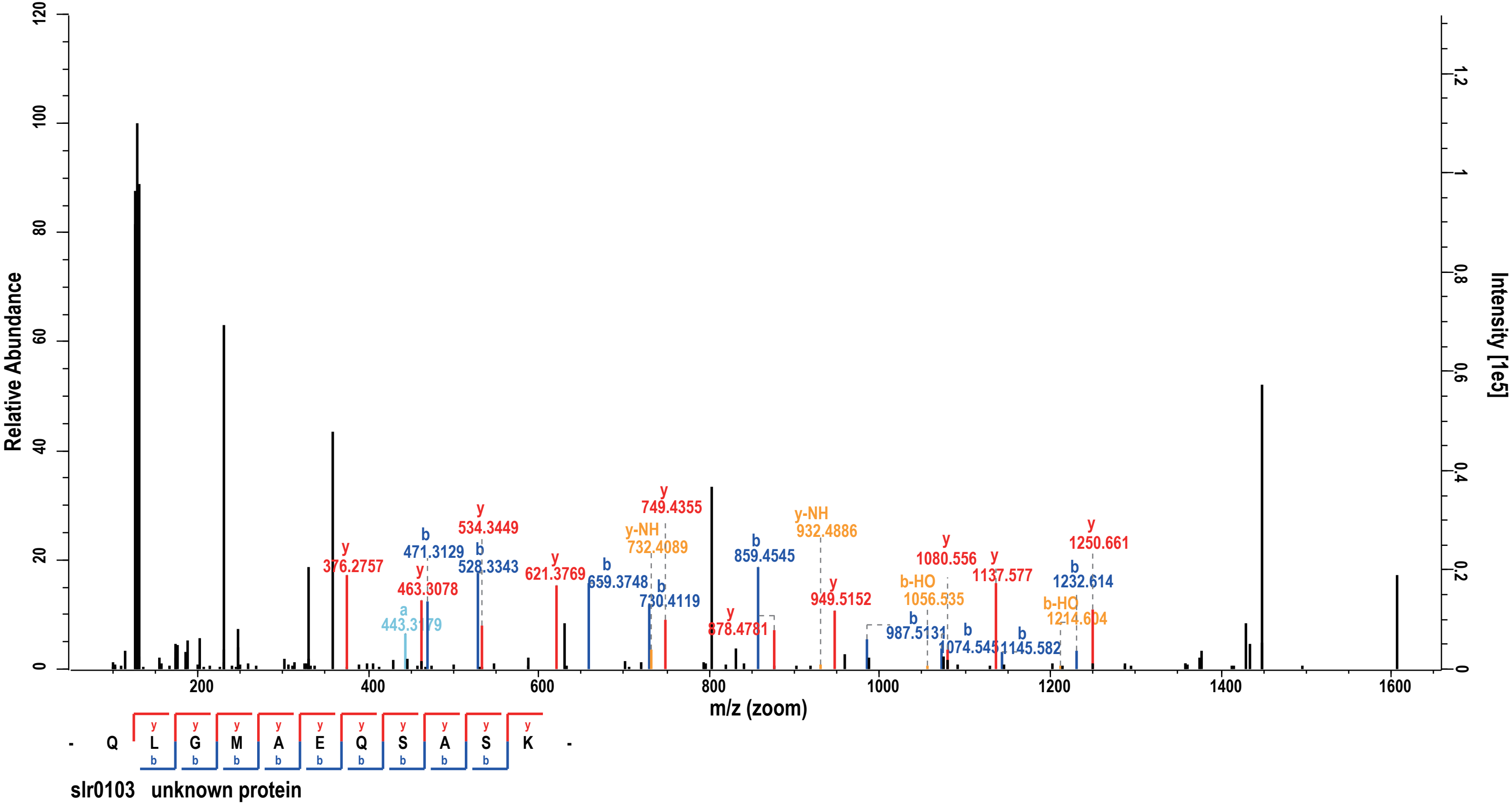

Raw File	Scan	Method	Score	m/z
HCC_TMT_3_F4_20180514	6165	FTMS; HCD	83.73	677.39

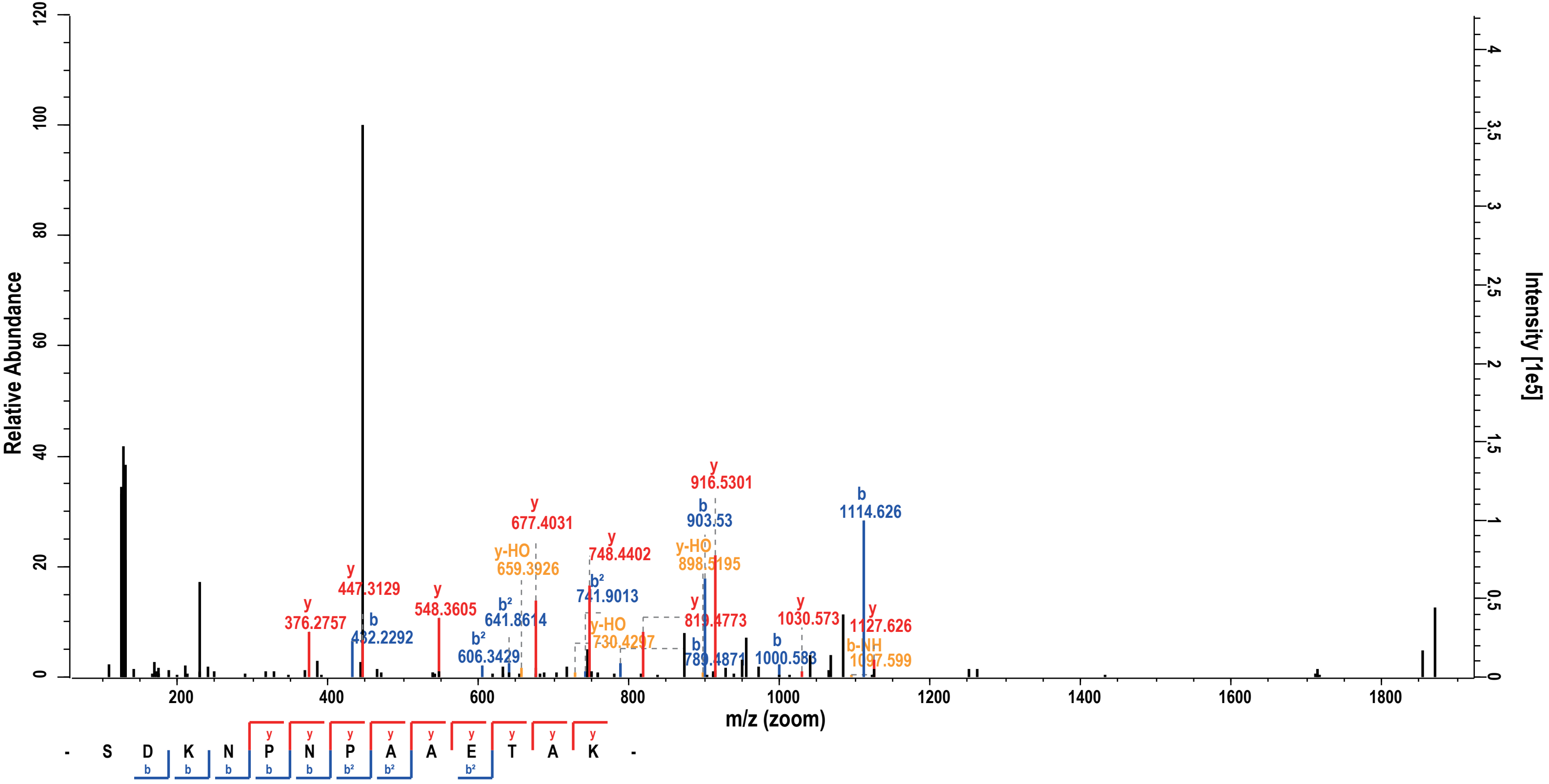

slr0112 unknown protein

Raw File	Scan	Method	Score	m/z
HCC_TMT_1_F9_20180514	15897	FTMS; HCD	98.01	762.42

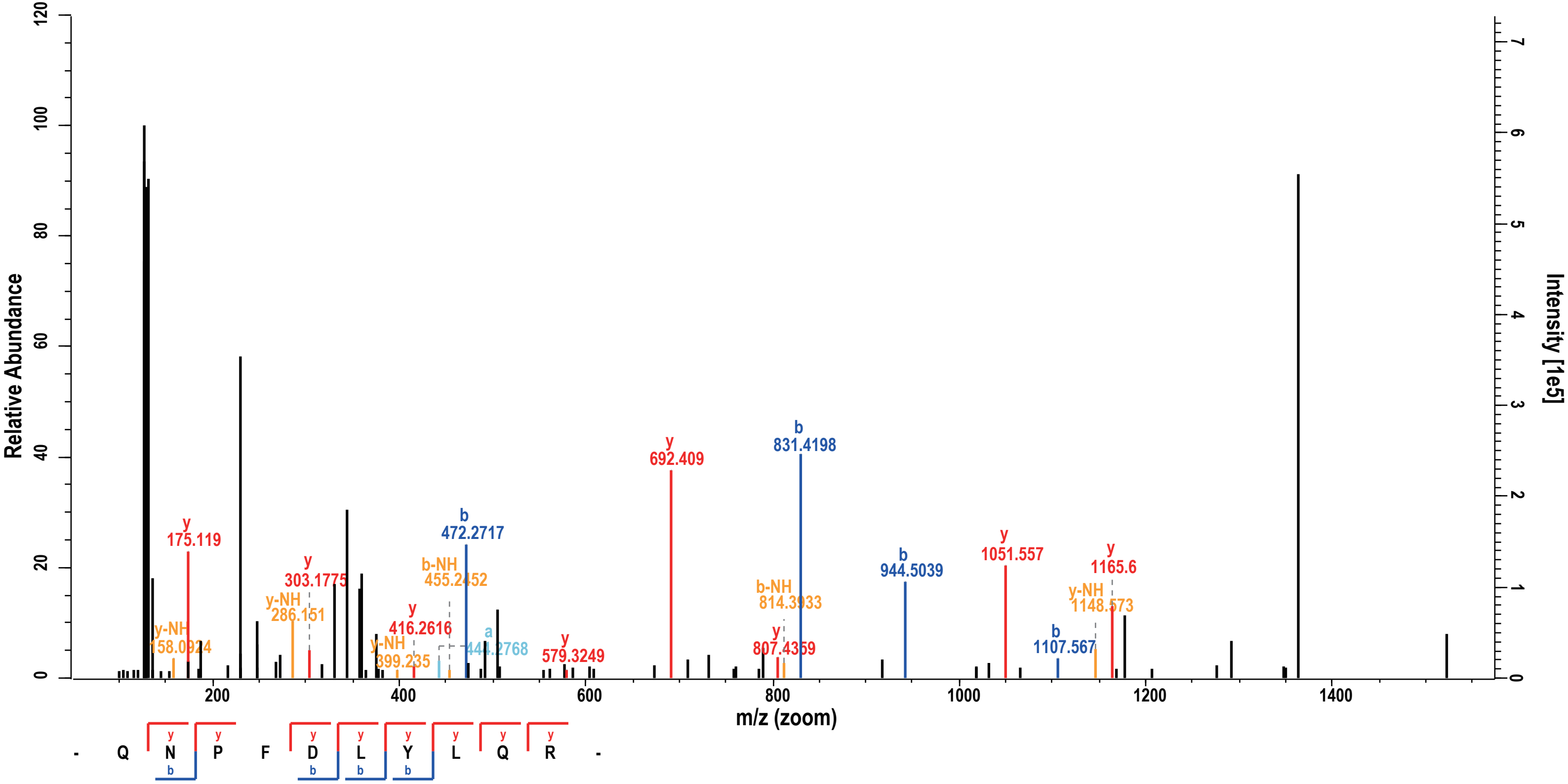

slr0243 hypothetical protein

Raw File	Scan	Method	Score	m/z
HCC_TMT_2_F4_20180514	10673	FTMS; HCD	94.69	807.47

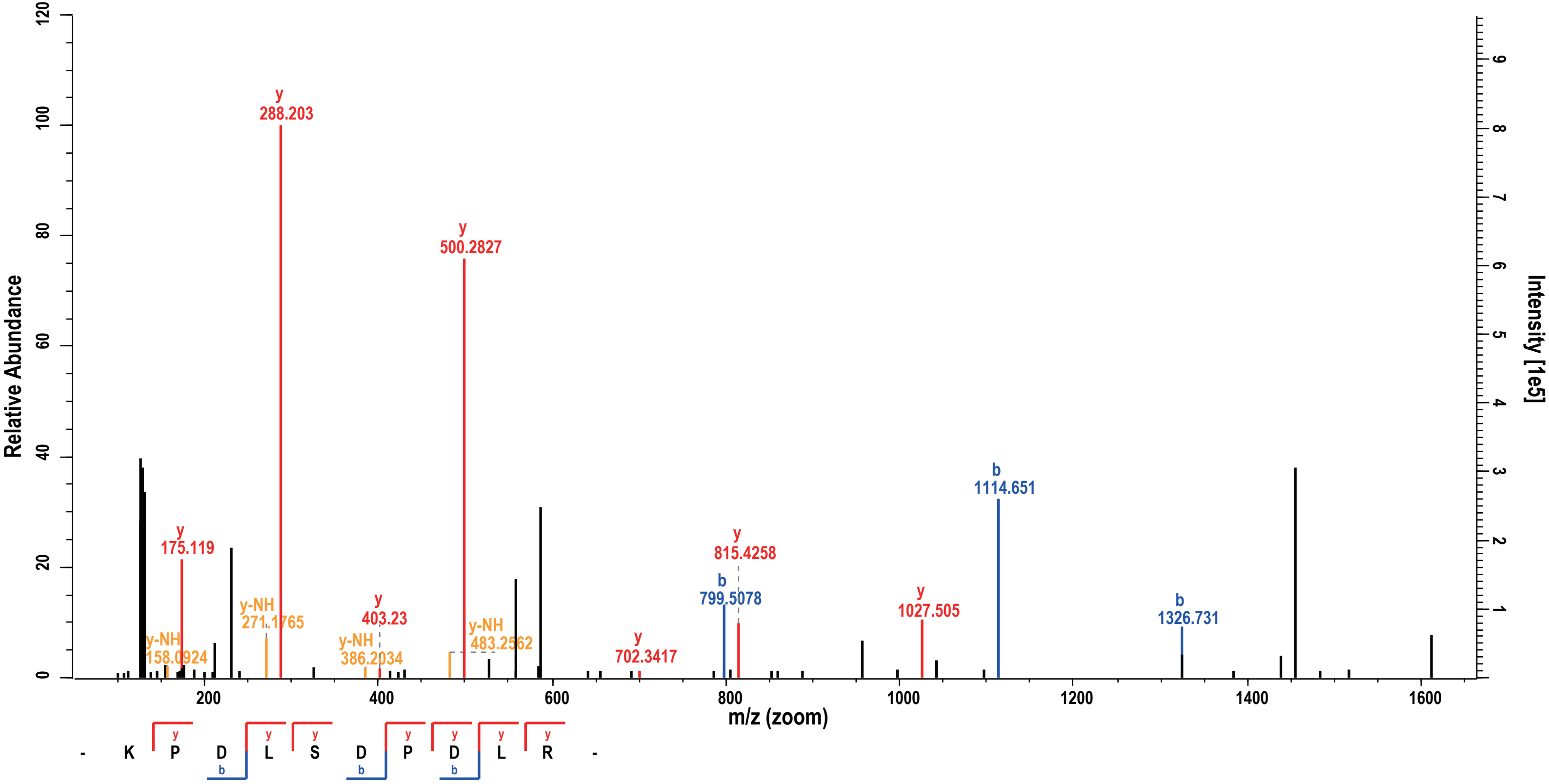

slr0343 cytochrome b6-f complex subunit 4

Raw File	Scan	Method	Score	m/z
HCC_TMT_1_F12_20180514	8392	FTMS; HCD	134.84	745.91

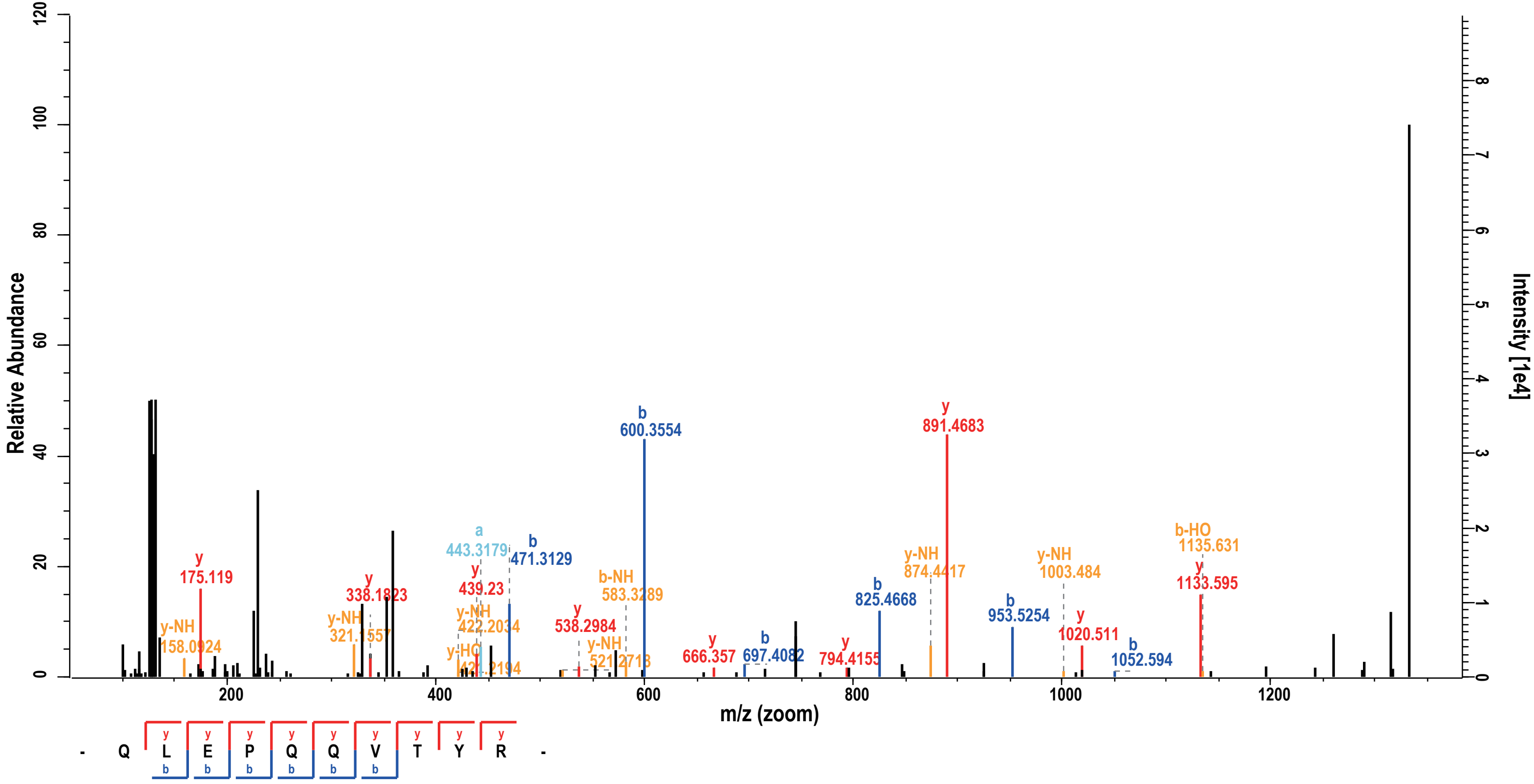

slr0345 unknown protein

Raw File	Scan	Method	Score	m/z
HCC_TMT_1_F10_20180514	7093	FTMS; HCD	209.1	754.39

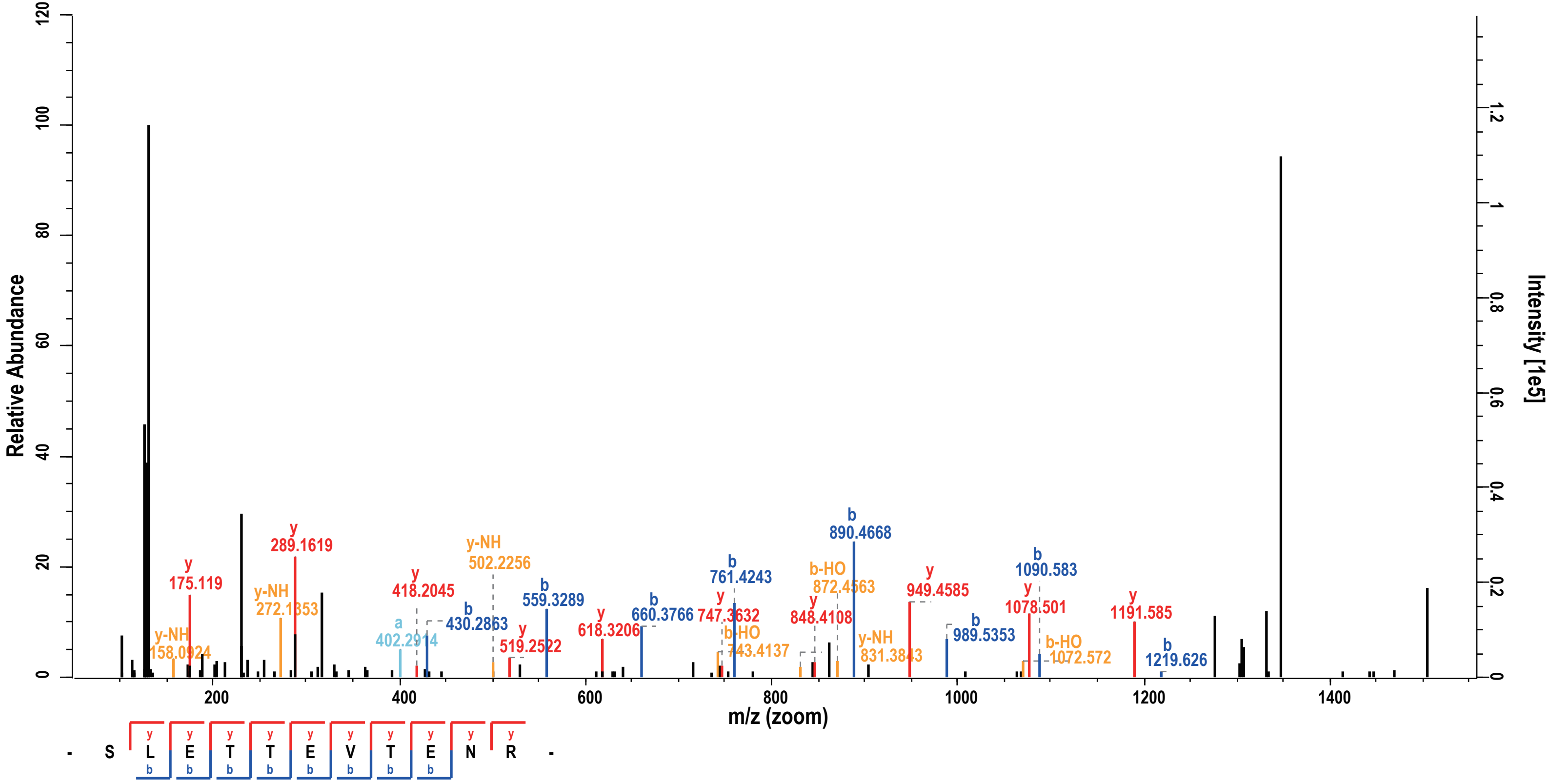

slr0358 unknown protein

Raw File	Scan	Method	Score	m/z
HCC_TMT_3_F3_20180514	15726	FTMS; HCD	129.47	633.86

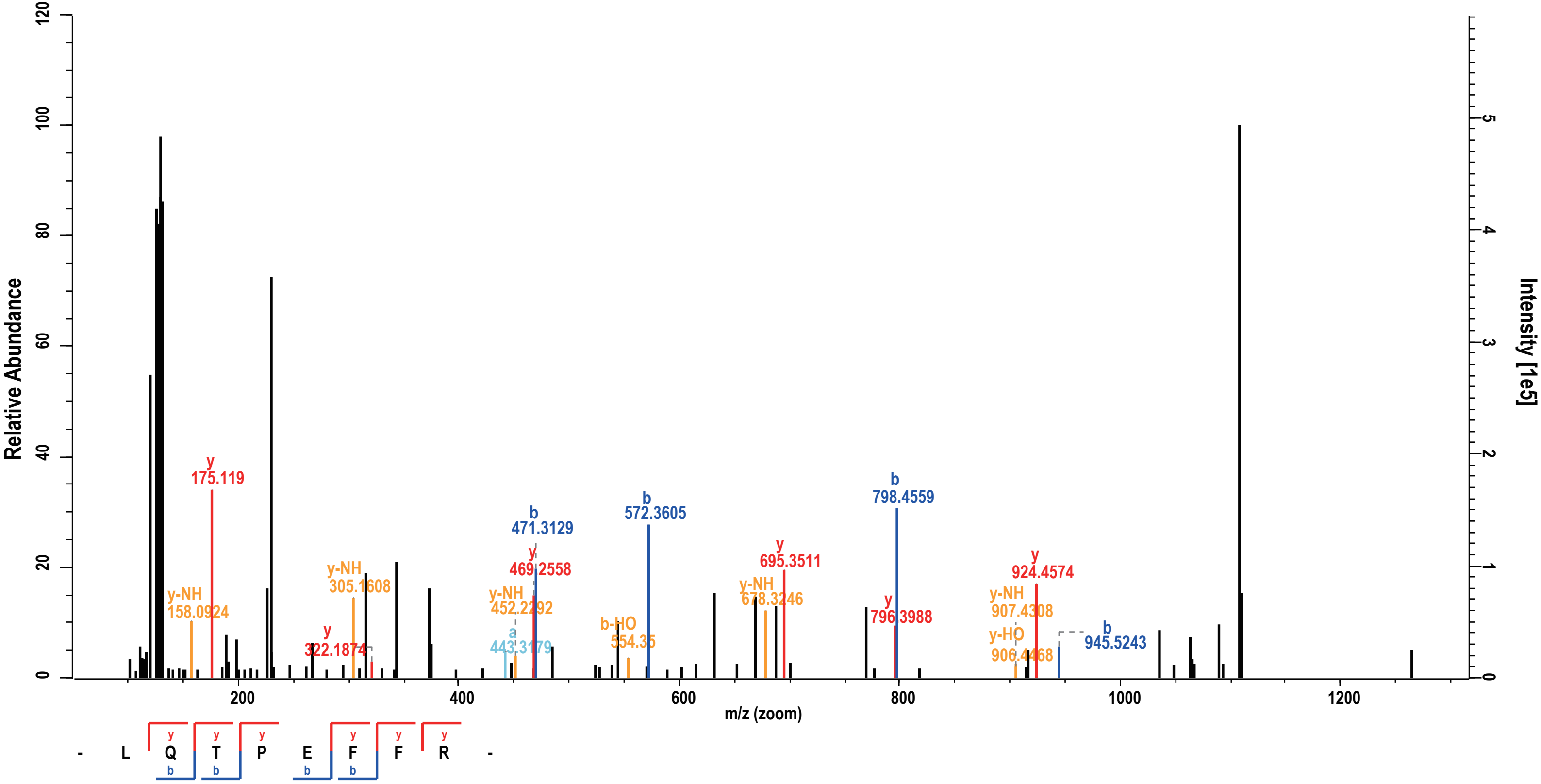

slr0400 hypothetical protein

Raw File	Scan	Method	Score	m/z
HCC_TMT_2_F9_20180514	5307	FTMS; HCD	66.43	569

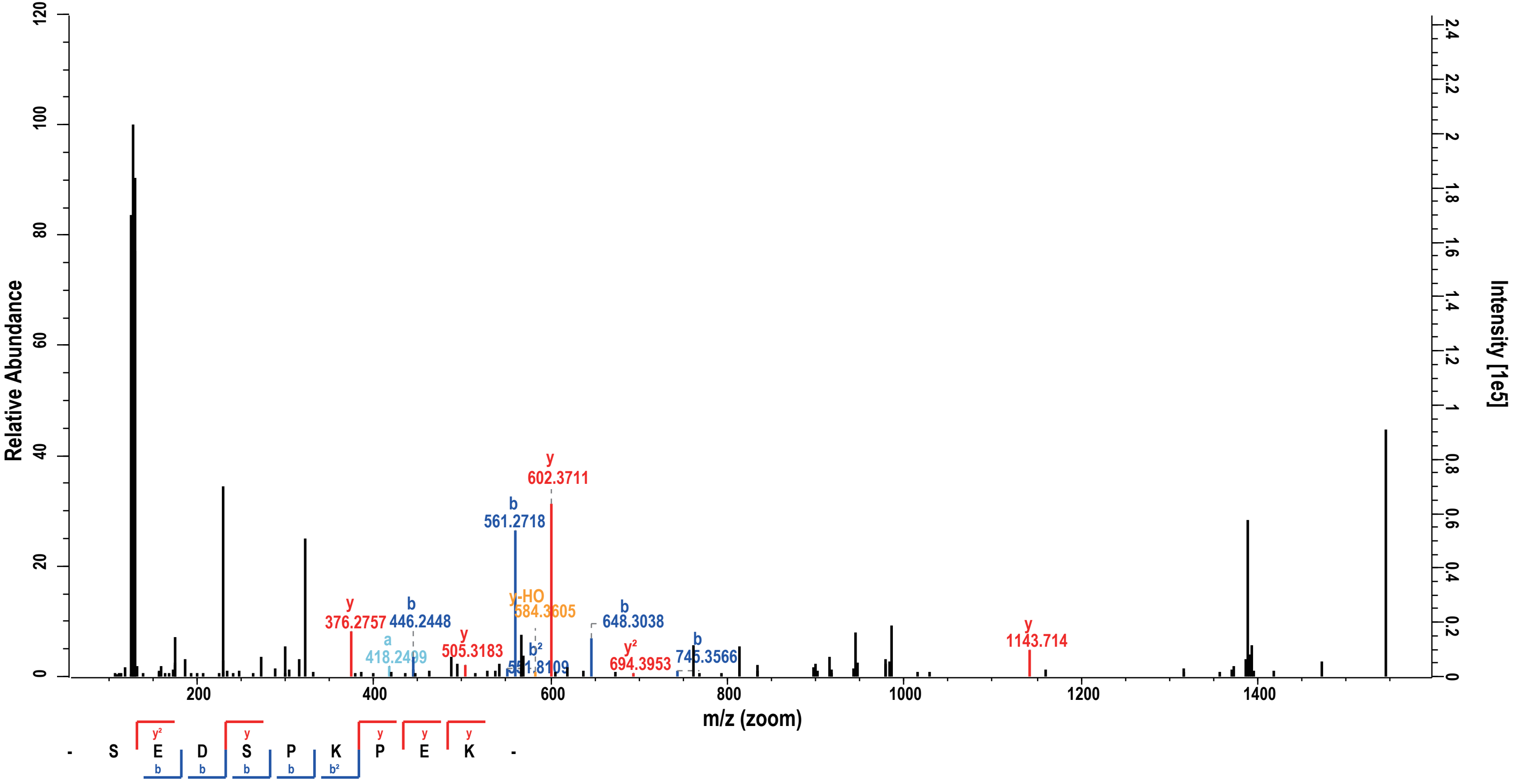

slr0438 hypothetical protein

Raw File	Scan	Method	Score	m/z
HCC_TMT_2_F15_20180514	9223	FTMS; HCD	116.24	826.4

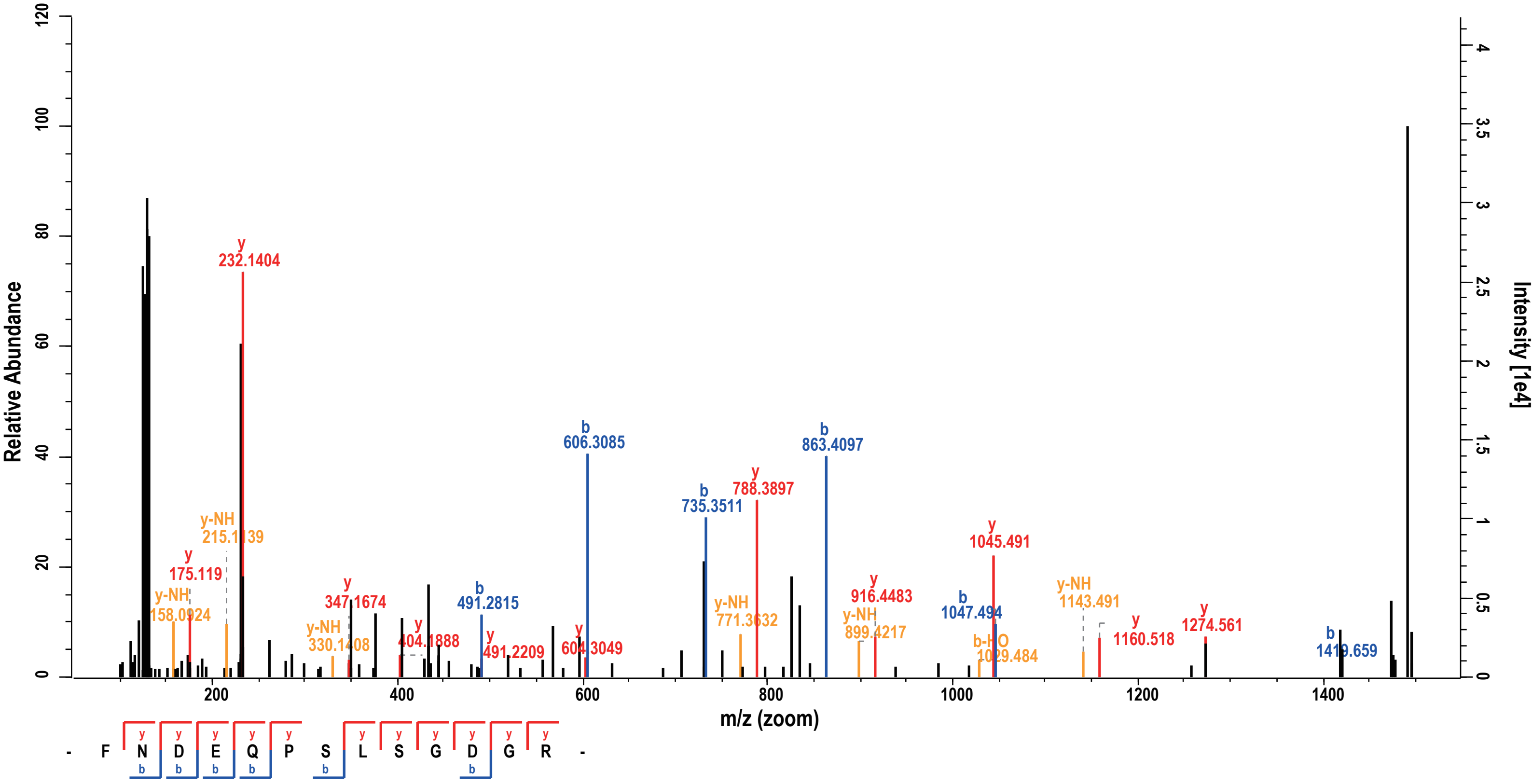

slr0505 hypothetical protein

Raw File	Scan	Method	Score	m/z
HCC_TMT_2_F6_20180514	12658	FTMS; HCD	83.25	989.16

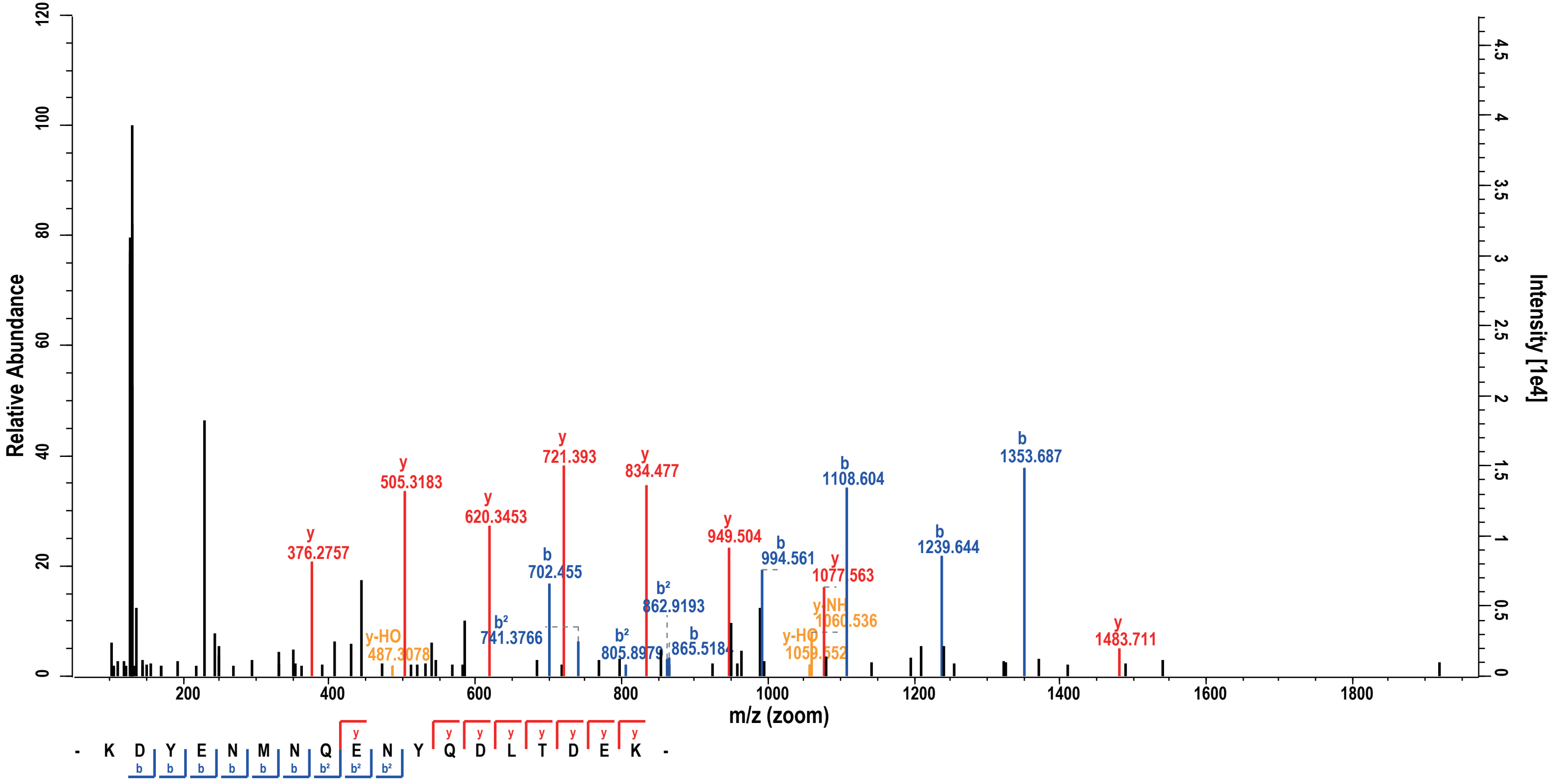

slr0582 unknown protein

Raw File	Scan	Method	Score	m/z
HCC_TMT_3_F3_20180514	12053	FTMS; HCD	142.92	776.49

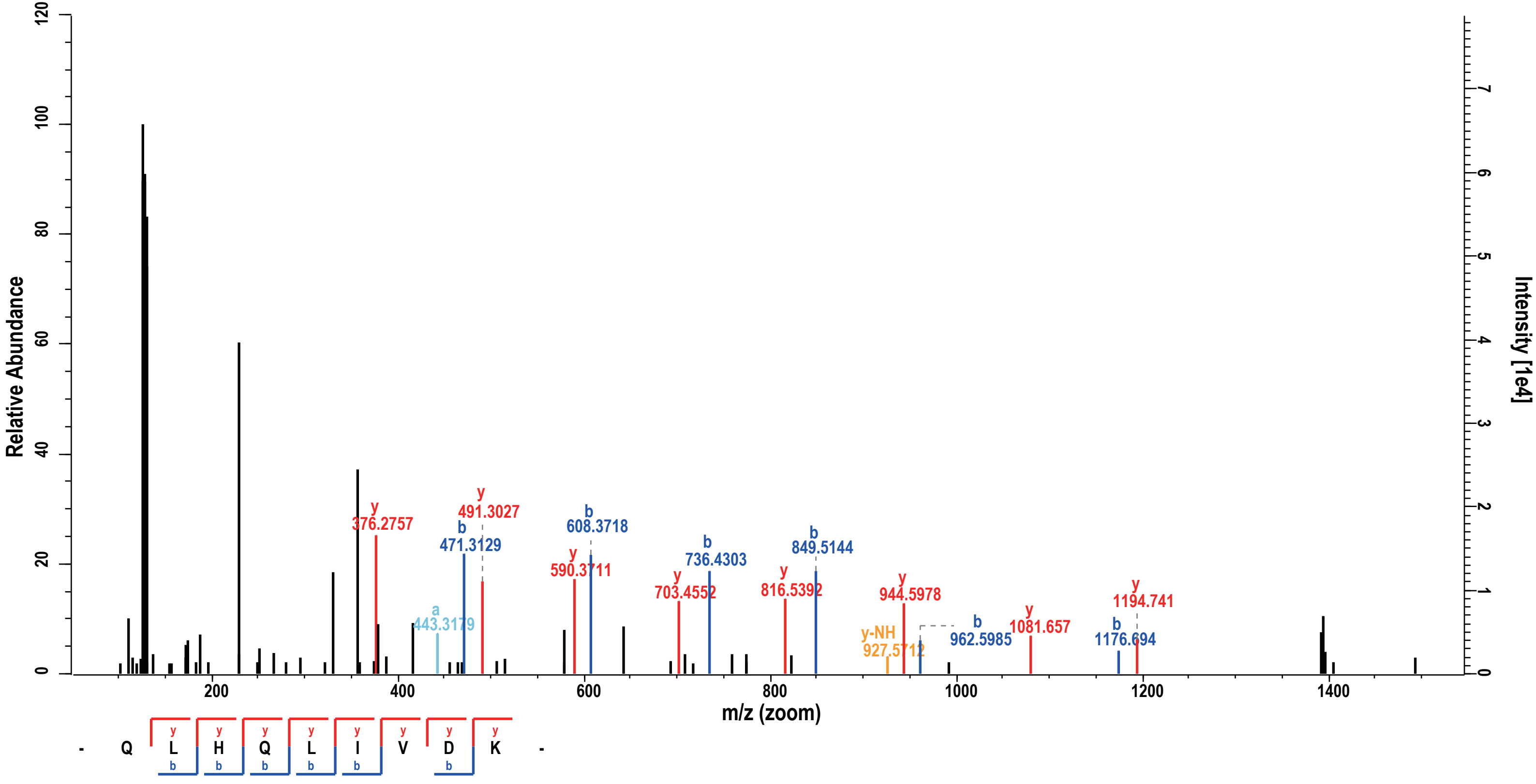

slr0598 hypothetical protein

Raw File	Scan	Method	Score	m/z
HCC_TMT_1_F6_20180514	4372	FTMS; HCD	117.16	525.28

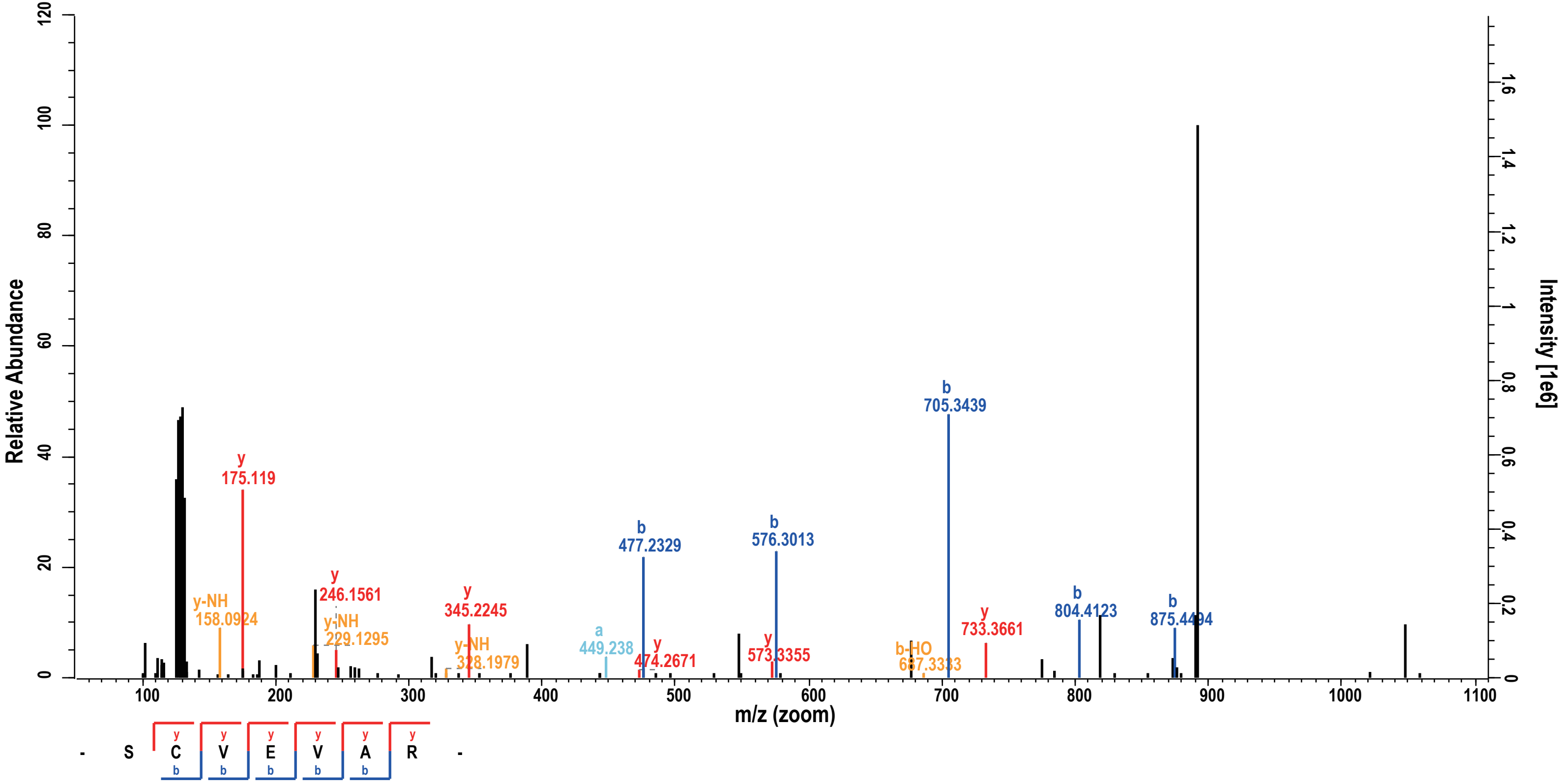

slr0709 hypothetical protein

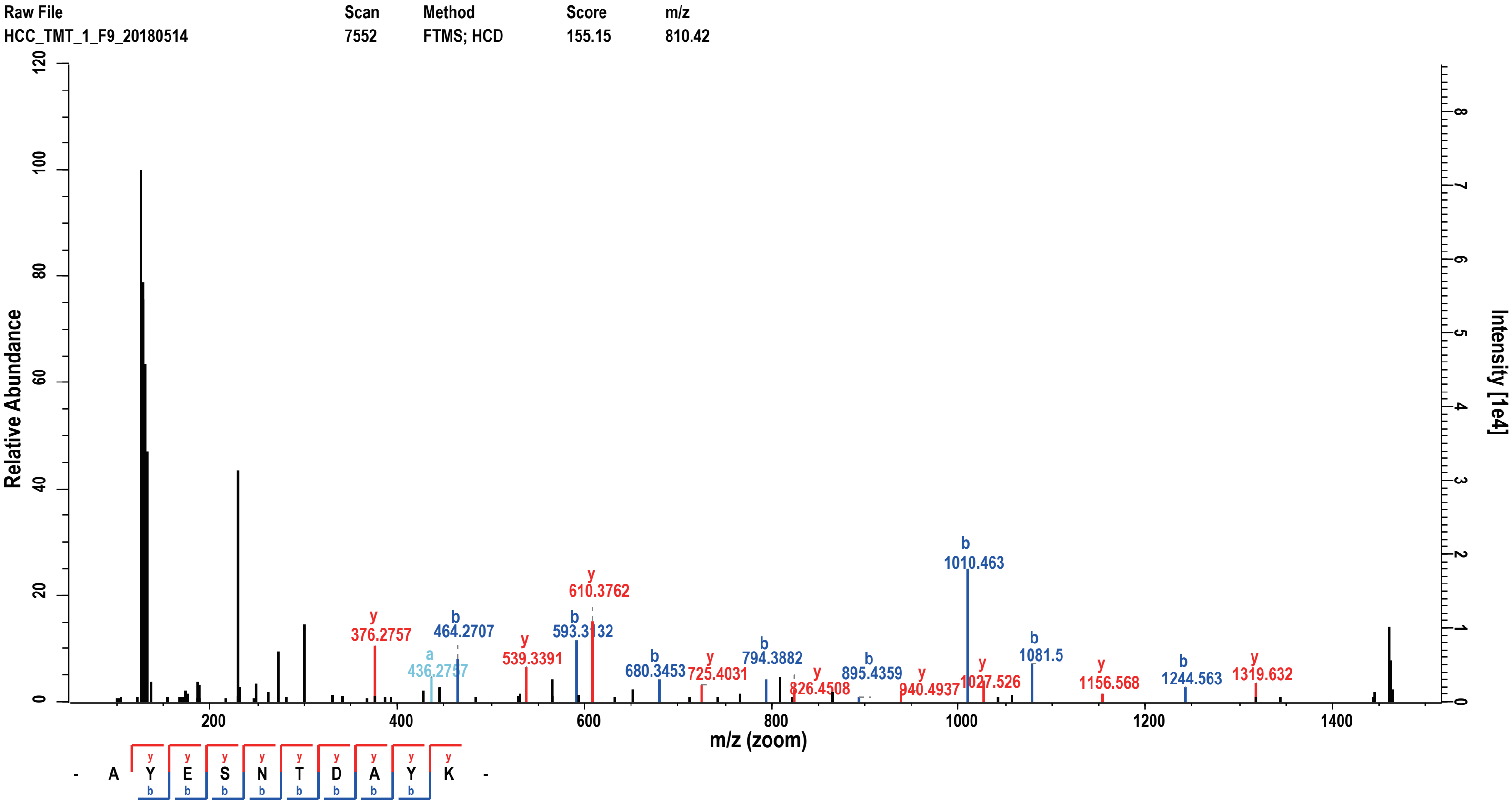

slr0725 hypothetical protein

Raw File	Scan	Method	Score	m/z
HCC_TMT_2_F6_20180514	11060	FTMS; HCD	94.69	754.92

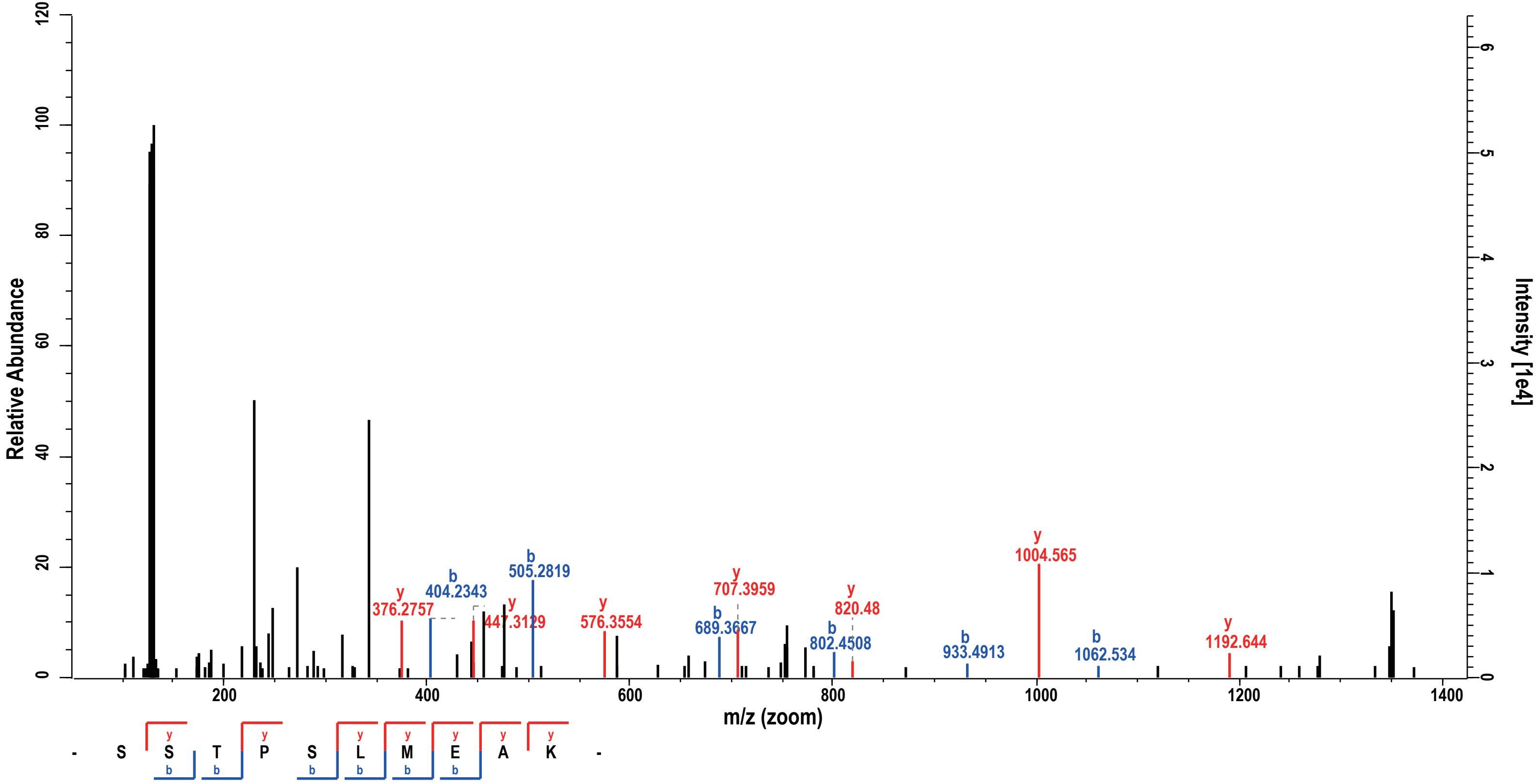

slr0770 hypothetical protein

Raw File	Scan	Method	Score	m/z
HCC_TMT_2_F14_20180514	2823	FTMS; HCD	125.08	508.79

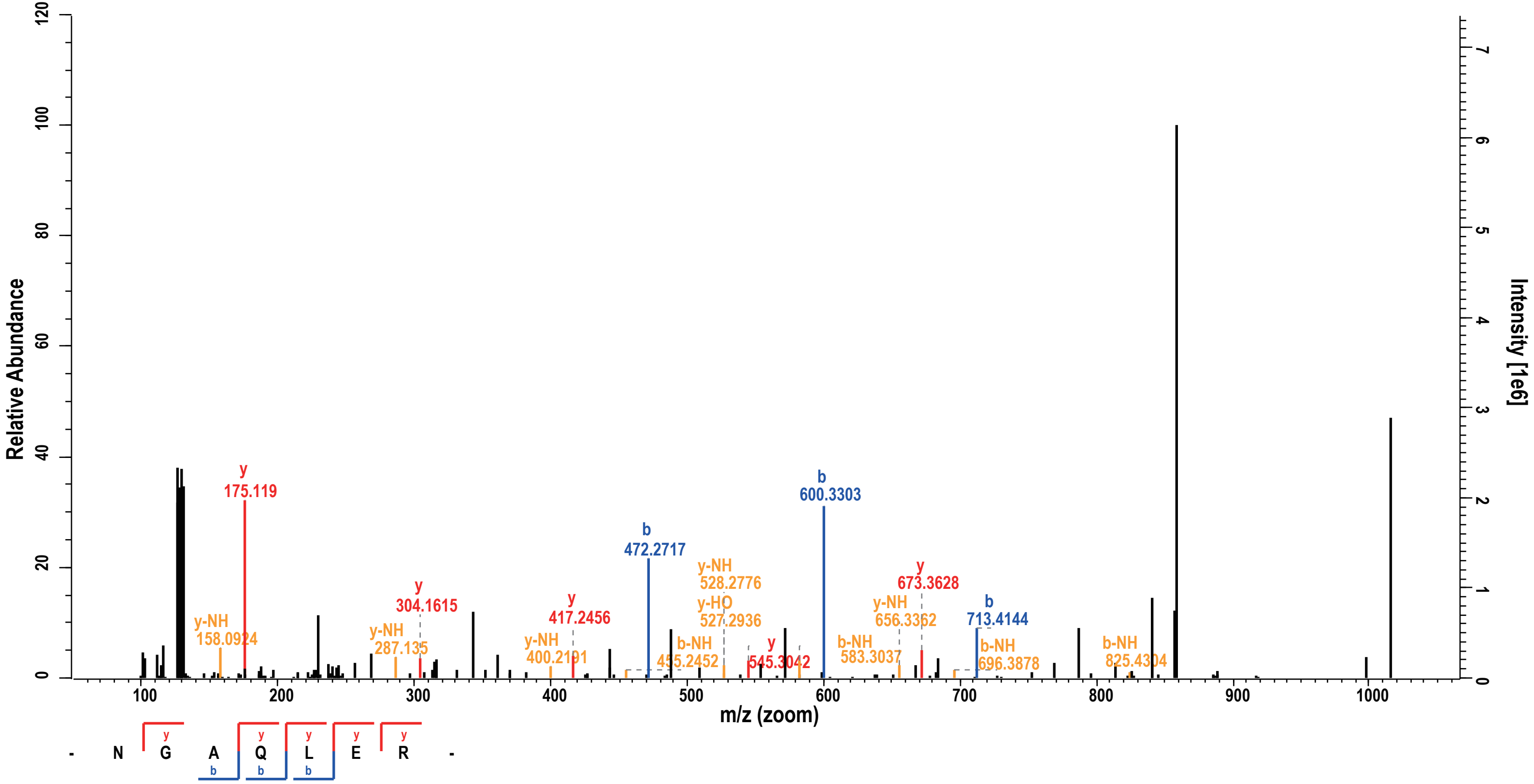

slr0797 cobalt-transporting P-type ATPase (cobalt efflux pump) involved in cobalt tolerance

Raw File	Scan	Method	Score	m/z
HCC_TMT_1_F6_20180514	8433	FTMS; HCD	110.41	623.35

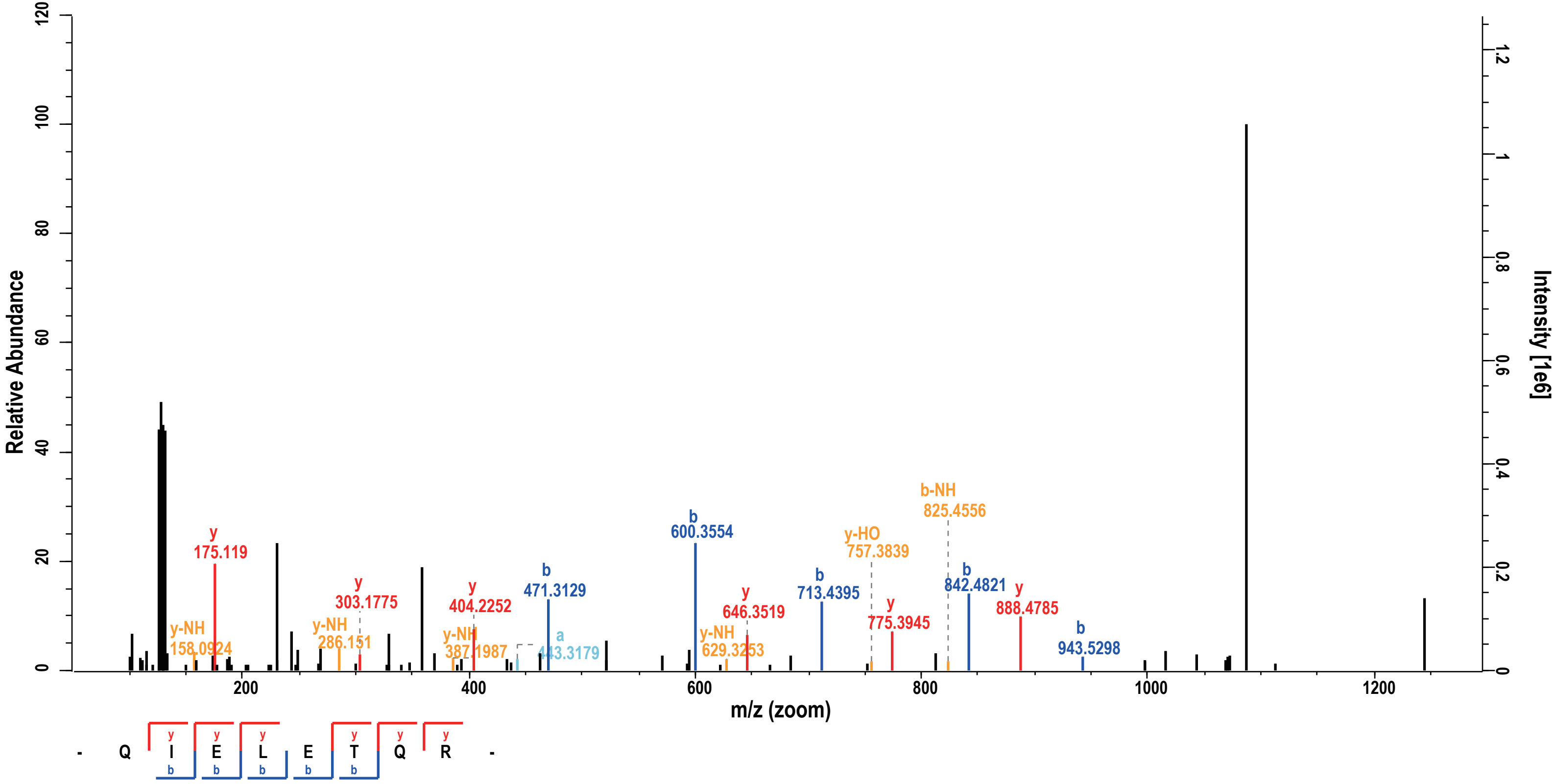

slr0852 hypothetical protein

Raw File	Scan	Method	Score	m/z
HCC_TMT_2_F13_20180514	10809	FTMS; HCD	143.89	737.44

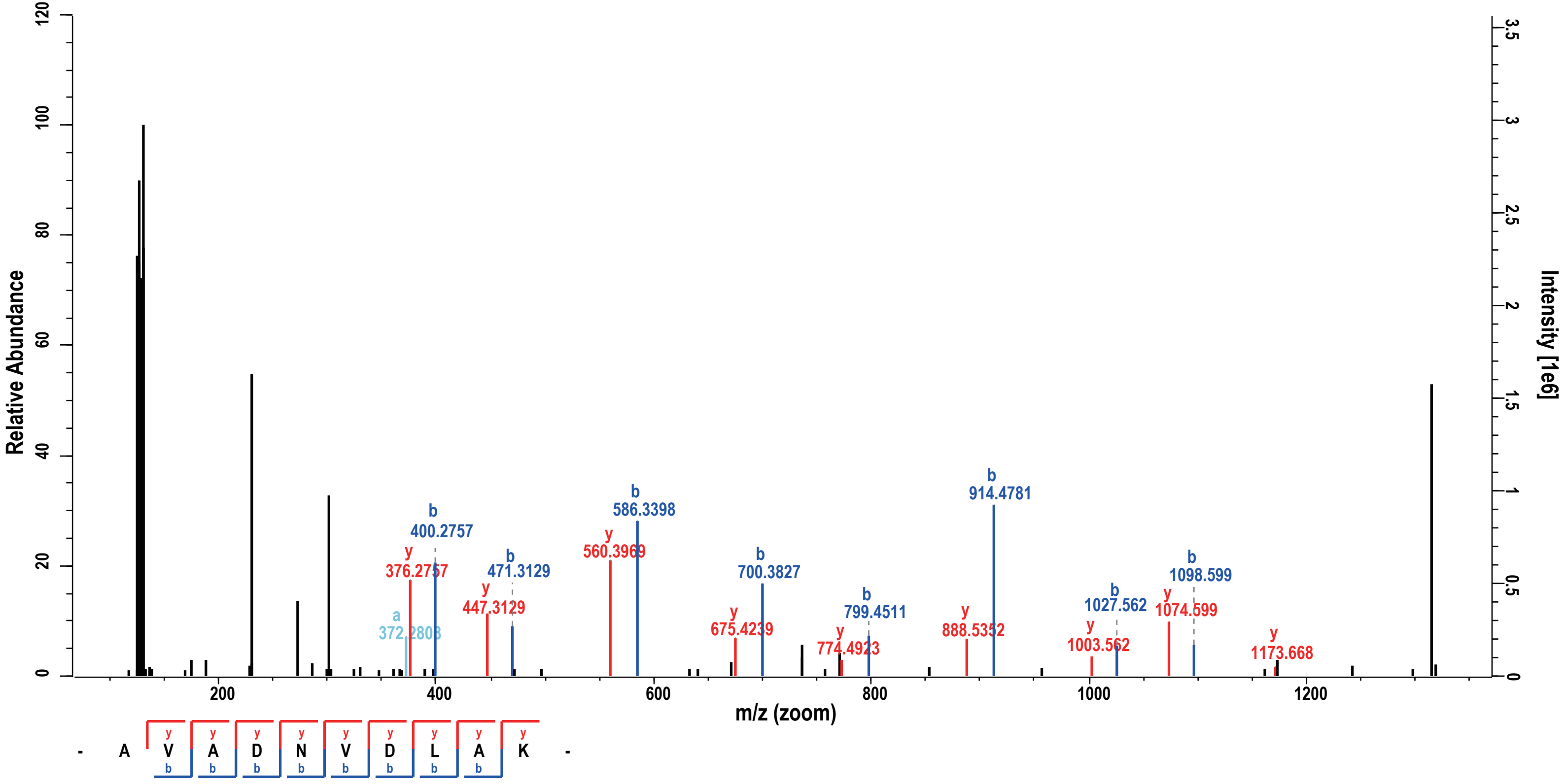

slr0949 Integral membrane protein of the ABC-type Nat permease for neutral amino acids NatD

Raw File	Scan	Method	Score	m/z
HCC_TMT_1_F8_20180514	1768	FTMS; HCD	141.52	518.78

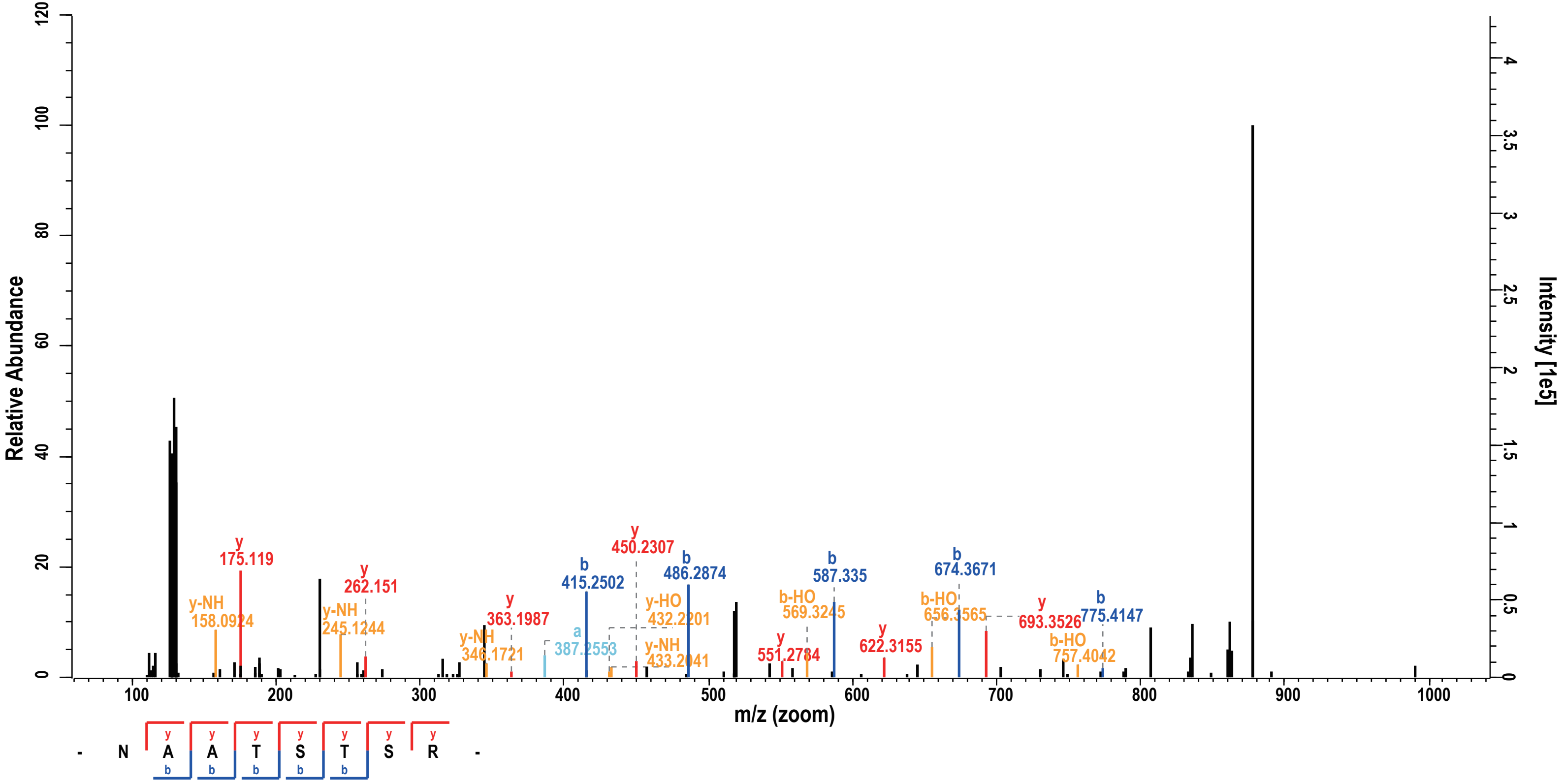

slr0954 hypothetical protein

Raw File	Scan	Method	Score	m/z
HCC_TMT_2_F4_20180514	11637	FTMS; HCD	119.76	971.56

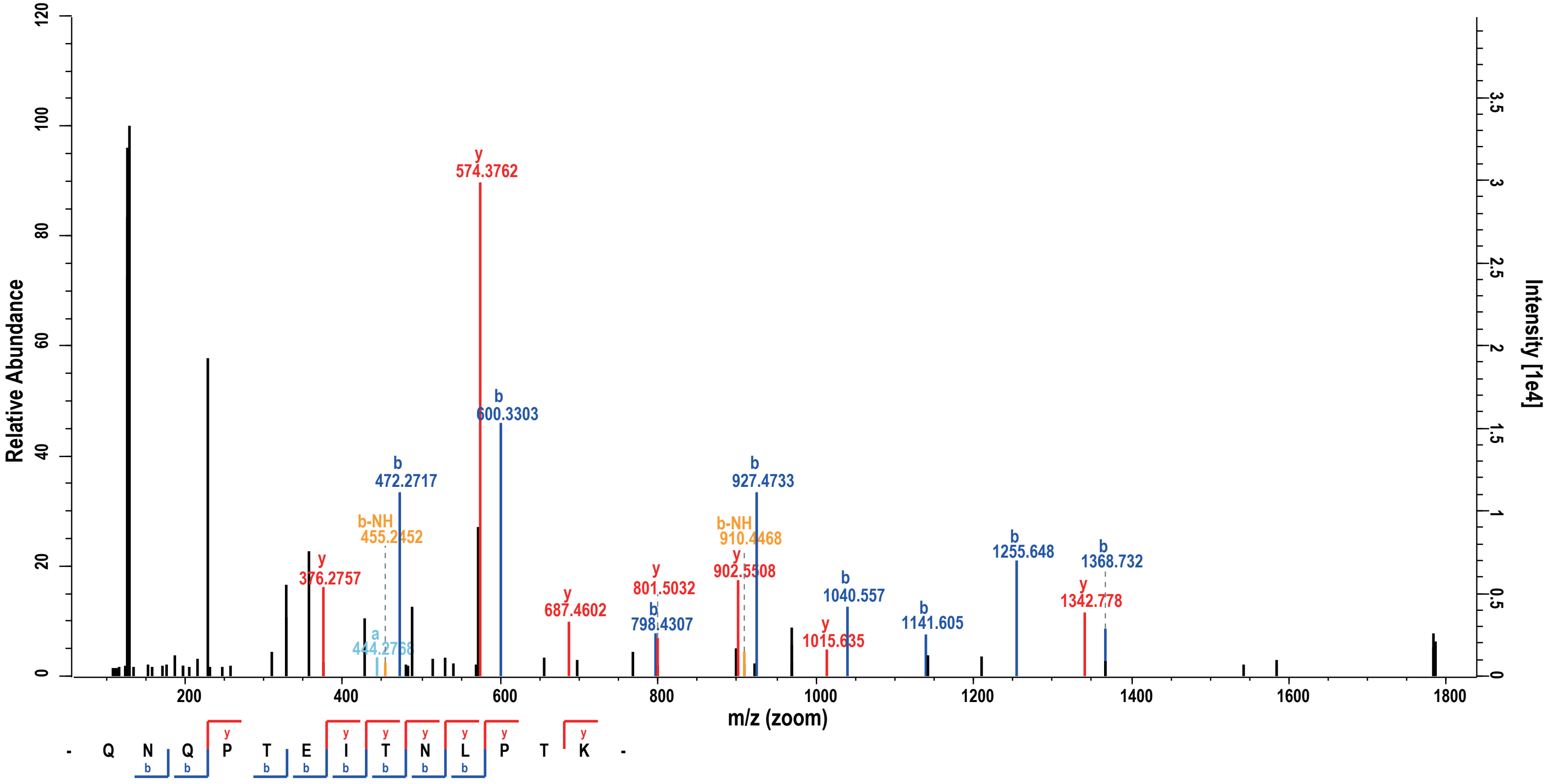

slr0981 hypothetical protein

Raw File	Scan	Method	Score	m/z
HCC_TMT_3_F14_20180514	16501	FTMS; HCD	124.29	742.39

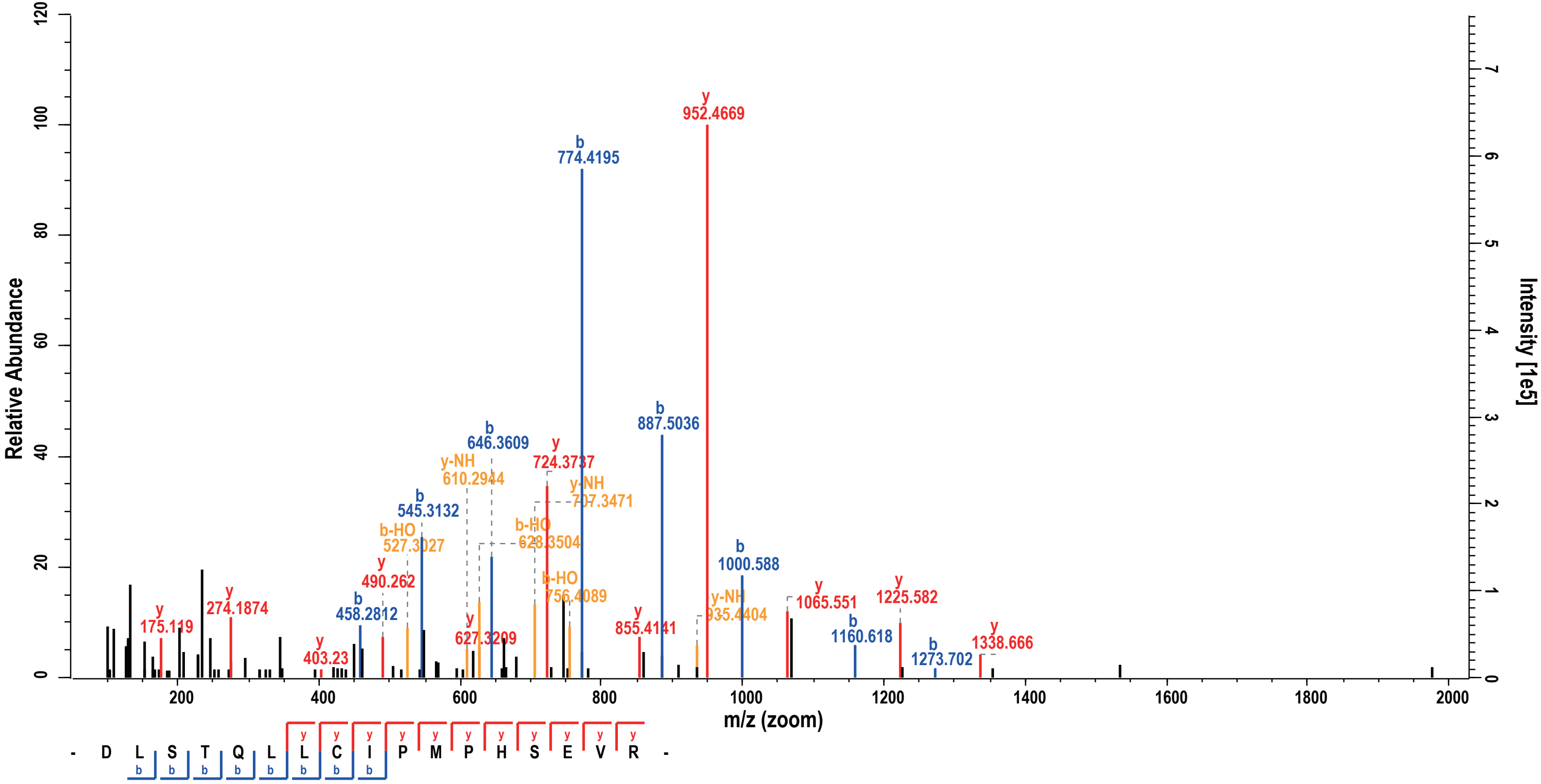

slr0992 probable tRNA/rRNA methyltransferase

Raw File	Scan	Method	Score	m/z
HCC_TMT_3_F8_20180514	5842	FTMS; HCD	72.64	737.87

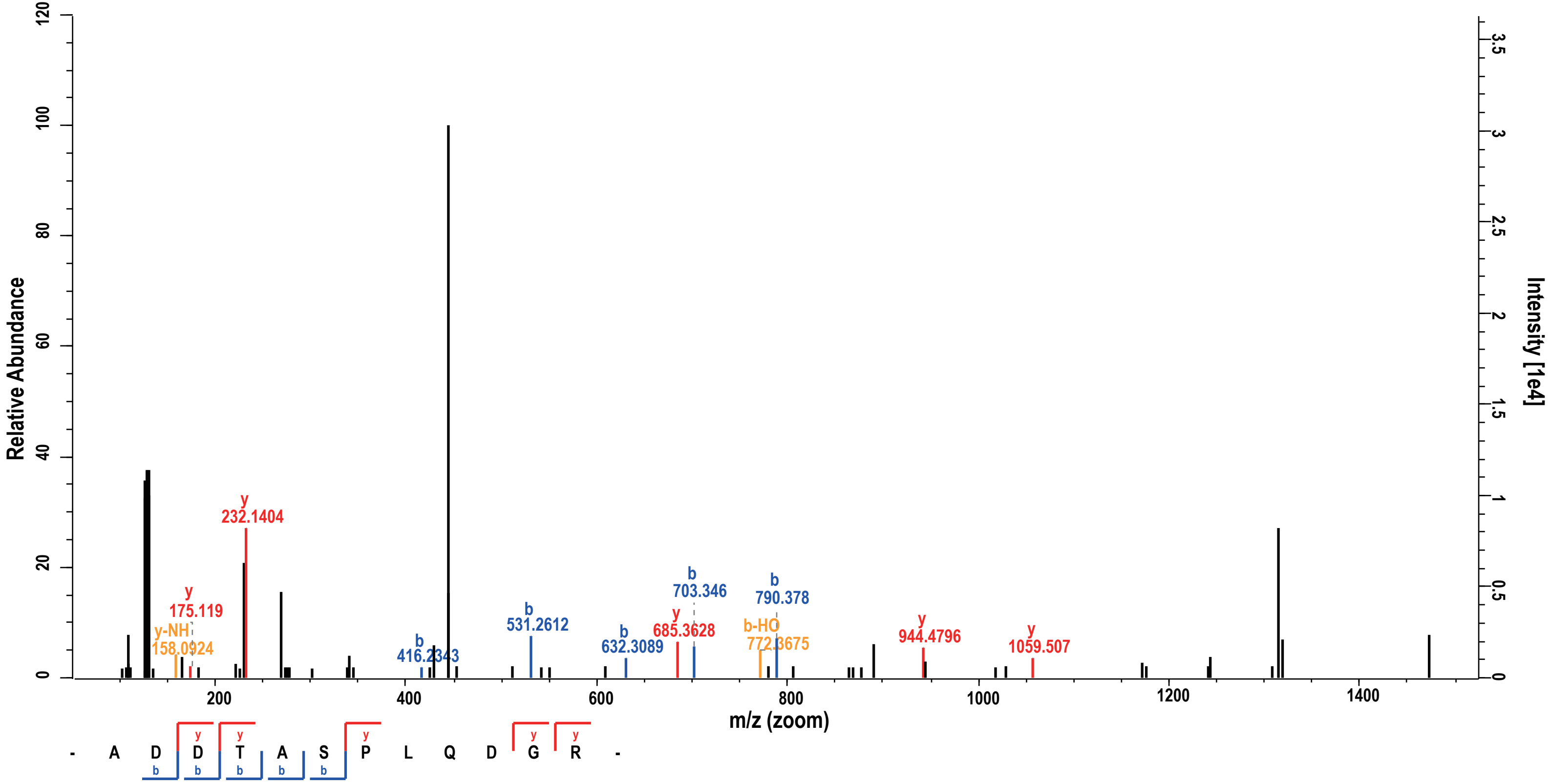

slr1074 unknown protein

Raw File	Scan	Method	Score	m/z
HCC_TMT_2_F12_20180514	5476	FTMS; HCD	177.3	716.4

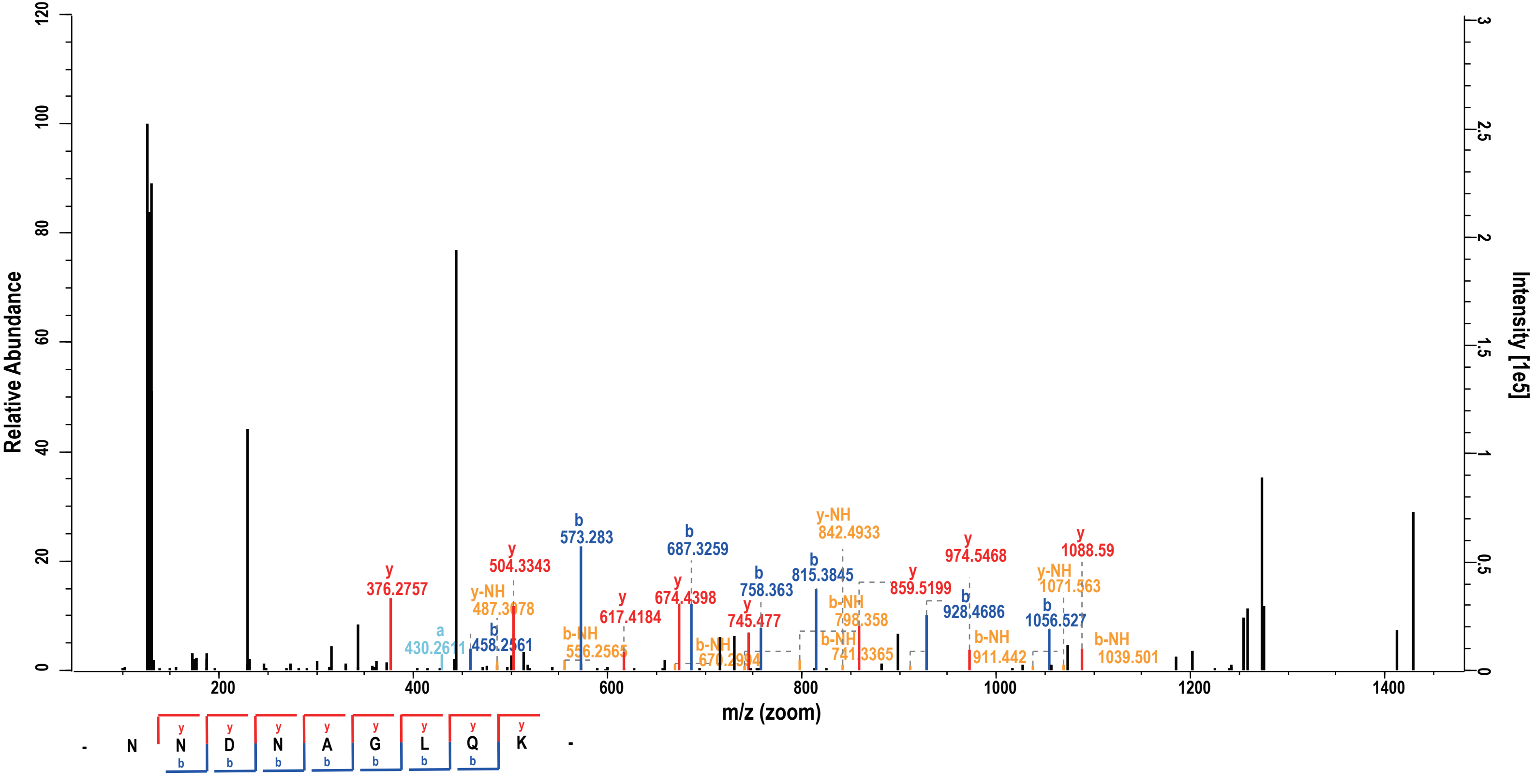

slr1138 cytochrome c oxidase subunit III

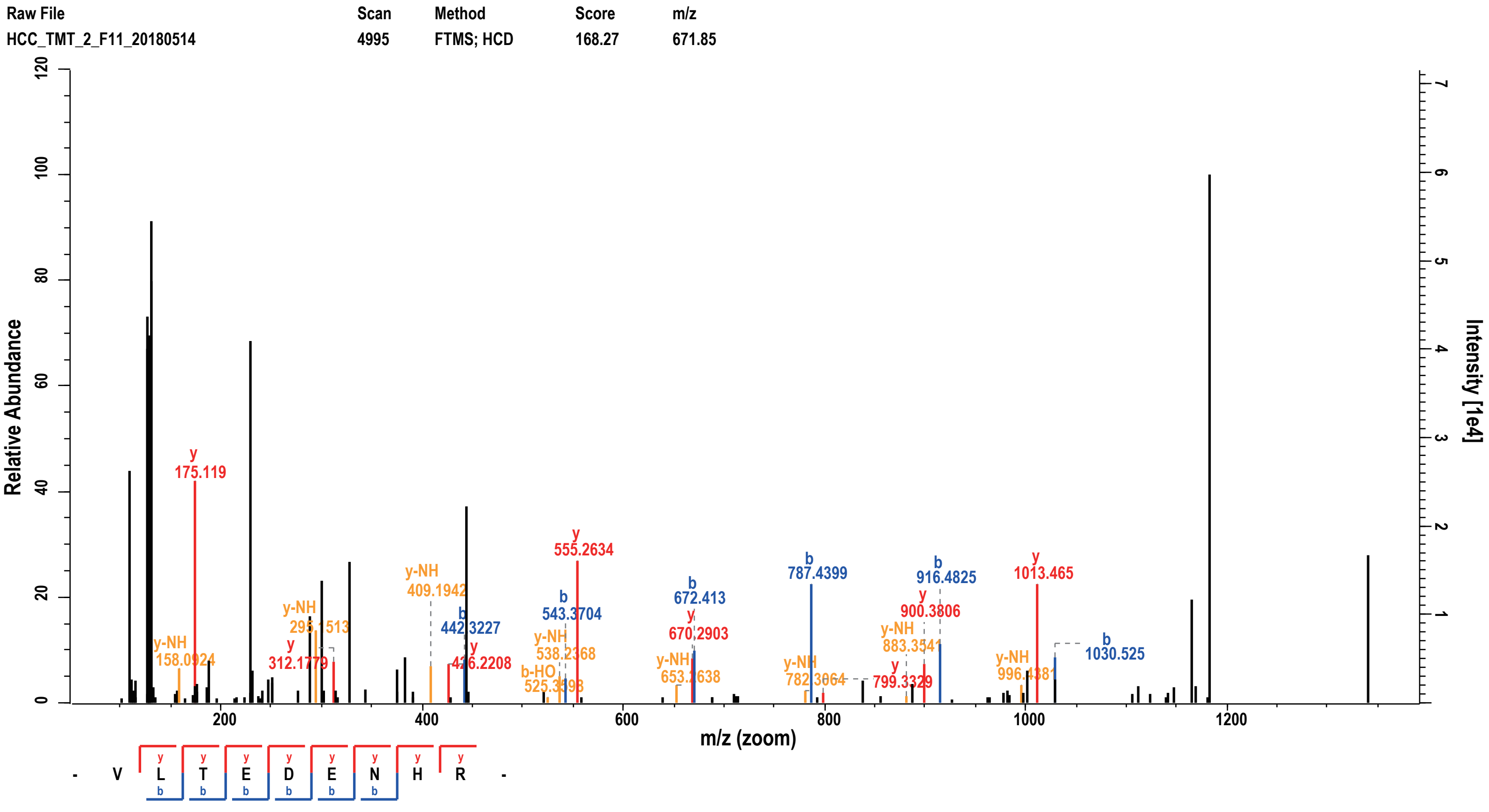

slr1229 sulfate permease

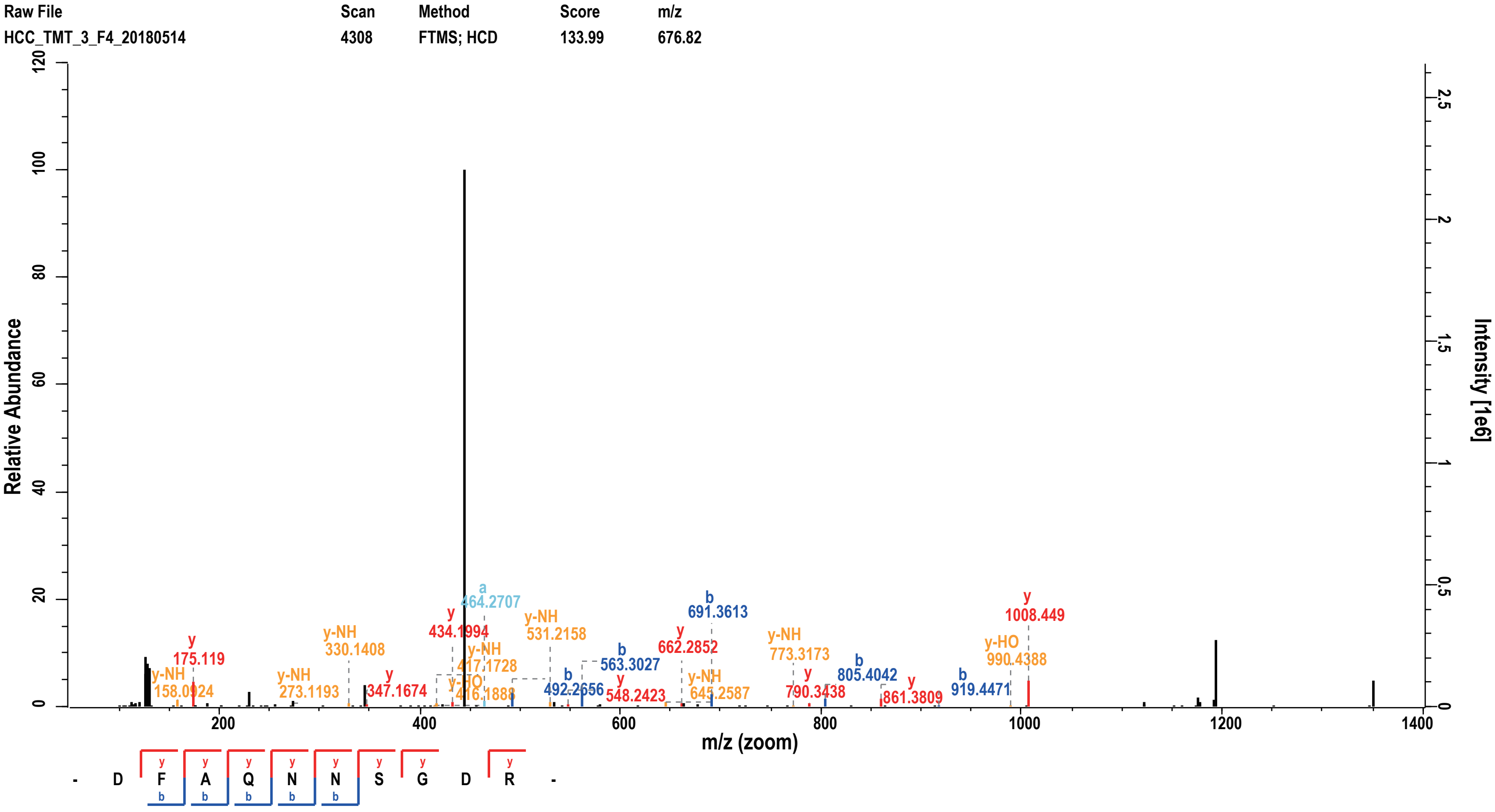

slr1443 serine/threonine kinase

Raw File	Scan	Method	Score	m/z
HCC_TMT_1_F9_20180514	9885	FTMS; HCD	144.9	605.36

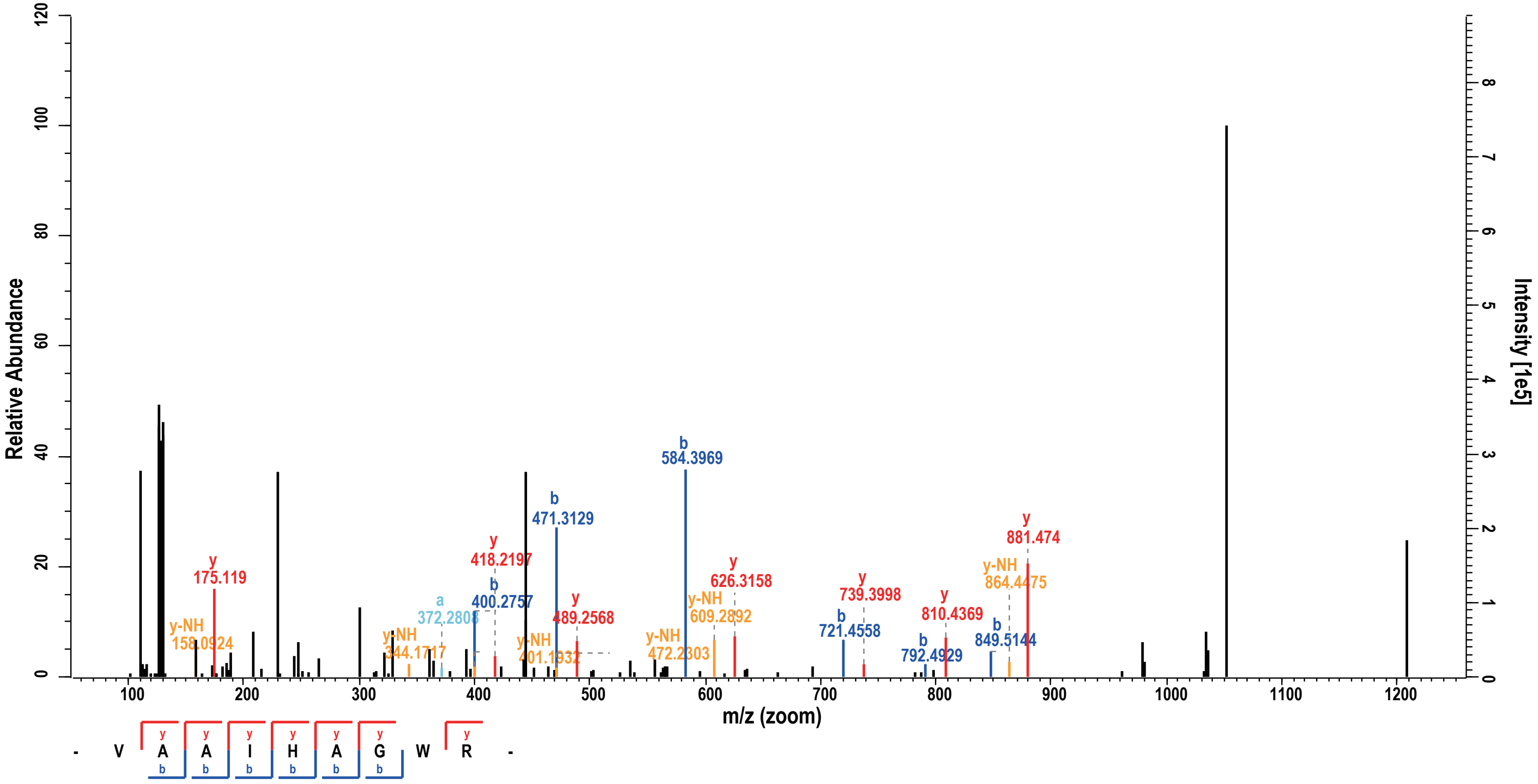

slr1573 hypothetical protein

Raw File	Scan	Method	Score	m/z
HCC_TMT_1_F13_20180514	10264	FTMS; HCD	133.81	851.47

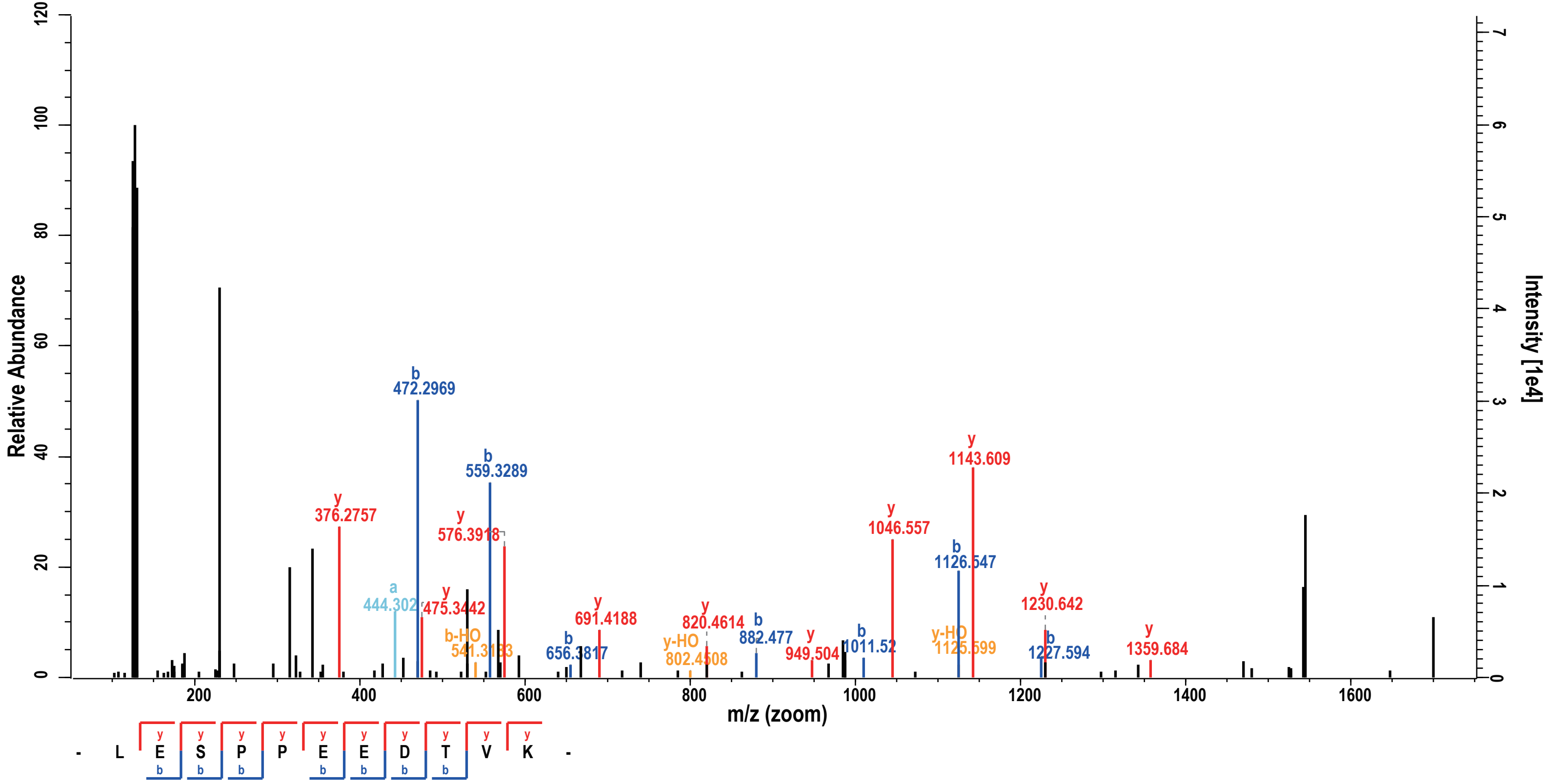

slr1584 two-component transcription regulator OmpR subfamily

Raw File	Scan	Method	Score	m/z
HCC_TMT_1_F13_20180514	15428	FTMS; HCD	137.05	657.9

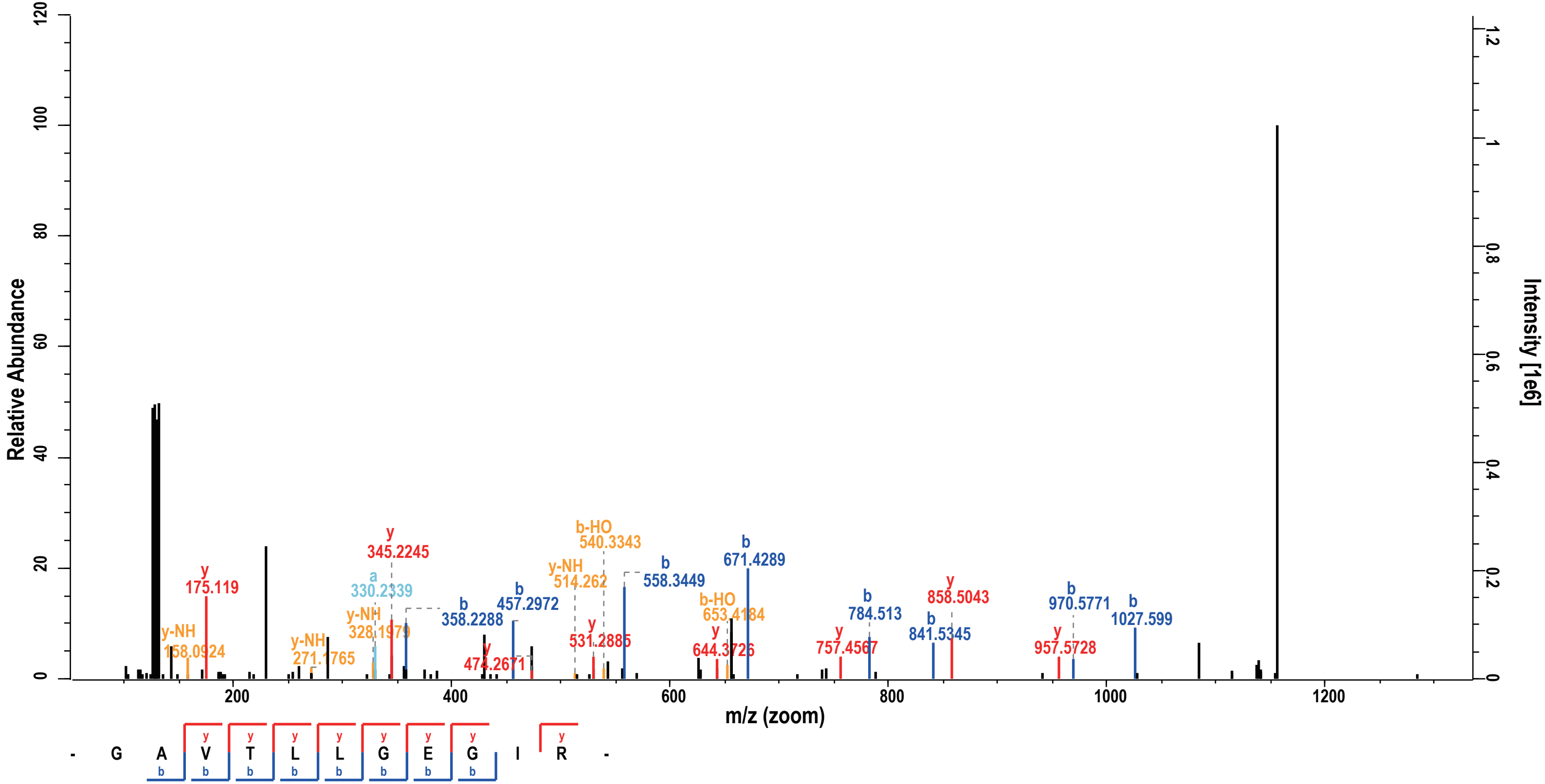

slr1636 unknown protein

Raw File	Scan	Method	Score	m/z
HCC_TMT_3_F3_20180514	8535	FTMS; HCD	136.57	663.89

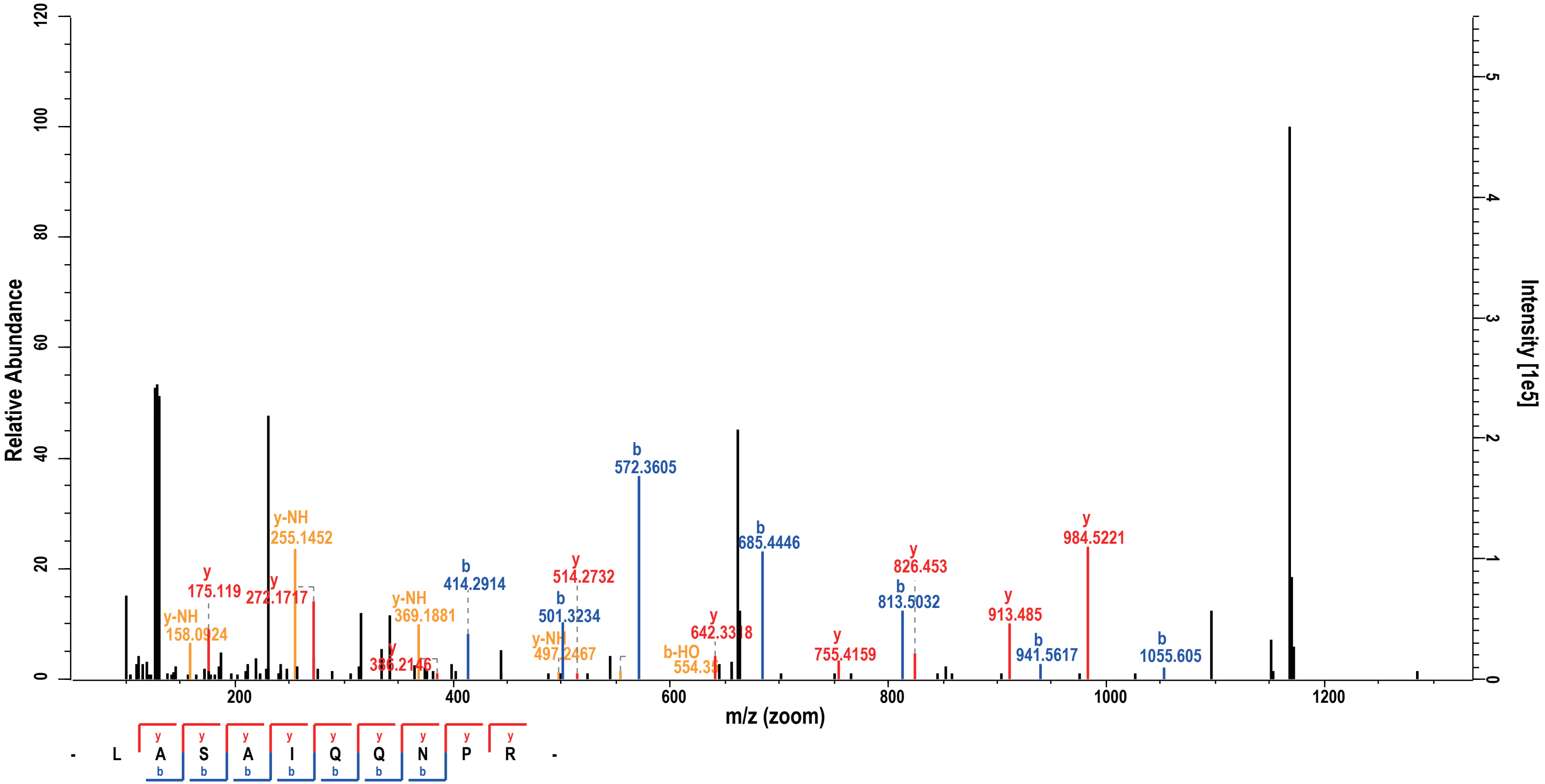

slr1647 hypothetical protein

Raw File	Scan	Method	Score	m/z
HCC_TMT_3_F9_20180514	8527	FTMS; HCD	112.37	496.31

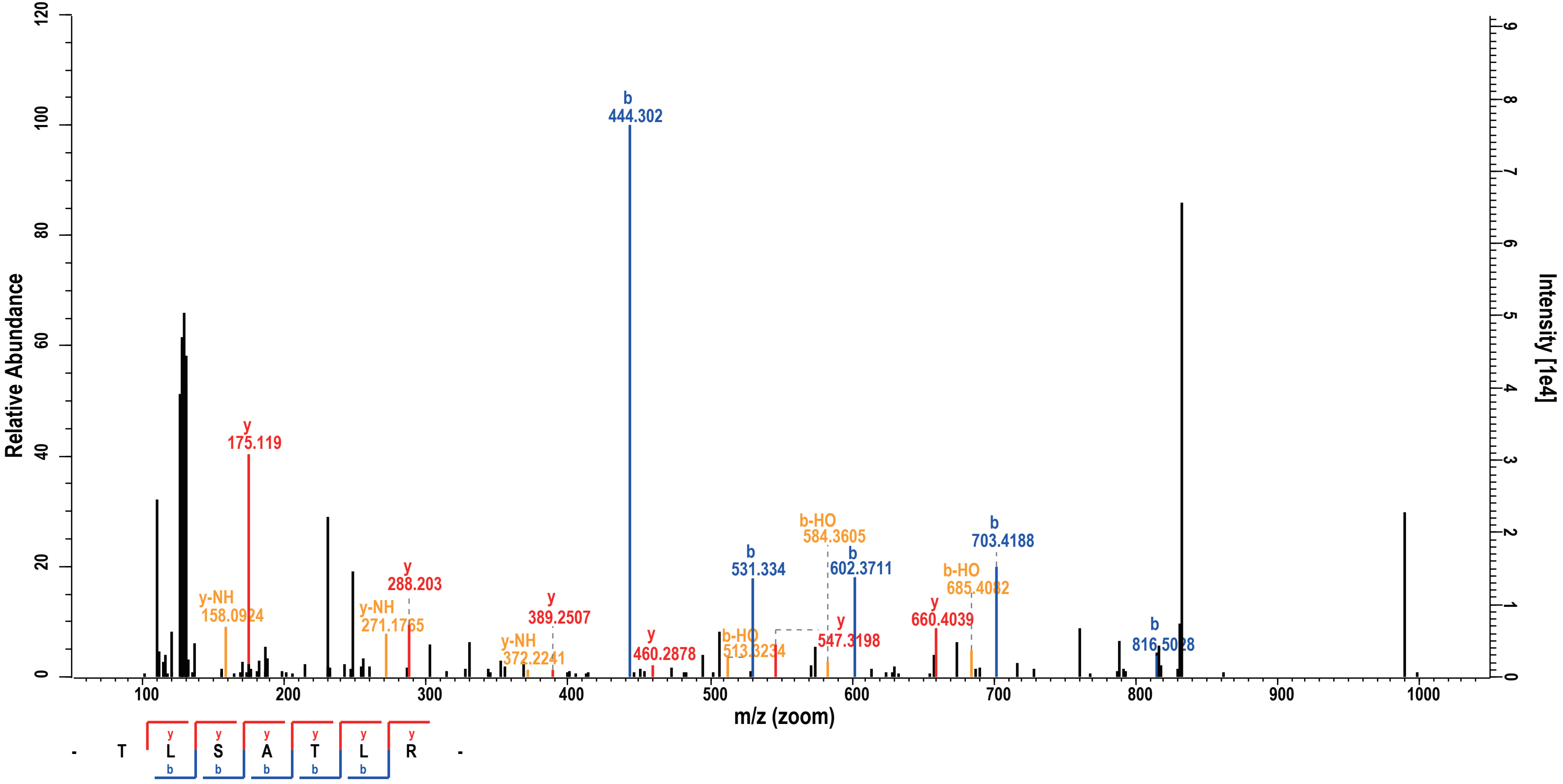

slr1657 hypothetical protein

Raw File	Scan	Method	Score	m/z
HCC_TMT_3_F8_20180514	2774	FTMS; HCD	101.41	637.88

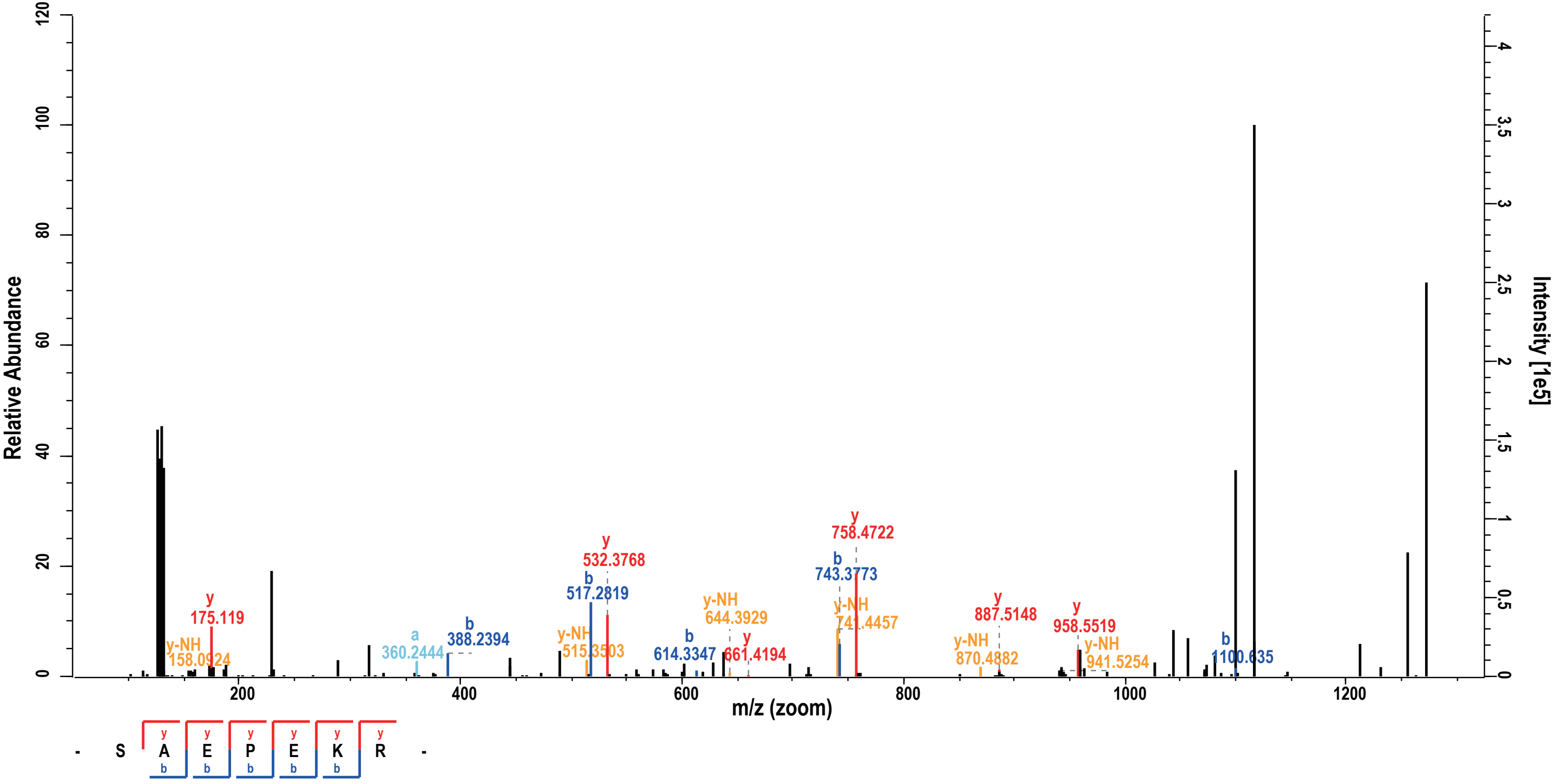

slr1676 hypothetical protein

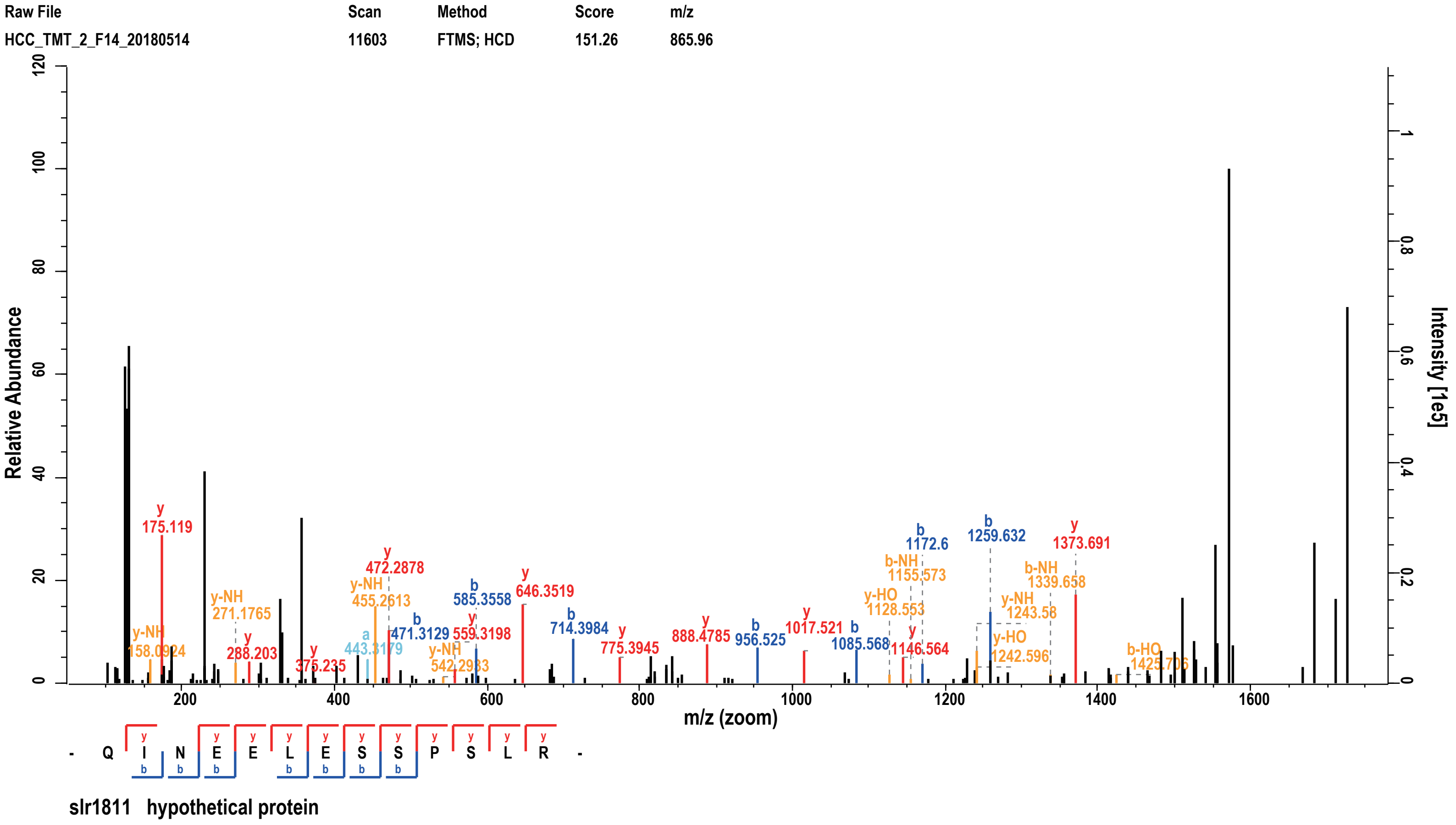
Raw File
HCC_TMT_2_F14_20180514
Scan
11603
Method
FTMS; HCD
Score
151.26
m/z
865.96
Relative Abundance
Intensity [1e5]
m/z (zoom)
y 175.119
y-NH 158.0924
y-NH 271.1765
y 288.203
y 375.235
a 443.3179
y-NH 455.2613
y 472.2878
b 471.3129
y-NH 542.2933
y 559.3198
b 585.3558
y 646.3519
b 714.3984
y 775.3945
y 888.4785
b 956.525
y 1017.521
b 1085.568
y-HO 1128.553
y 1146.564
b-NH 1155.573
b 1172.6
y-HO 1242.596
y-NH 1243.58
b 1259.632
b-NH 1339.658
y 1373.691
b-HO 1425.706
- Q I N E E L E S S P S L R -
slr1811 hypothetical protein

Raw File	Scan	Method	Score	m/z
HCC_TMT_1_F3_20180514	13972	FTMS; HCD	160.36	730.45

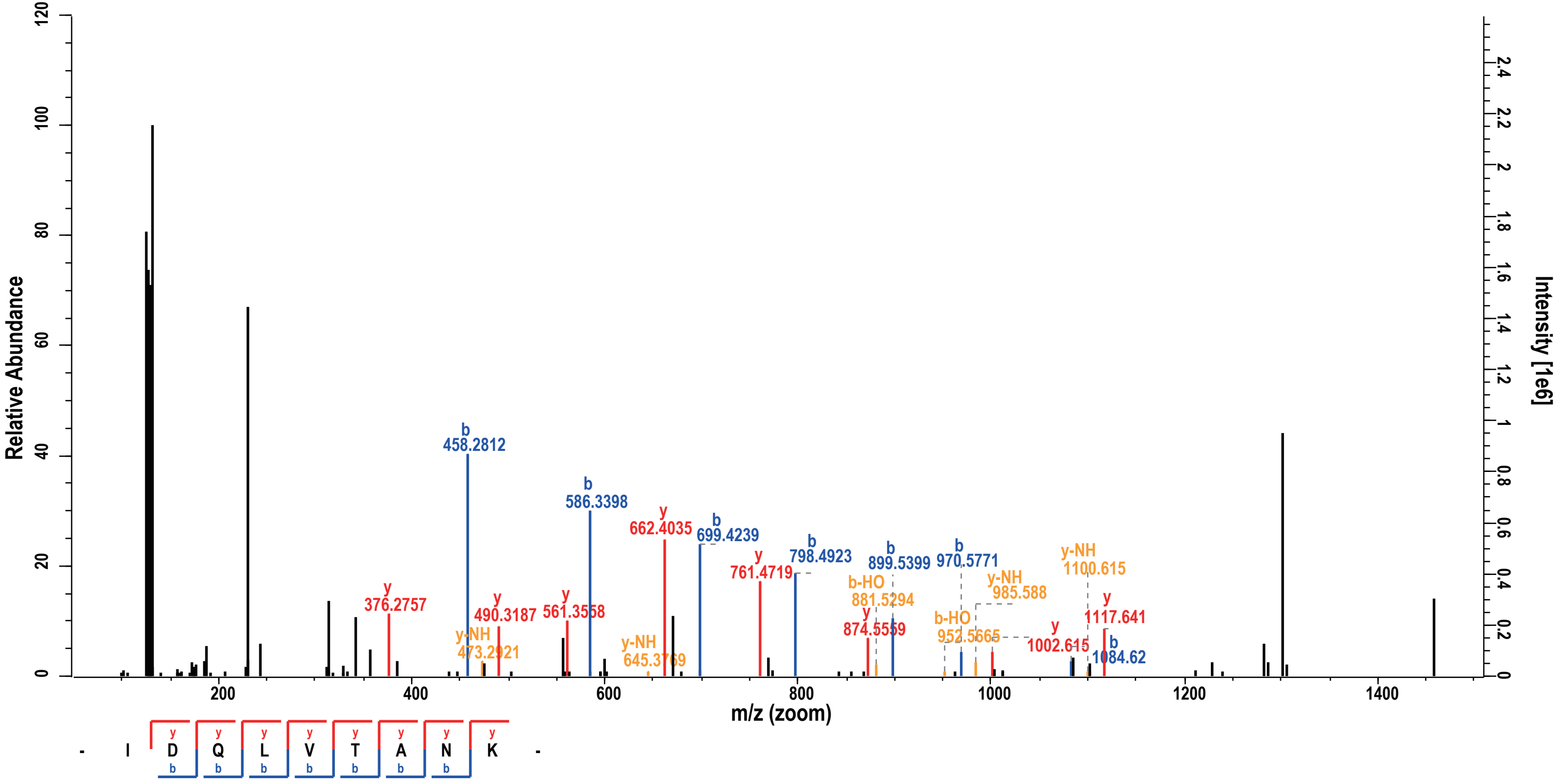

slr1846 hypothetical protein YCF64

Raw File	Scan	Method	Score	m/z
HCC_TMT_3_F9_20180514	16118	FTMS; HCD	113.22	981.6

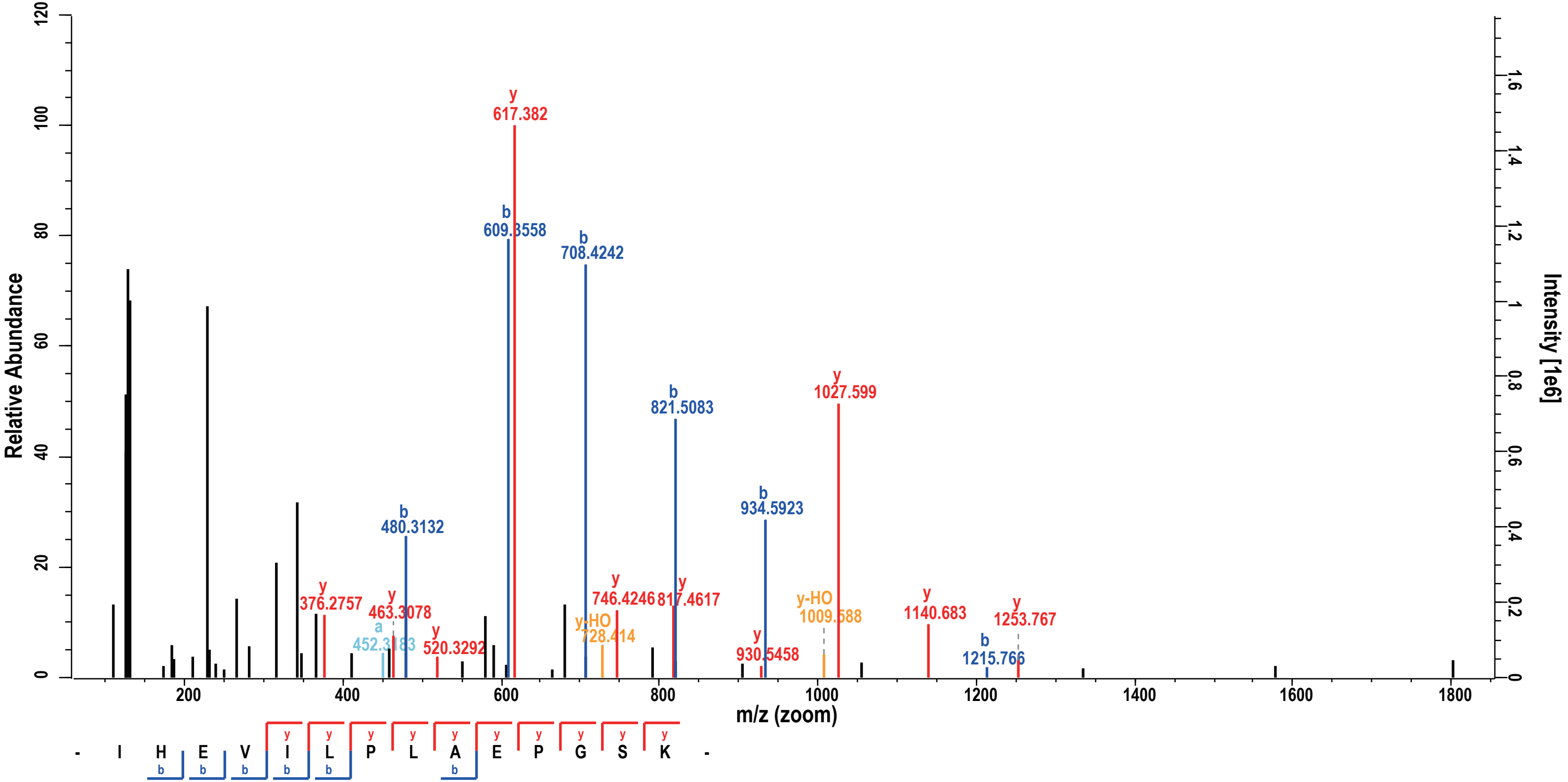

slr1856 phosphoprotein substrate of icfG gene cluster

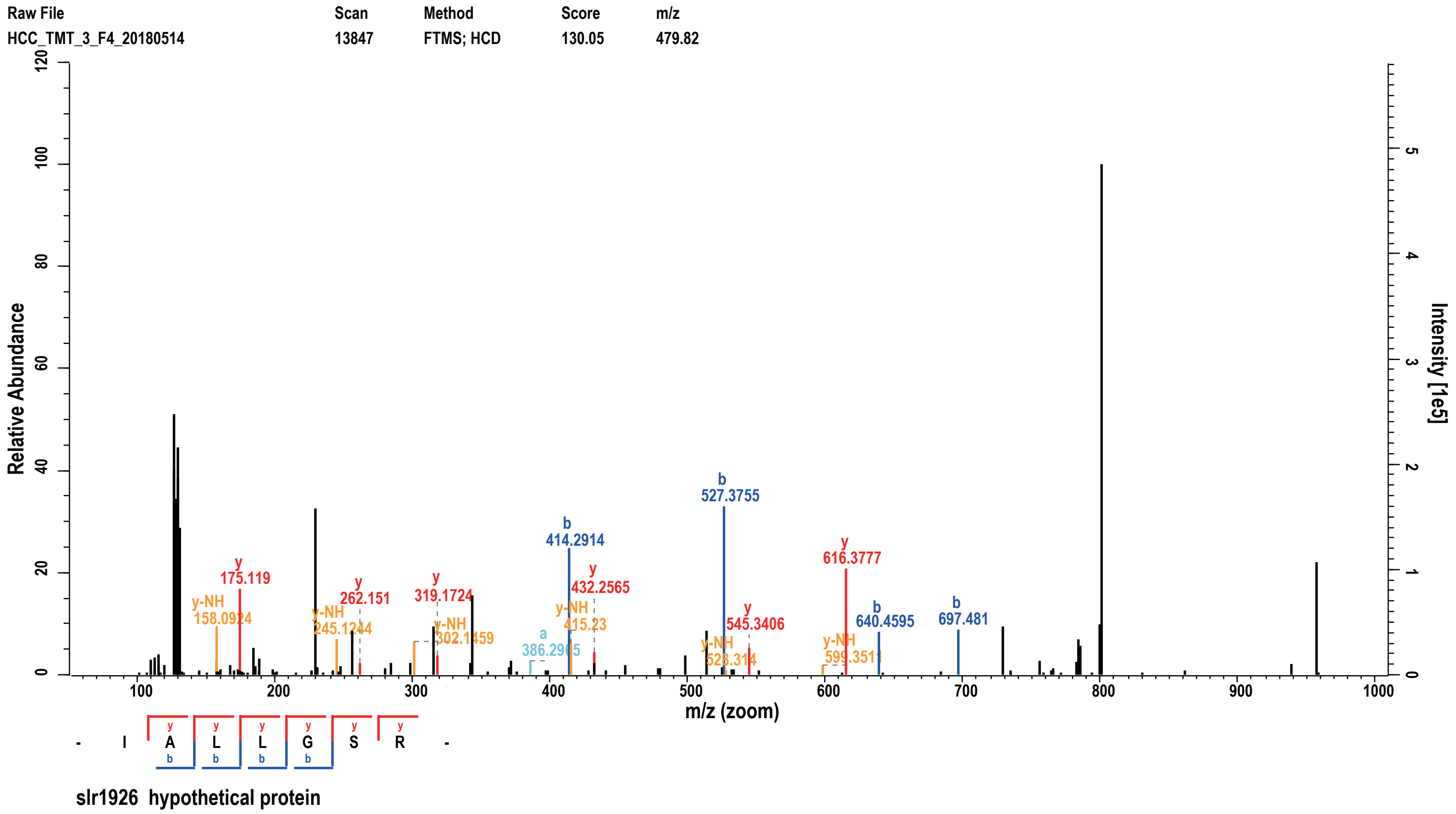
Raw File
HCC_TMT_3_F4_20180514
Scan
13847
Method
FTMS; HCD
Score
130.05
m/z
479.82
Relative Abundance
Intensity [1e5]
m/z (zoom)
y 175.119
y-NH 158.0924
y 262.151
y-NH 245.1244
y 319.1724
y-NH 302.1459
a 386.2965
b 414.2914
y-NH 415.23
y 432.2565
b 527.3755
y-NH 528.314
y 545.3406
y-NH 599.3511
y 616.3777
b 640.4595
b 697.481
- I A L L G S R -
slr1926 hypothetical protein

Raw File	Scan	Method	Score	m/z
HCC_TMT_3_F7_20180514	13497	FTMS; HCD	100.07	541.81

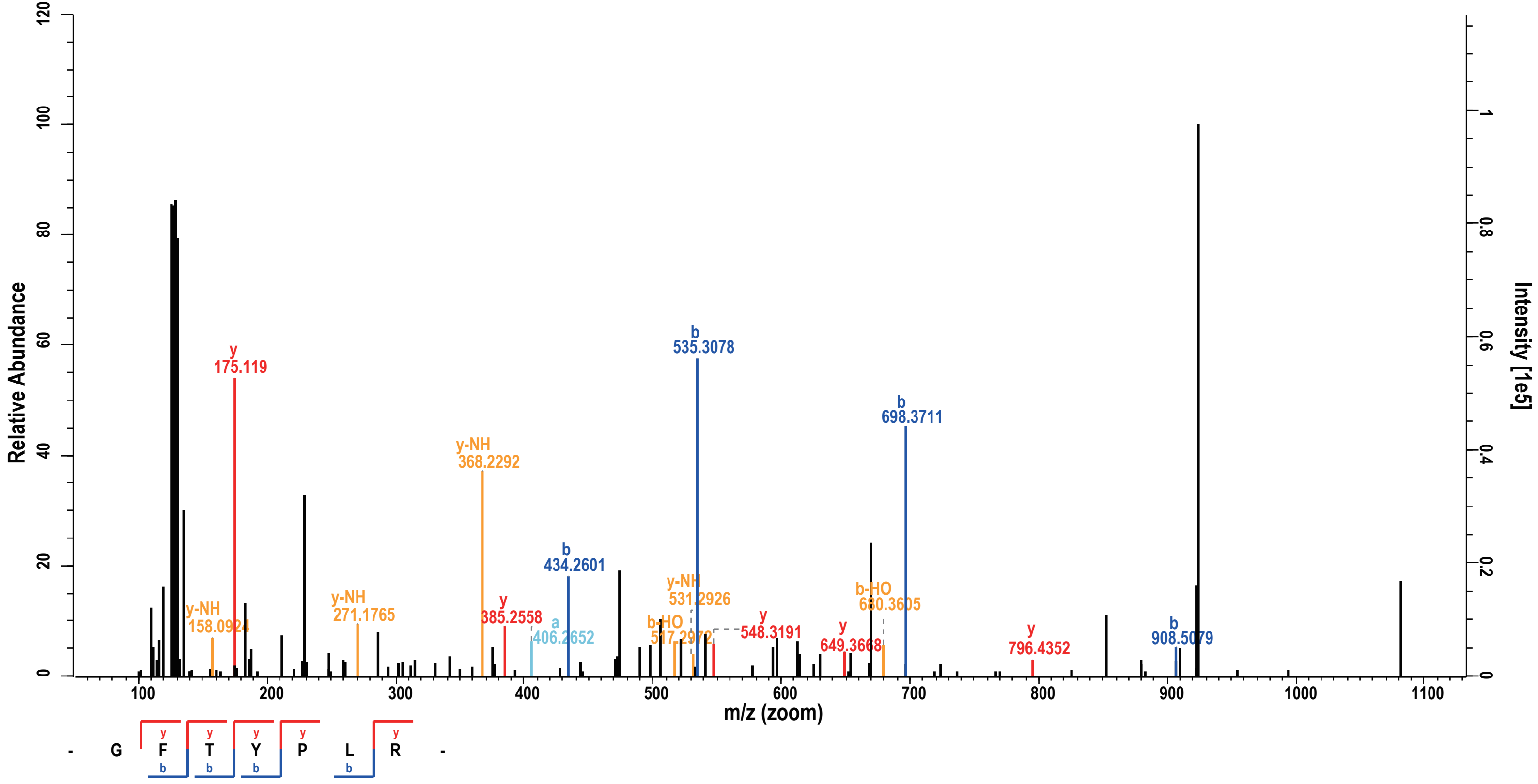

slr1980 hypothetical protein

Raw File	Scan	Method	Score	m/z
HCC_TMT_1_F10_20180514	14515	FTMS; HCD	132.8	520.35

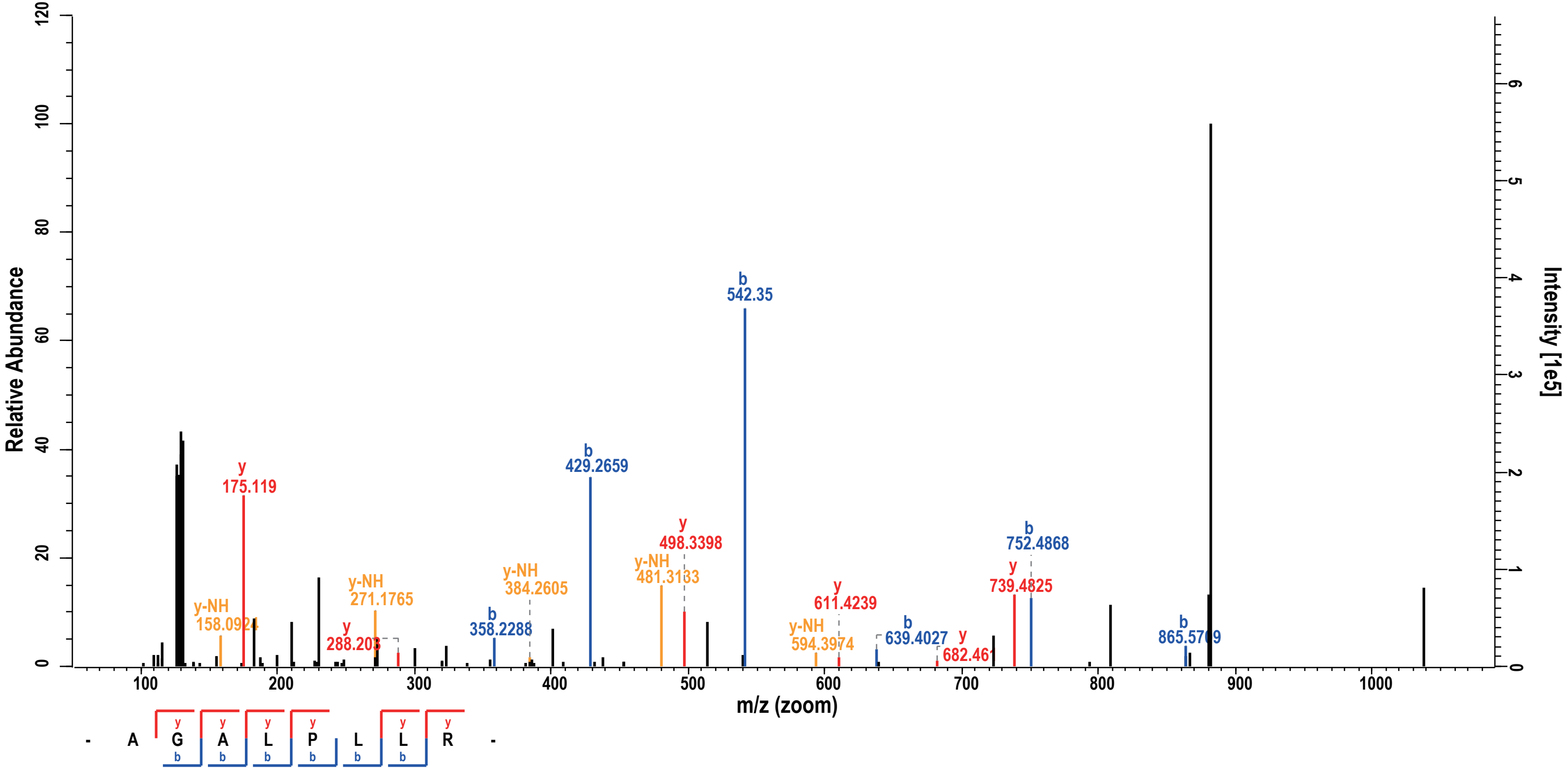

slr2007 NADH dehydrogenase subunit 4

Raw File	Scan	Method	Score	m/z
HCC_TMT_1_F5_20180514	12001	FTMS; HCD	117.89	753.46

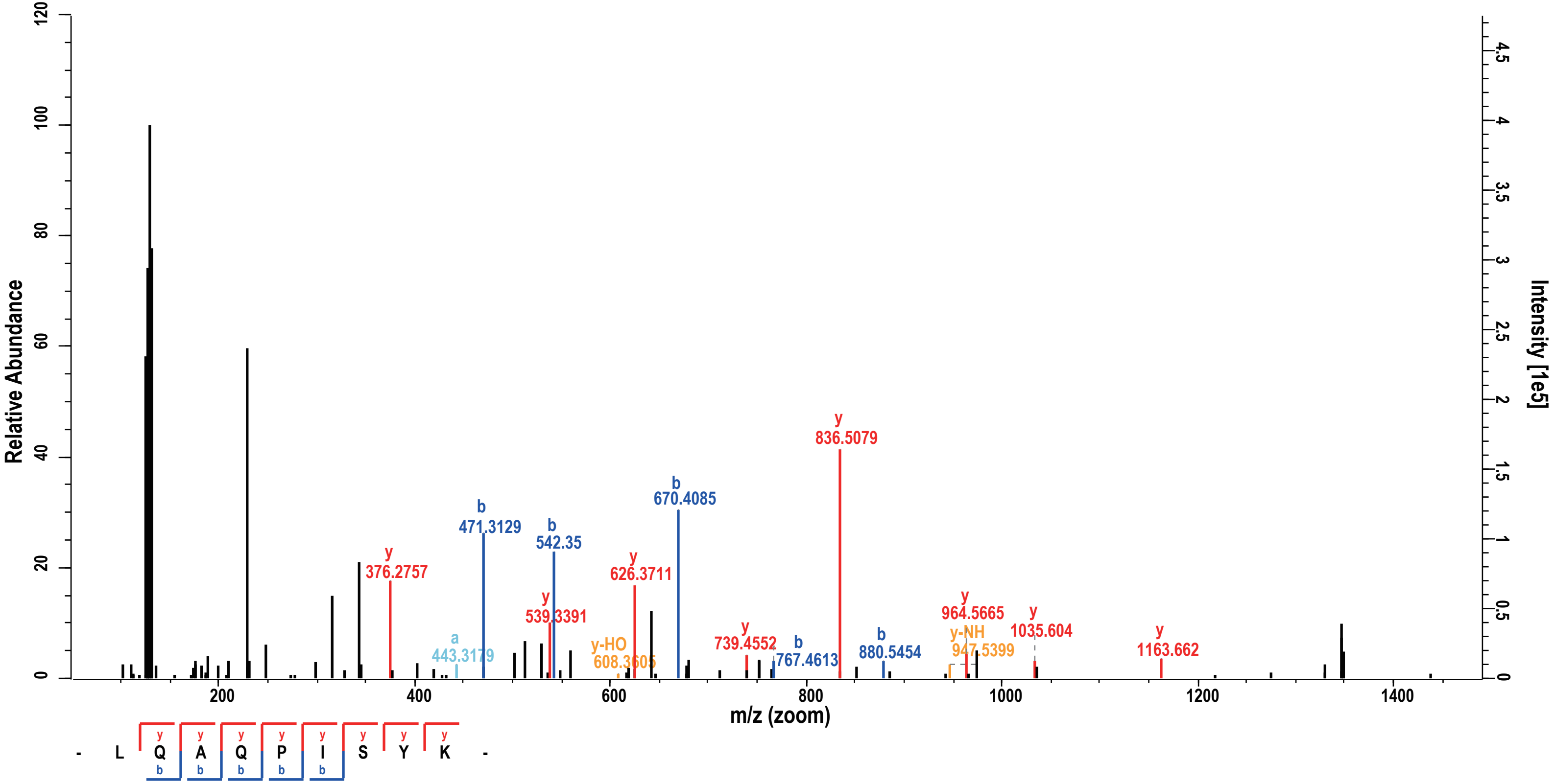

slr2009 NADH dehydrogenase subunit 4

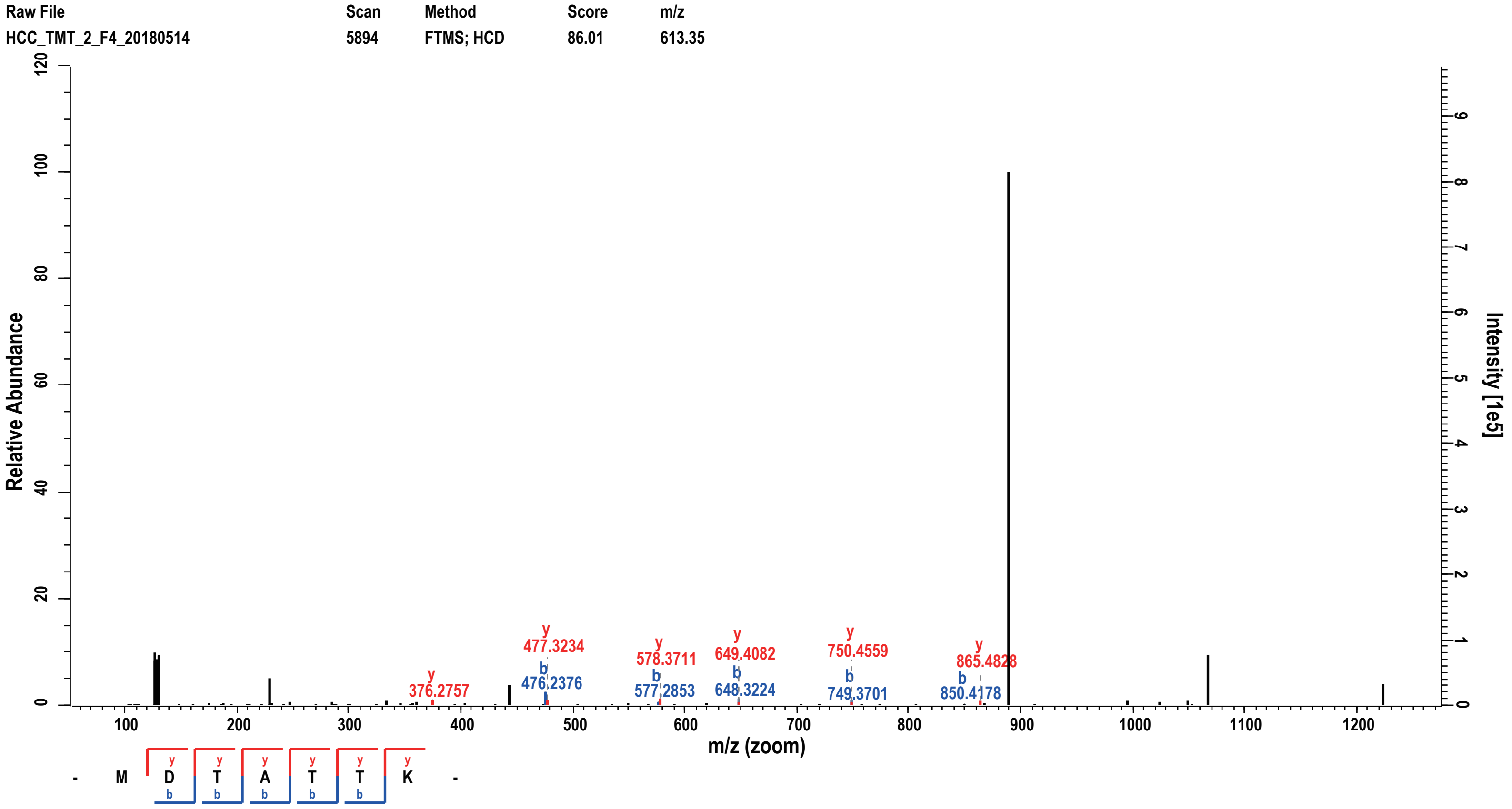

slr2099 two-component hybrid sensor and regulator

Raw File	Scan	Method	Score	m/z
HCC_TMT_3_F10_20180514	15490	FTMS; HCD	160.59	671.92

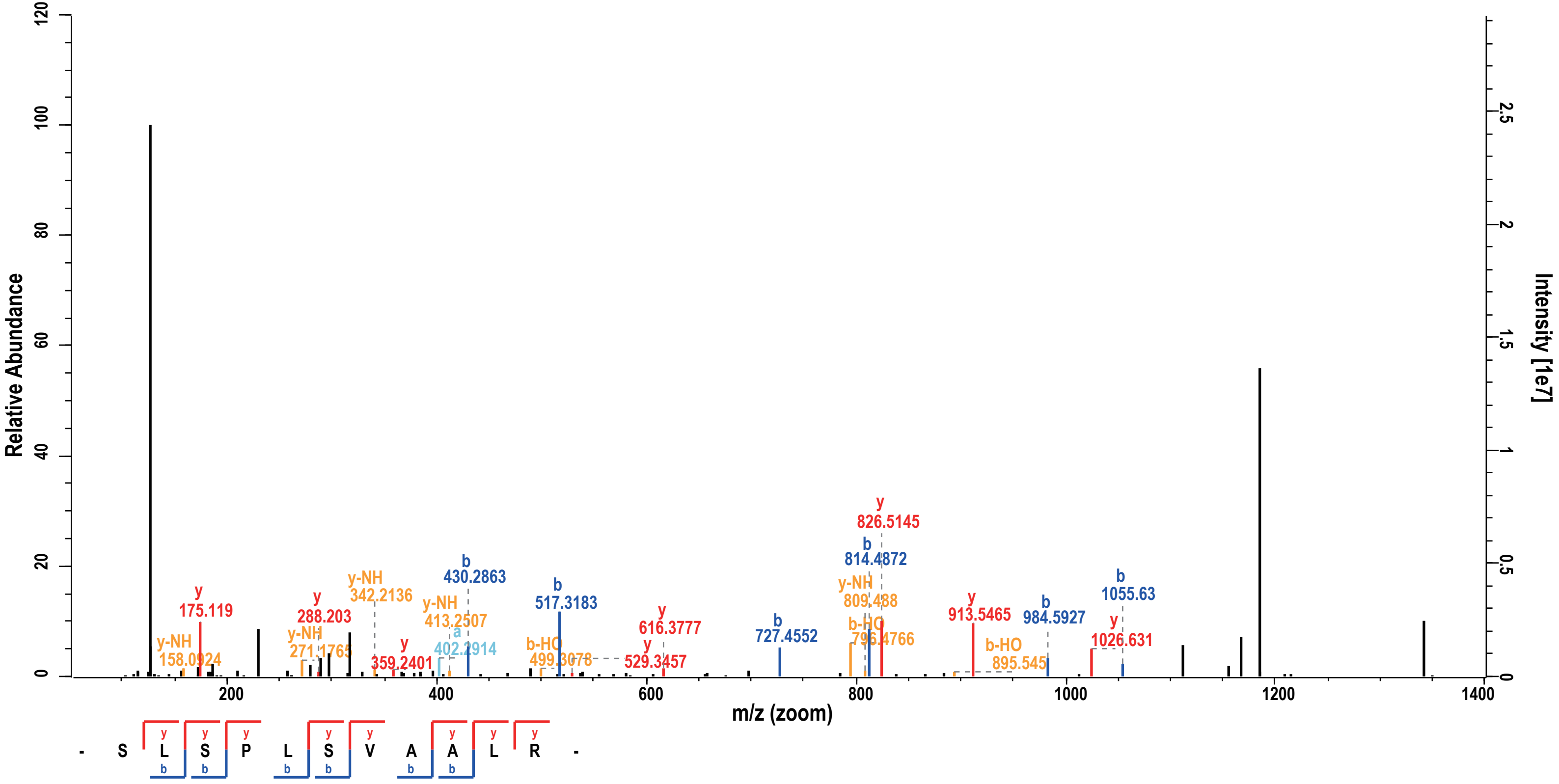

slr6081 unknown protein

Raw File	Scan	Method	Score	m/z
HCC_TMT_1_F15_20180514	14903	FTMS; HCD	96.16	724.08

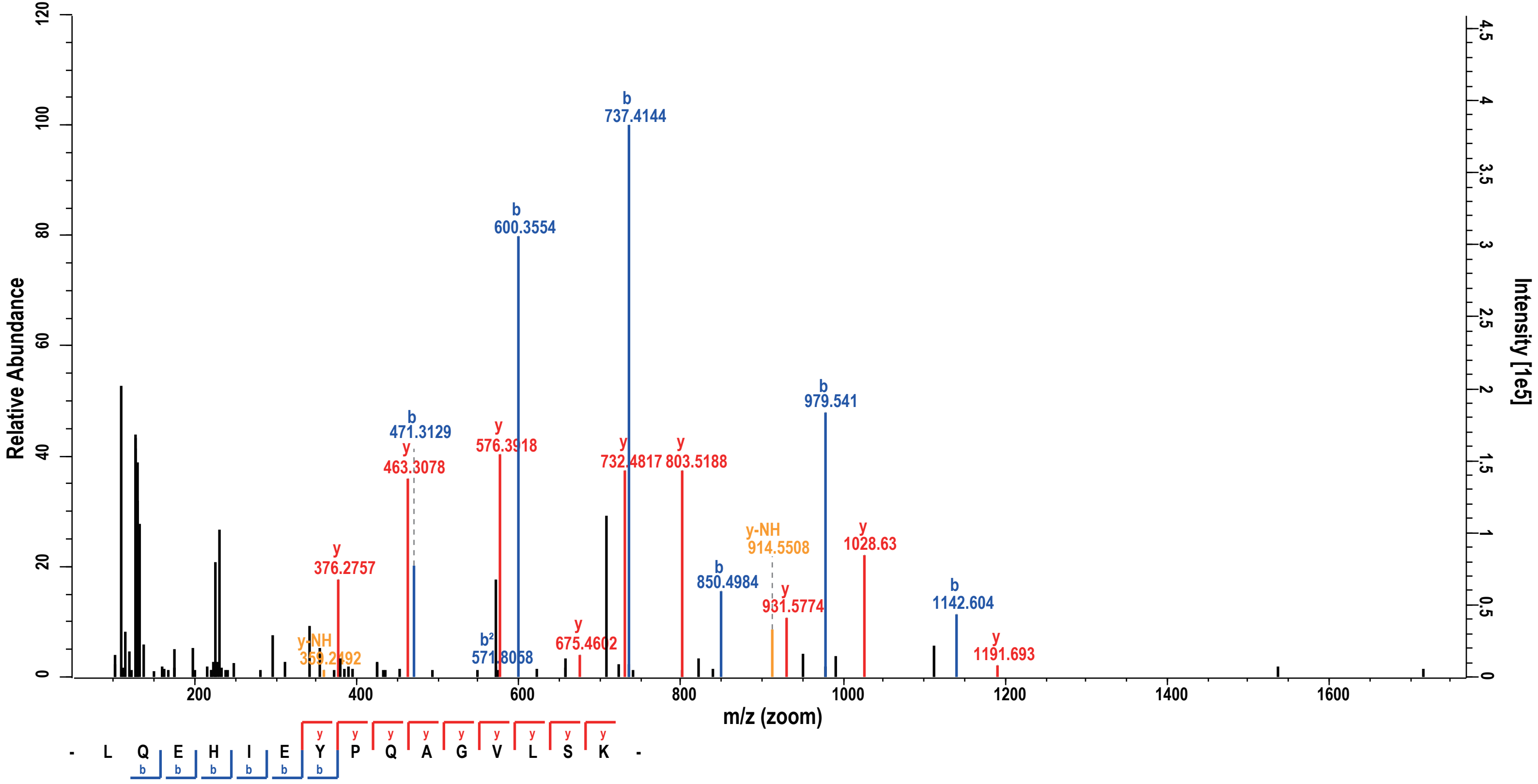

slr8022 hypothetical protein

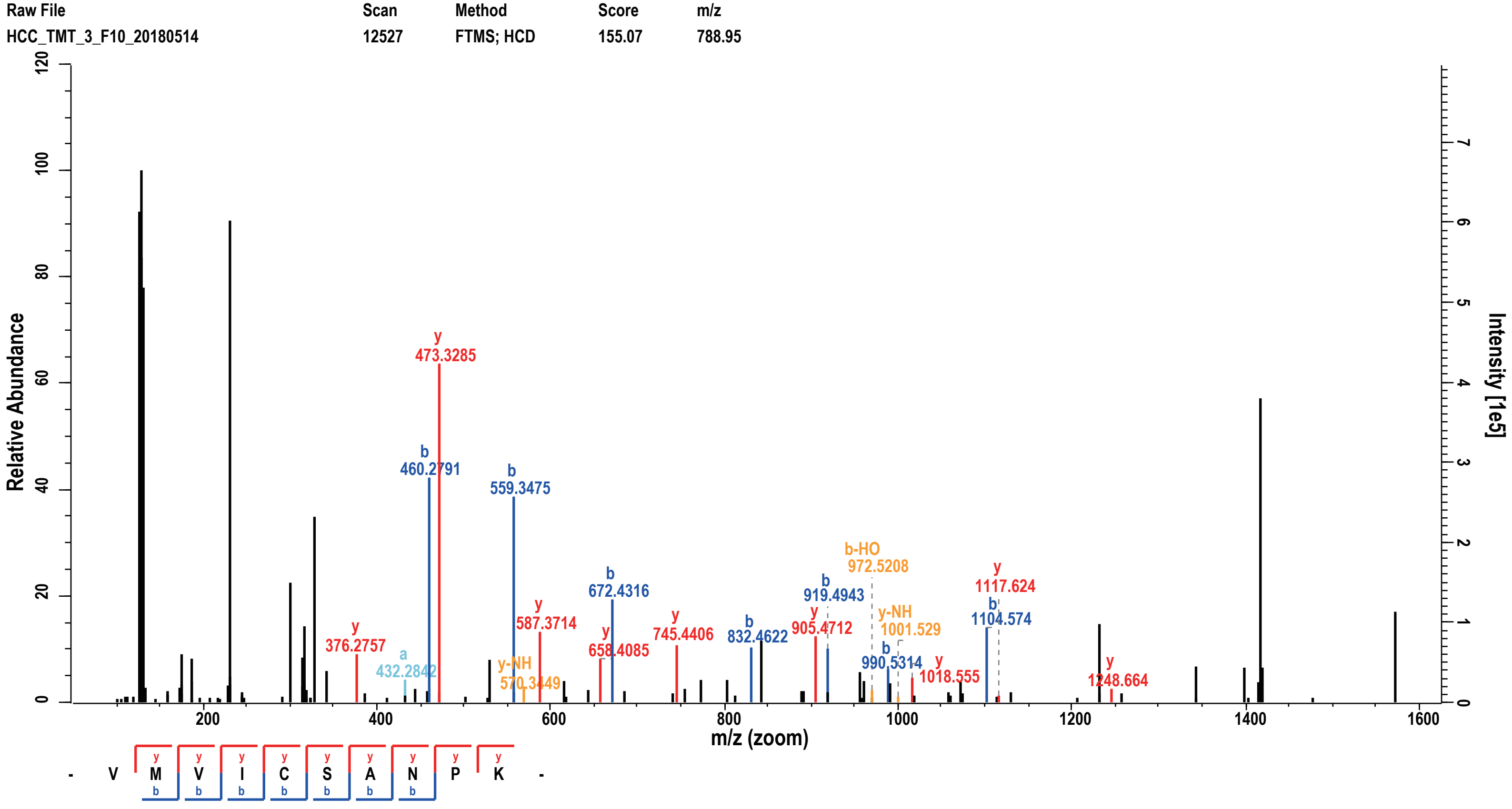

sml0006 50S ribosomal protein L36

Raw File	Scan	Method	Score	m/z
HCC_TMT_3_F12_20180514	11108	FTMS; HCD	139.97	579.35

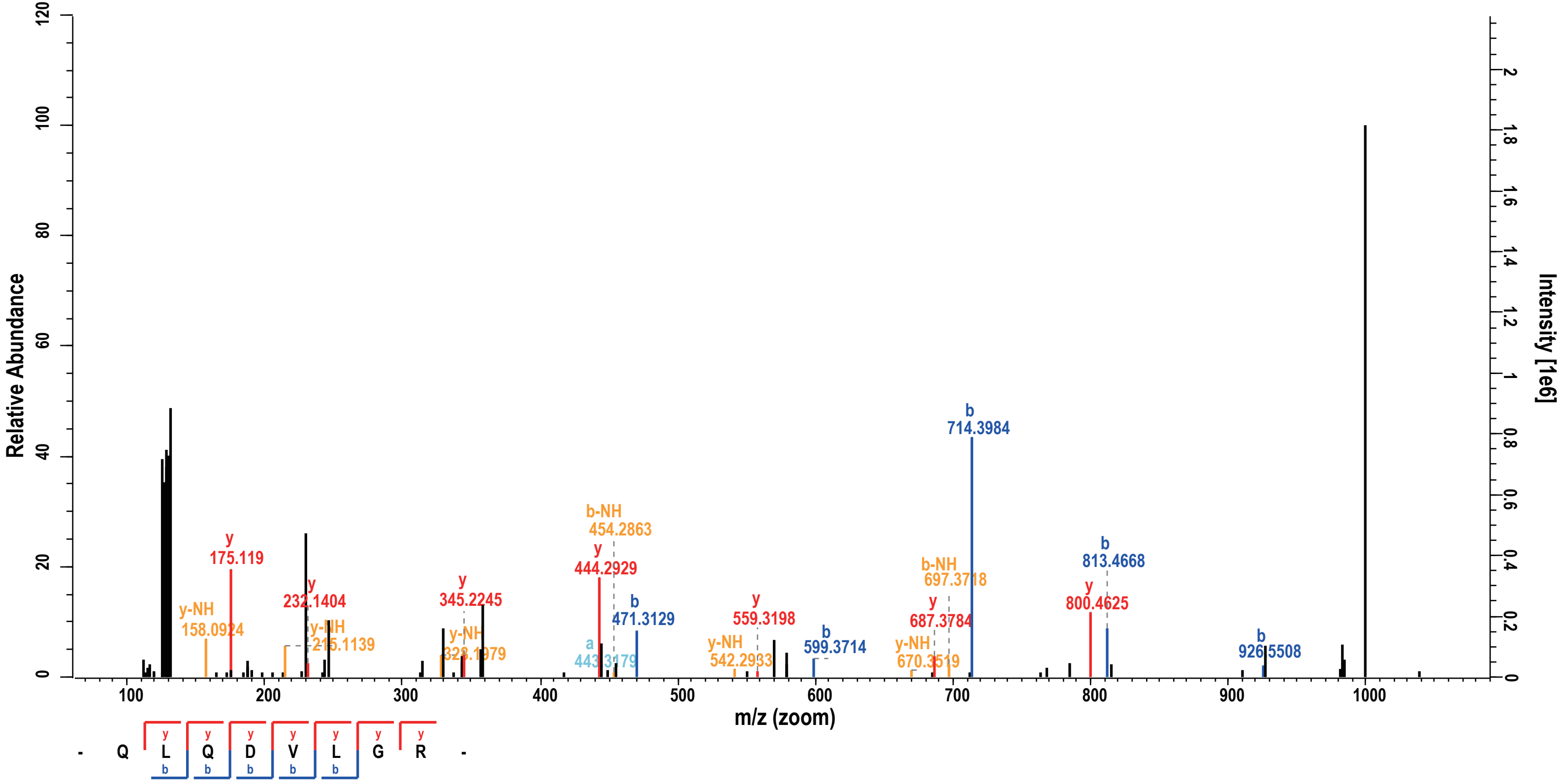

sml0007 photosystem II protein Y

Raw File	Scan	Method	Score	m/z
HCC_TMT_1_F3_20180514	17156	FTMS; HCD	72.23	689.37

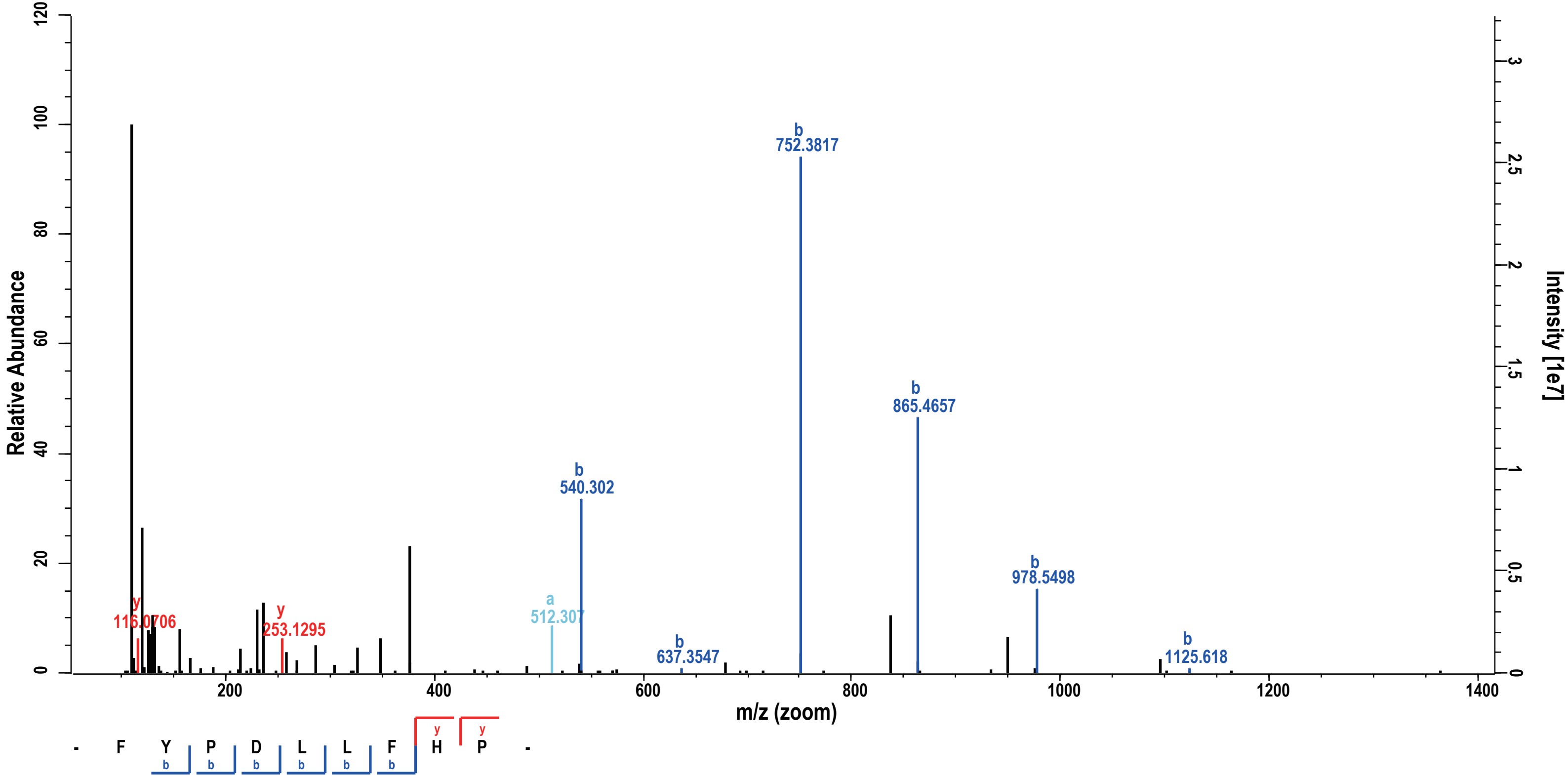

sml0008 photosystem I subunit IX

Raw File	Scan	Method	Score	m/z
HCC_TMT_2_F14_20180514	13118	FTMS; HCD	127.84	656.4

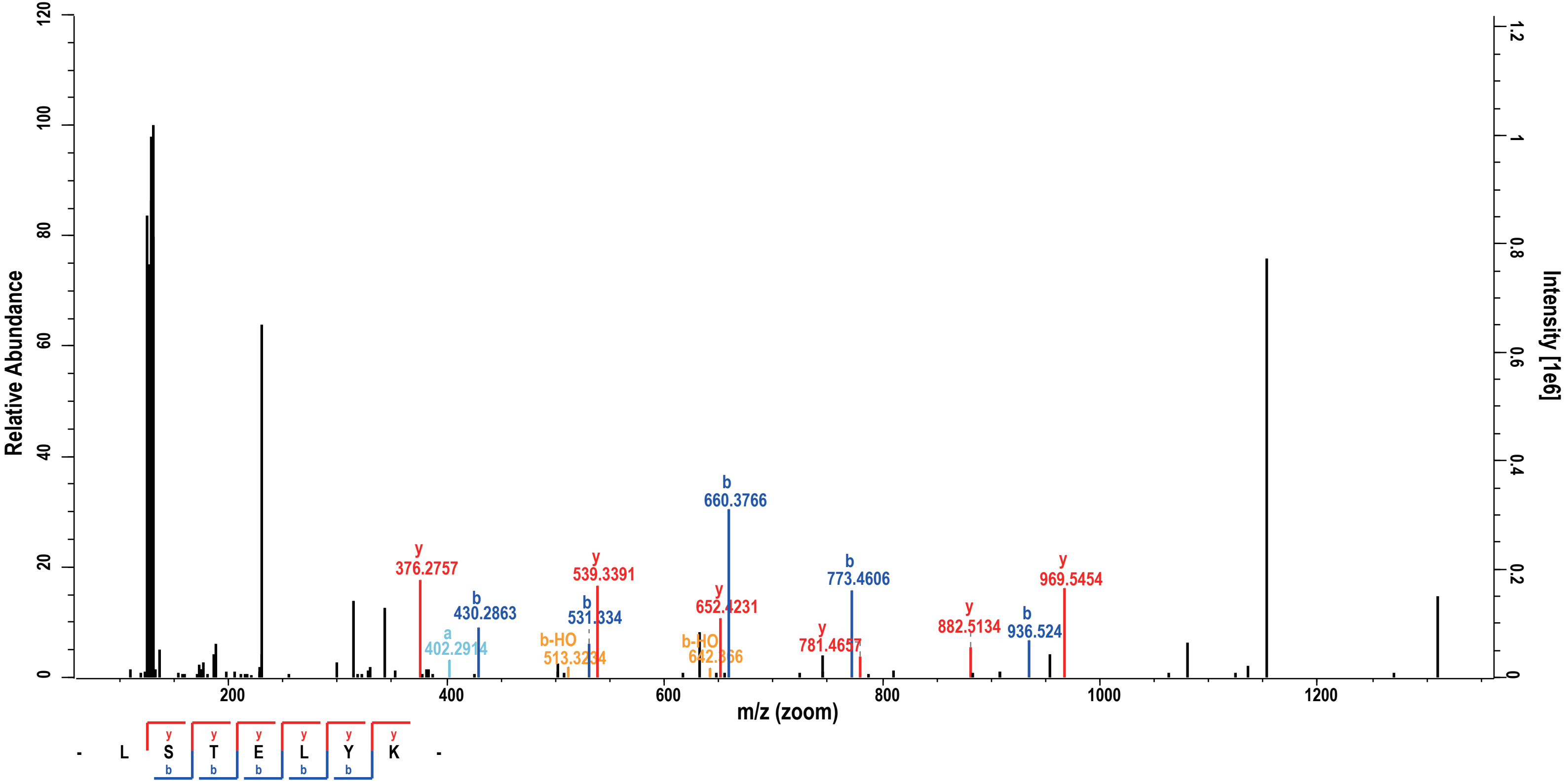

smr0005 photosystem I subunit XII

Raw File	Scan	Method	Score	m/z
HCC_TMT_2_F4_20180514	16076	FTMS; HCD	203.85	1088.09

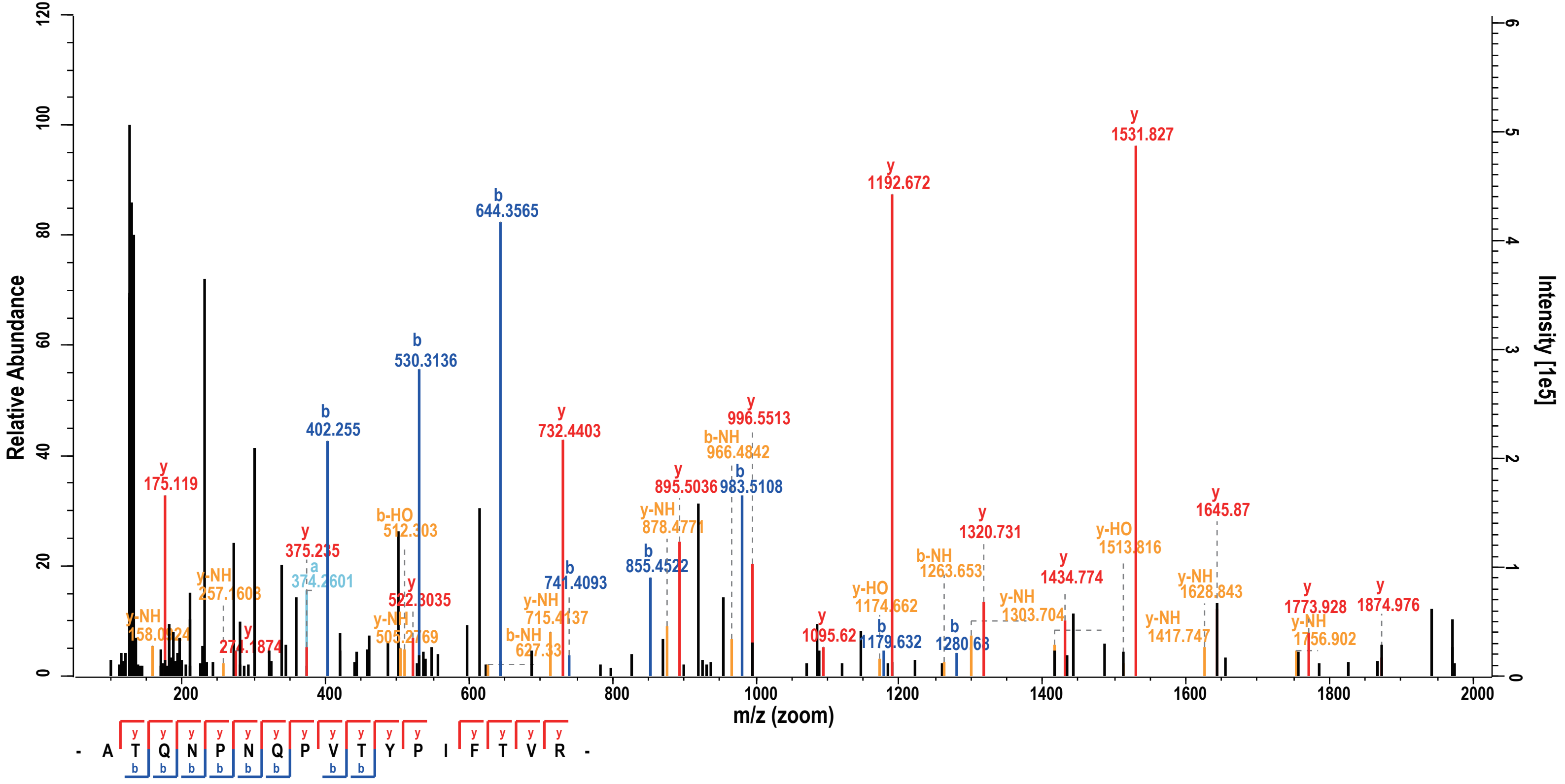

smr0006 cytochrome b559 b subunit

Raw File	Scan	Method	Score	m/z
HCC_TMT_2_F11_20180514	5972	FTMS; HCD	104.37	542.82

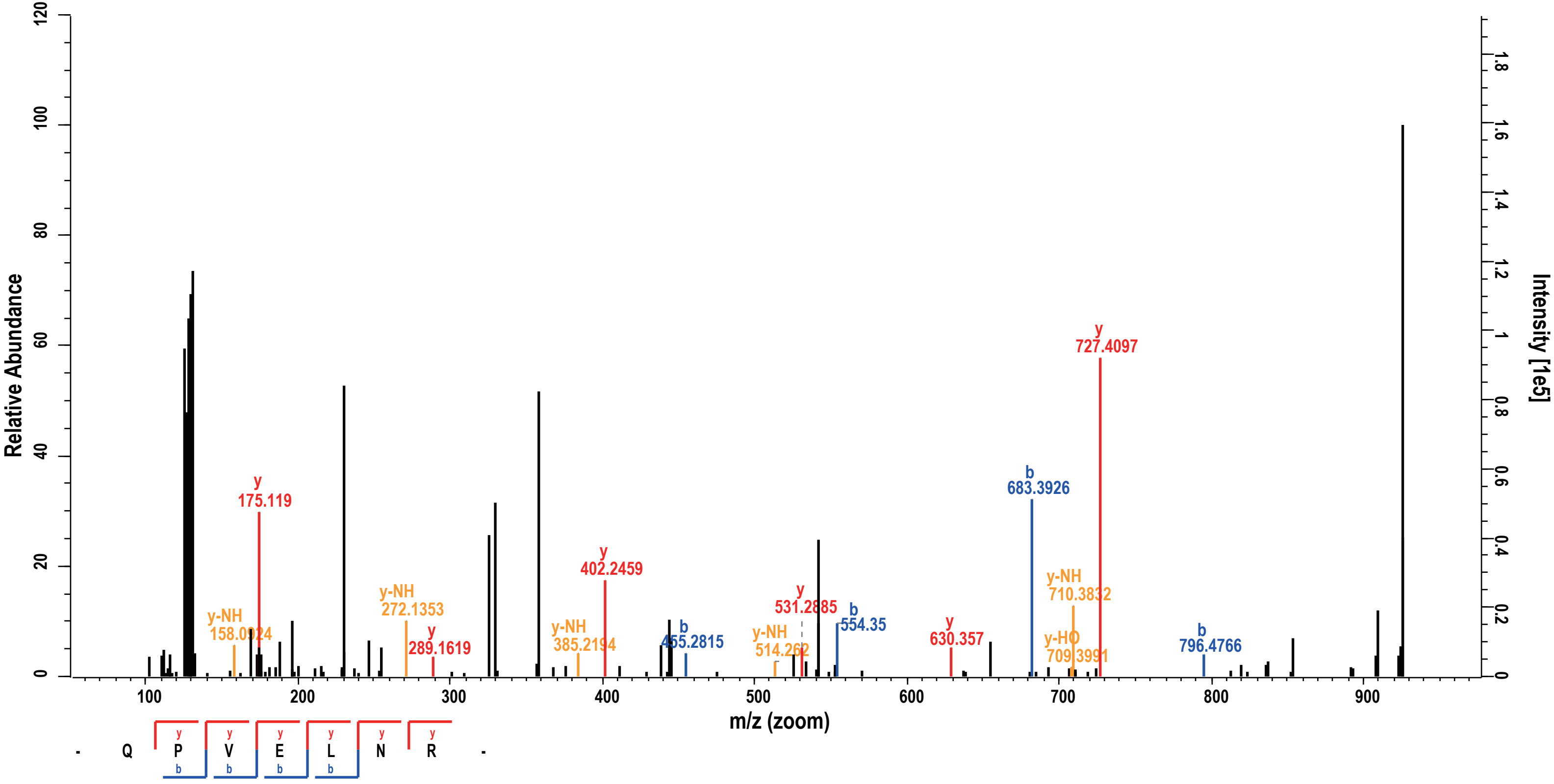

smr0007 photosystem II PsbL protein

Raw File	Scan	Method	Score	m/z
HCC_TMT_1_F7_20180514	7506	FTMS; HCD	90.66	741.42

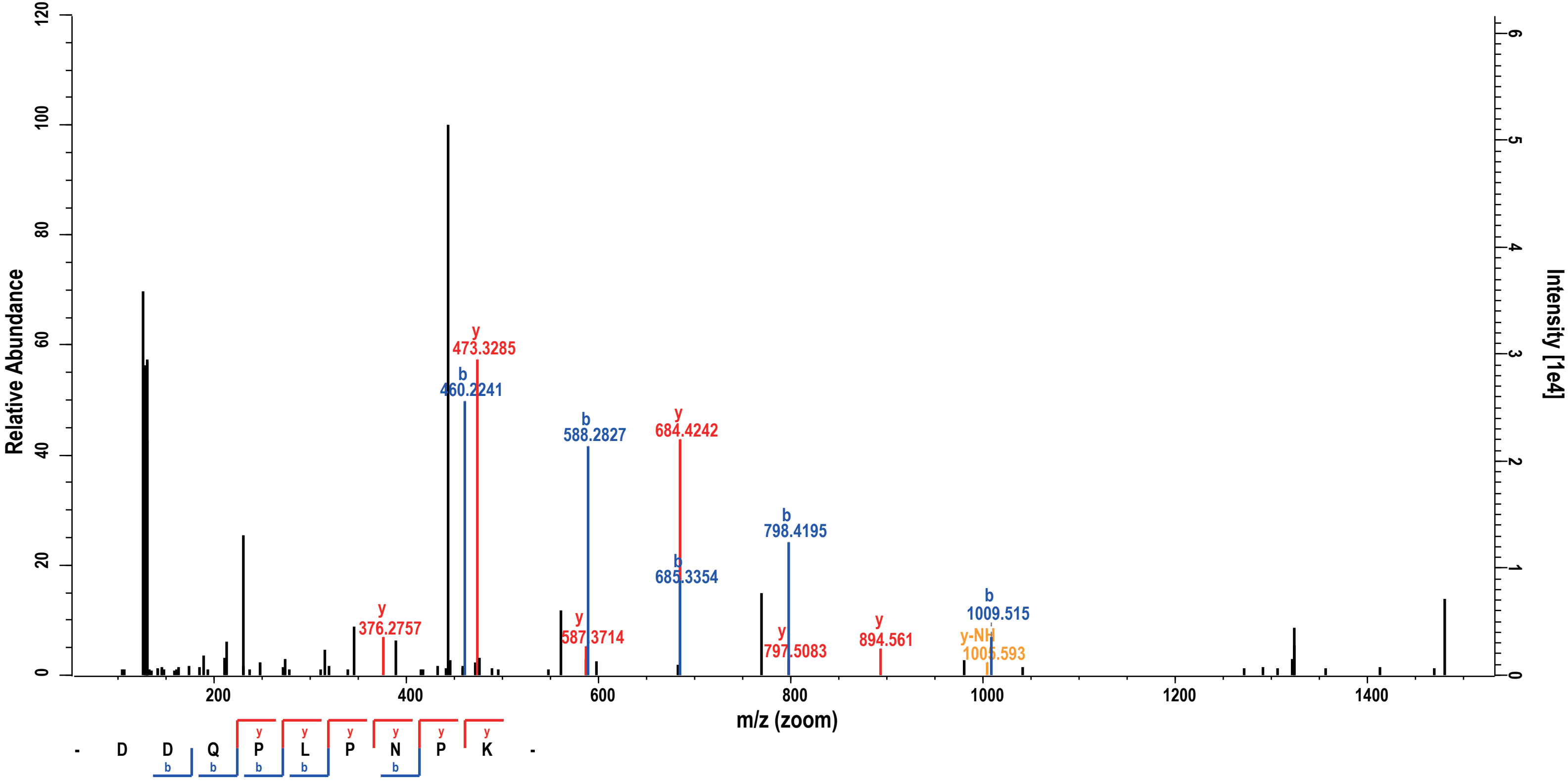

ssl0350 unknown protein

Raw File	Scan	Method	Score	m/z
HCC_TMT_1_F4_20180514	10913	FTMS; HCD	184.03	895.49

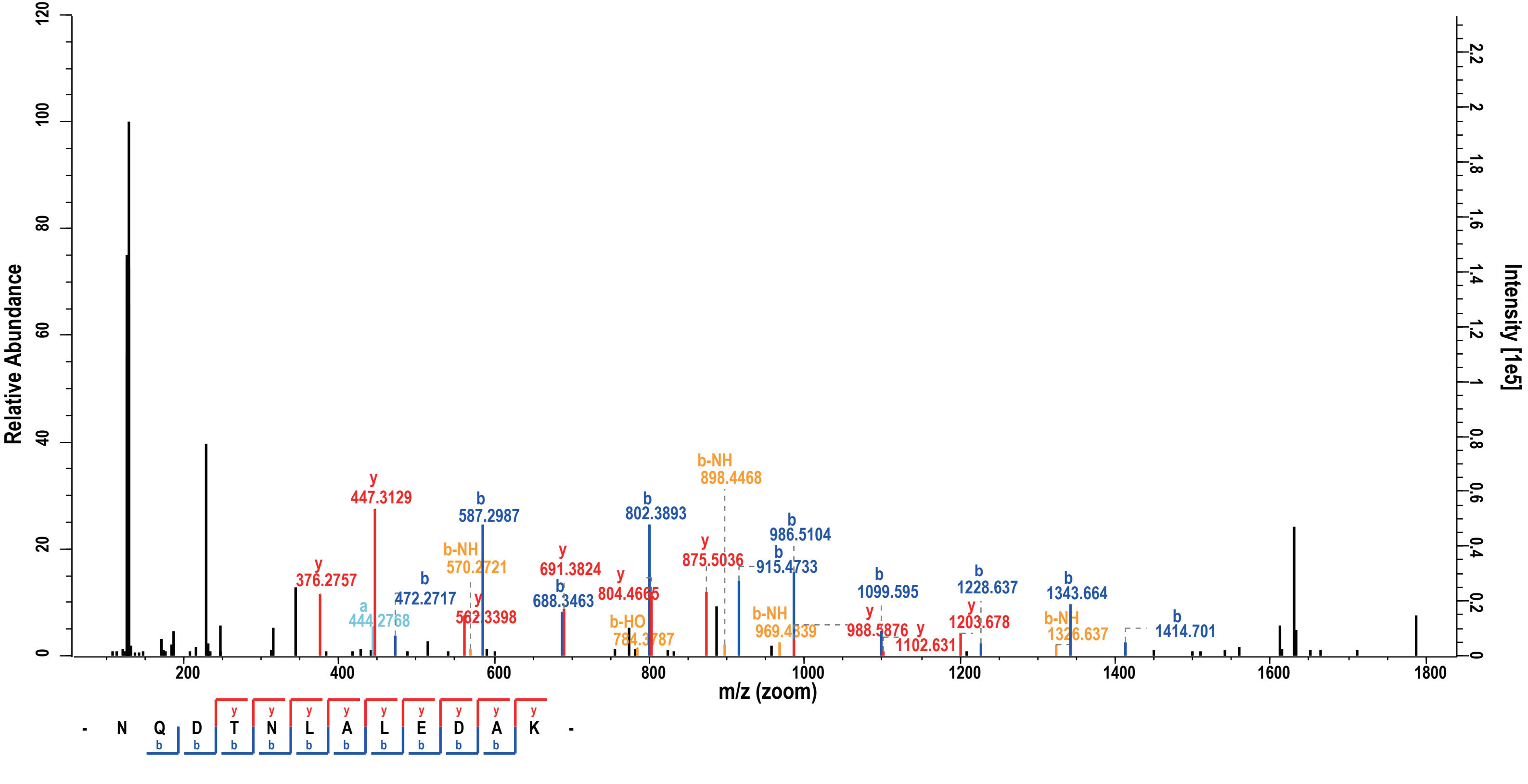

ssl0483 hypothetical protein

Raw File	Scan	Method	Score	m/z
HCC_TMT_1_F13_20180514	7464	FTMS; HCD	106.42	667.85

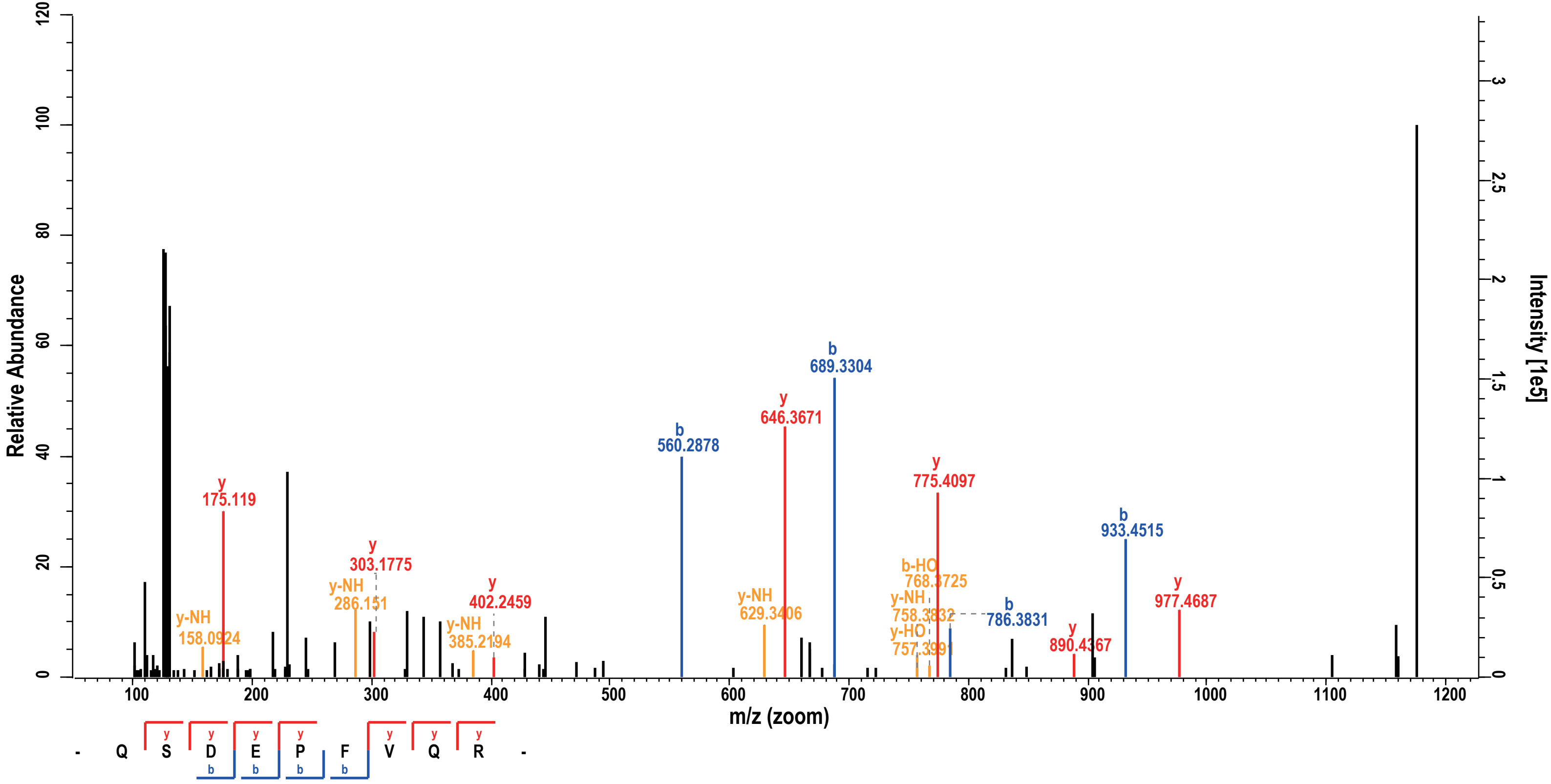

ssl0788 hypothetical protein

Raw File	Scan	Method	Score	m/z
HCC_TMT_1_F14_20180514	2009	FTMS; HCD	97.07	562.27

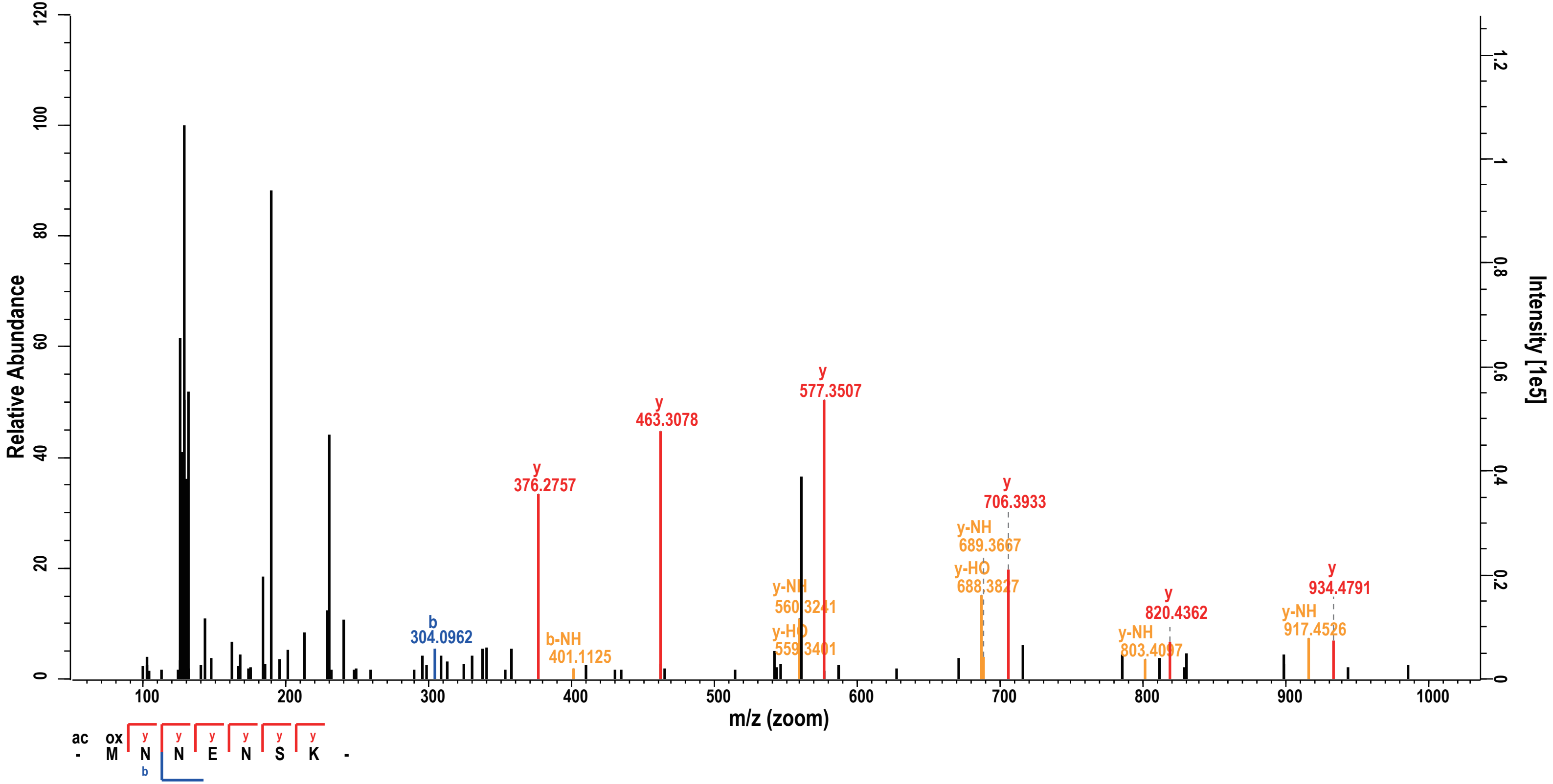

ssl1633 high light-inducible polypeptide HliC, CAB/ELIP/HLIP superfamily

Raw File	Scan	Method	Score	m/z
HCC_TMT_1_F13_20180514	13754	FTMS; HCD	85.53	490.31

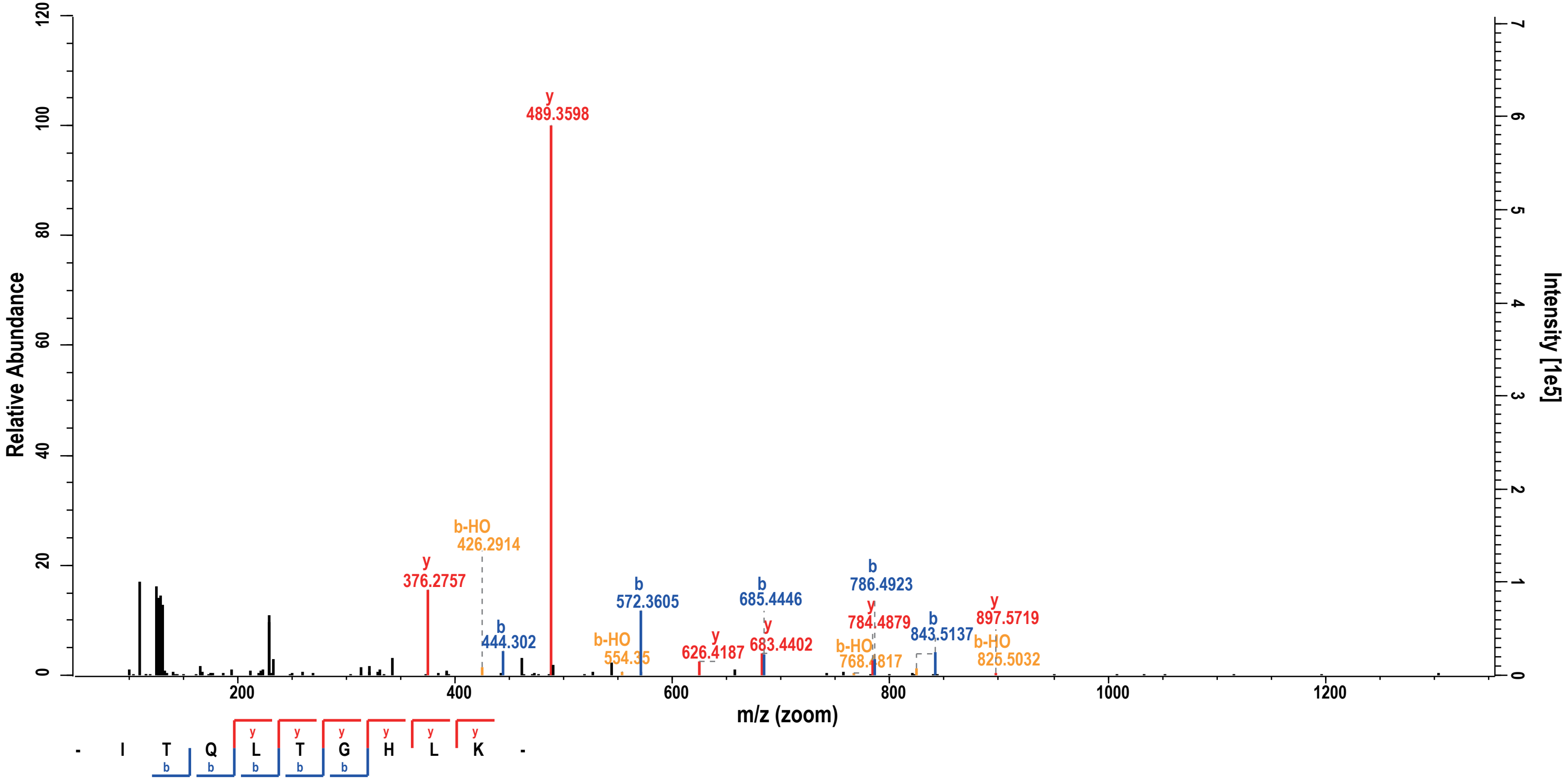

ssl1784 30S ribosomal protein S15

Raw File	Scan	Method	Score	m/z
HCC_TMT_3_F13_20180514	11085	FTMS; HCD	117.16	603.38

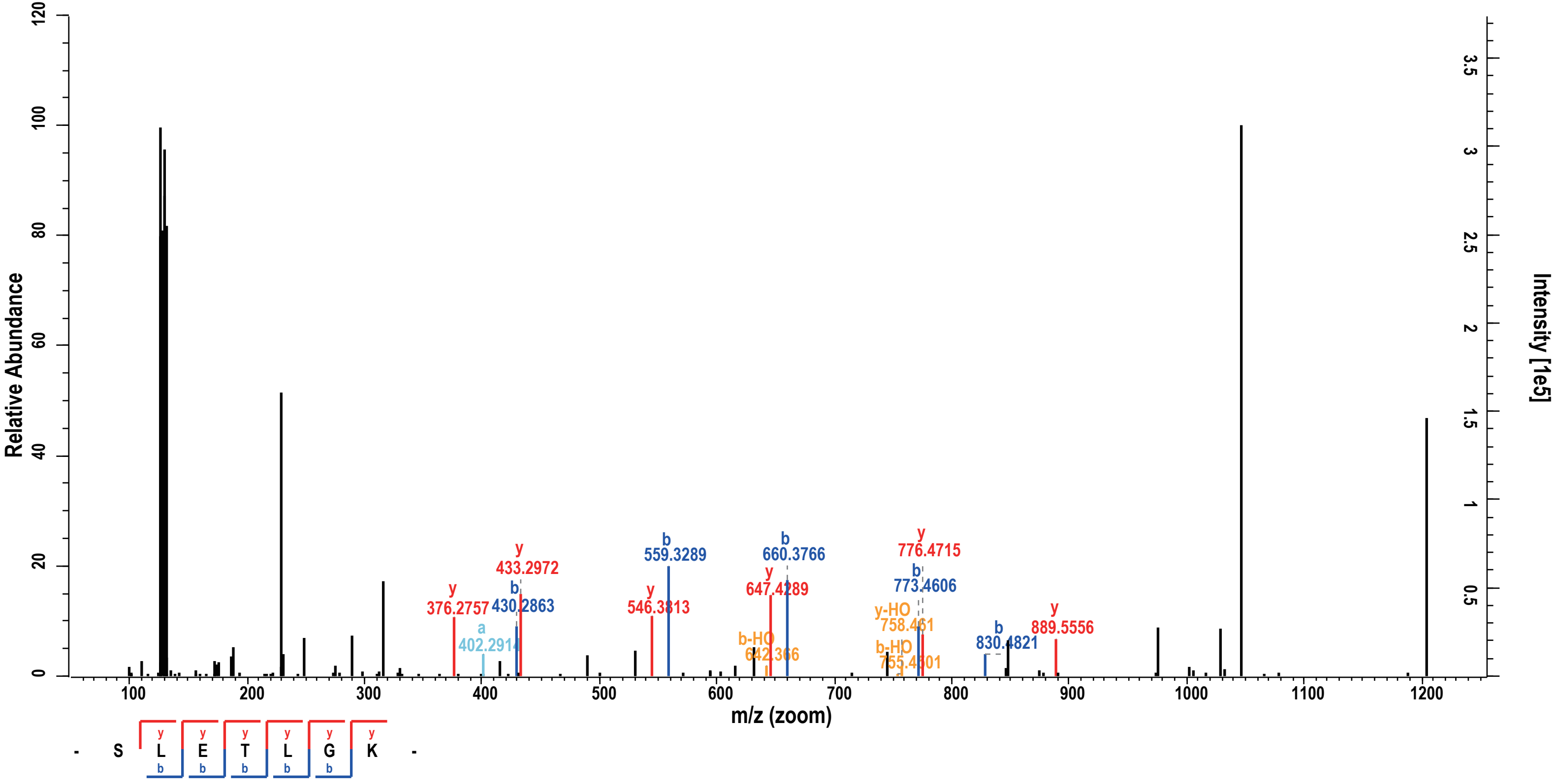

ssl2065 unknown protein

Raw File	Scan	Method	Score	m/z
HCC_TMT_1_F15_20180514	3872	FTMS; HCD	106.36	608.85

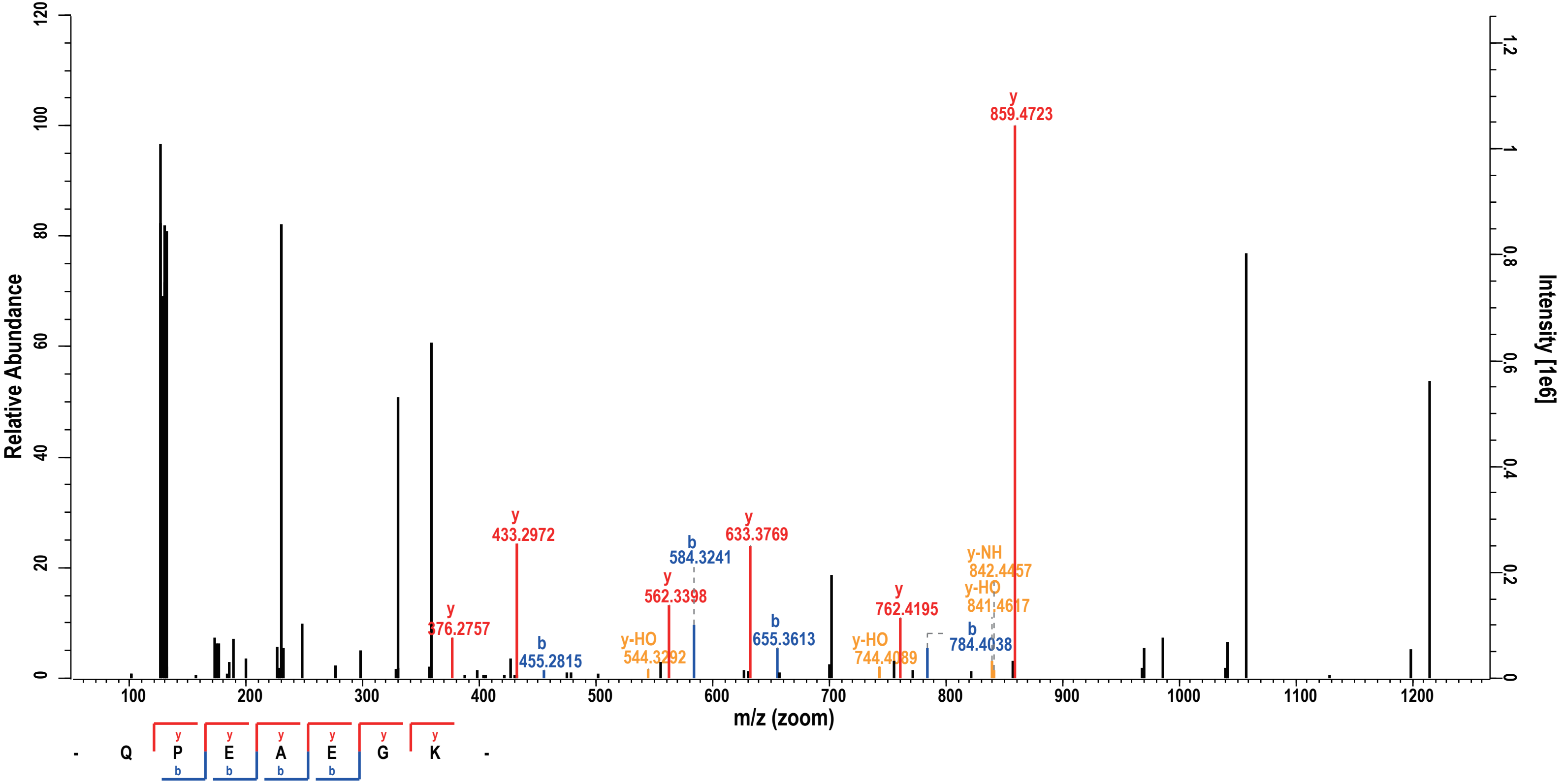

ssl2615 ATP synthase C chain of CF(0)

Raw File	Scan	Method	Score	m/z
HCC_TMT_1_F9_20180514	9052	FTMS; HCD	125.75	760.94

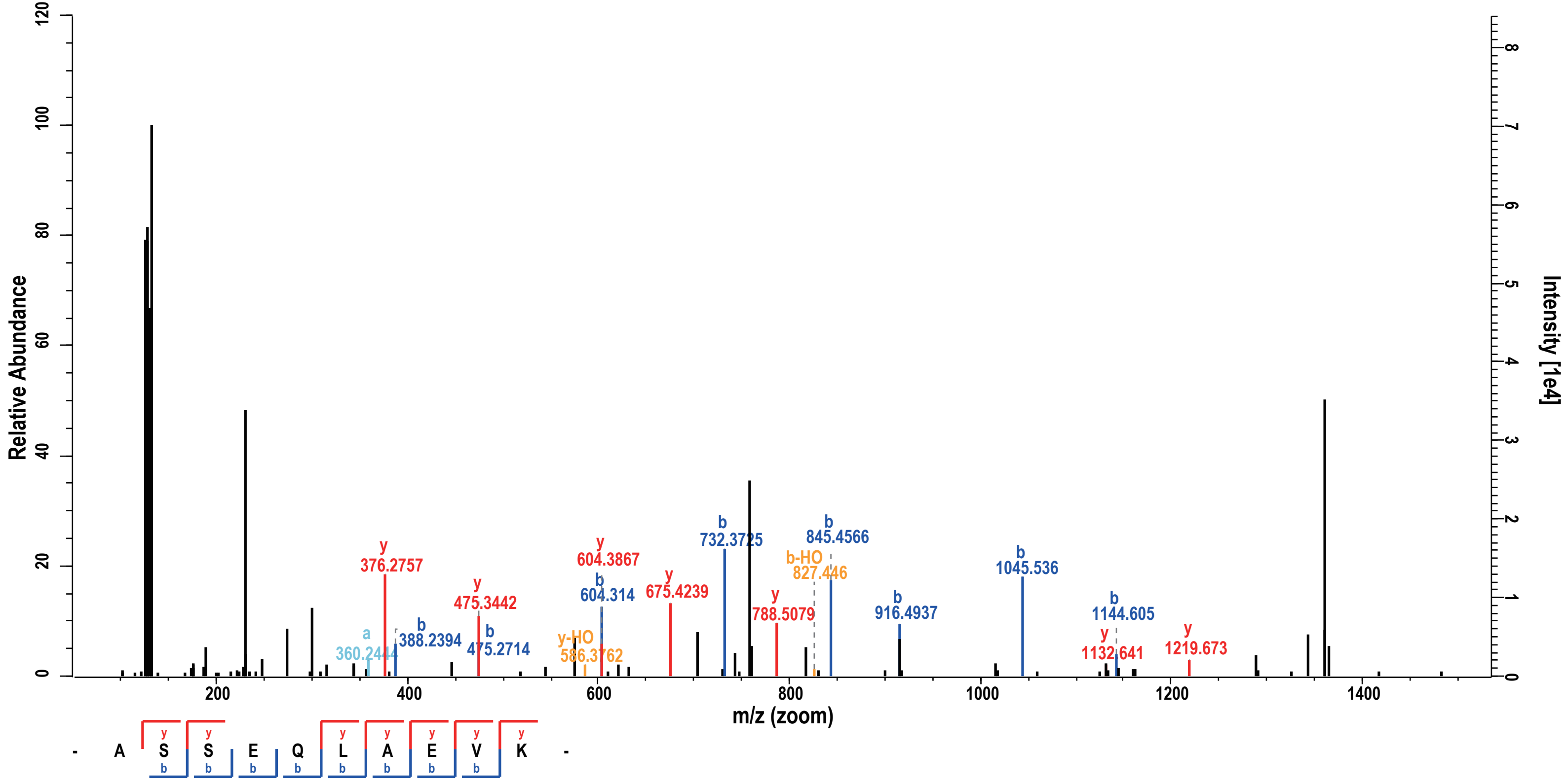

ssl2648 hypothetical protein

Raw File	Scan	Method	Score	m/z
HCC_TMT_3_F5_20180514	16196	FTMS; HCD	126.05	571.32

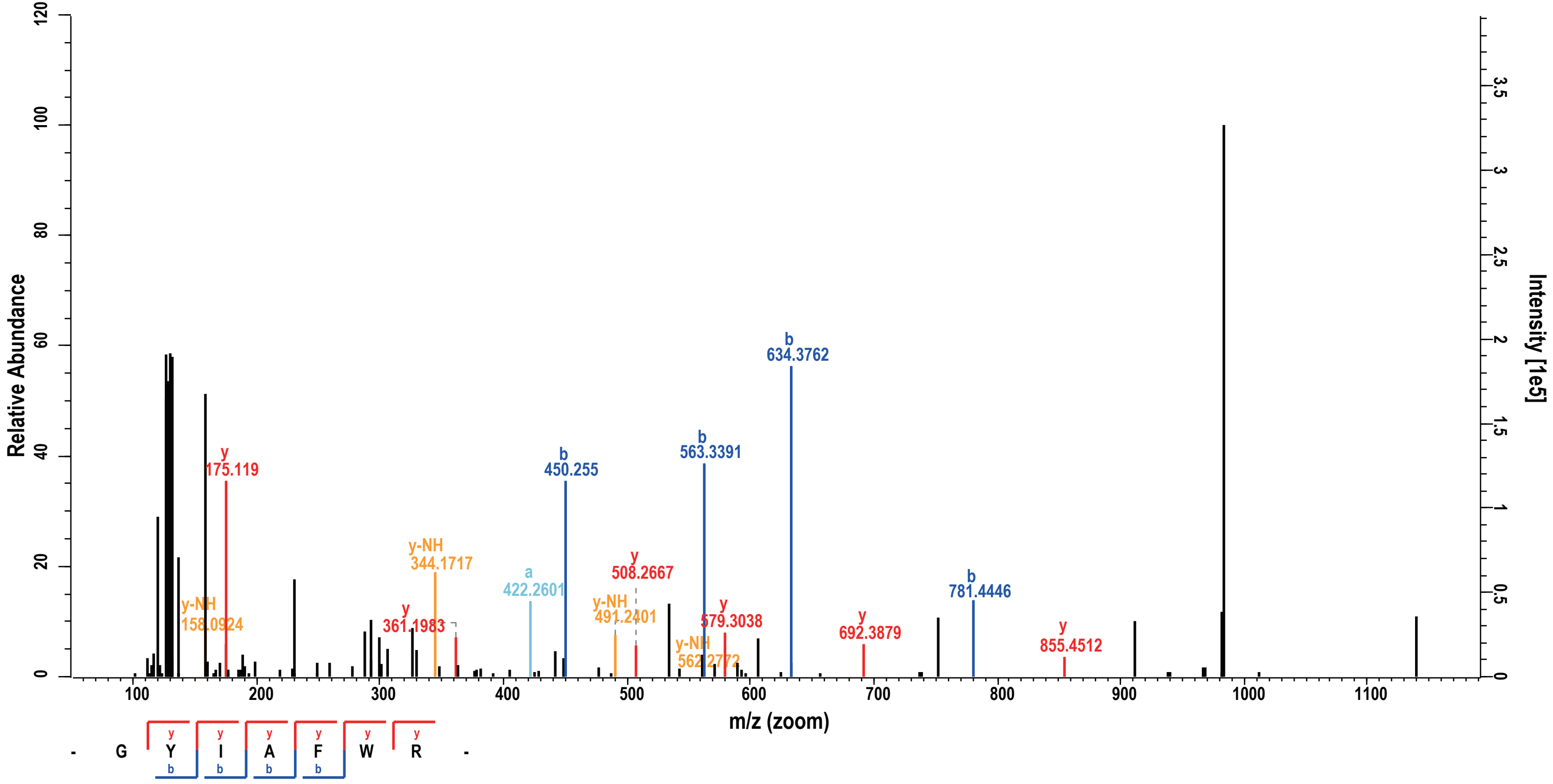

ssl3291 hypothetical protein

Raw File	Scan	Method	Score	m/z
HCC_TMT_1_F8_20180514	15574	FTMS; HCD	142.43	818.98

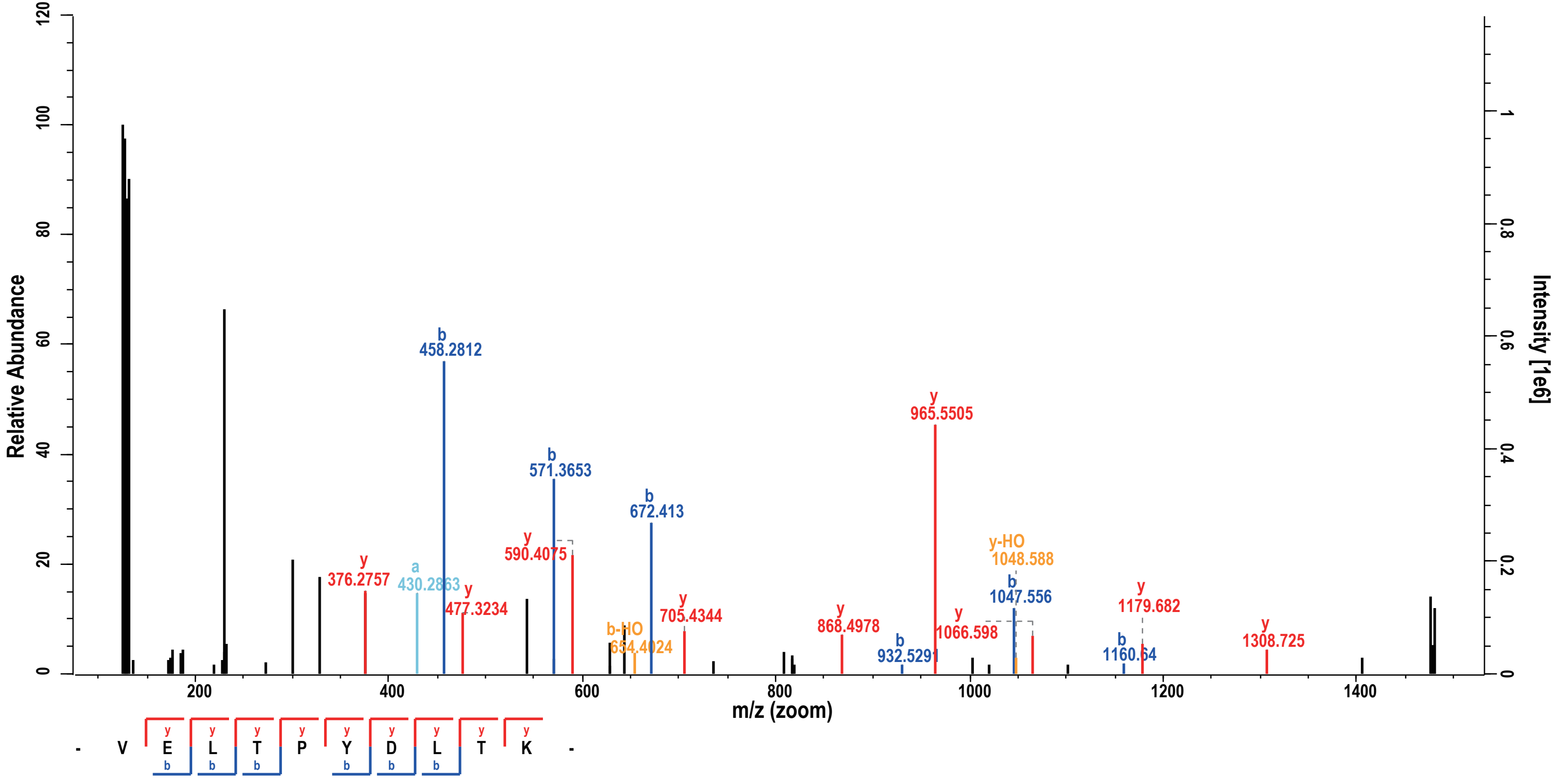

ssl3441 initiation factor IF-1

Raw File	Scan	Method	Score	m/z
HCC_TMT_3_F4_20180514	9450	FTMS; HCD	121.87	604.87

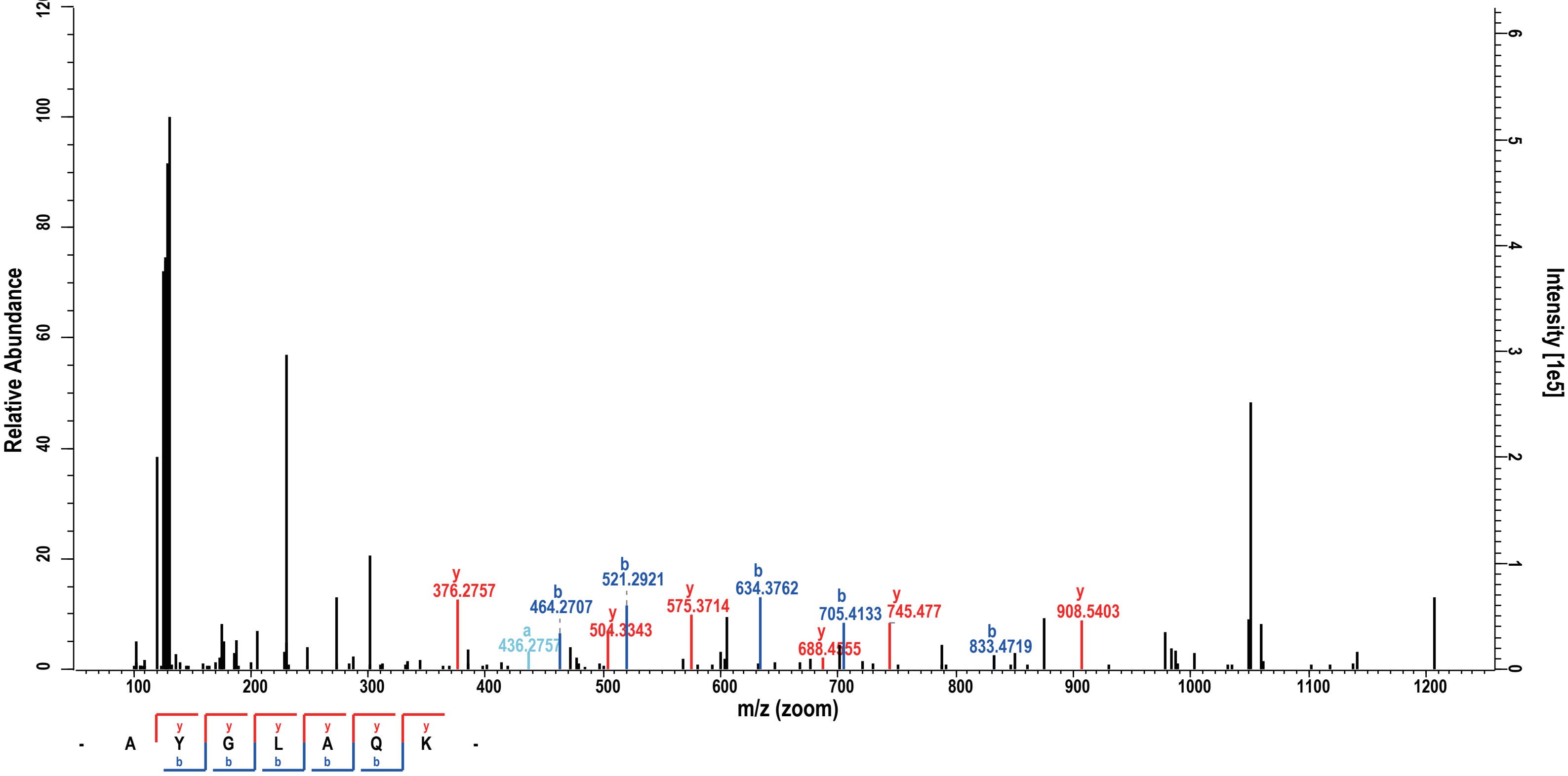

ssl5039 unknown protein

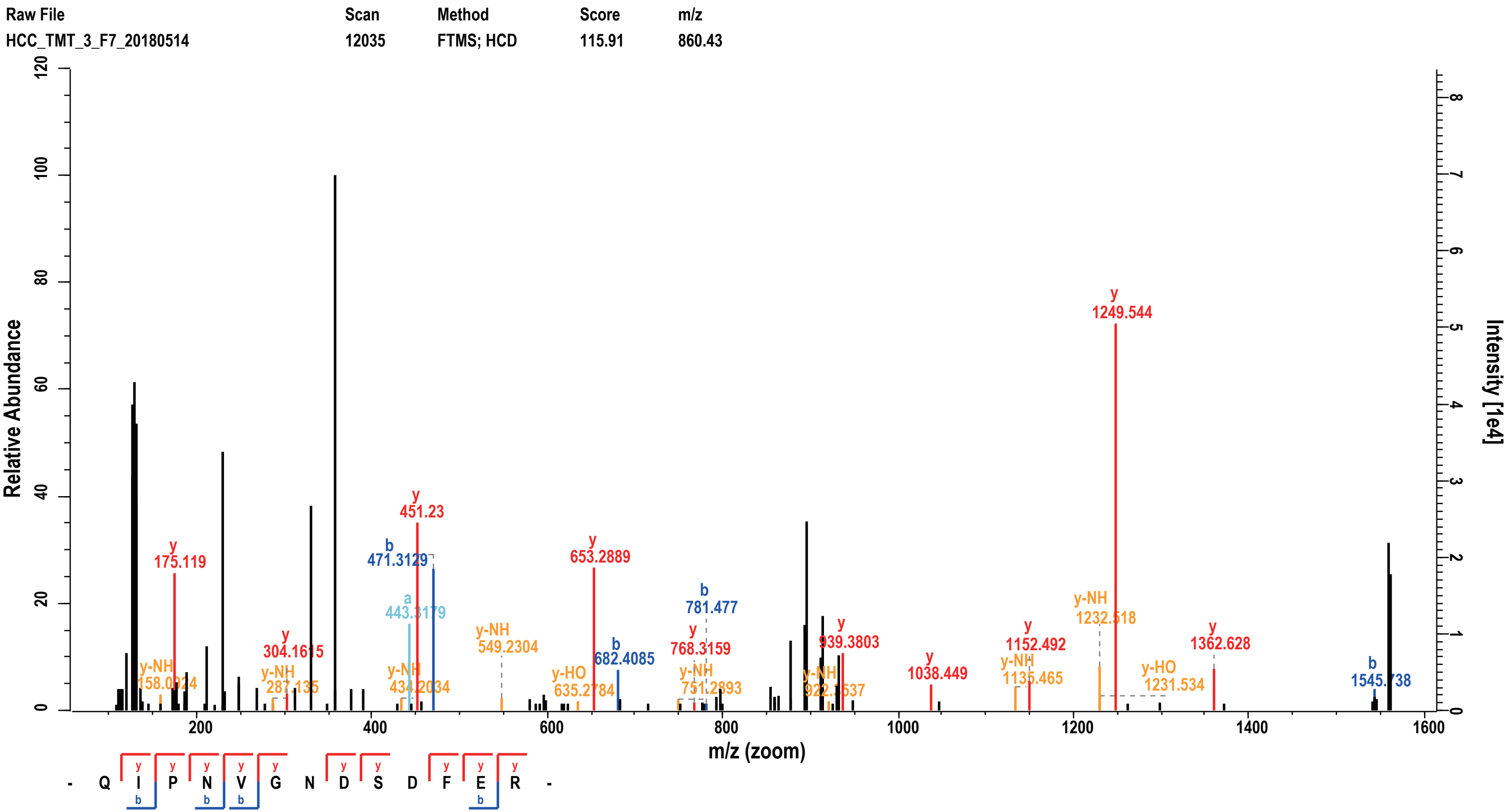

ssl7004 probable plasmid stability protein

Raw File	Scan	Method	Score	m/z
HCC_TMT_3_F13_20180514	1649	FTMS; HCD	112.41	547.79

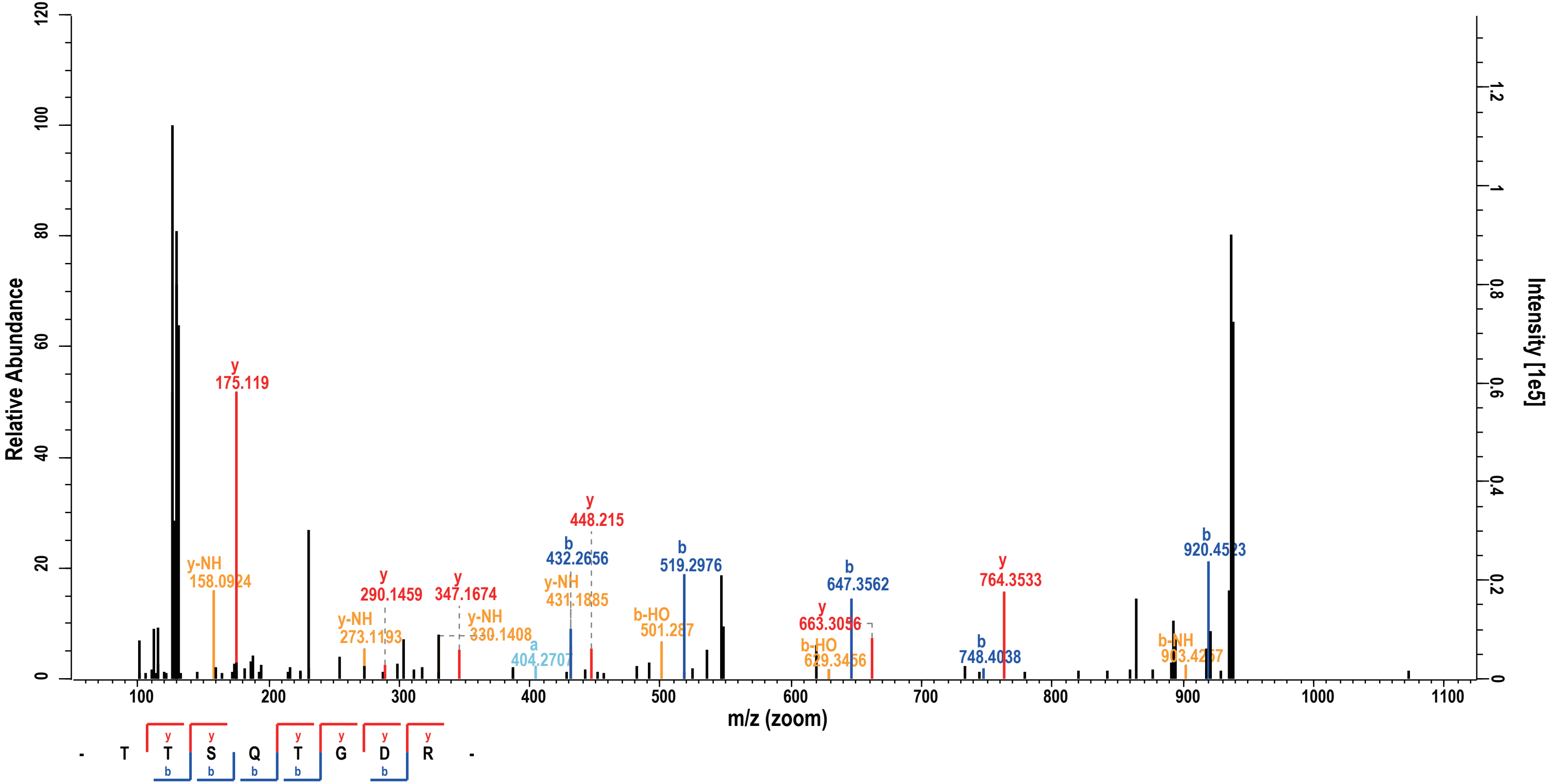

ssr0854 hypothetical protein

Raw File	Scan	Method	Score	m/z
HCC_TMT_2_F10_20180514	7736	FTMS; HCD	86.83	656.35

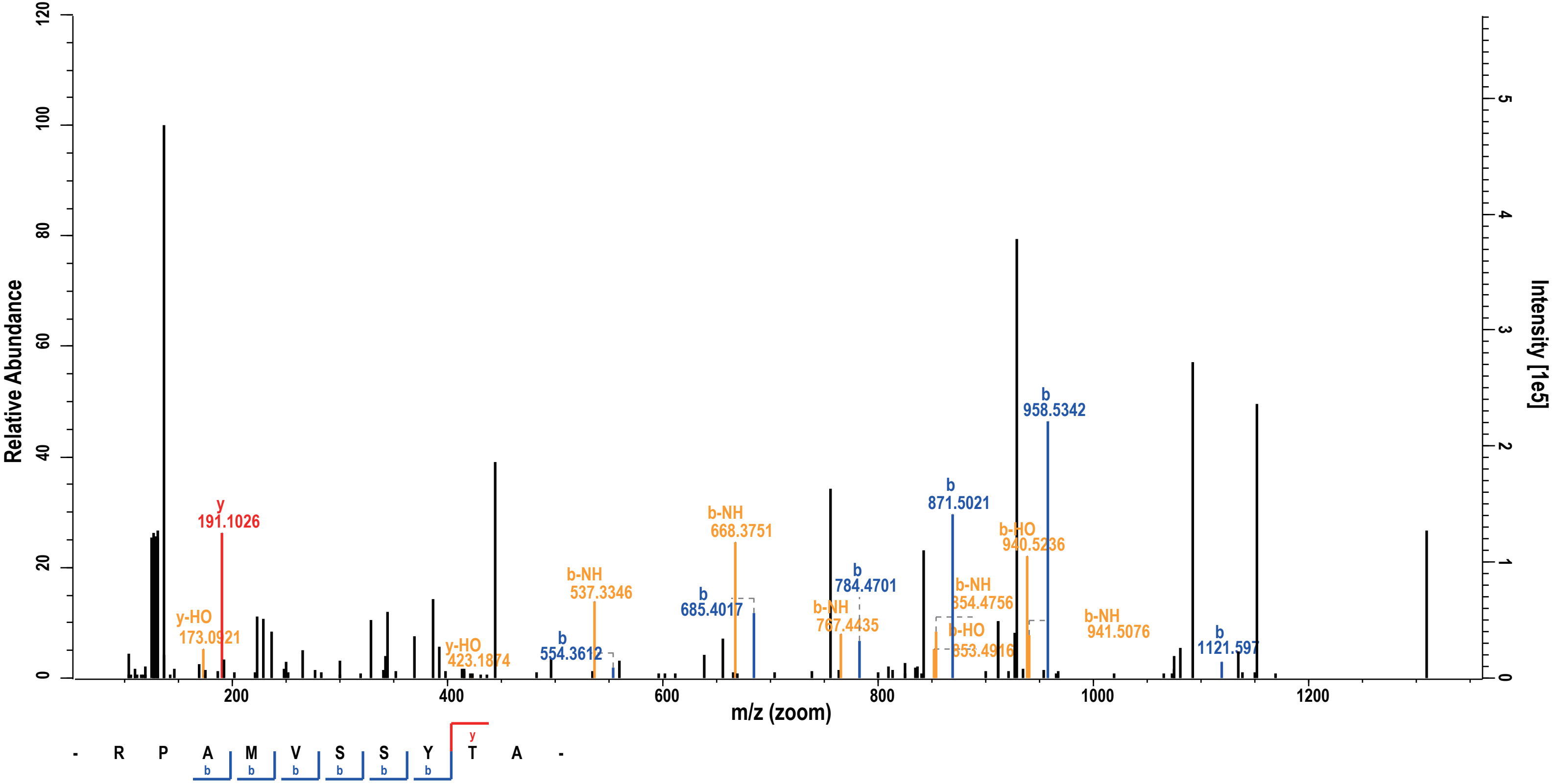

ssr1407 hypothetical protein

Raw File	Scan	Method	Score	m/z
HCC_TMT_1_F4_20180514	13753	FTMS; HCD	57.1	681.9

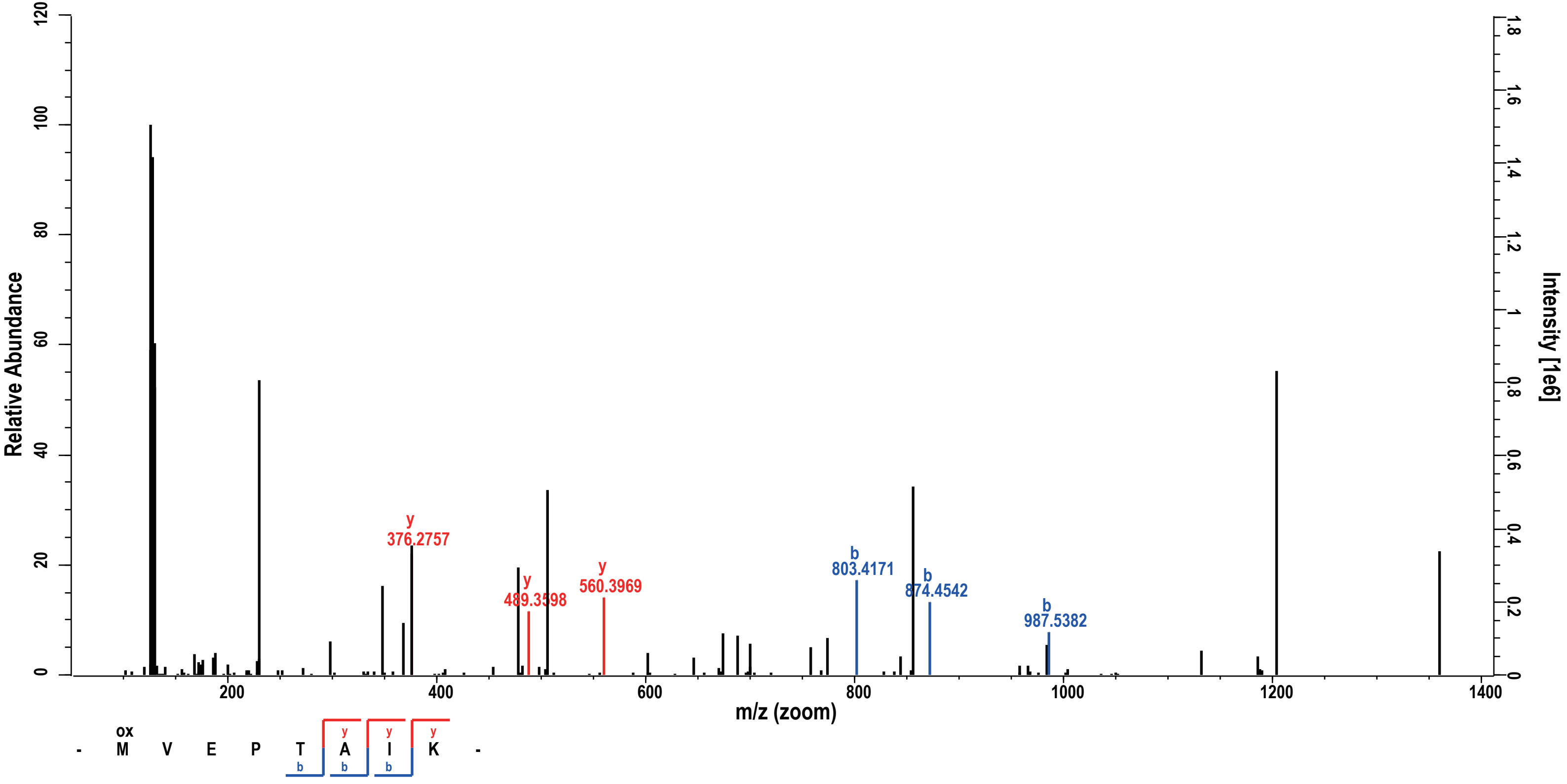

ssr2009 hypothetical protein

Raw File	Scan	Method	Score	m/z
HCC_TMT_2_F4_20180514	16920	FTMS; HCD	99.92	568.84

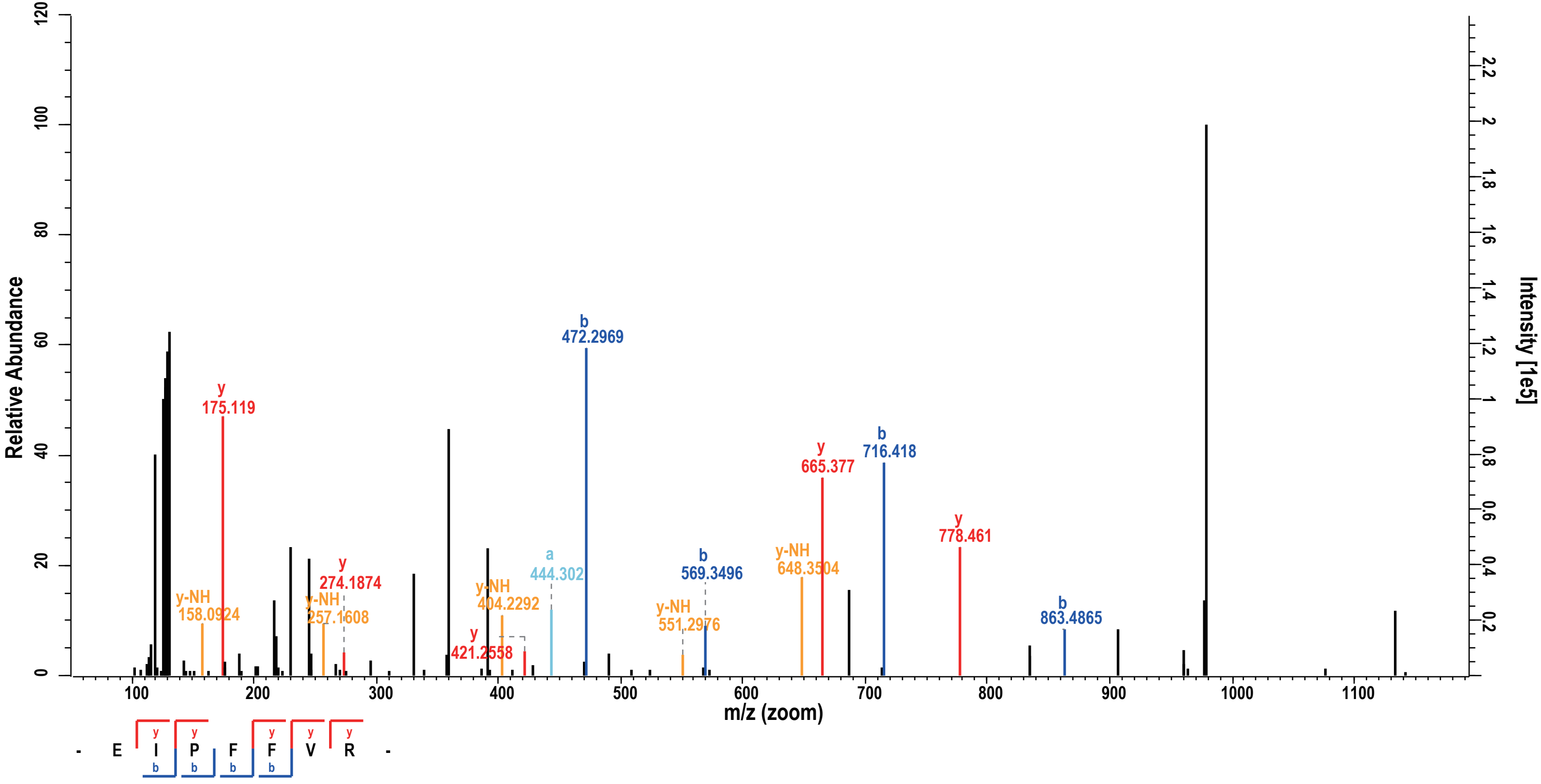

ssr2049 unknown protein

Raw File	Scan	Method	Score	m/z
HCC_TMT_1_F4_20180514	3000	FTMS; HCD	126.28	502.29

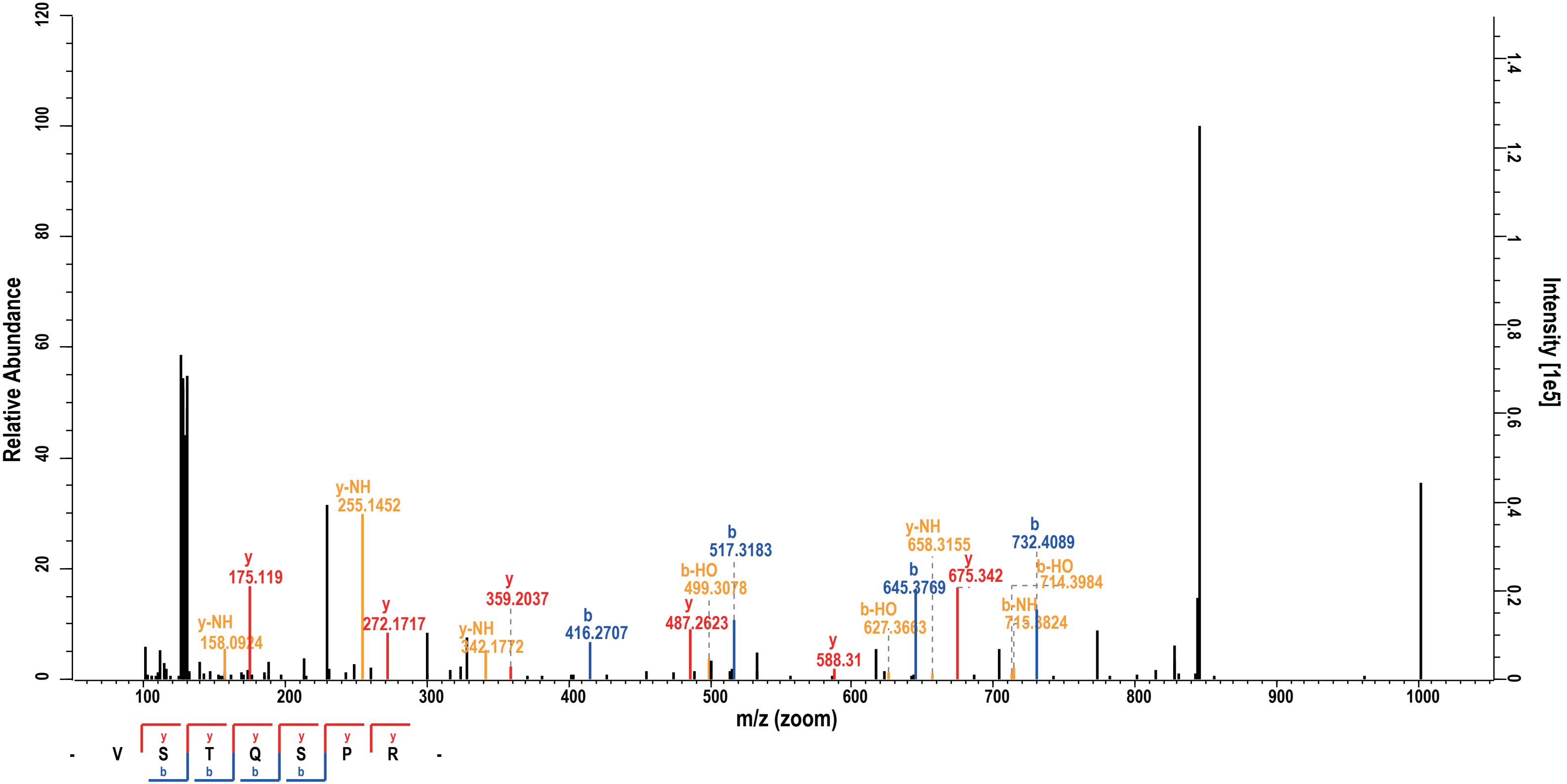

ssr2087 hypothetical protein

Raw File	Scan	Method	Score	m/z
HCC_TMT_1_F10_20180514	7579	FTMS; HCD	133.86	750.87

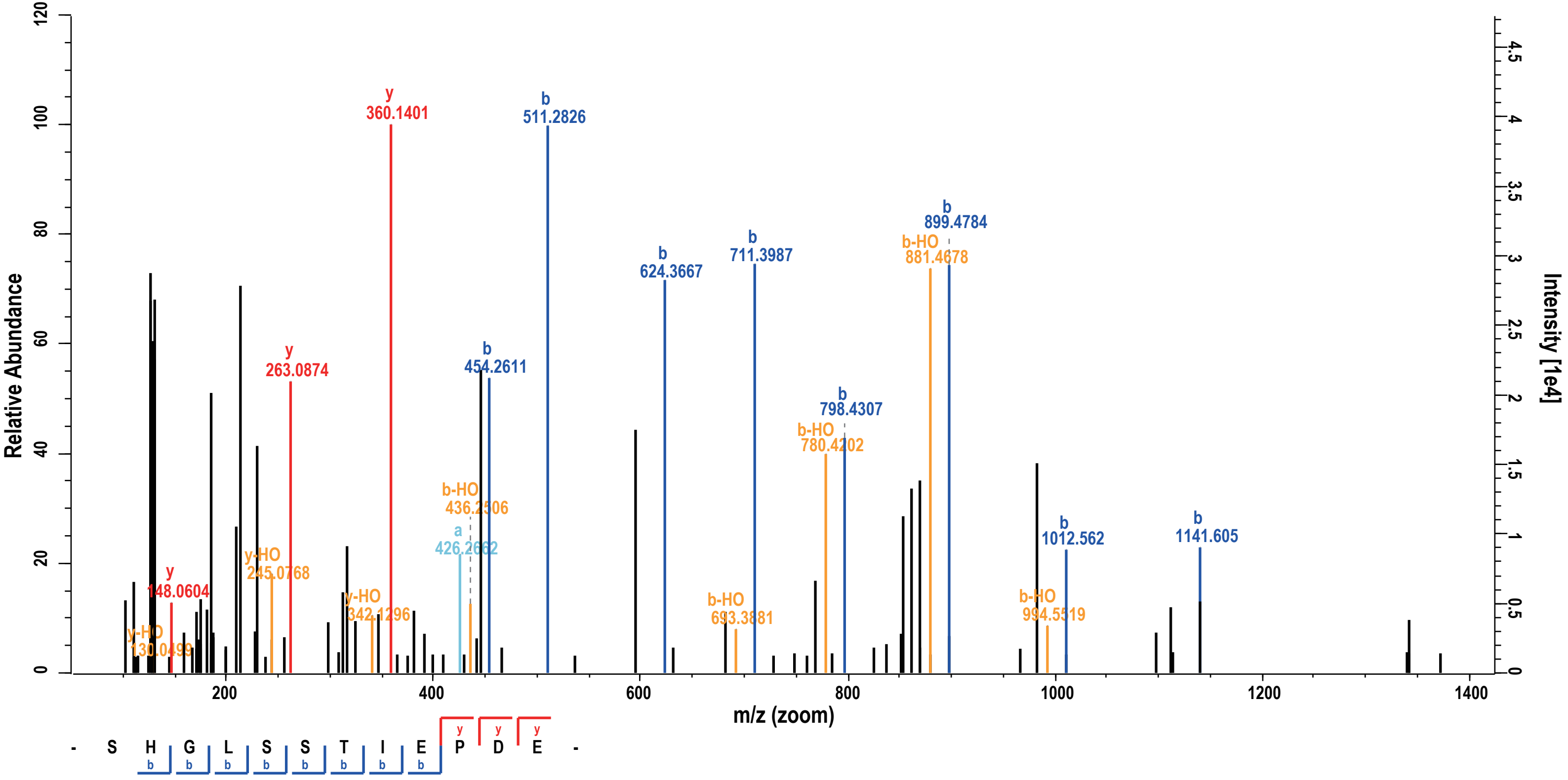

ssr2723 hypothetical protein

Raw File	Scan	Method	Score	m/z
HCC_TMT_3_F6_20180514	1812	FTMS; HCD	96.28	351.53

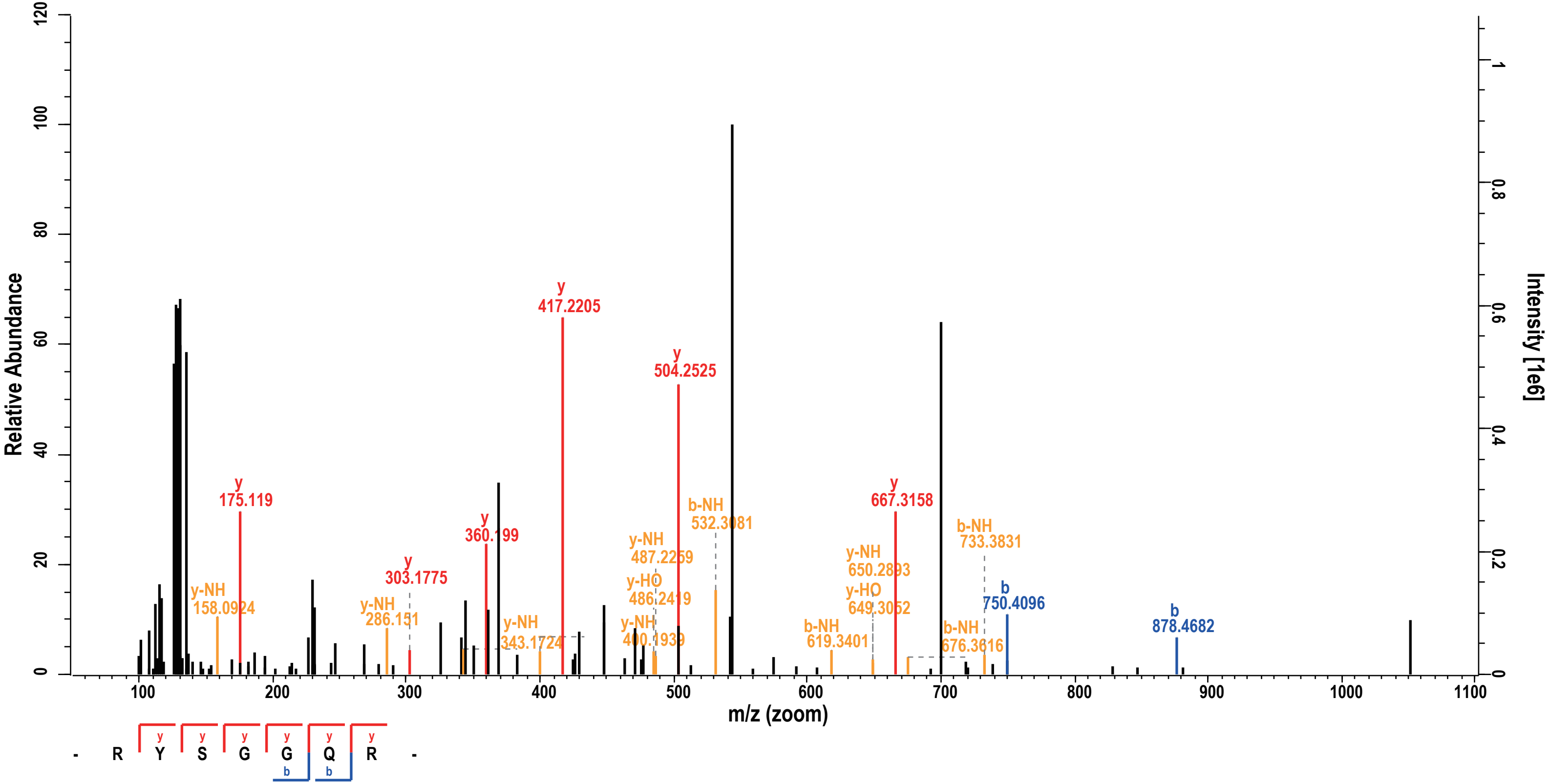

ssr3189 hypothetical protein

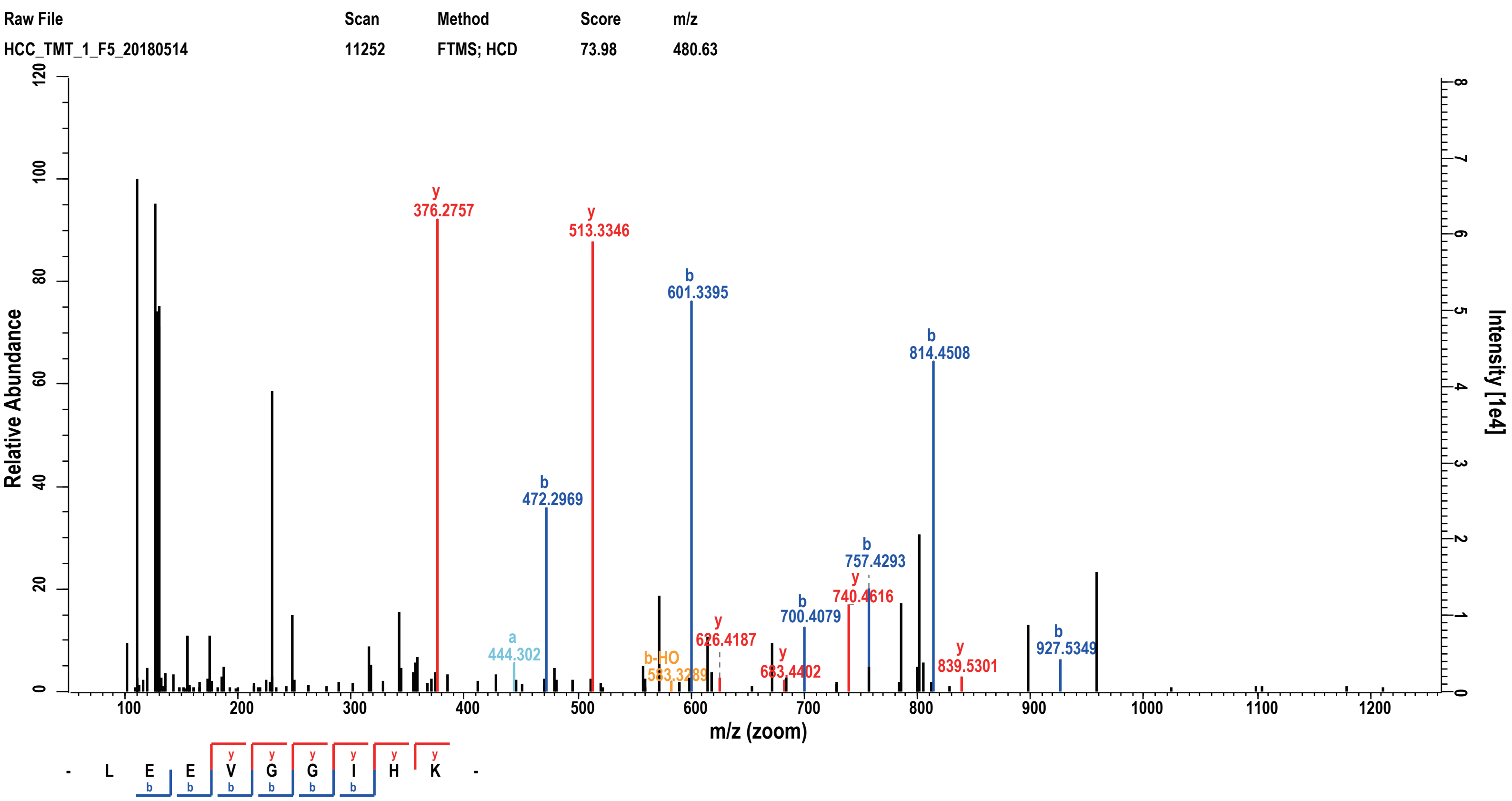

ssr9005 unknown protein [ORF-D]
